# Supplementary material for: Analysis of RecA-independent recombination events between short direct repeats related to a genomic island and to a plasmid in Escherichia coli K12
Source: PeerJ. 2017 May 9;5:e3293. doi: 10.7717/peerj.3293 (PMC5426353; doi:10.7717/peerj.3293)
Supplement: Supplemental Information 1 — Chromatograms of: (1) recombined sequences of the H47 GI model from a number of mutants affected in recombination functions, and (2) recombined sequences of the pUYFRT model. [file peerj-05-3293-s001.zip › raw material/29-ruvA_out1_FA.pdf]

Applied Biosystems

29-ruvA_out1_FA.ab1

29-ruvA_out1_FA

KB 1.2 KB.bcp

KB_3130_POP7_BDTv3.mob

Signal: G:227 A:128 T:180 C:155 AvgSig: 172

C#:1 W:E4 Plate Name:595

TS:21 CRL:105 QV20+:200

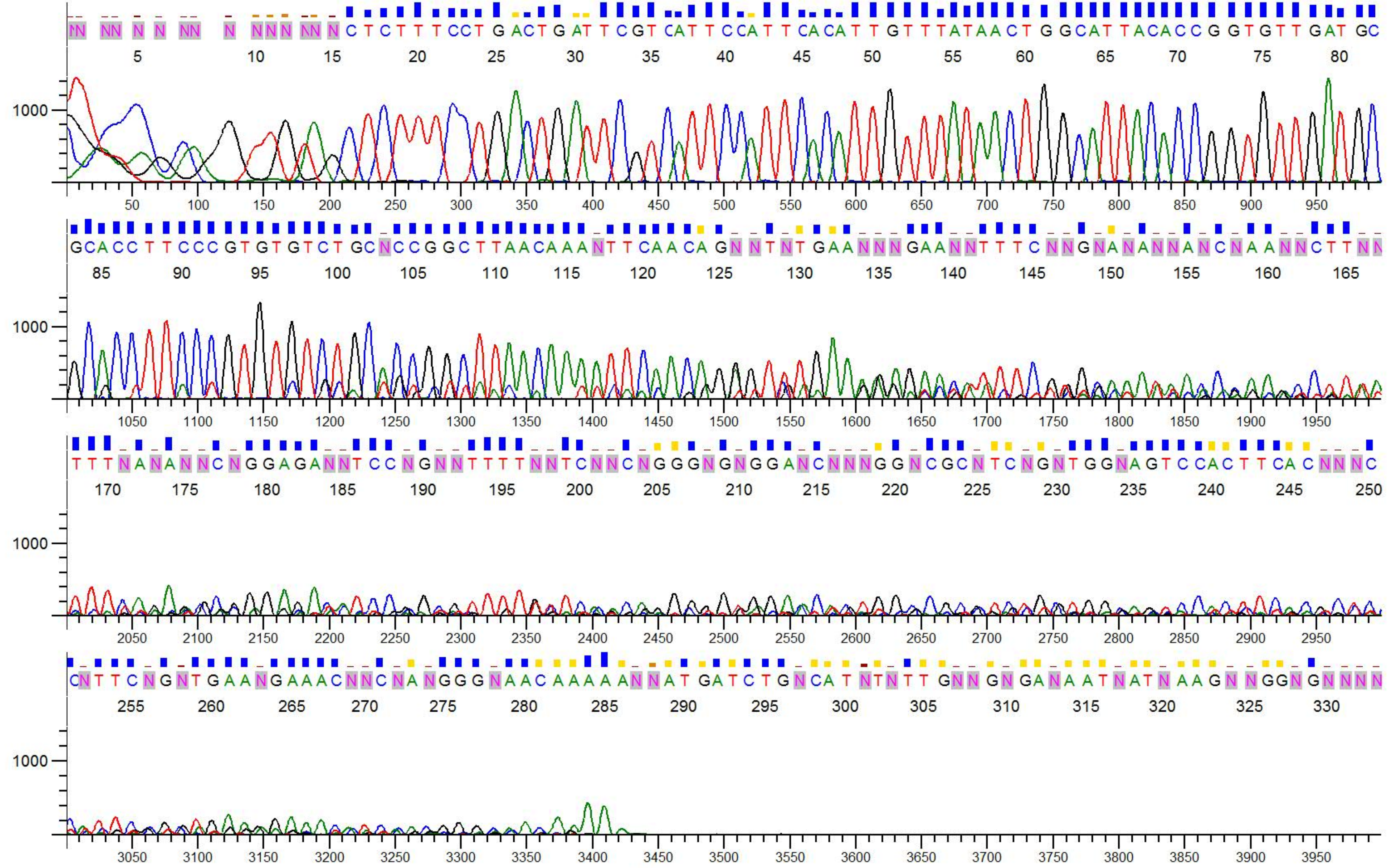

Inst Model/Name:3130/ABI3130GA-1352021

Pure Base QVs: 15 20

Mixed Base QVs: 10 16

Printed on: abr 04,2017 16:54:38 GMT

Sequence Scanner v1.0

Applied Biosystems

29-ruvA_out1_FA.ab1

29-ruvA_out1_FA

KB 1.2 KB.bcp

KB_3130_POP7_BDTv3.mob

Signal: G:227 A:128 T:180 C:155 AvgSig: 172

C#:1 W:E4 Plate Name:595

TS:21 CRL:105 QV20+:200

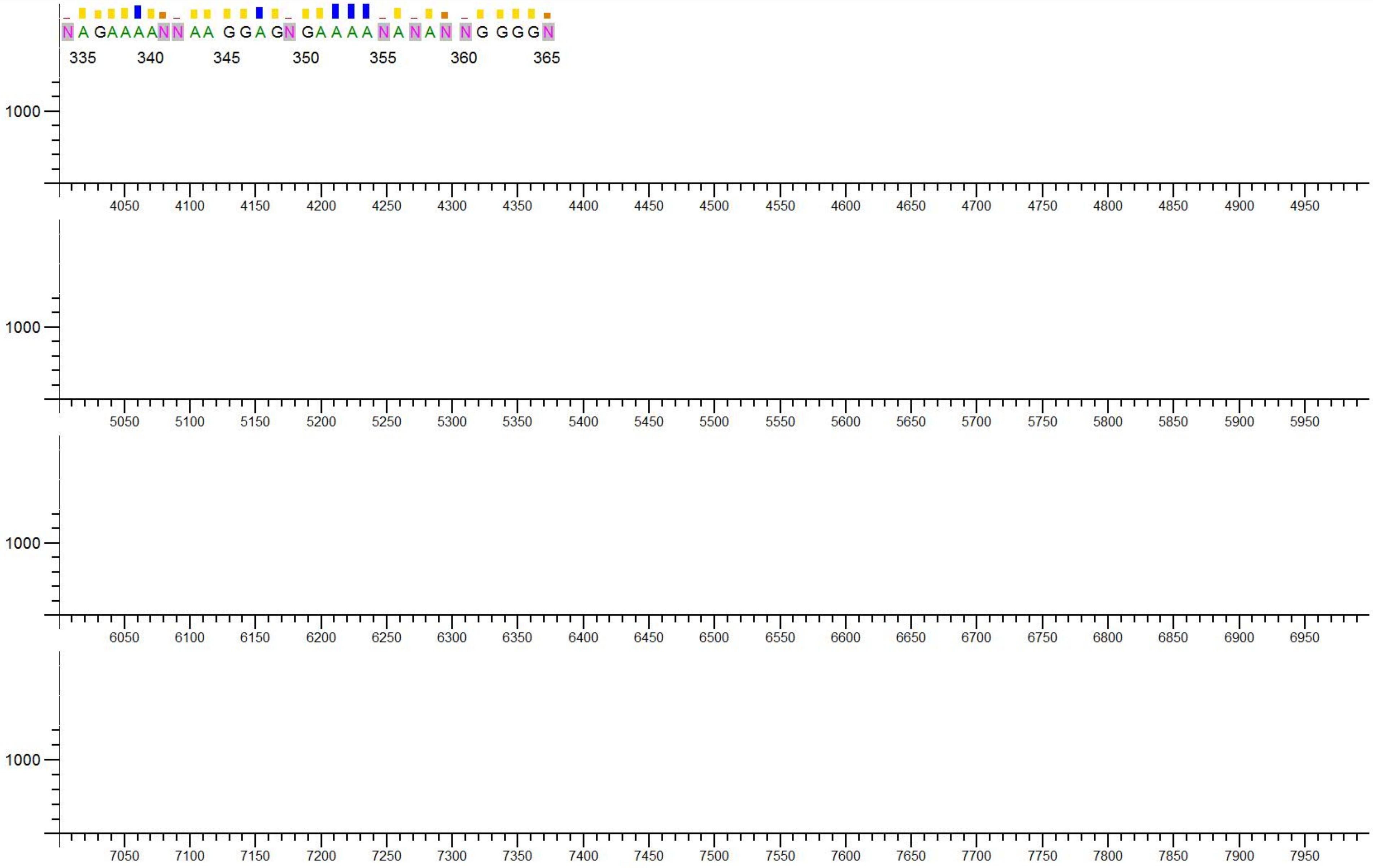

Inst Model/Name:3130/ABI3130GA-1352021

Pure Base QVs: 15 20

Mixed Base QVs: 10 16

Printed on: abr 04,2017 16:54:38 GMT

Sequence Scanner v1.0

Applied Biosystems

29-ruvA_out1_FA.ab1

29-ruvA_out1_FA

KB 1.2 KB.bcp

KB_3130_POP7_BDTv3.mob

Signal: G:227 A:128 T:180 C:155 AvgSig: 172

C#:1 W:E4 Plate Name:595

TS:21 CRL:105 QV20+:200

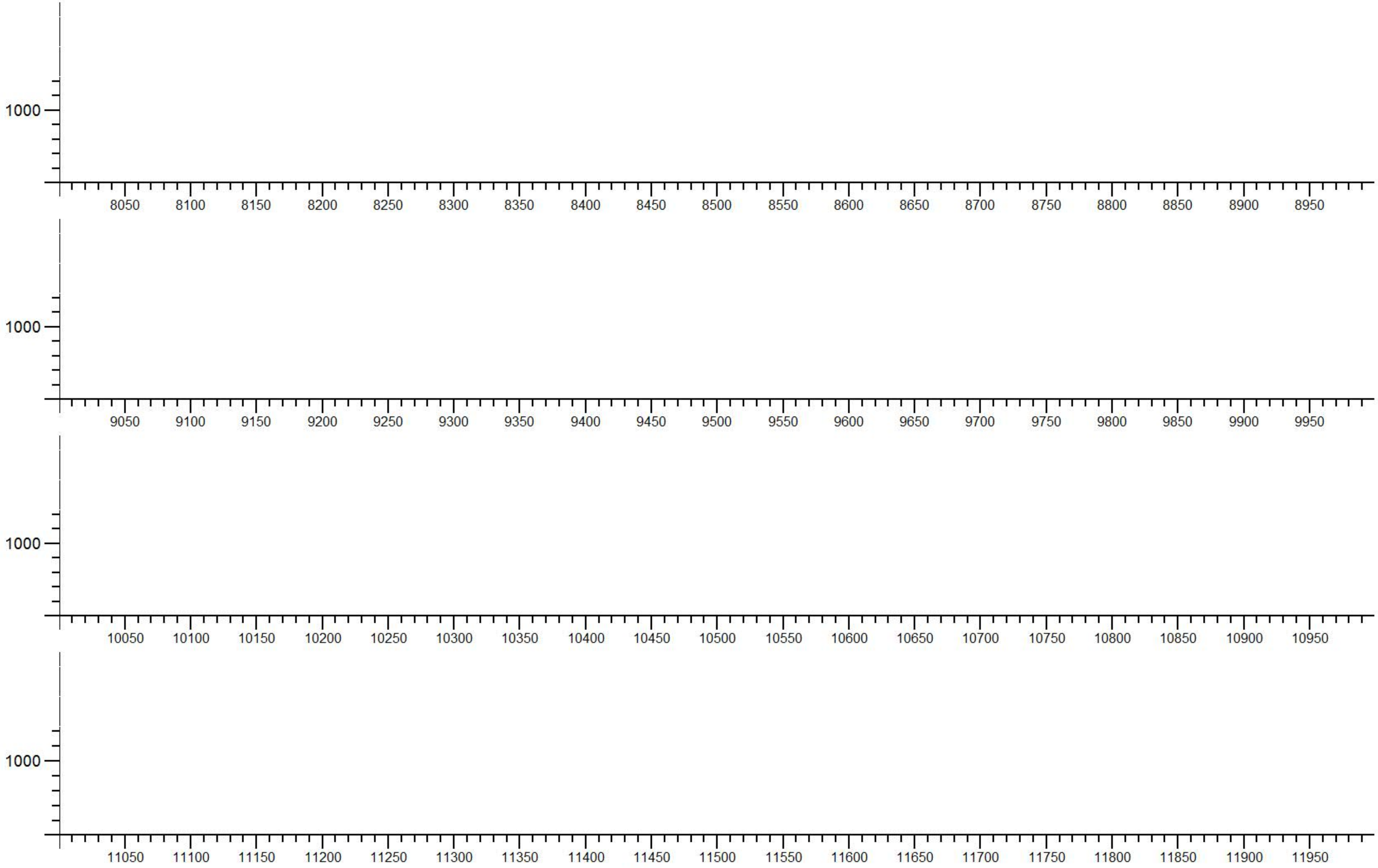

Inst Model/Name:3130/ABI3130GA-1352021

Pure Base QVs: 15 20

Mixed Base QVs: 10 16

Printed on: abr 04,2017 16:54:38 GMT

Sequence Scanner v1.0

29-ruvA_out1_FA.ab1

29-ruvA_out1_FA

KB 1.2 KB.bcp

KB_3130_POP7_BDTv3.mob

Signal: G:227 A:128 T:180 C:155 AvgSig: 172

C#:1 W:E4 Plate Name:595

TS:21 CRL:105 QV20+:200

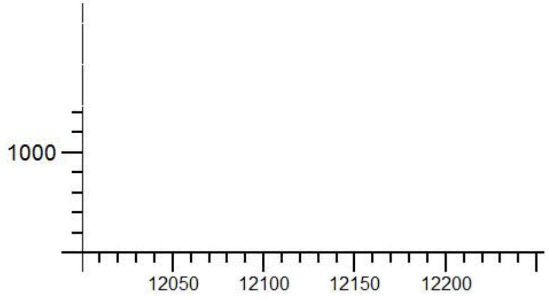

Inst Model/Name:3130/ABI3130GA-1352021

Sequence Scanner v1.0

Pure Base QVs:

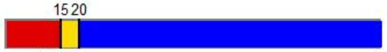

Mixed Base QVs:

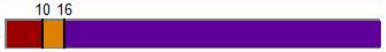

Printed on: abr 04,2017 16:54:38 GMT
