# Supplementary material for: Analysis of RecA-independent recombination events between short direct repeats related to a genomic island and to a plasmid in Escherichia coli K12
Source: PeerJ. 2017 May 9;5:e3293. doi: 10.7717/peerj.3293 (PMC5426353; doi:10.7717/peerj.3293)

Applied Biosystems

39-RecE_out1_FA.ab1

39-RecE_out1_FA

KB 1.2 KB.bcp

KB_3130_POP7_BDTv3.mob

Signal: G:160 A:103 T:139 C:177 AvgSig: 144

C#:3 W:G5 Plate Name:555

TS:21 CRL:108 QV20+:160

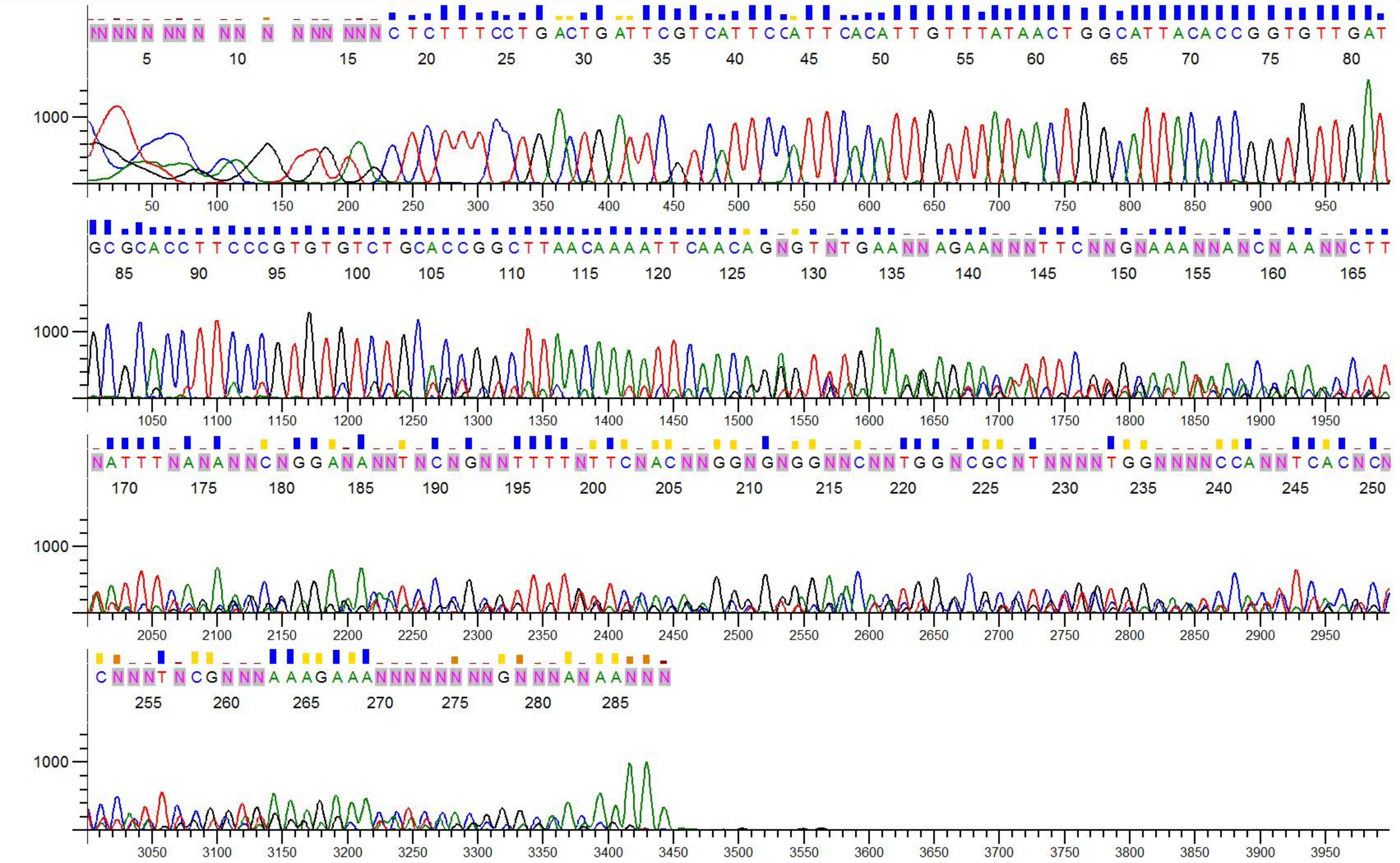

Inst Model/Name:3130/ABI3130GA-1352021

Sequence Scanner v1.0

Pure Base QVs: 15 20

Mixed Base QVs: 10 16

Printed on: abr 04,2017 16:59:13 GMT

Signal: G:160 A:103 T:139 C:177 AvgSig: 144

C#:3 W:G5 Plate Name:555

TS:21 CRL:108 QV20+:160

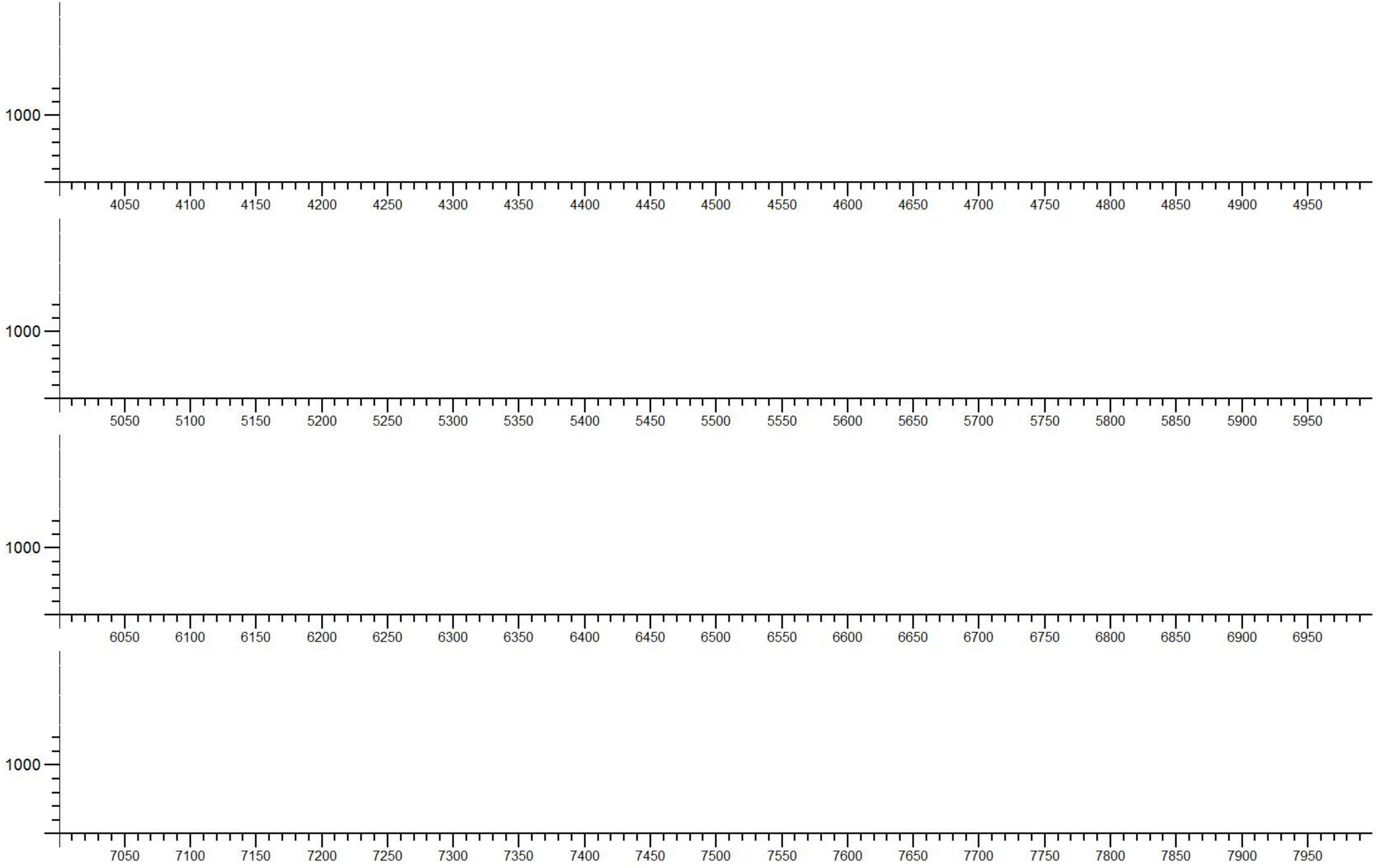

Signal: G:160 A:103 T:139 C:177 AvgSig: 144

C#:3 W:G5 Plate Name:555

TS:21 CRL:108 QV20+:160

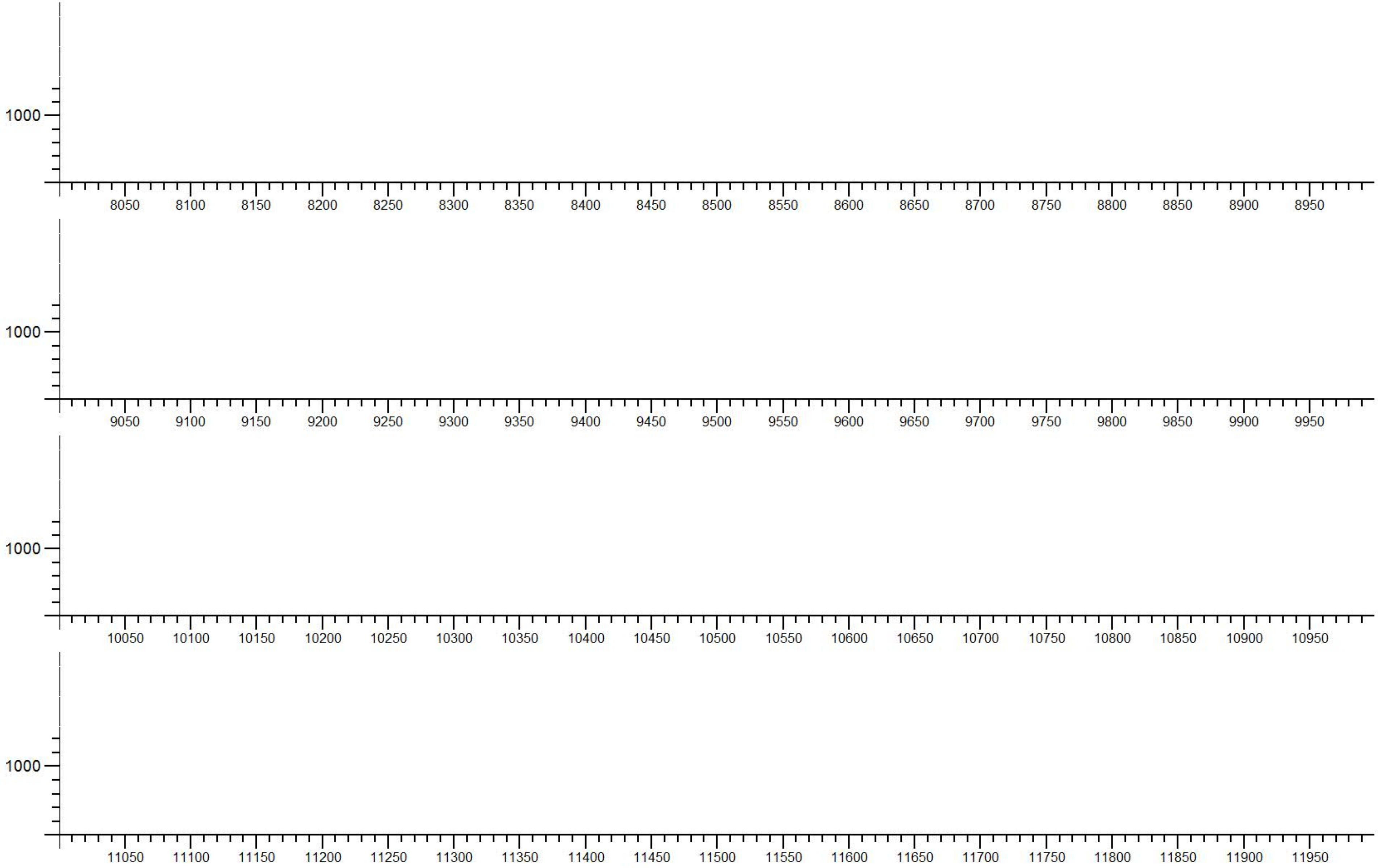

Signal: G:160 A:103 T:139 C:177 AvgSig: 144

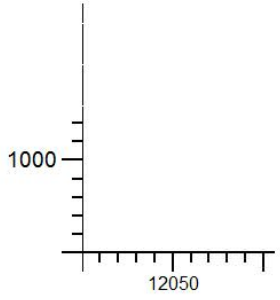

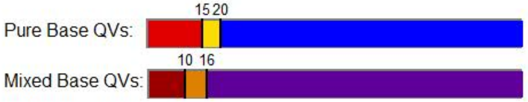

Supplement: Supplemental Information 1 — Chromatograms of: (1) recombined sequences of the H47 GI model from a number of mutants affected in recombination functions, and (2) recombined sequences of the pUYFRT model. [file peerj-05-3293-s001.zip › raw material/39-RecE_out1_FA.pdf]
