# Supplementary material for: Analysis of RecA-independent recombination events between short direct repeats related to a genomic island and to a plasmid in Escherichia coli K12
Source: PeerJ. 2017 May 9;5:e3293. doi: 10.7717/peerj.3293 (PMC5426353; doi:10.7717/peerj.3293)

Applied Biosystems

53-YbcK_out1_FA.ab1

53-YbcK_out1_FA

KB 1.2 KB.bcp

KB_3130_POP7_BDTv3.mob

Signal: G:2018 A:1553 T:1969 C:1799 AvgSig: 1834

C#:1 W:E7 Plate Name:490

TS:15 CRL:27 QV20+:197

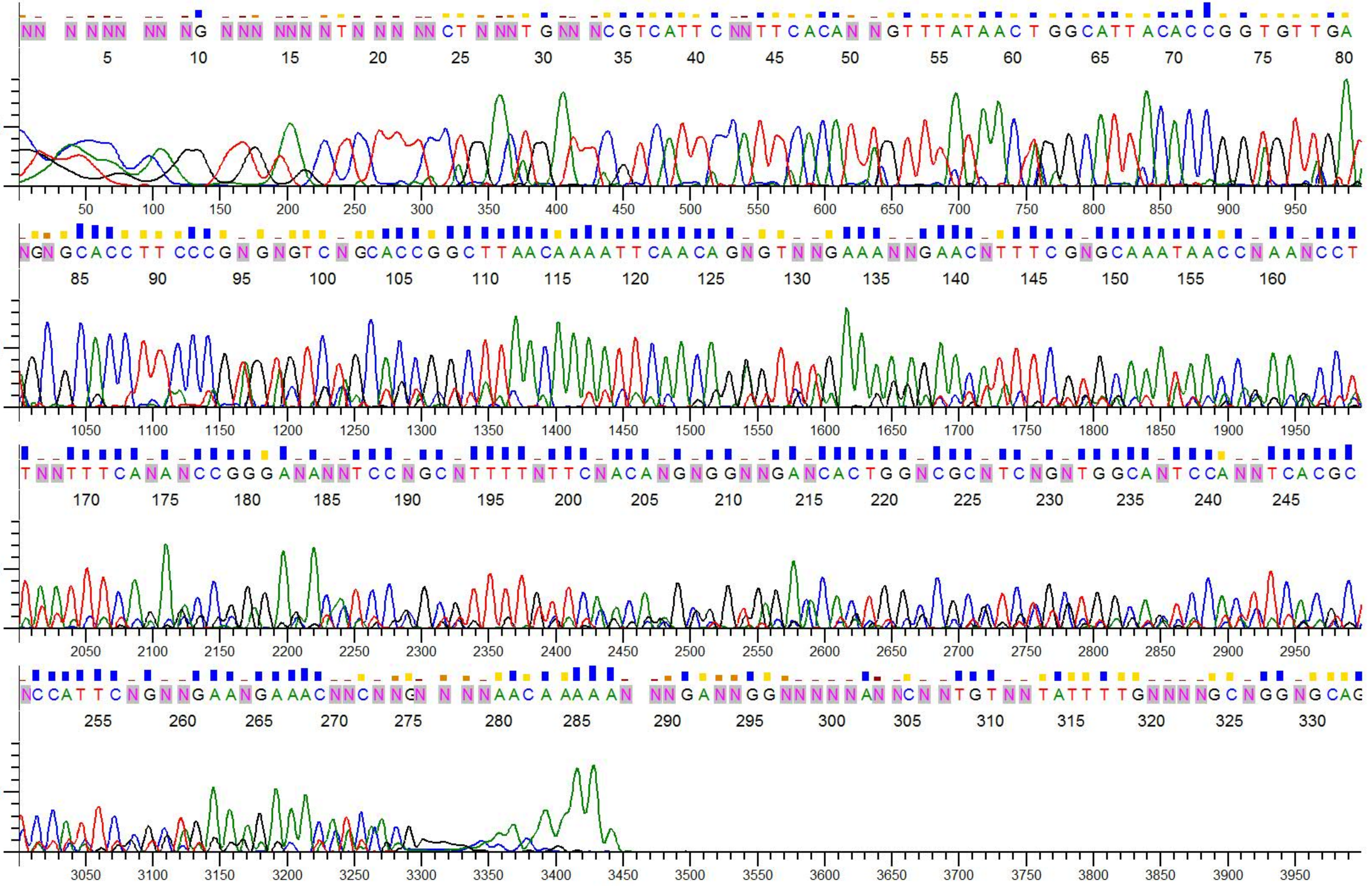

Inst Model/Name:3130/ABI3130GA-1352021

Sequence Scanner v1.0

Pure Base QVs: 15 20

Mixed Base QVs: 10 16

Printed on: abr 04,2017 18:10:12 GMT

53-YbcK_out1_FA.ab1 KB 1.2 KB.bcp

53-YbcK_out1_FA KB_3130_POP7_BDTv3.mob

Signal: G:2018 A:1553 T:1969 C:1799 AvgSig: 1834 C#:1 W:E7 Plate Name:490 TS:15 CRL:27 QV20+:197

ACNCACGGGNNGGNGNGCNTNNANACNGGNGTNATGNNAGTNATAAANNNTGTGAATGGAANGANNANTCNGTNANGNNAGAG

335 340 345 350 355 360 365 370 375 380 385 390 395 400 405 410 415

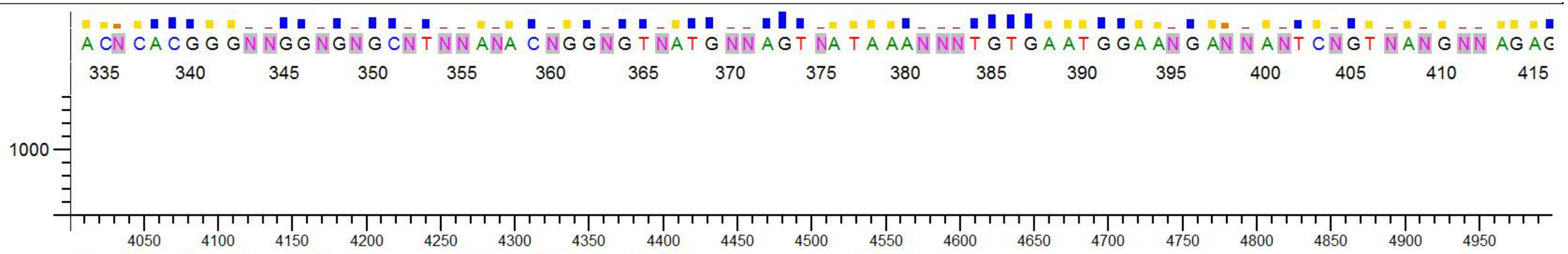

CNNCNNCNNGNNGGNTGNNNGNGNNGAGTNNGNANNNNNNNNNNCGGNNN

420 425 430 435 440 445 450 455 460 465

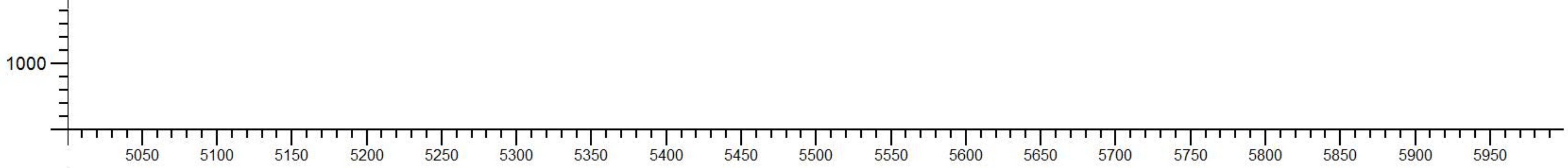

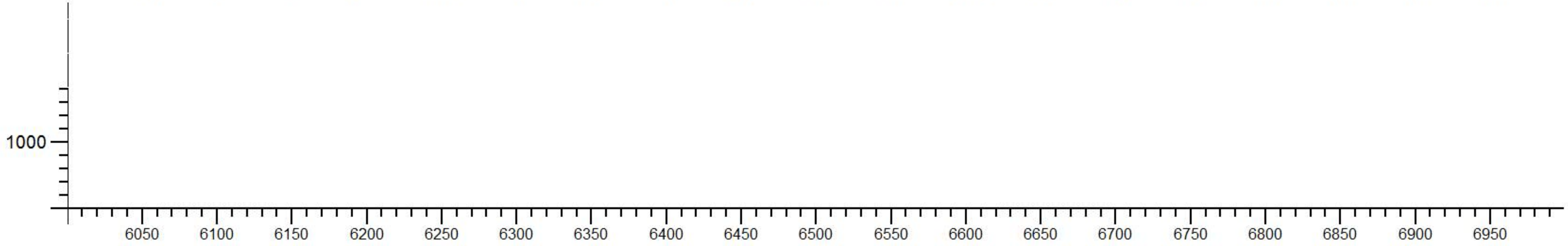

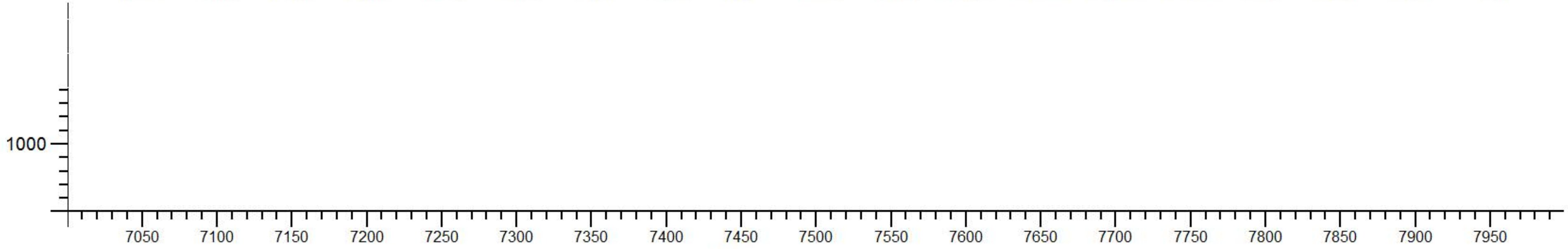

Inst Model/Name:3130/ABI3130GA-1352021

Pure Base QVs: 15 20

Mixed Base QVs: 10 16

Printed on: abr 04,2017 18:10:12 GMT

Sequence Scanner v1.0

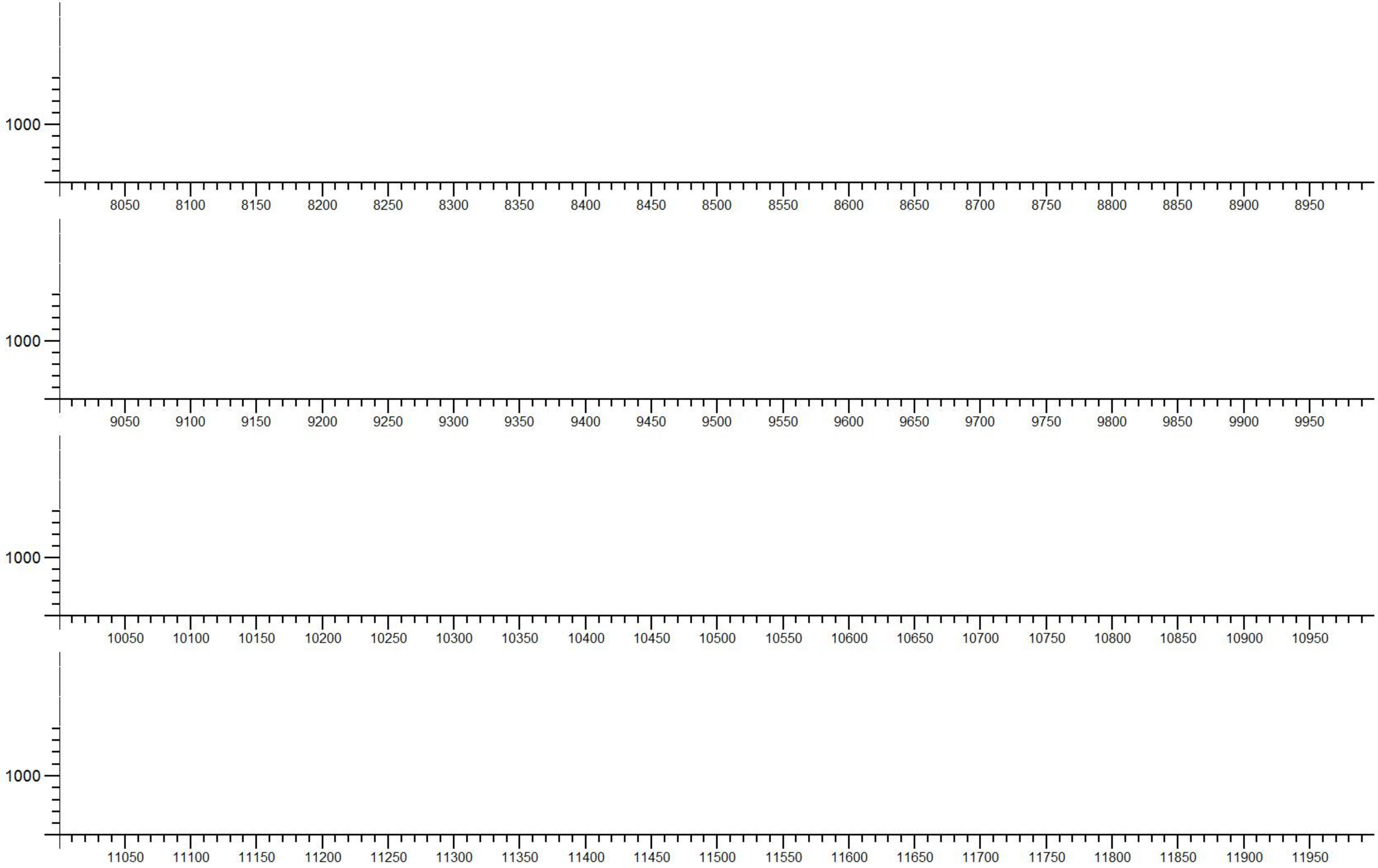

Pure Base QVs:
Mixed Base QVs:

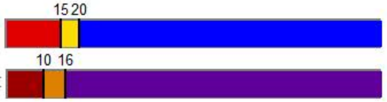

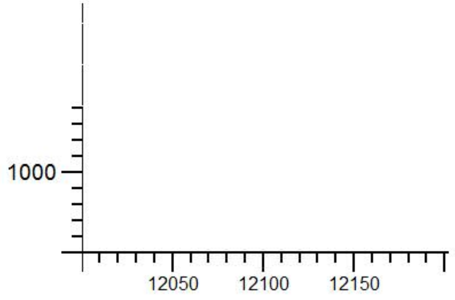

Inst Model/Name:3130/ABI3130GA-1352021

Sequence Scanner v1.0

Pure Base QVs:

Mixed Base QVs:

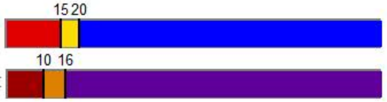

Supplement: Supplemental Information 1 — Chromatograms of: (1) recombined sequences of the H47 GI model from a number of mutants affected in recombination functions, and (2) recombined sequences of the pUYFRT model. [file peerj-05-3293-s001.zip › raw material/53-YbcK_out1_FA.pdf]
