# Supplementary material for: Analysis of RecA-independent recombination events between short direct repeats related to a genomic island and to a plasmid in Escherichia coli K12
Source: PeerJ. 2017 May 9;5:e3293. doi: 10.7717/peerj.3293 (PMC5426353; doi:10.7717/peerj.3293)
Supplement: Supplemental Information 1 — Chromatograms of: (1) recombined sequences of the H47 GI model from a number of mutants affected in recombination functions, and (2) recombined sequences of the pUYFRT model. [file peerj-05-3293-s001.zip › raw material/61-pinQpinR_out1_FA.pdf]

Applied Biosystems

61-pinQpinR_out1_FA.ab1

61-PinQpinR_out1_FA

KB 1.2 KB.bcp

KB_3130_POP7_BDTv3.mob

Signal: G:1016 A:558 T:770 C:665 AvgSig: 752

C#:1 W:E8 Plate Name:490

TS:18 CRL:102 QV20+:250

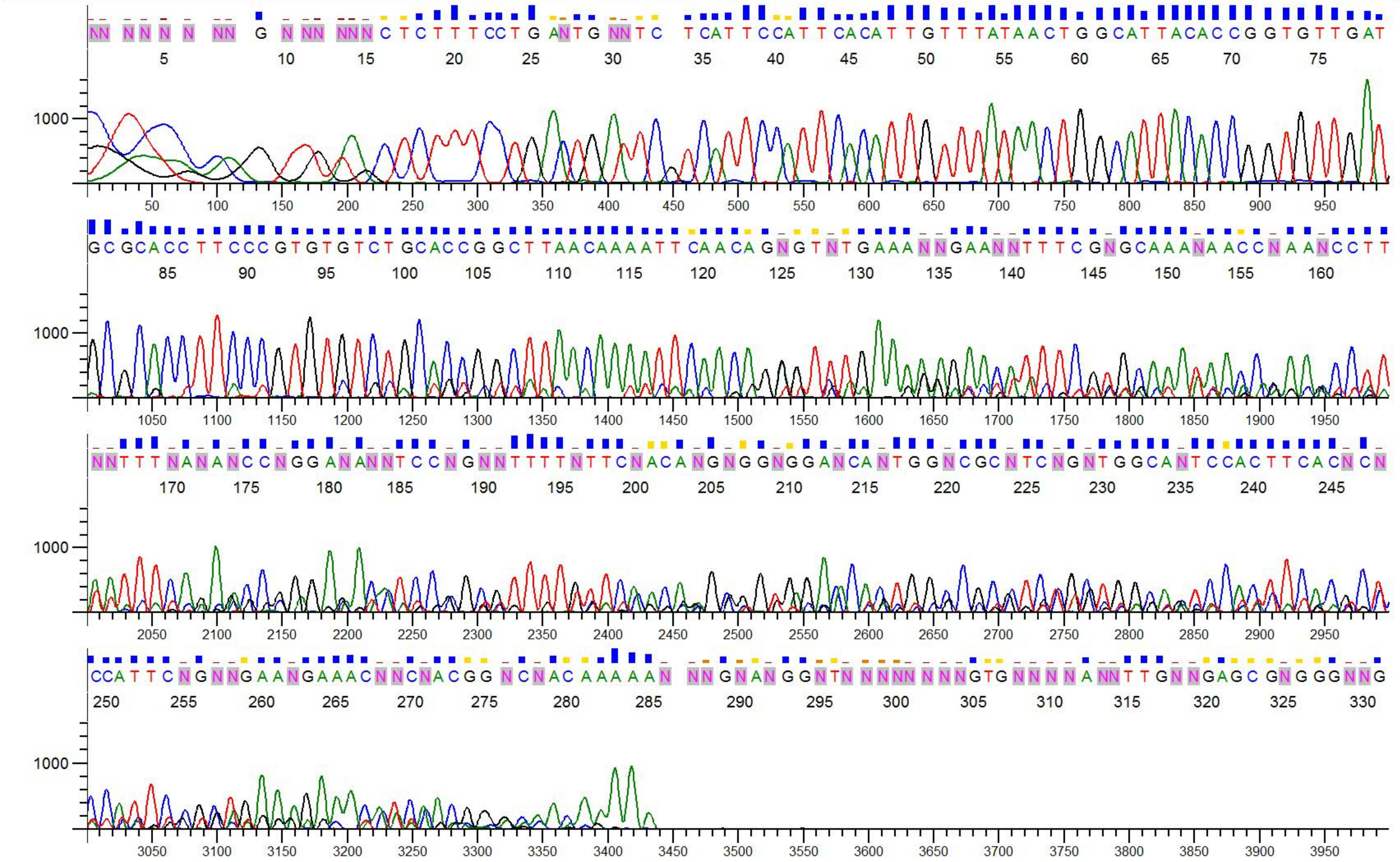

Inst Model/Name:3130/ABI3130GA-1352021

Pure Base QVs: 15 20

Mixed Base QVs: 10 16

Printed on: abr 04,2017 18:25:05 GMT

Sequence Scanner v1.0

ACANTNGGGNGGGNGNNCNTNTNNACNGNNGTNNNGTNANTNNTAAANNNTGTNNATNGAATGACGAGTCNNNCNGGNNANAG

335 340 345 350 355 360 365 370 375 380 385 390 395 400 405 410

1000

4050 4100 4150 4200 4250 4300 4350 4400 4450 4500 4550 4600 4650 4700 4750 4800 4850 4900 4950

CNCNANCCNGNNGNCTGNNNGGAGNGNGTANNNANGANATGNACNGNN

420 425 430 435 440 445 450 455 460

1000

5050 5100 5150 5200 5250 5300 5350 5400 5450 5500 5550 5600 5650 5700 5750 5800 5850 5900 5950

1000

6050 6100 6150 6200 6250 6300 6350 6400 6450 6500 6550 6600 6650 6700 6750 6800 6850 6900 6950

1000

7050 7100 7150 7200 7250 7300 7350 7400 7450 7500 7550 7600 7650 7700 7750 7800 7850 7900 7950

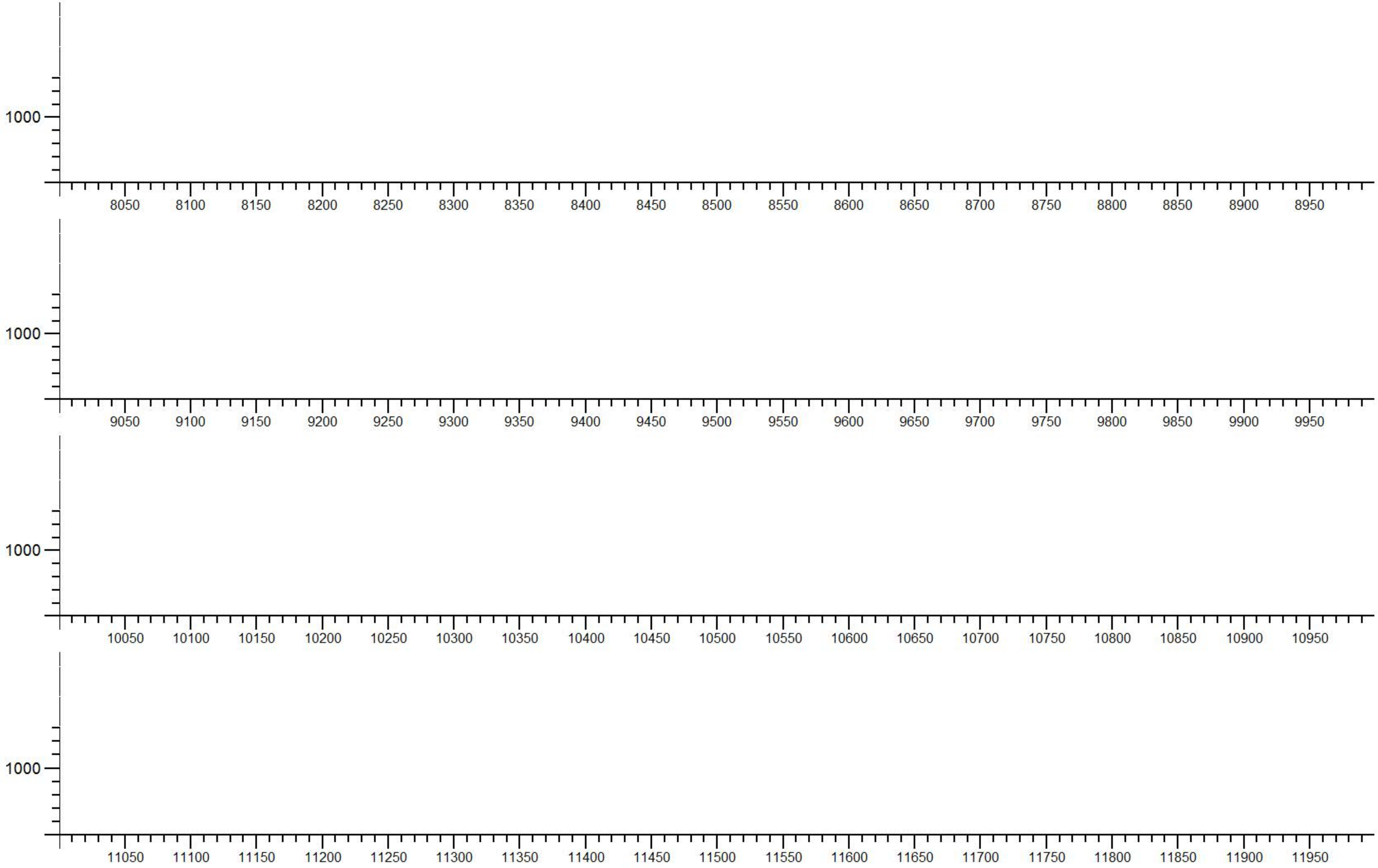
1000
8050 8100 8150 8200 8250 8300 8350 8400 8450 8500 8550 8600 8650 8700 8750 8800 8850 8900 8950
1000
9050 9100 9150 9200 9250 9300 9350 9400 9450 9500 9550 9600 9650 9700 9750 9800 9850 9900 9950
1000
10050 10100 10150 10200 10250 10300 10350 10400 10450 10500 10550 10600 10650 10700 10750 10800 10850 10900 10950
1000
11050 11100 11150 11200 11250 11300 11350 11400 11450 11500 11550 11600 11650 11700 11750 11800 11850 11900 11950

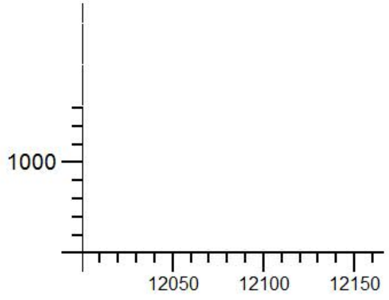
1000
12050
12100
12150
