# Supplementary figures and images for: Analysis of RecA-independent recombination events between short direct repeats related to a genomic island and to a plasmid in Escherichia coli K12
Source: PeerJ. 2017 May 9;5:e3293. doi: 10.7717/peerj.3293 (PMC5426353; doi:10.7717/peerj.3293)

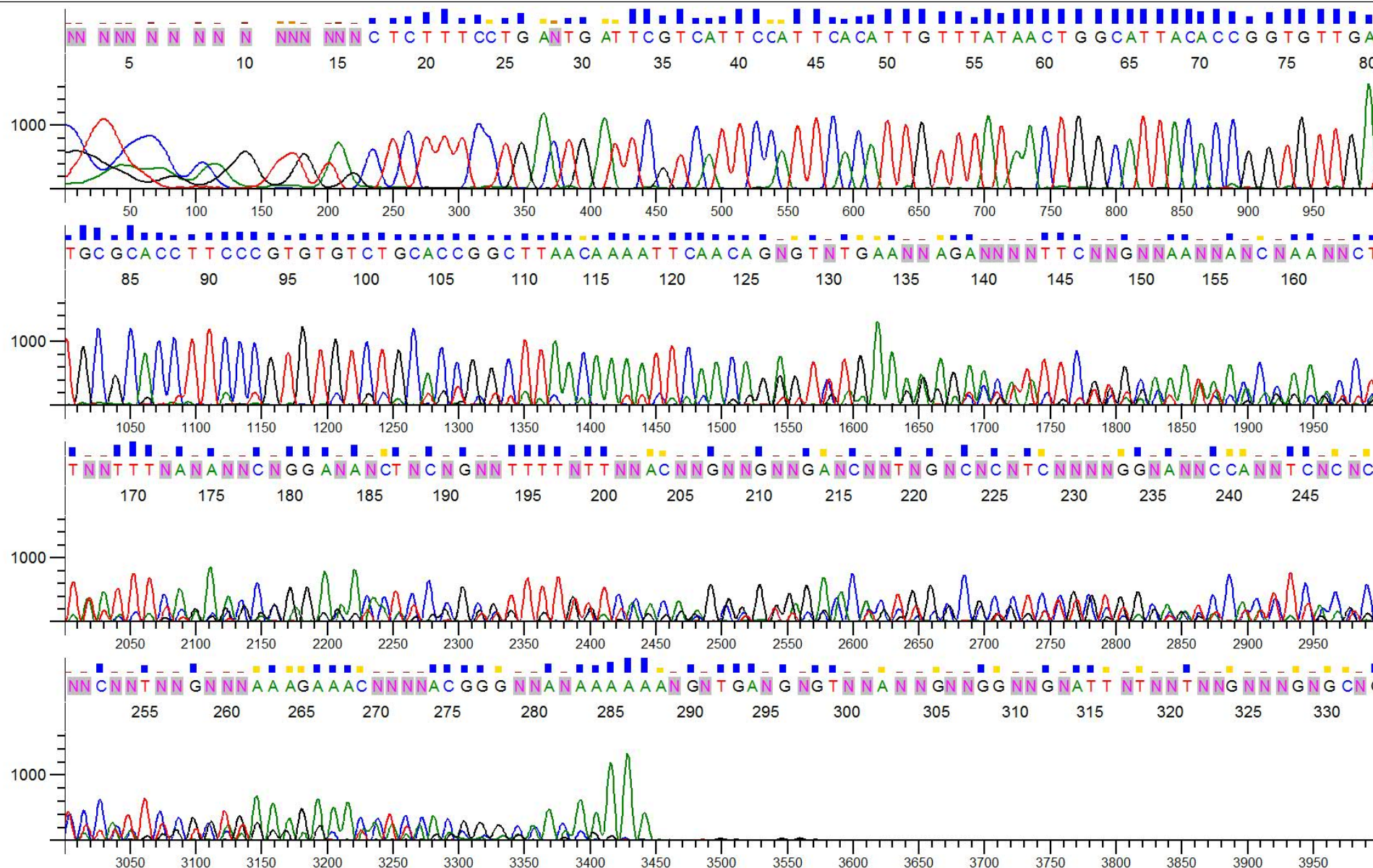

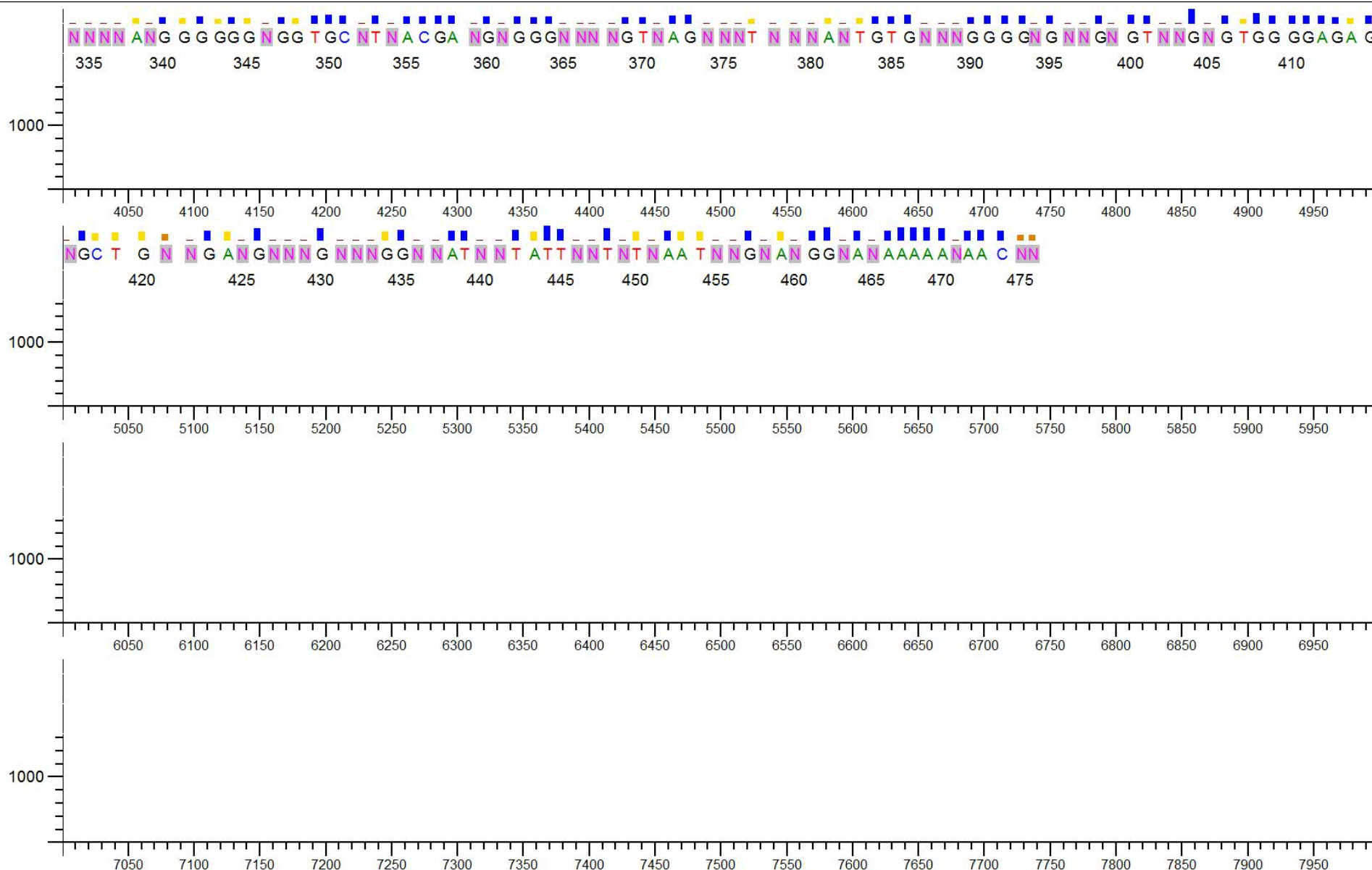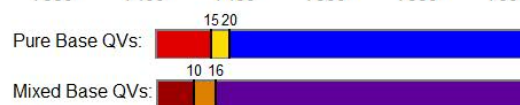

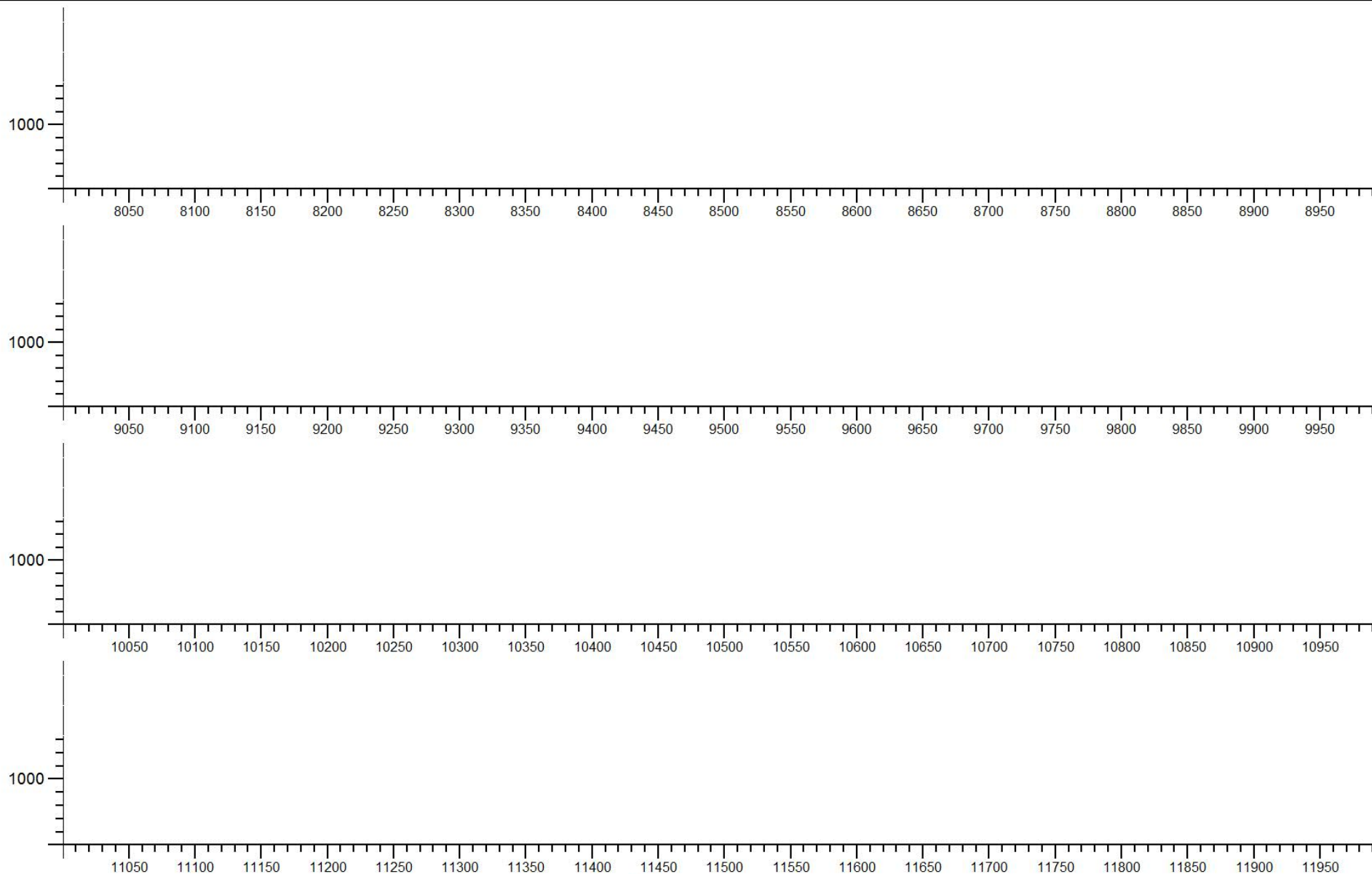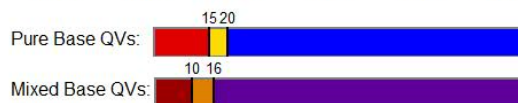

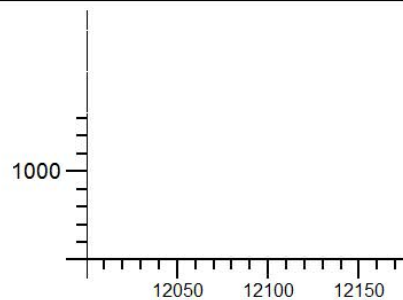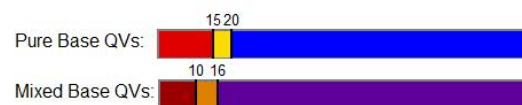

Supplement: Supplemental Information 1 — Chromatograms of: (1) recombined sequences of the H47 GI model from a number of mutants affected in recombination functions, and (2) recombined sequences of the pUYFRT model. [file peerj-05-3293-s001.zip › raw material/11-RecD_out1_FA.pdf]

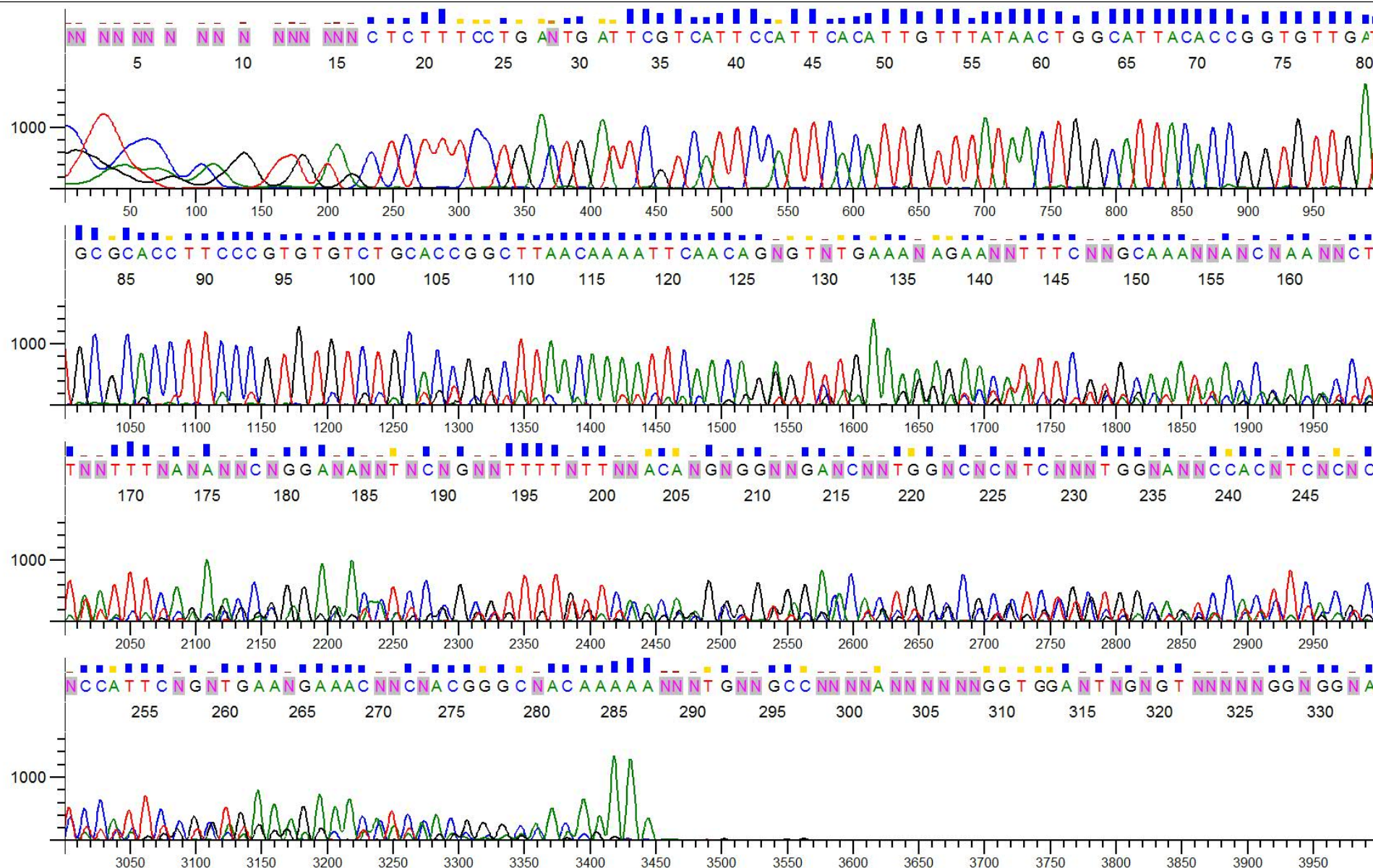

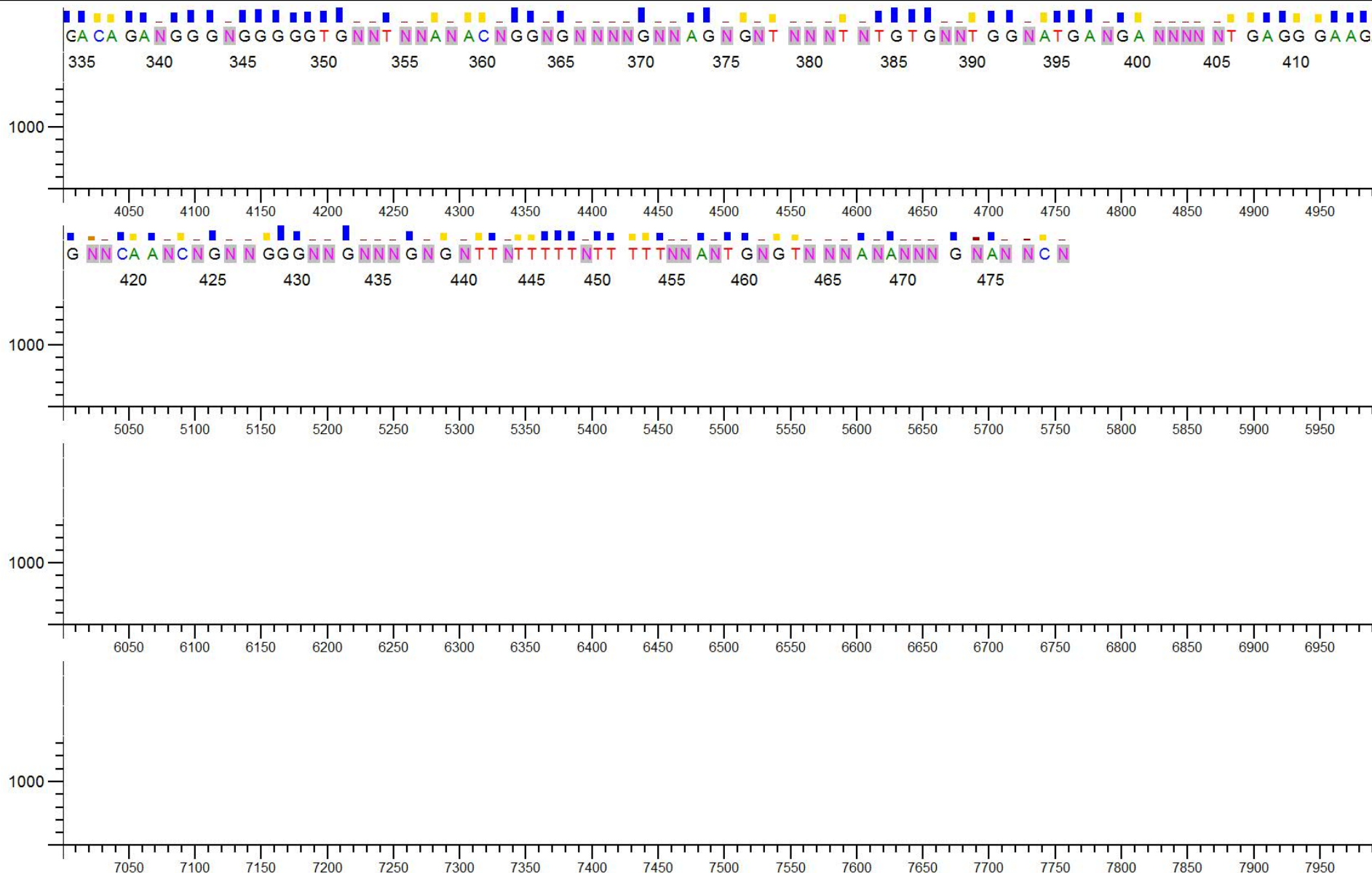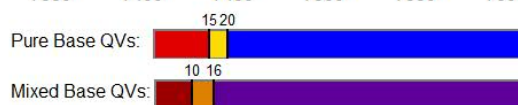

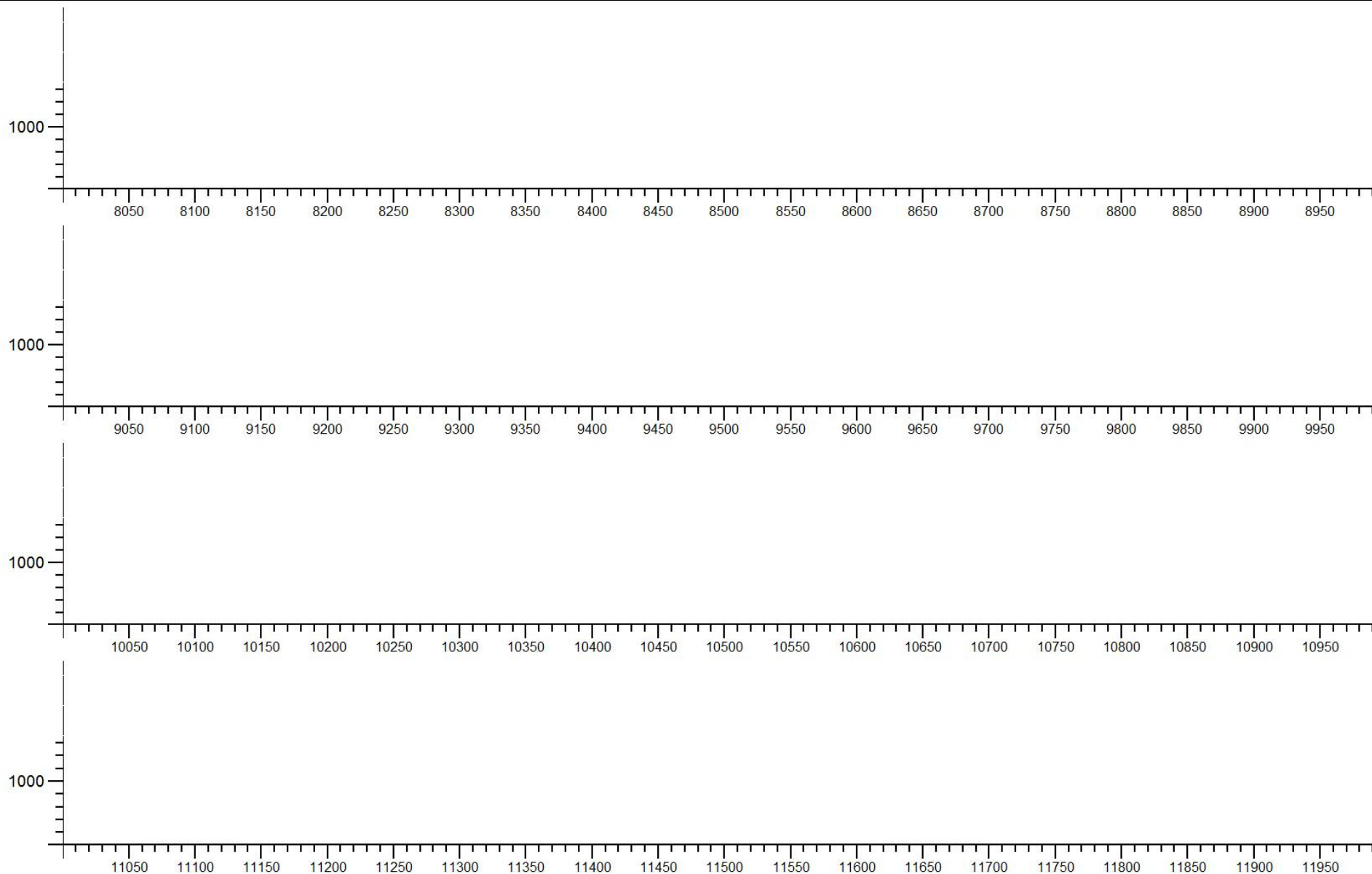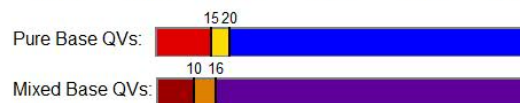

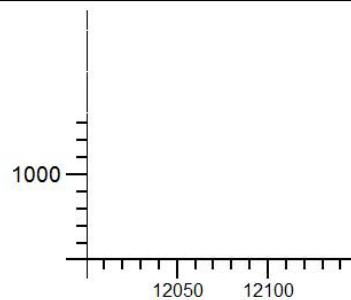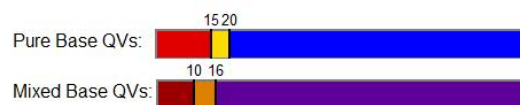

Supplement: Supplemental Information 1 — Chromatograms of: (1) recombined sequences of the H47 GI model from a number of mutants affected in recombination functions, and (2) recombined sequences of the pUYFRT model. [file peerj-05-3293-s001.zip › raw material/12-RecJ_out1_FA.pdf]

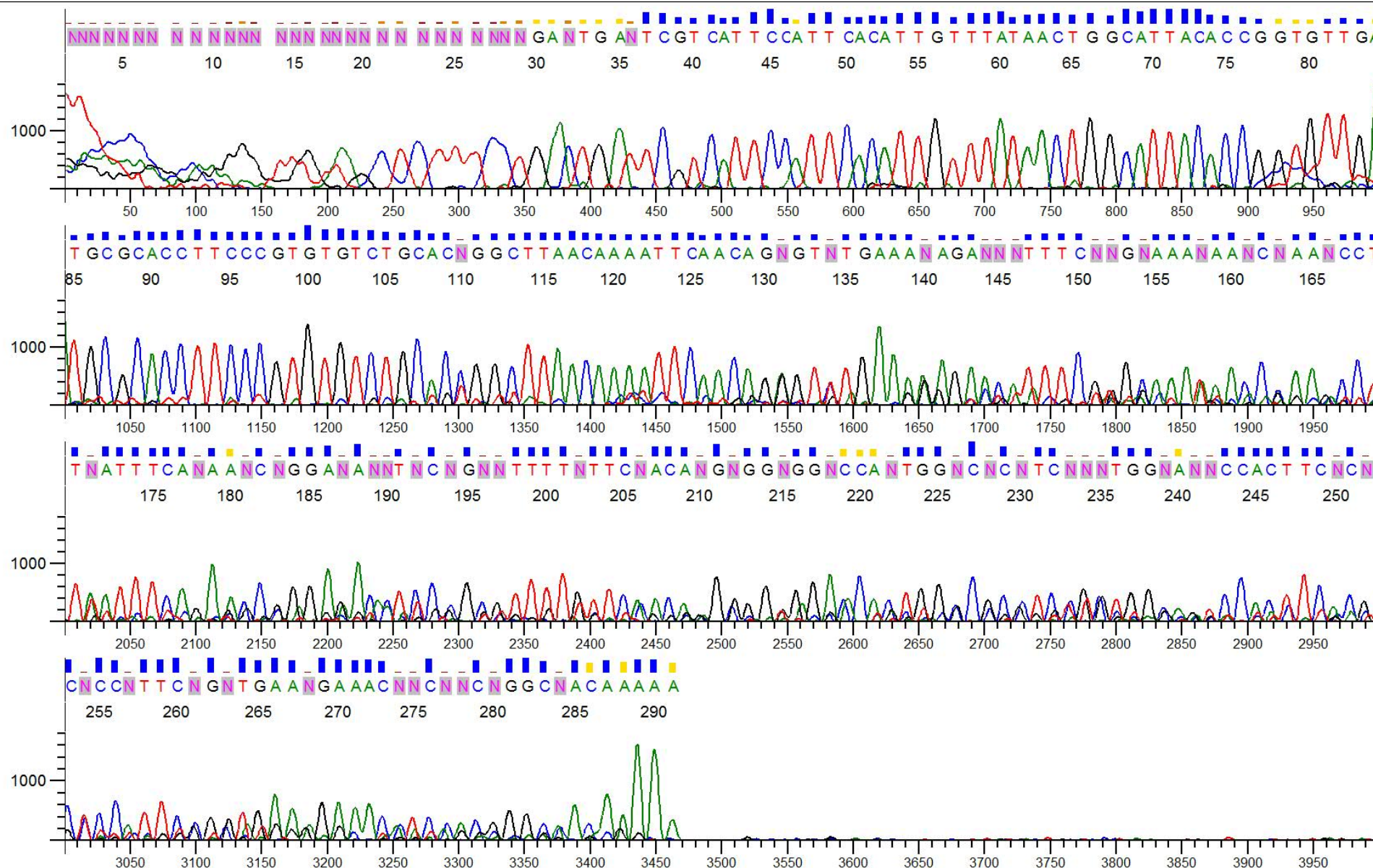

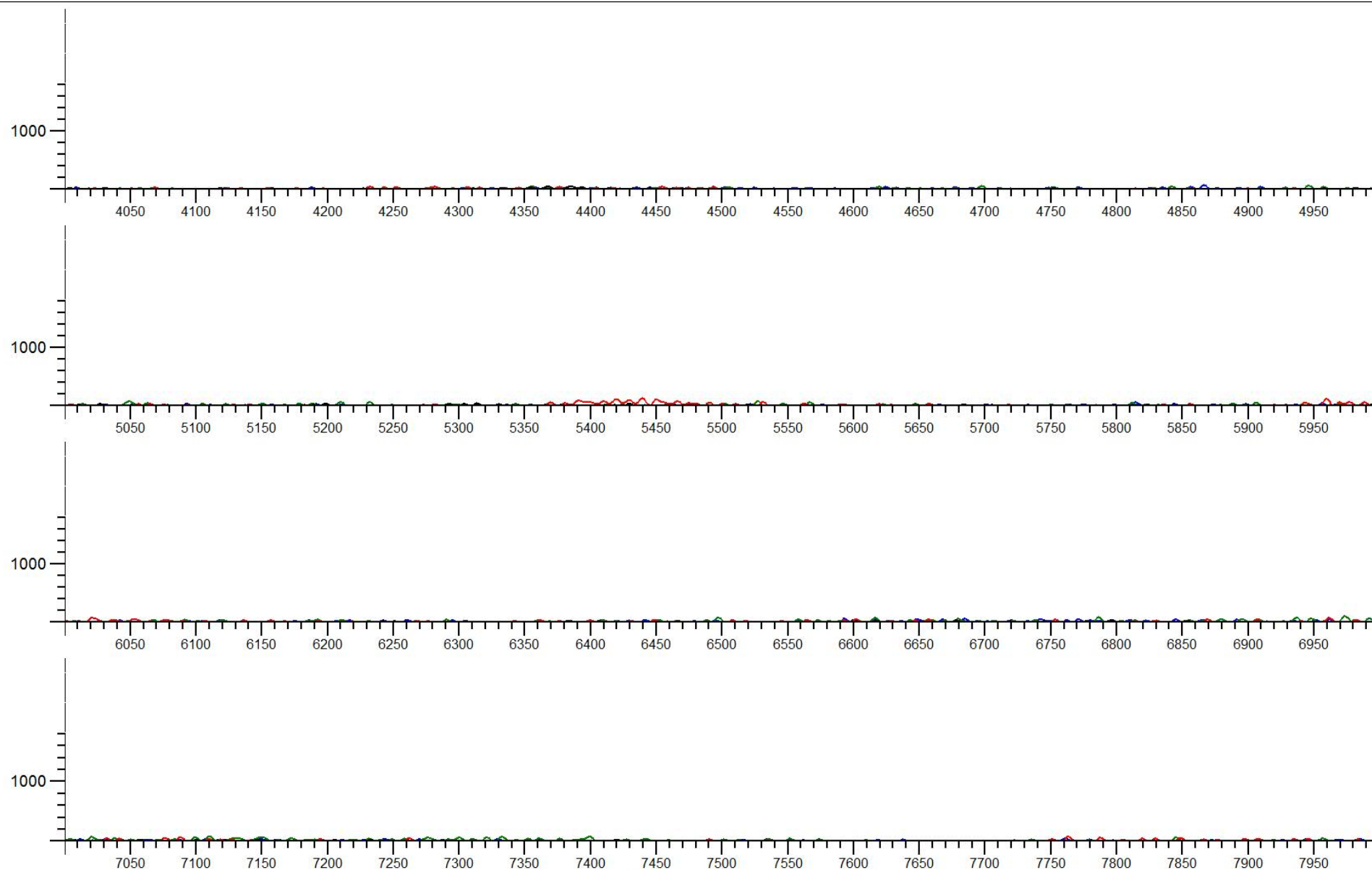

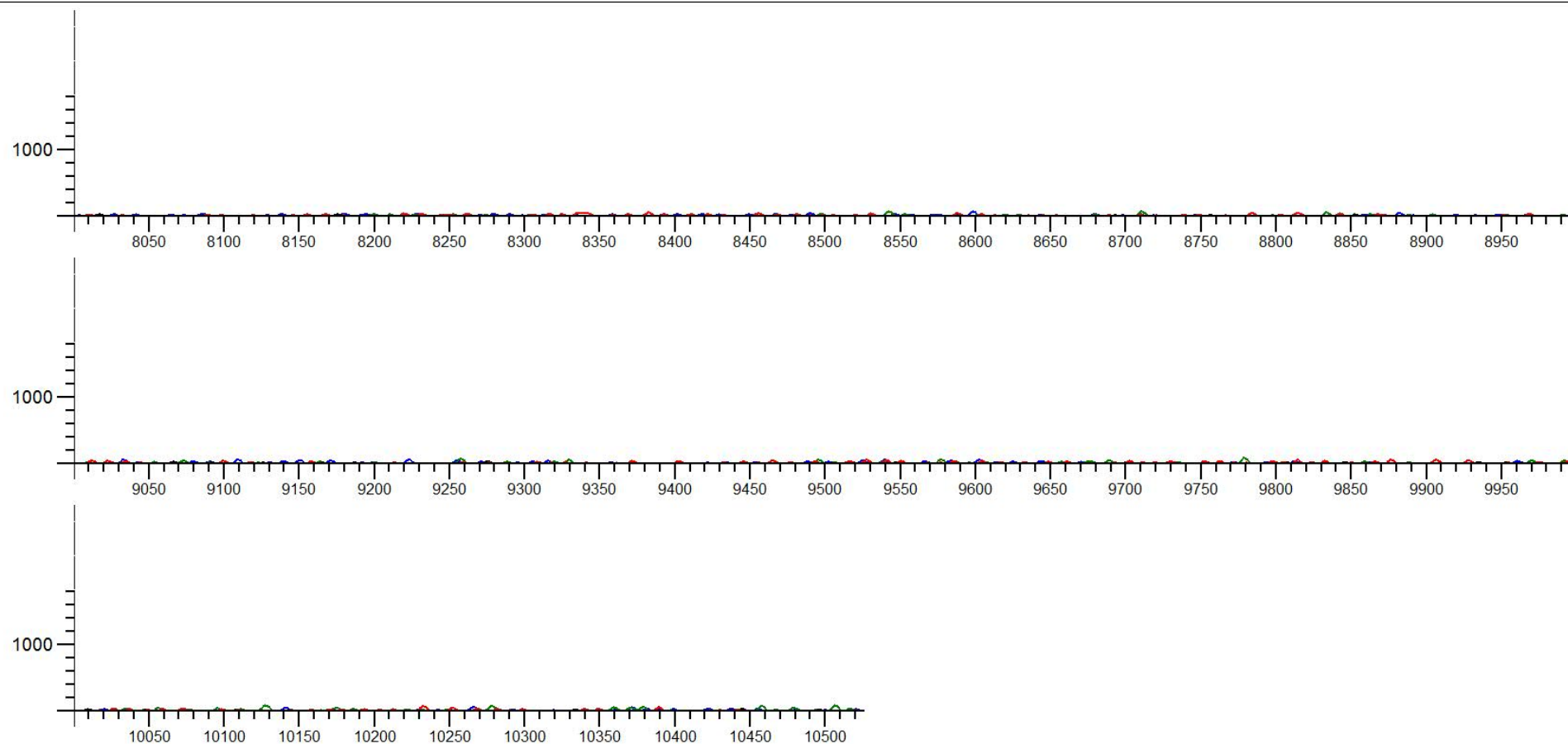

Supplement: Supplemental Information 1 — Chromatograms of: (1) recombined sequences of the H47 GI model from a number of mutants affected in recombination functions, and (2) recombined sequences of the pUYFRT model. [file peerj-05-3293-s001.zip › raw material/13-RecG_out1_FA.pdf]

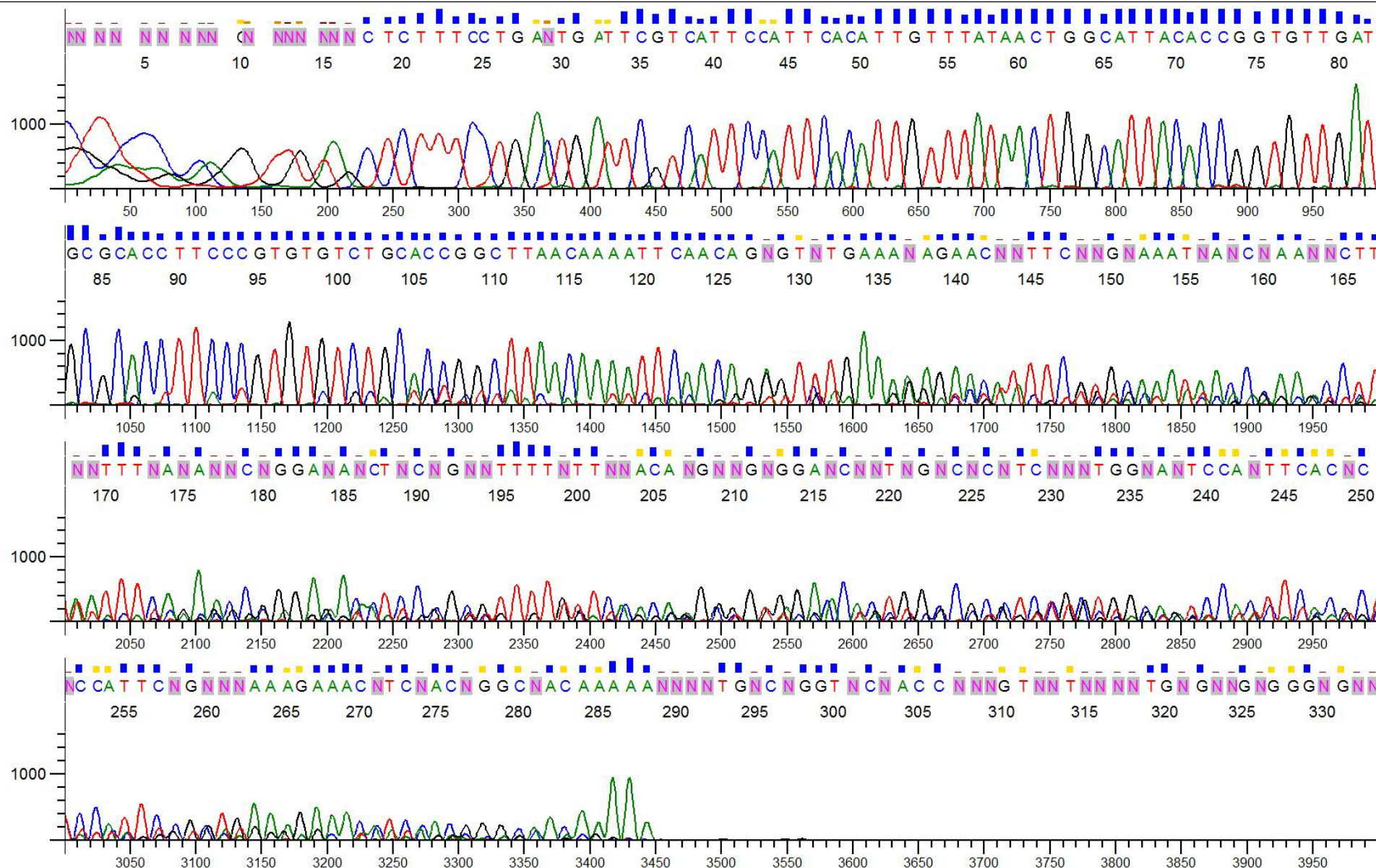

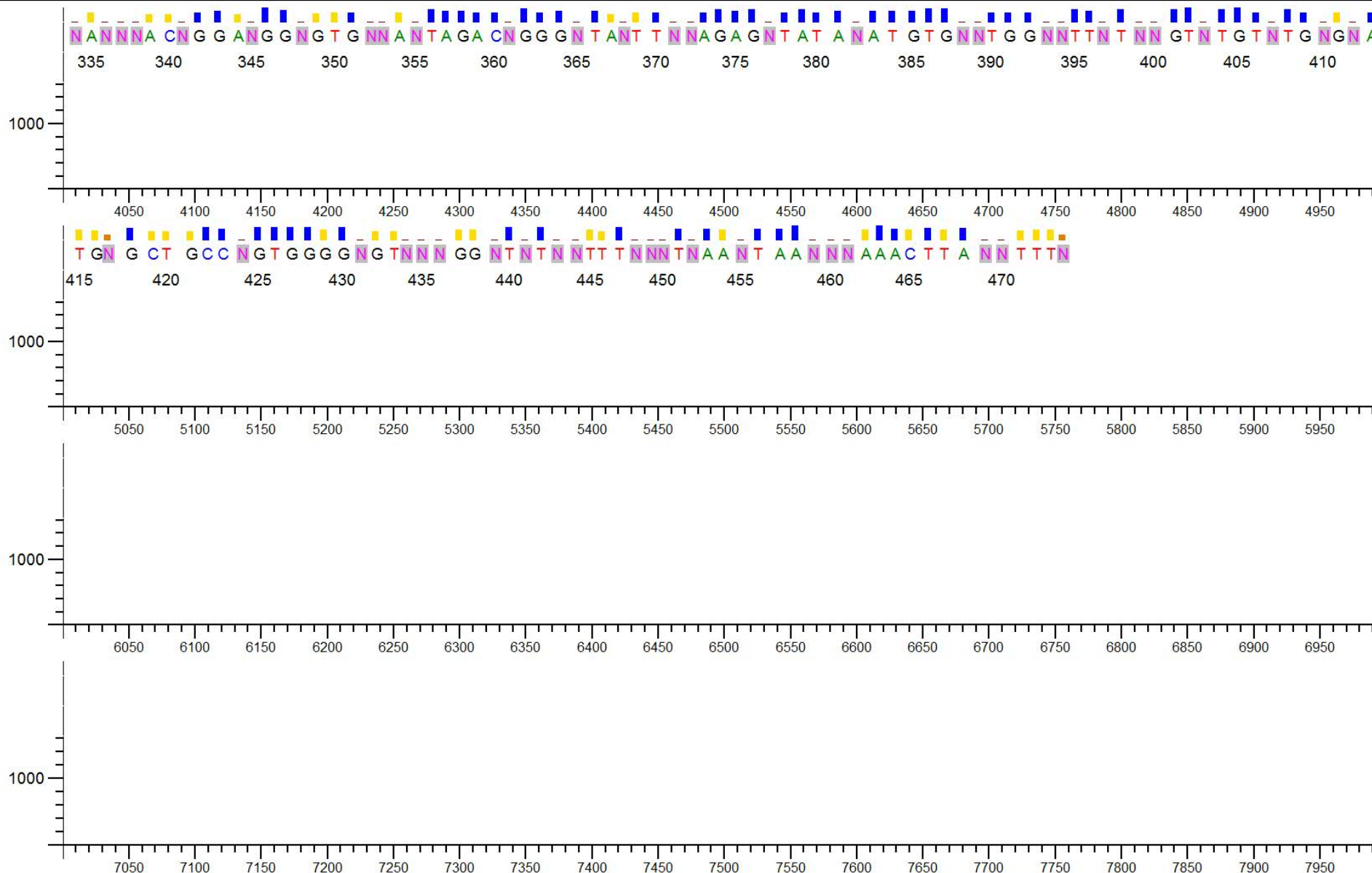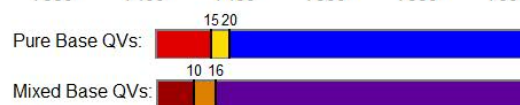

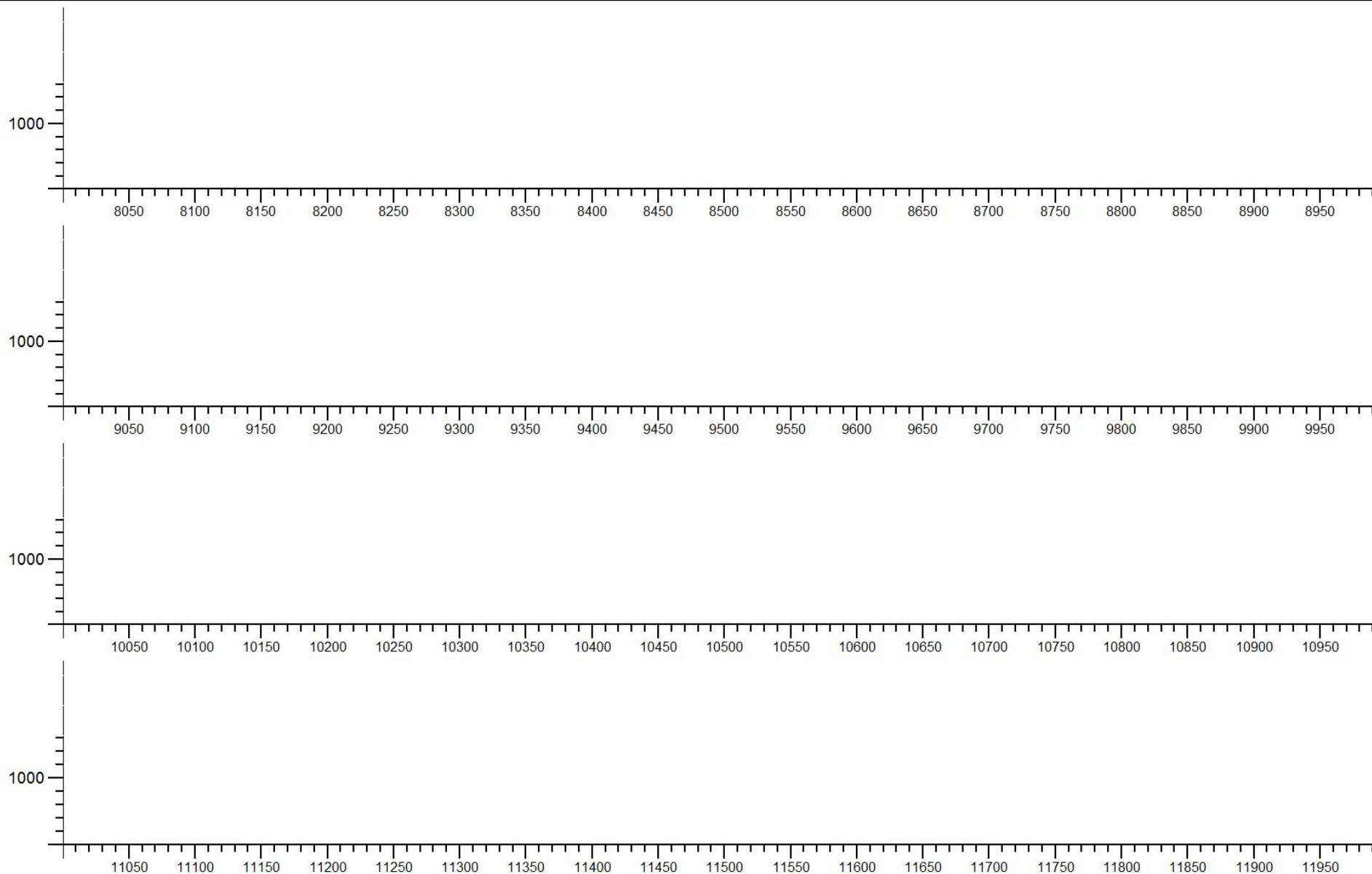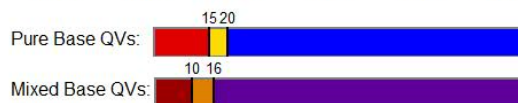

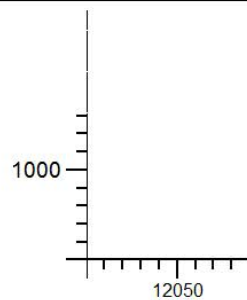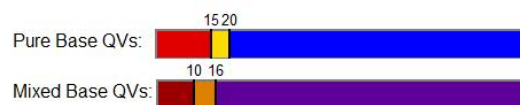

Supplement: Supplemental Information 1 — Chromatograms of: (1) recombined sequences of the H47 GI model from a number of mutants affected in recombination functions, and (2) recombined sequences of the pUYFRT model. [file peerj-05-3293-s001.zip › raw material/14-SbcD_out1_FA.pdf]

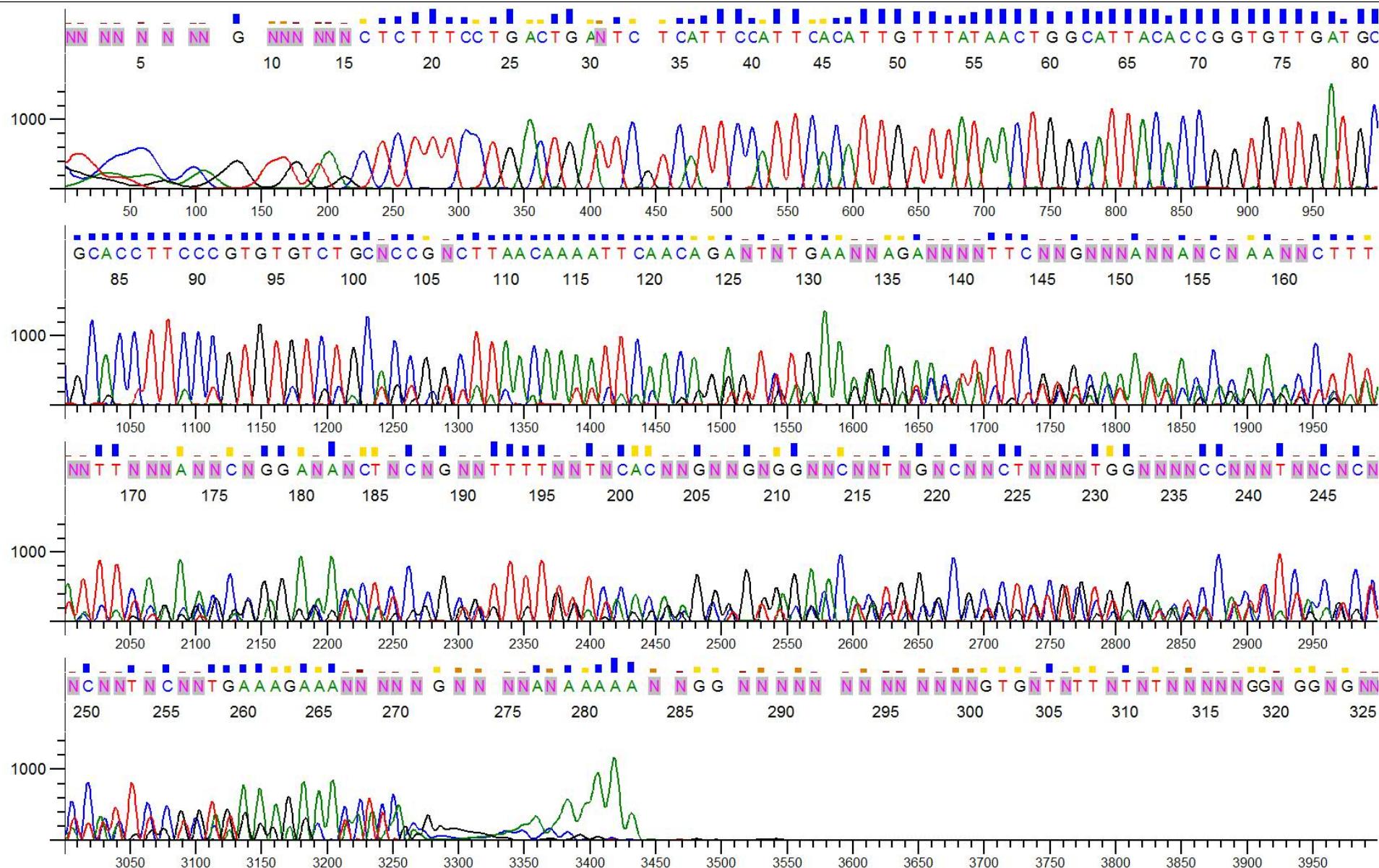

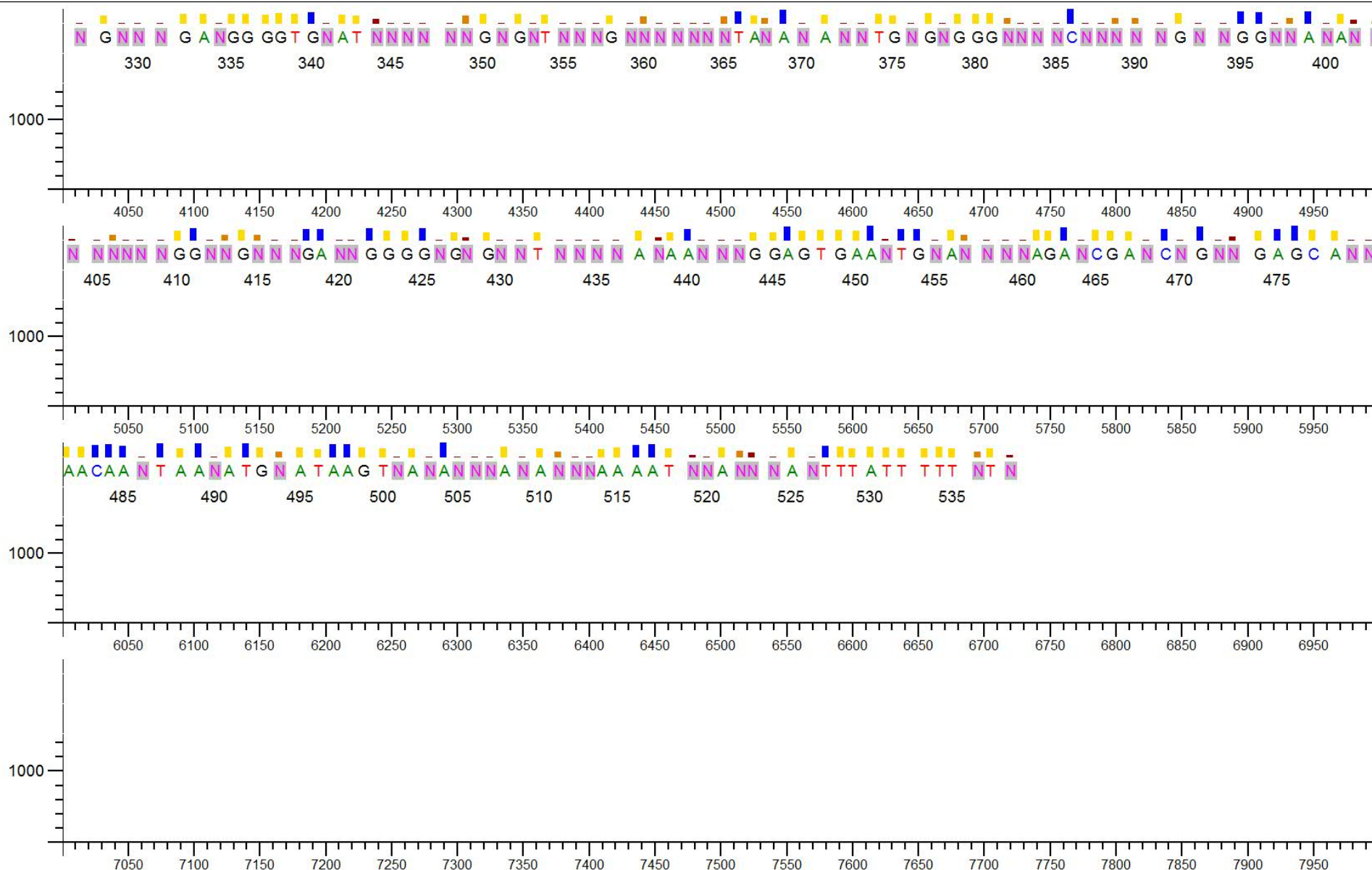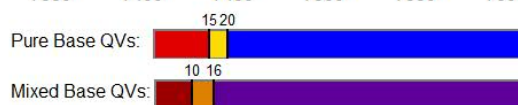

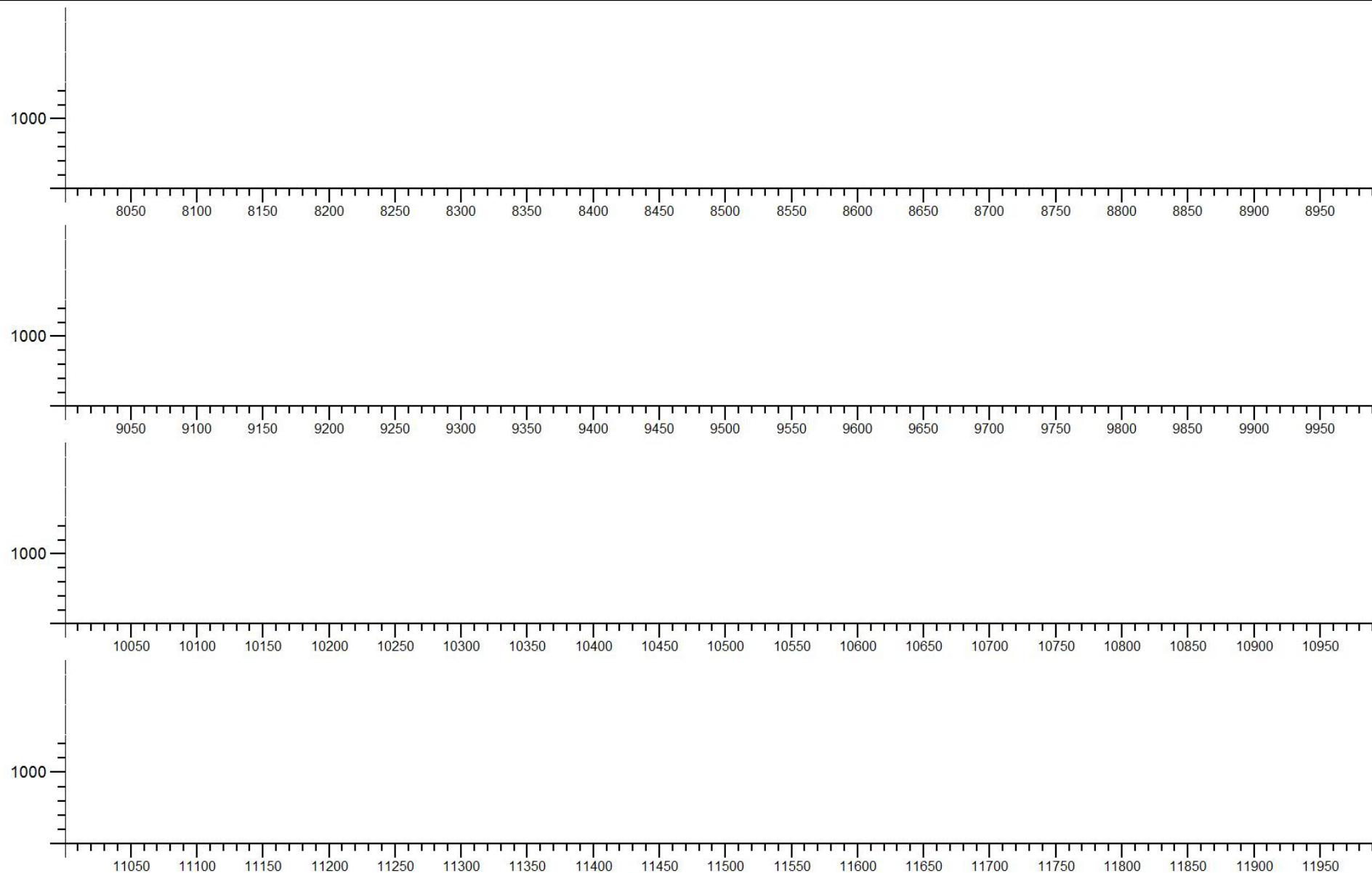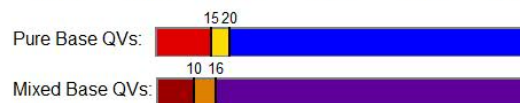

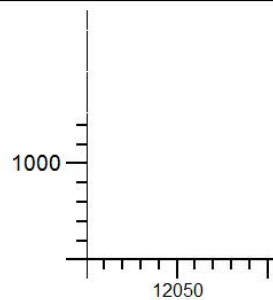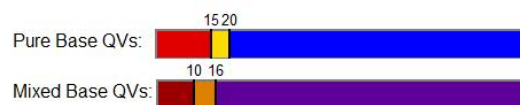

Supplement: Supplemental Information 1 — Chromatograms of: (1) recombined sequences of the H47 GI model from a number of mutants affected in recombination functions, and (2) recombined sequences of the pUYFRT model. [file peerj-05-3293-s001.zip › raw material/1-recR_out1_FA.pdf]

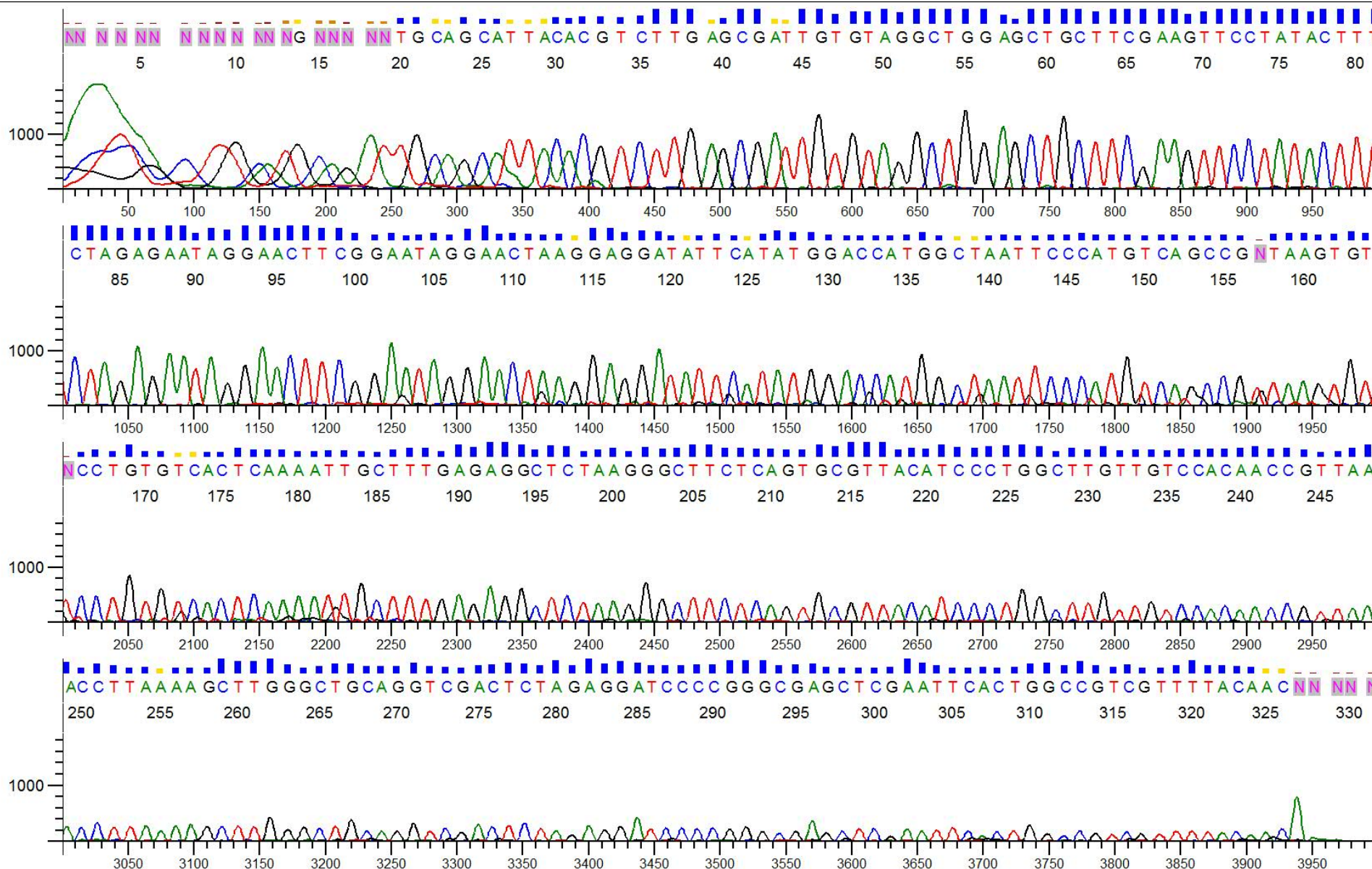

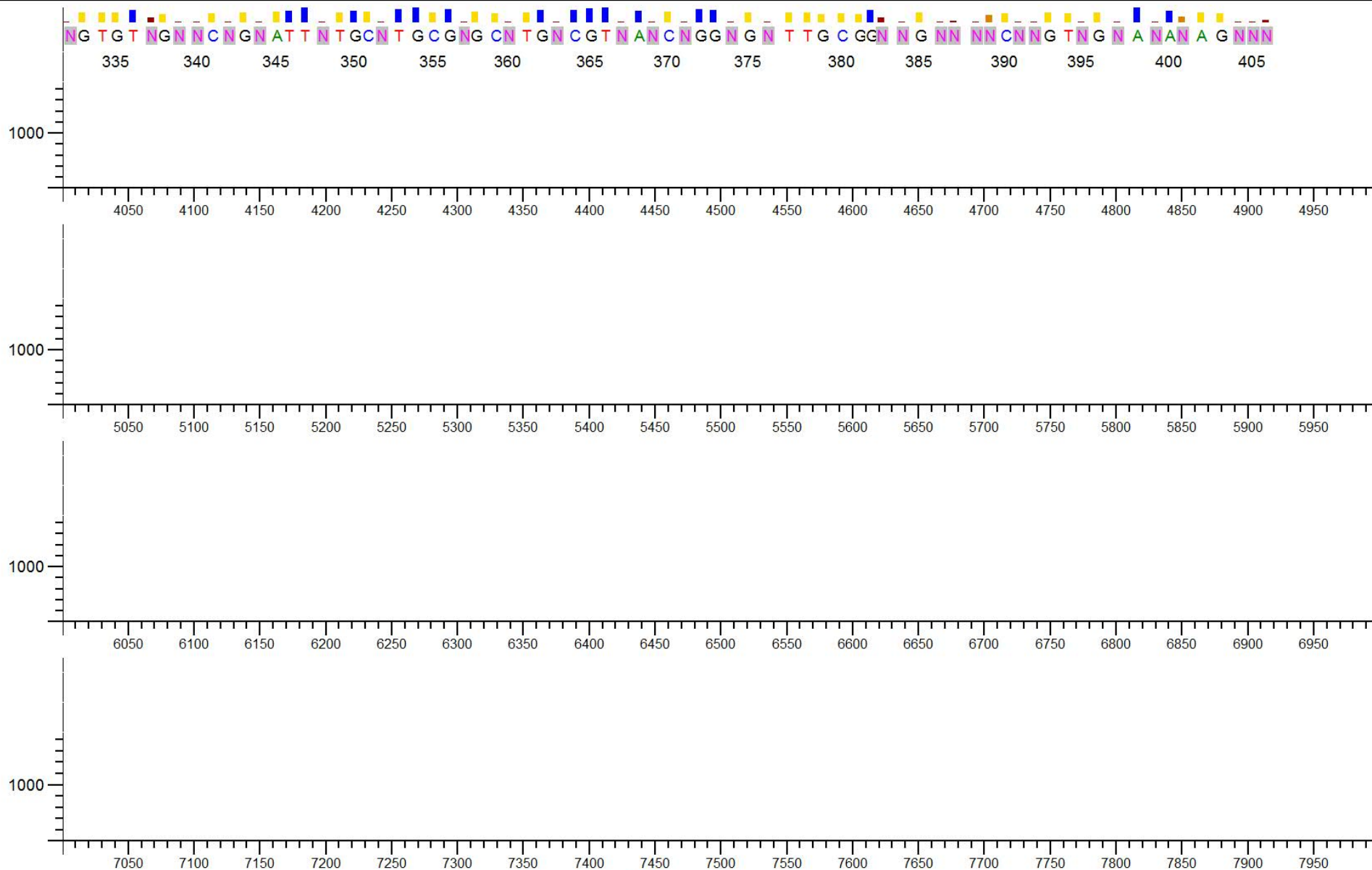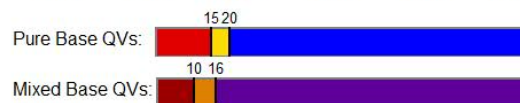

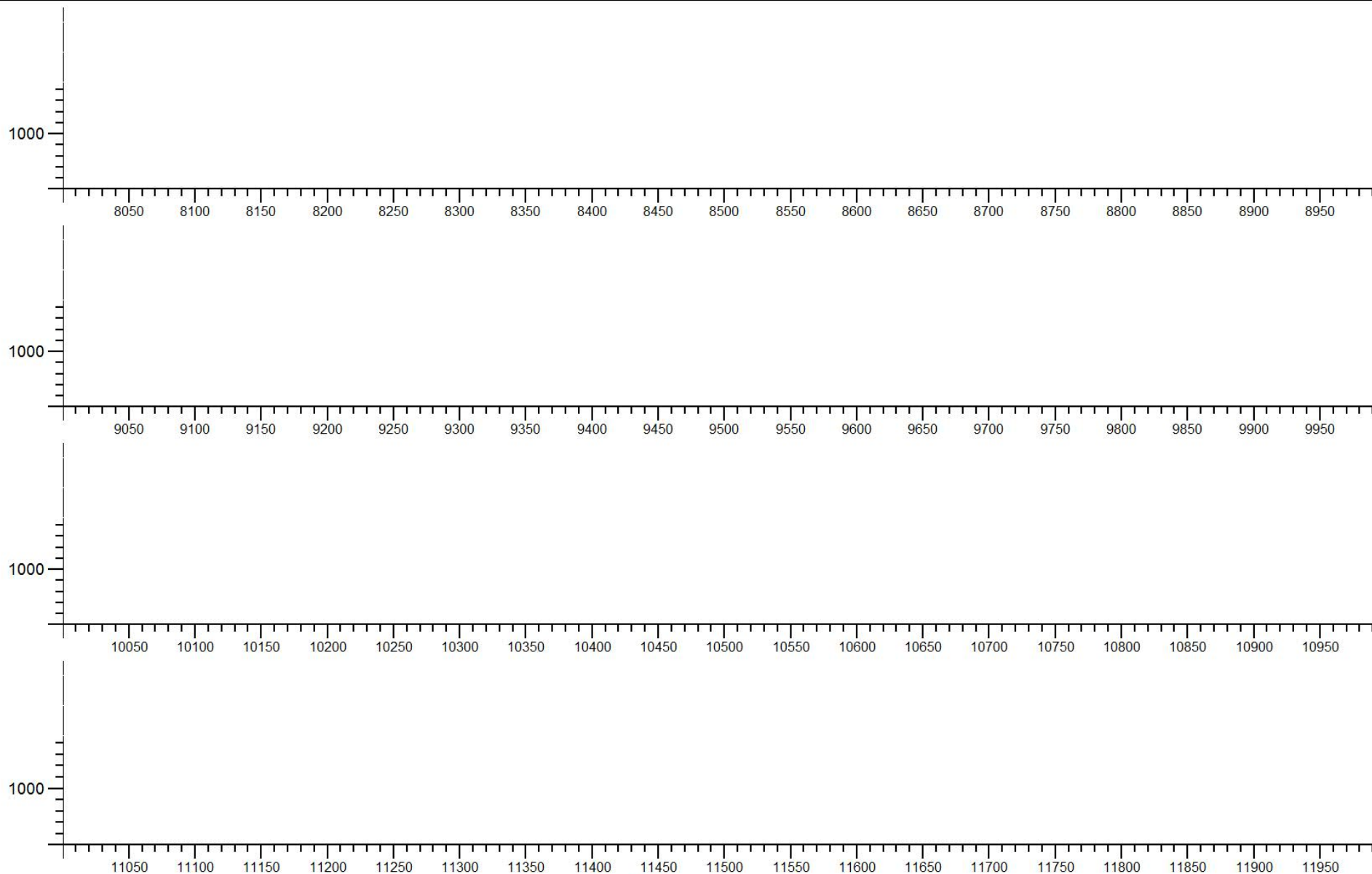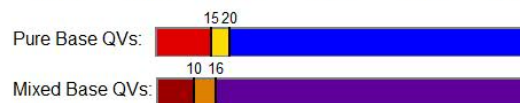

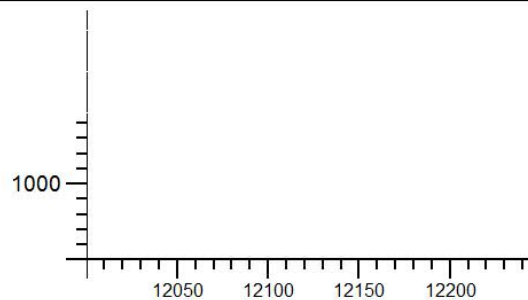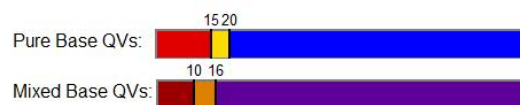

Supplement: Supplemental Information 1 — Chromatograms of: (1) recombined sequences of the H47 GI model from a number of mutants affected in recombination functions, and (2) recombined sequences of the pUYFRT model. [file peerj-05-3293-s001.zip › raw material/22-pUYFRT_recTA_outR_FA.pdf]

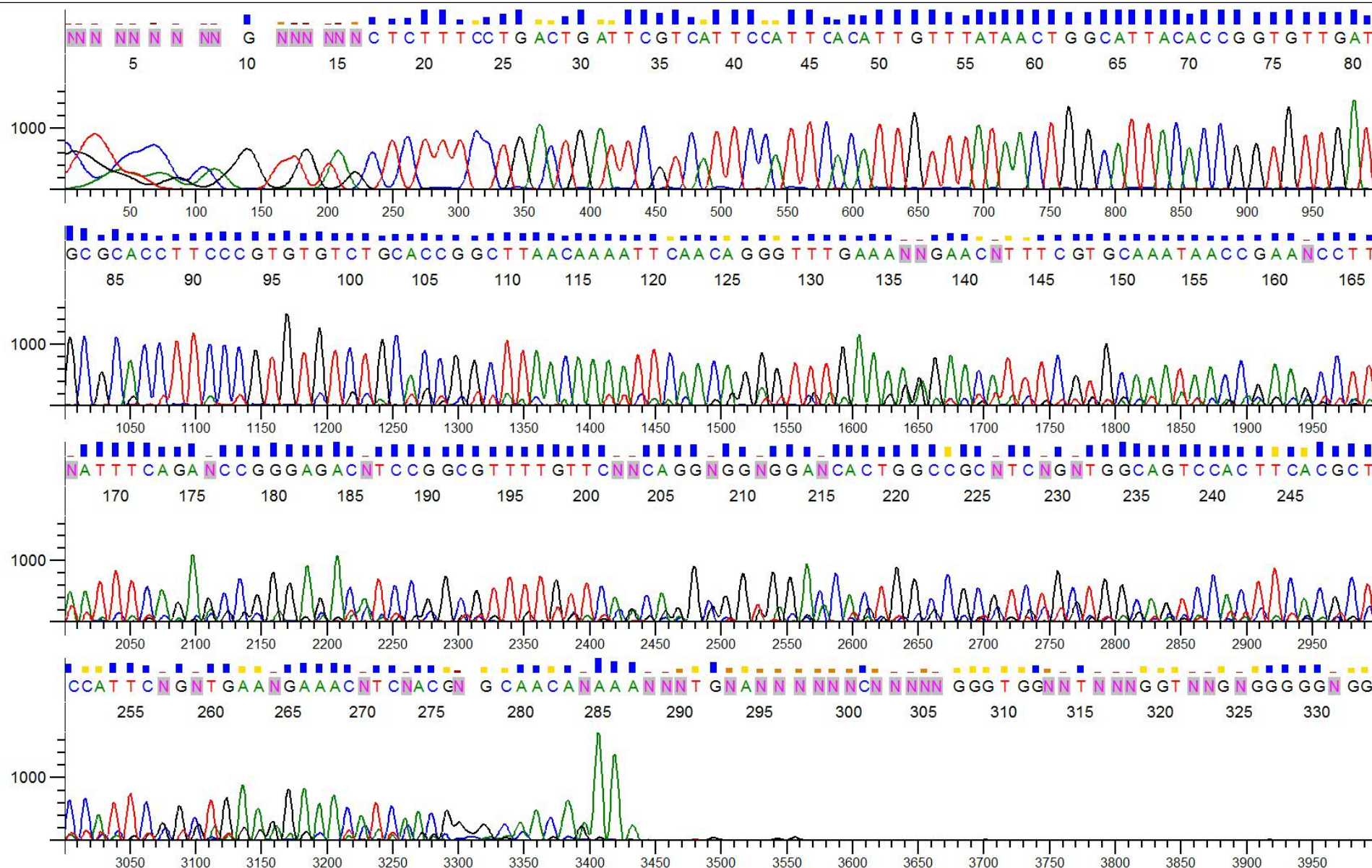

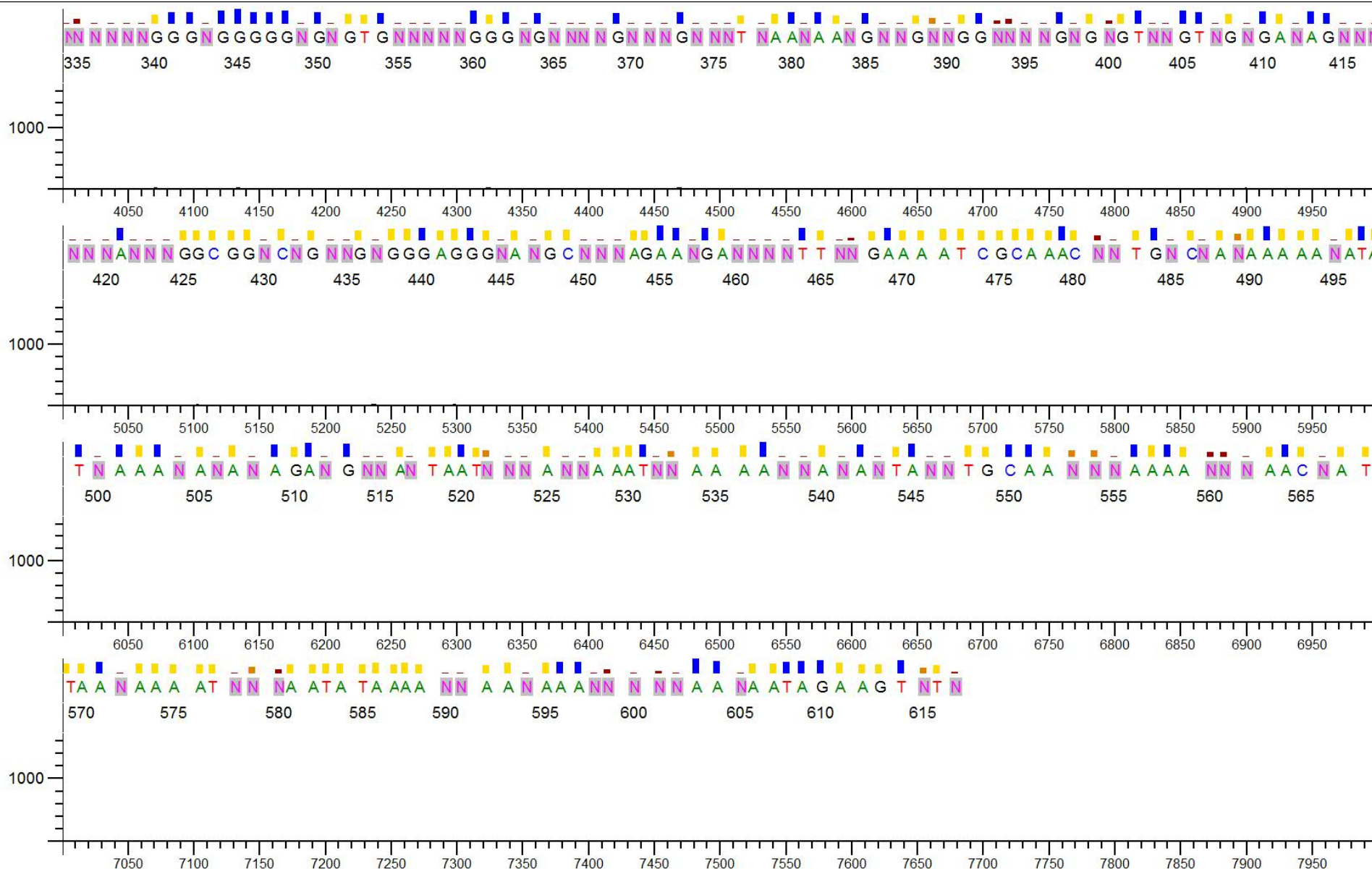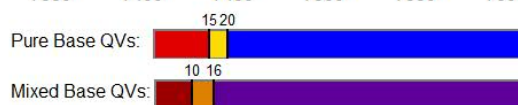

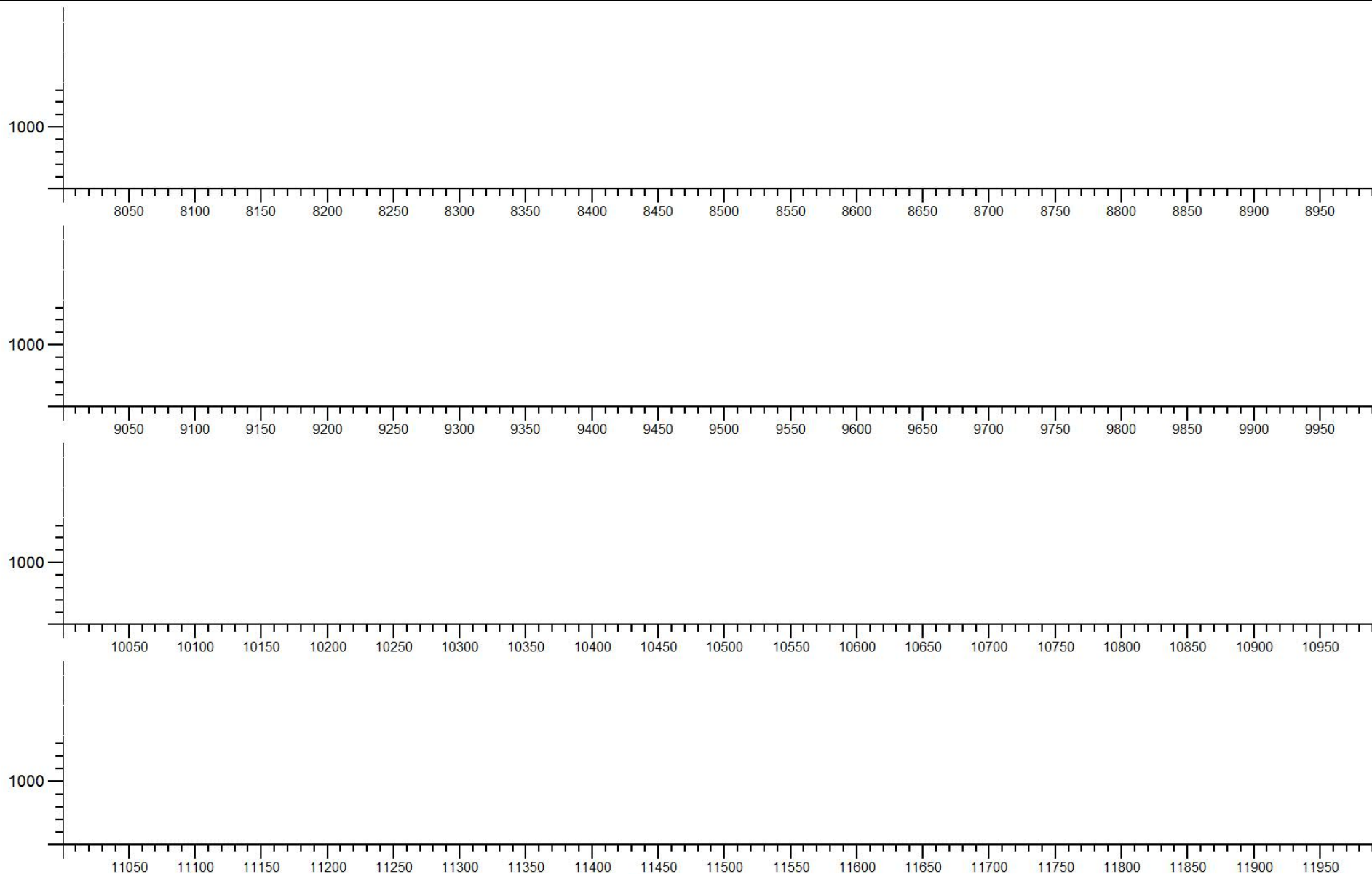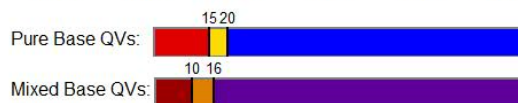

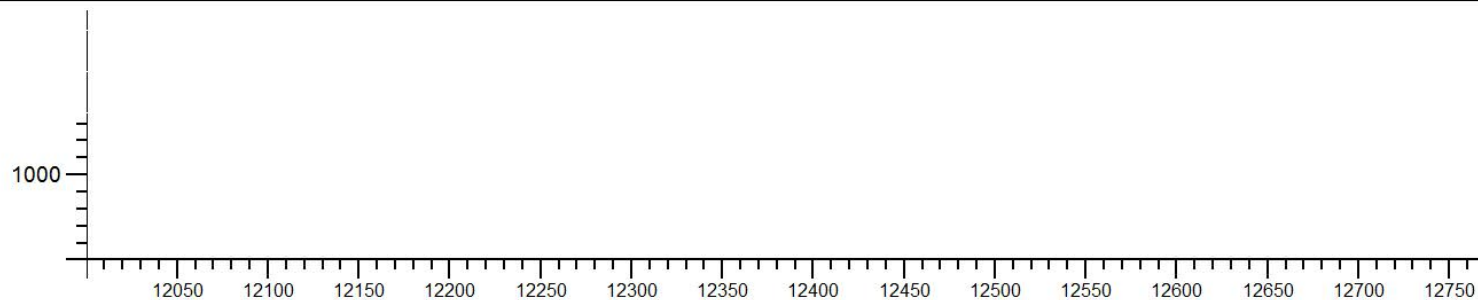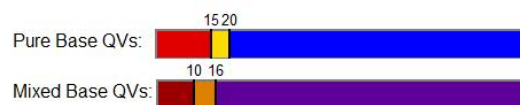

Supplement: Supplemental Information 1 — Chromatograms of: (1) recombined sequences of the H47 GI model from a number of mutants affected in recombination functions, and (2) recombined sequences of the pUYFRT model. [file peerj-05-3293-s001.zip › raw material/26-intDR_out1_FA.pdf]

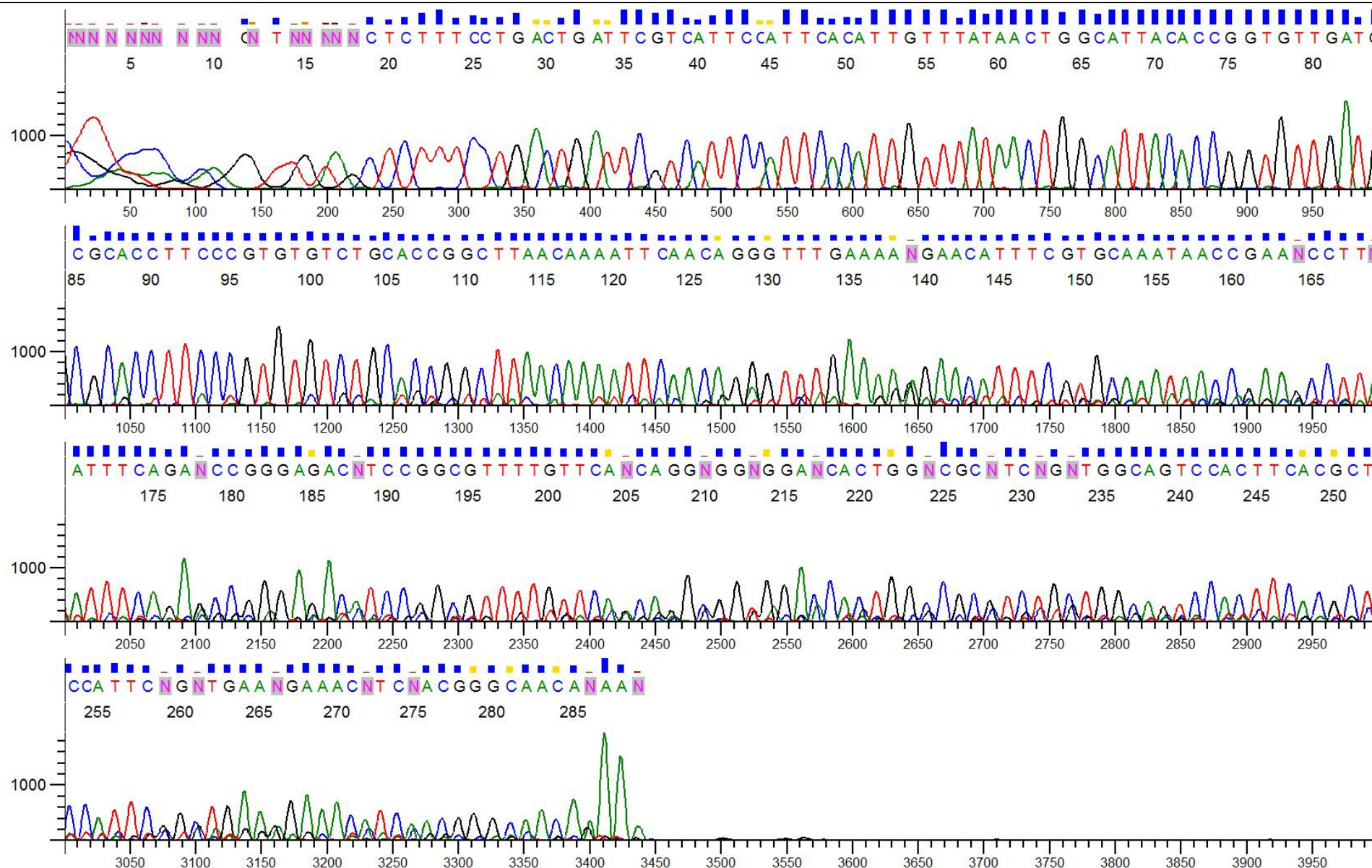

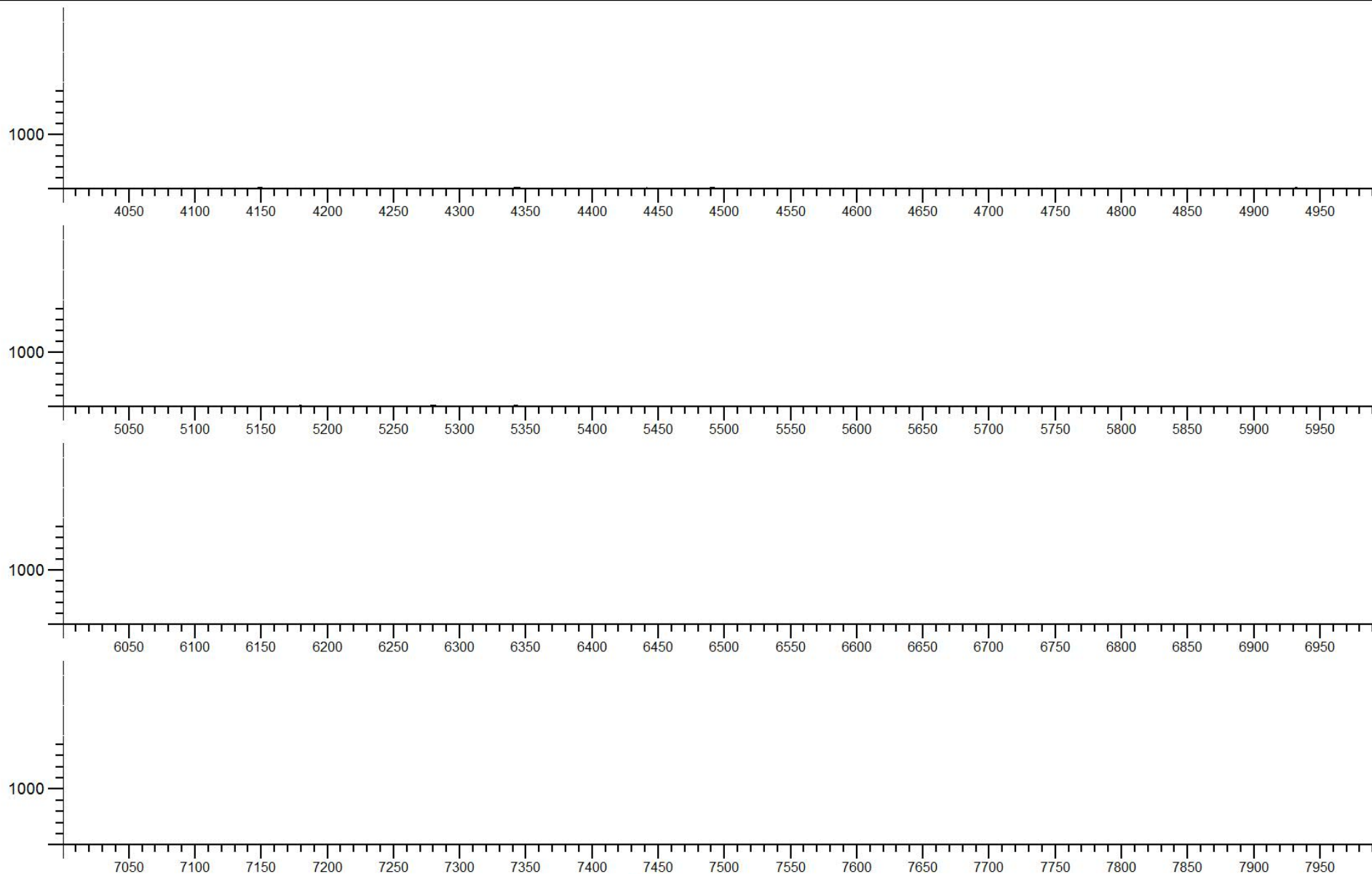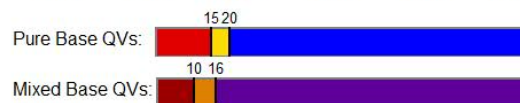

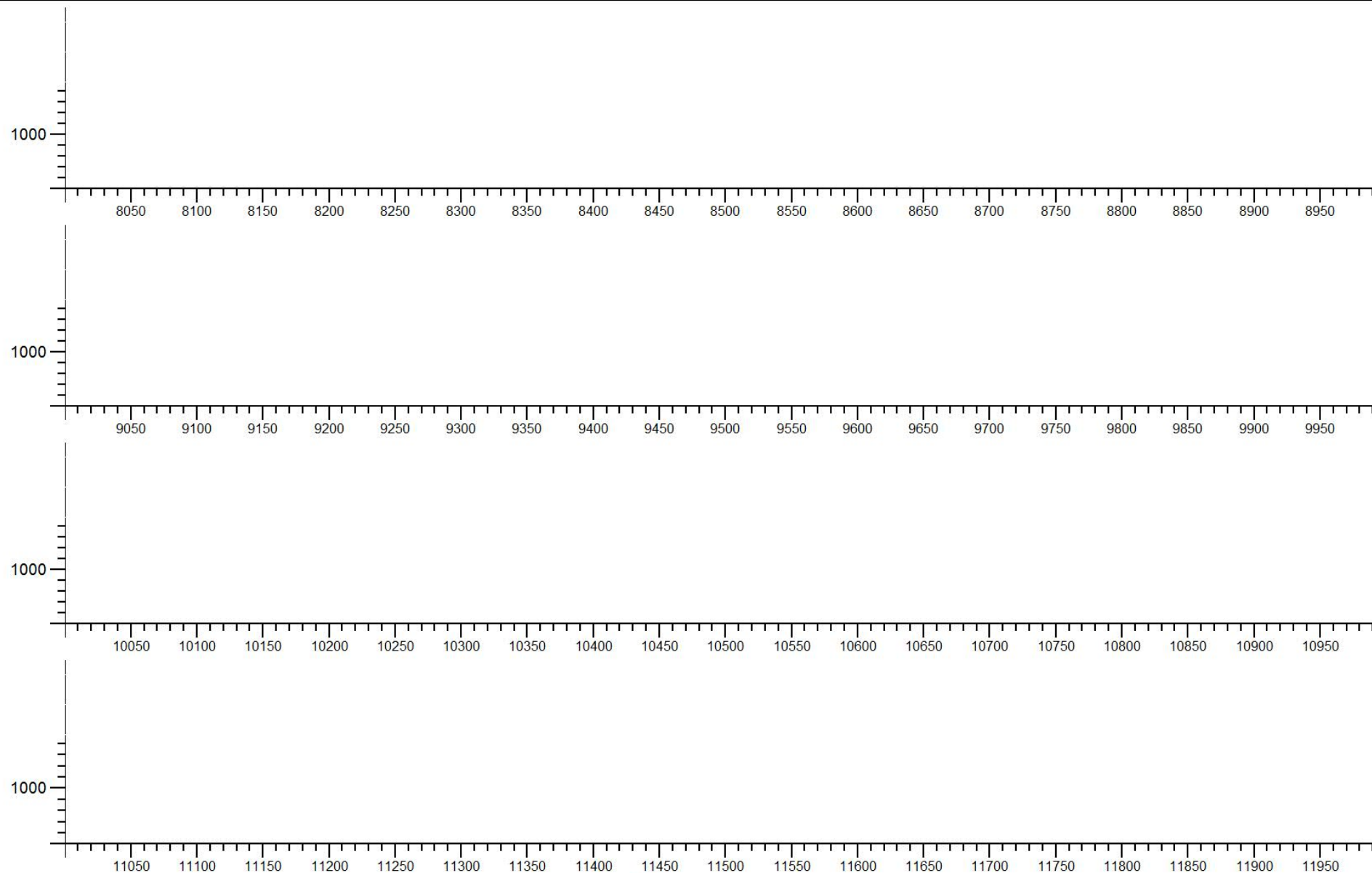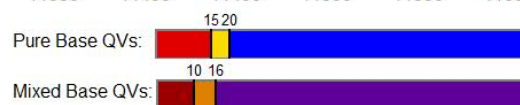

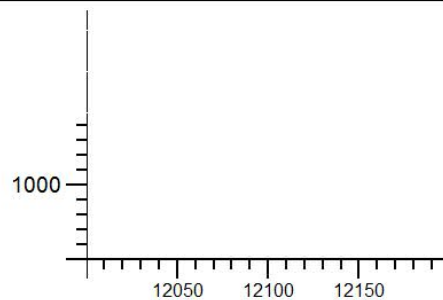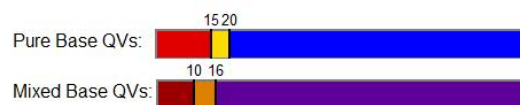

Supplement: Supplemental Information 1 — Chromatograms of: (1) recombined sequences of the H47 GI model from a number of mutants affected in recombination functions, and (2) recombined sequences of the pUYFRT model. [file peerj-05-3293-s001.zip › raw material/27-intDQ_out1_FA.pdf]

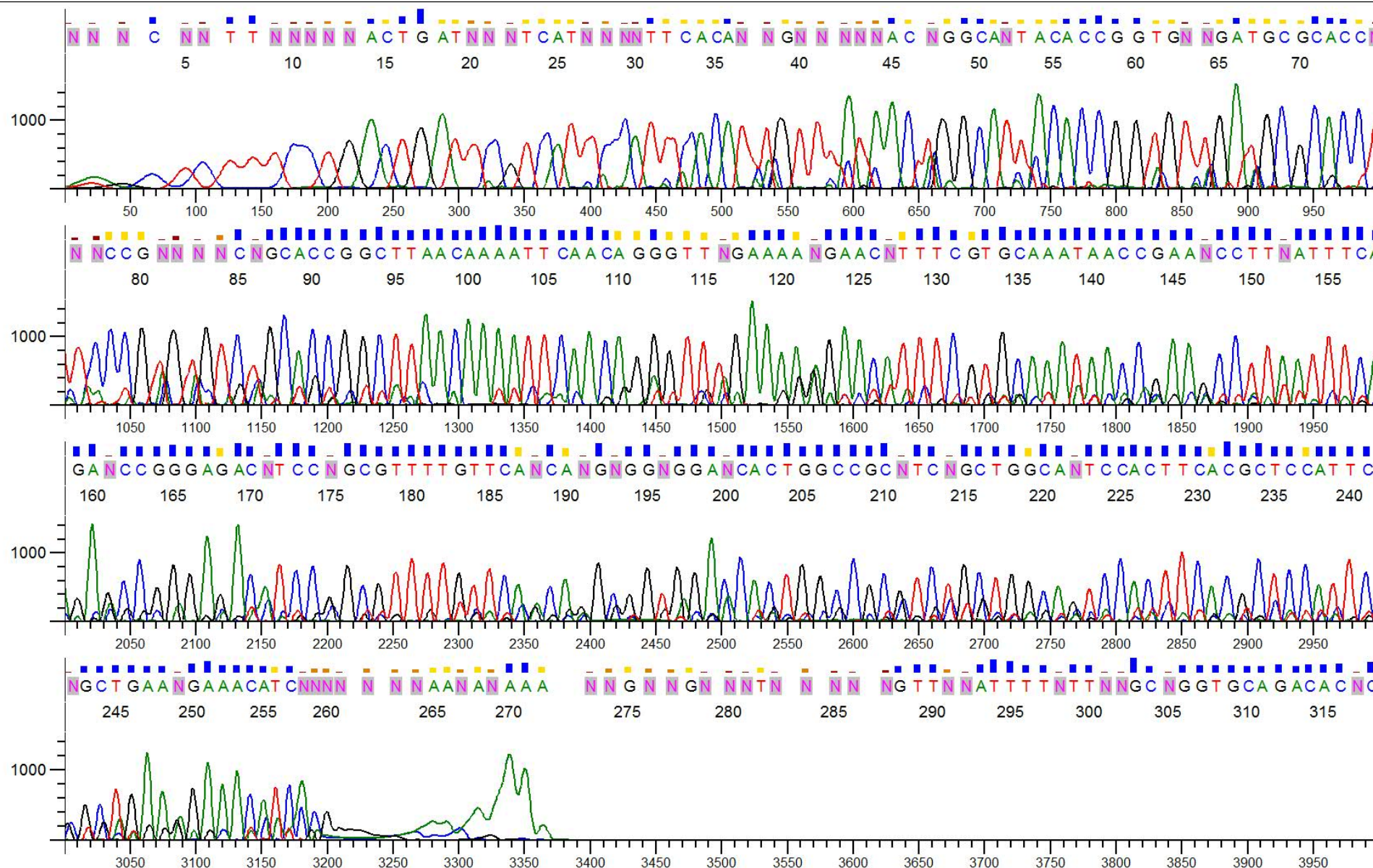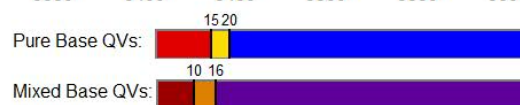

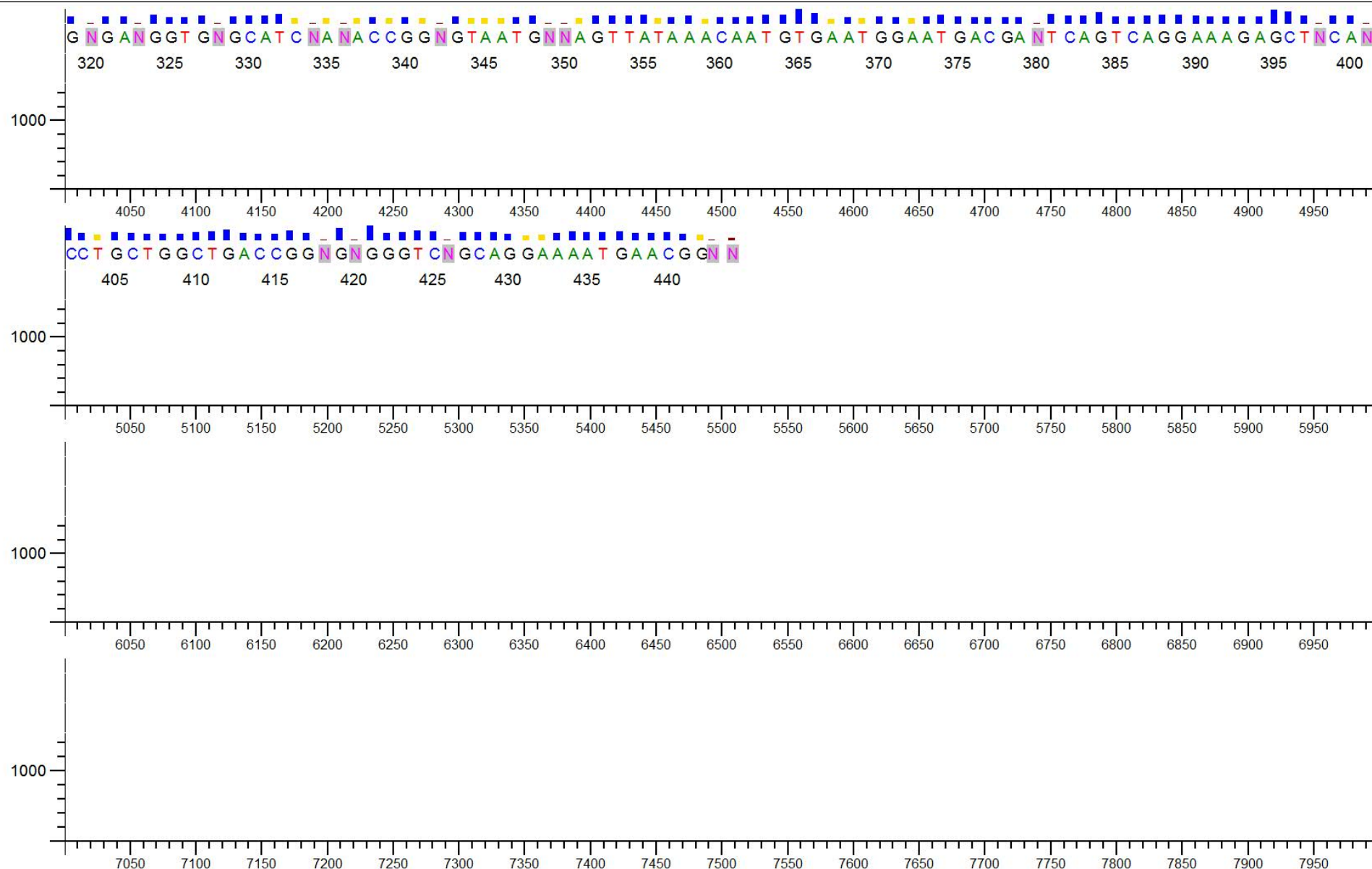

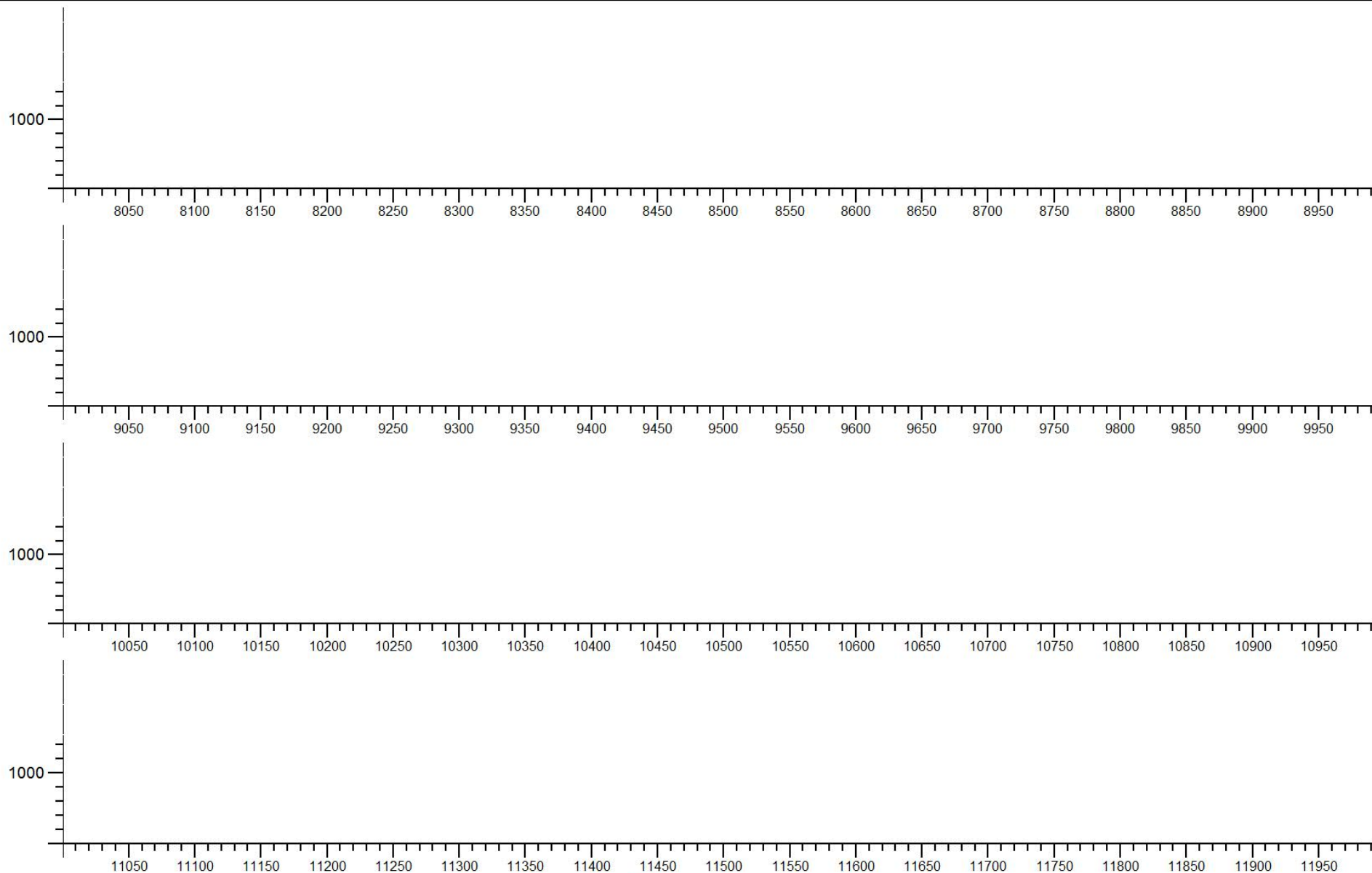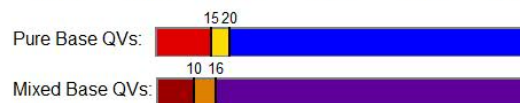

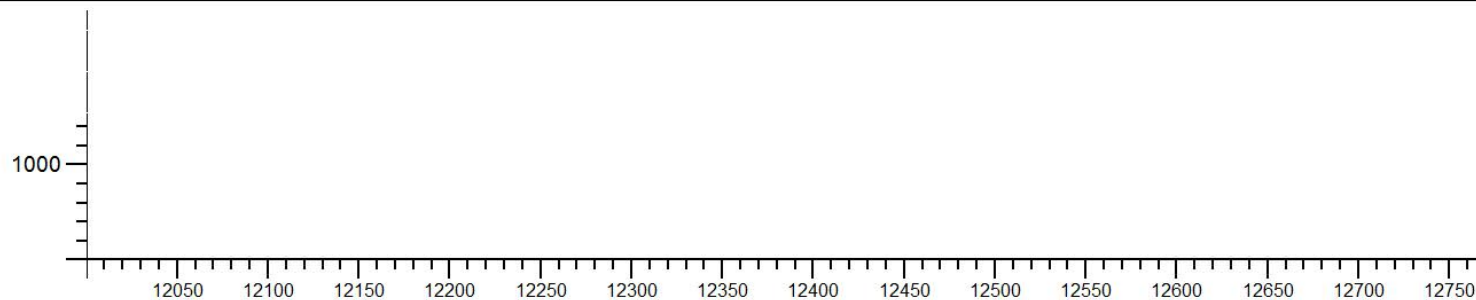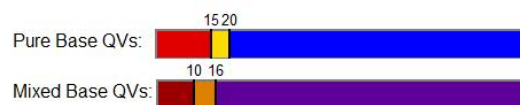

Supplement: Supplemental Information 1 — Chromatograms of: (1) recombined sequences of the H47 GI model from a number of mutants affected in recombination functions, and (2) recombined sequences of the pUYFRT model. [file peerj-05-3293-s001.zip › raw material/28-intDA_out1_FA.pdf]

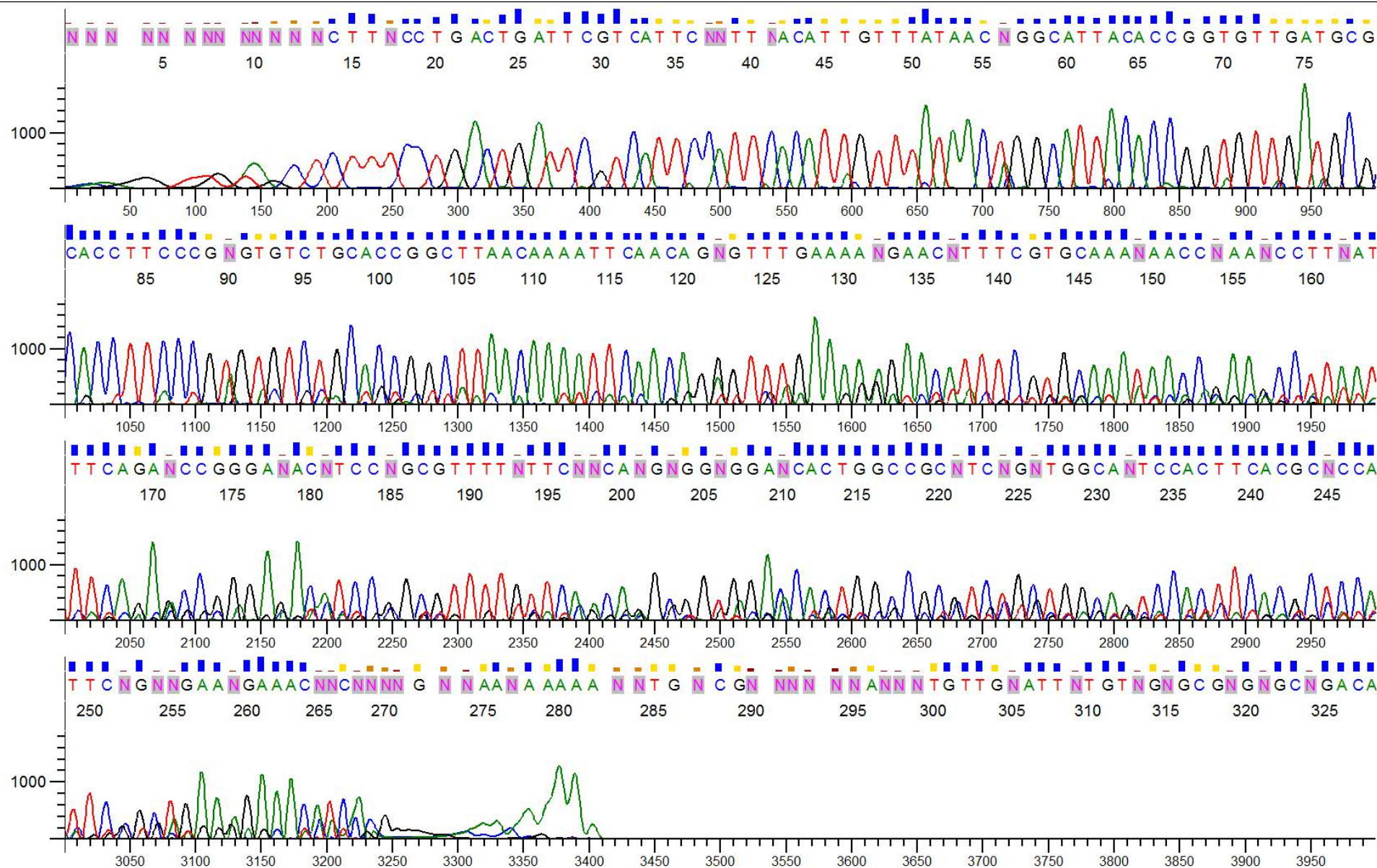

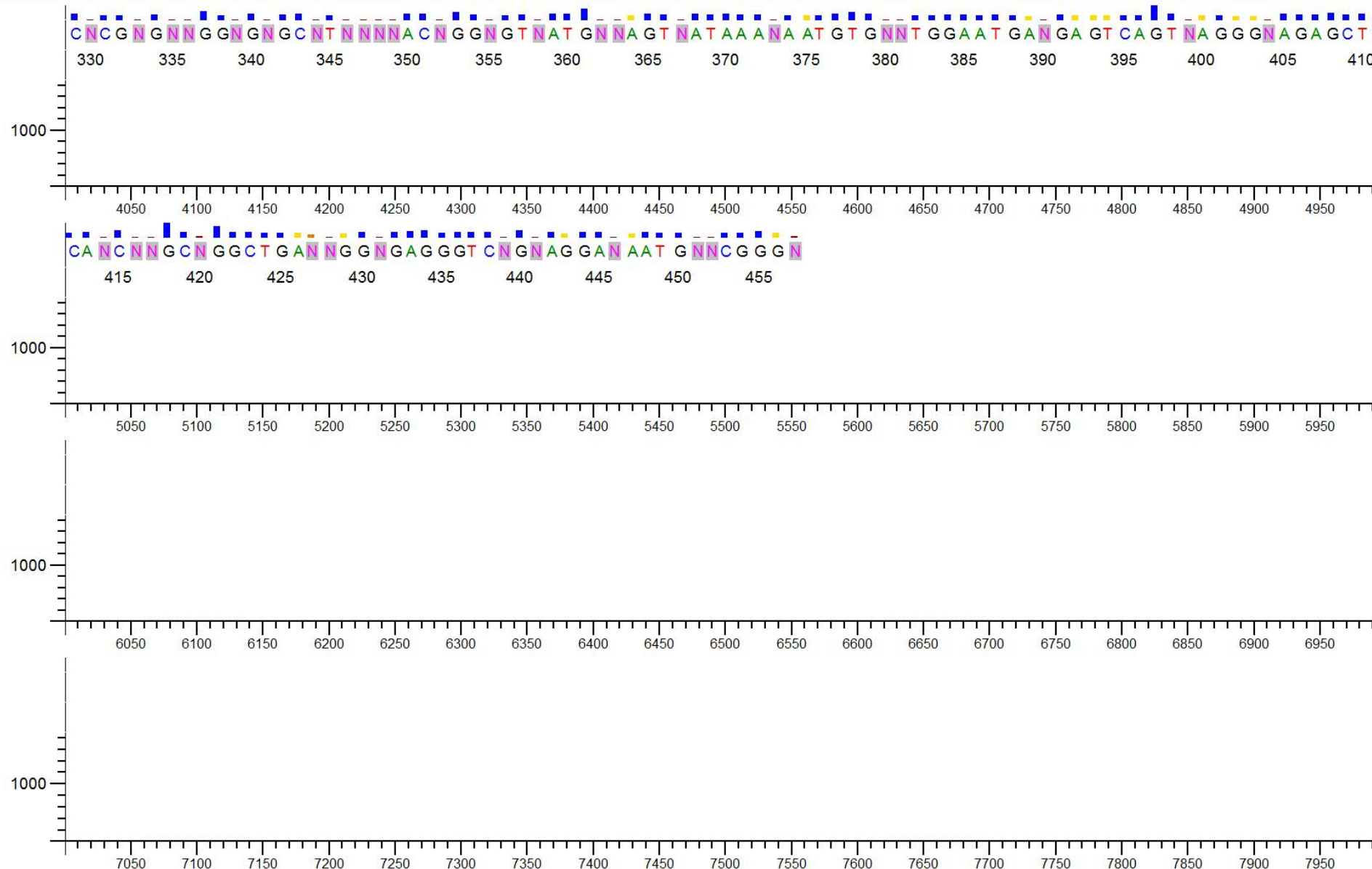

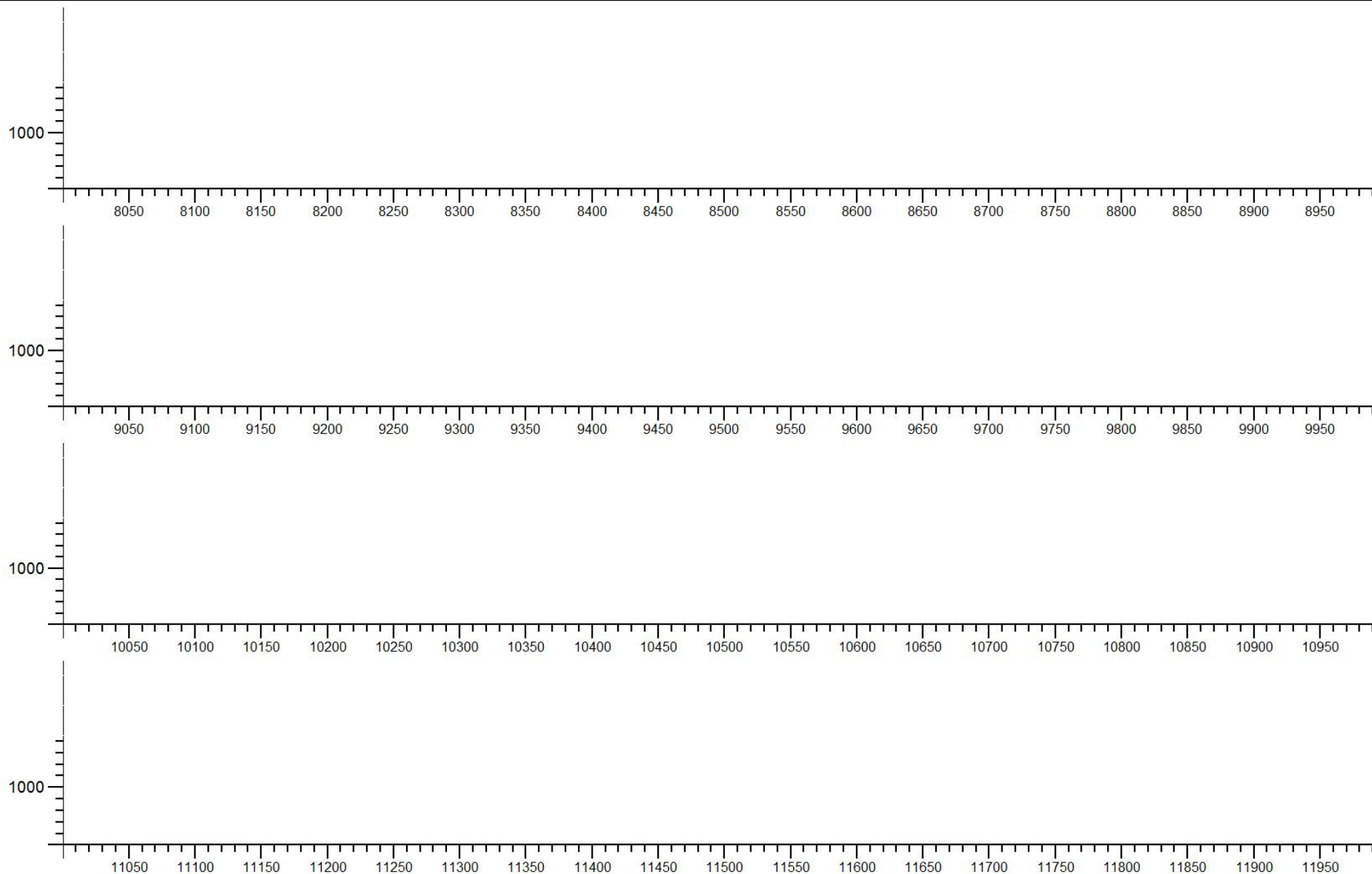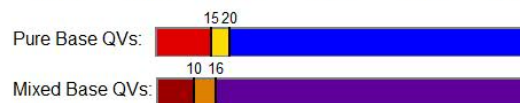

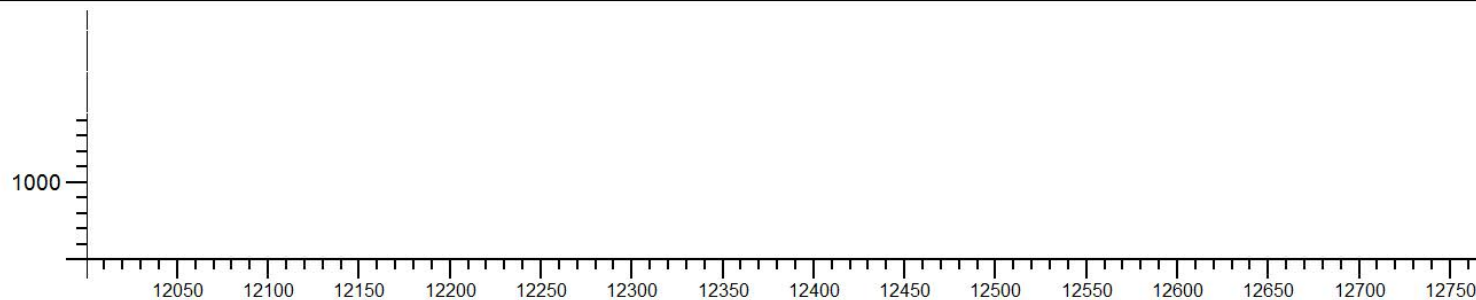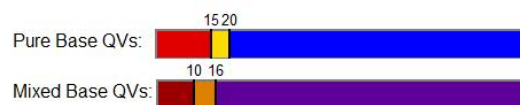

Supplement: Supplemental Information 1 — Chromatograms of: (1) recombined sequences of the H47 GI model from a number of mutants affected in recombination functions, and (2) recombined sequences of the pUYFRT model. [file peerj-05-3293-s001.zip › raw material/29-intAQ_out1_FA.pdf]

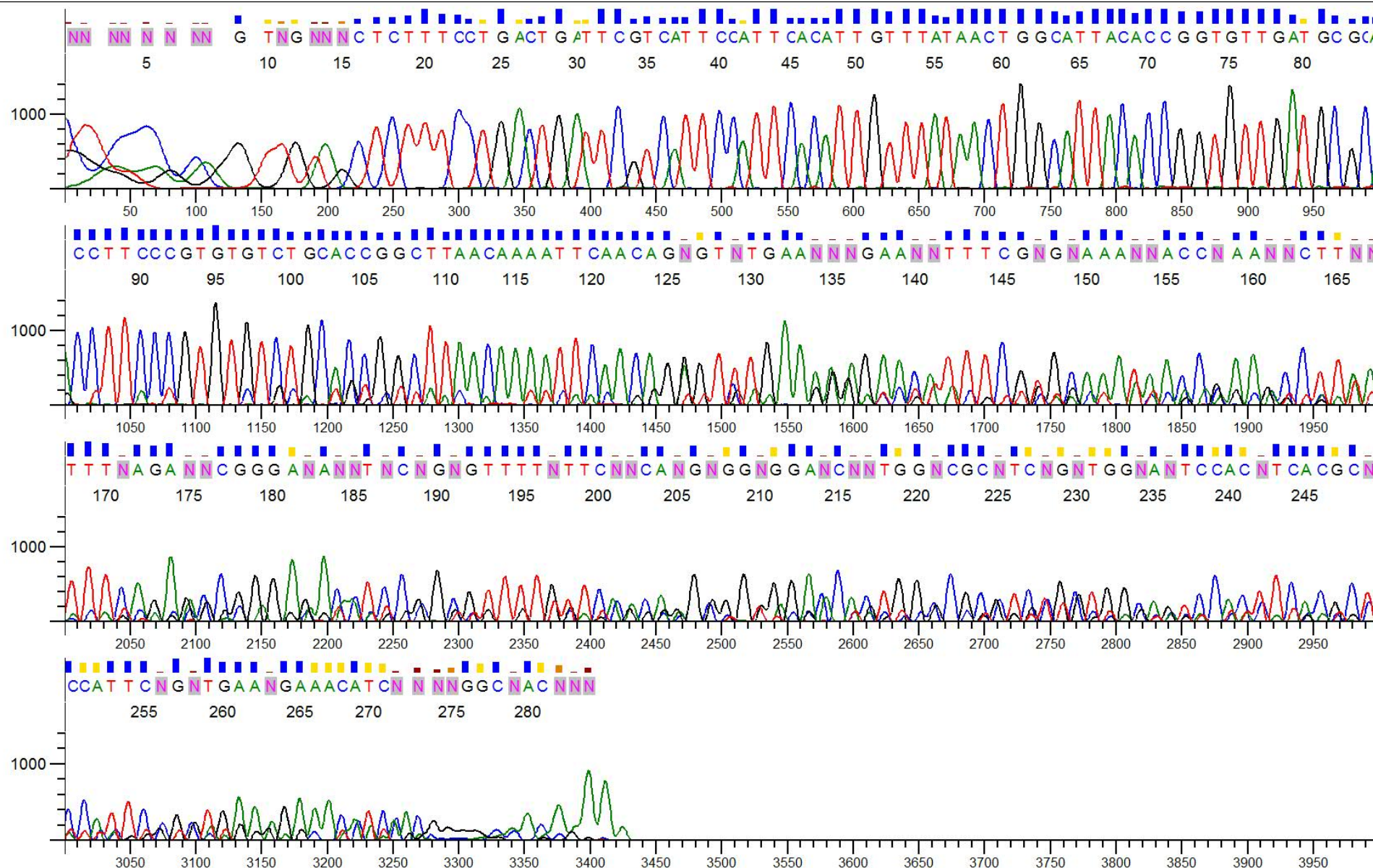

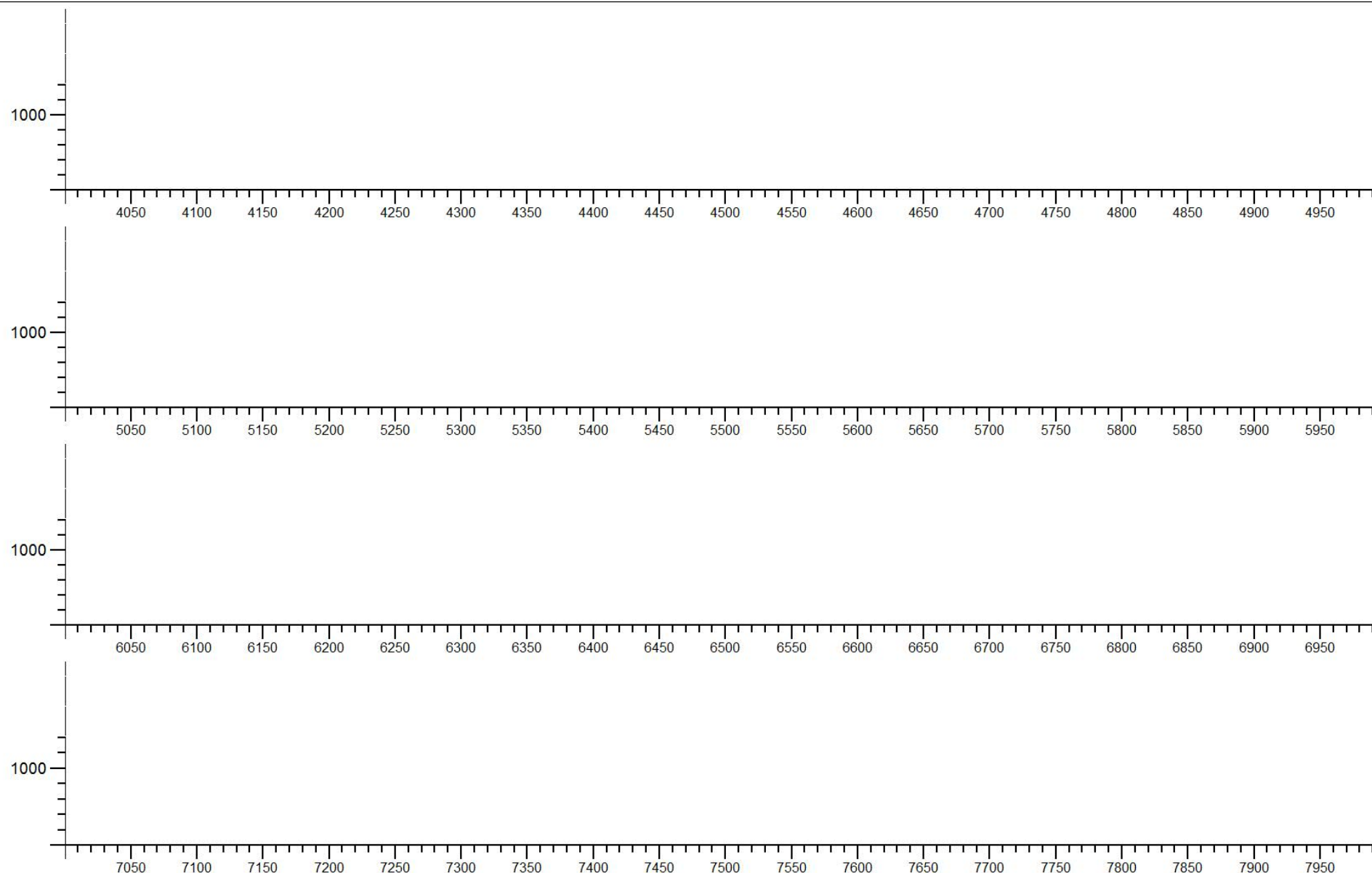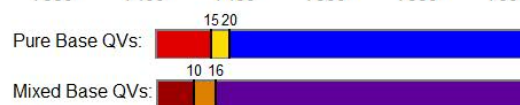

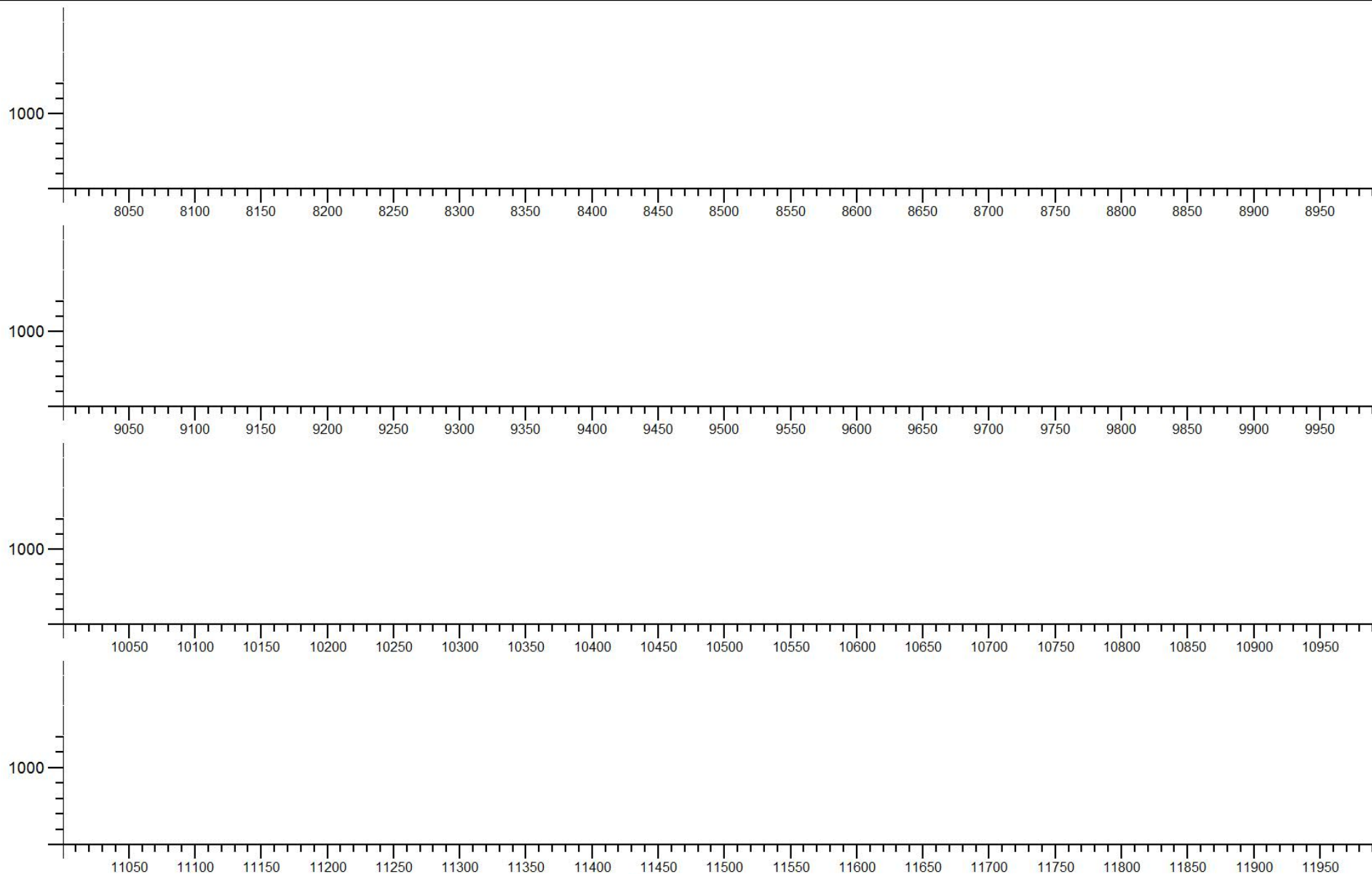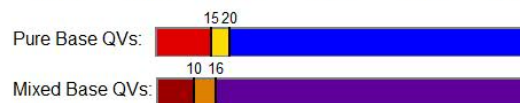

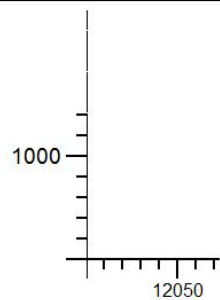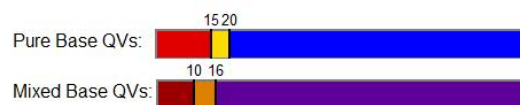

Supplement: Supplemental Information 1 — Chromatograms of: (1) recombined sequences of the H47 GI model from a number of mutants affected in recombination functions, and (2) recombined sequences of the pUYFRT model. [file peerj-05-3293-s001.zip › raw material/2-RecO_out1_FA.pdf]

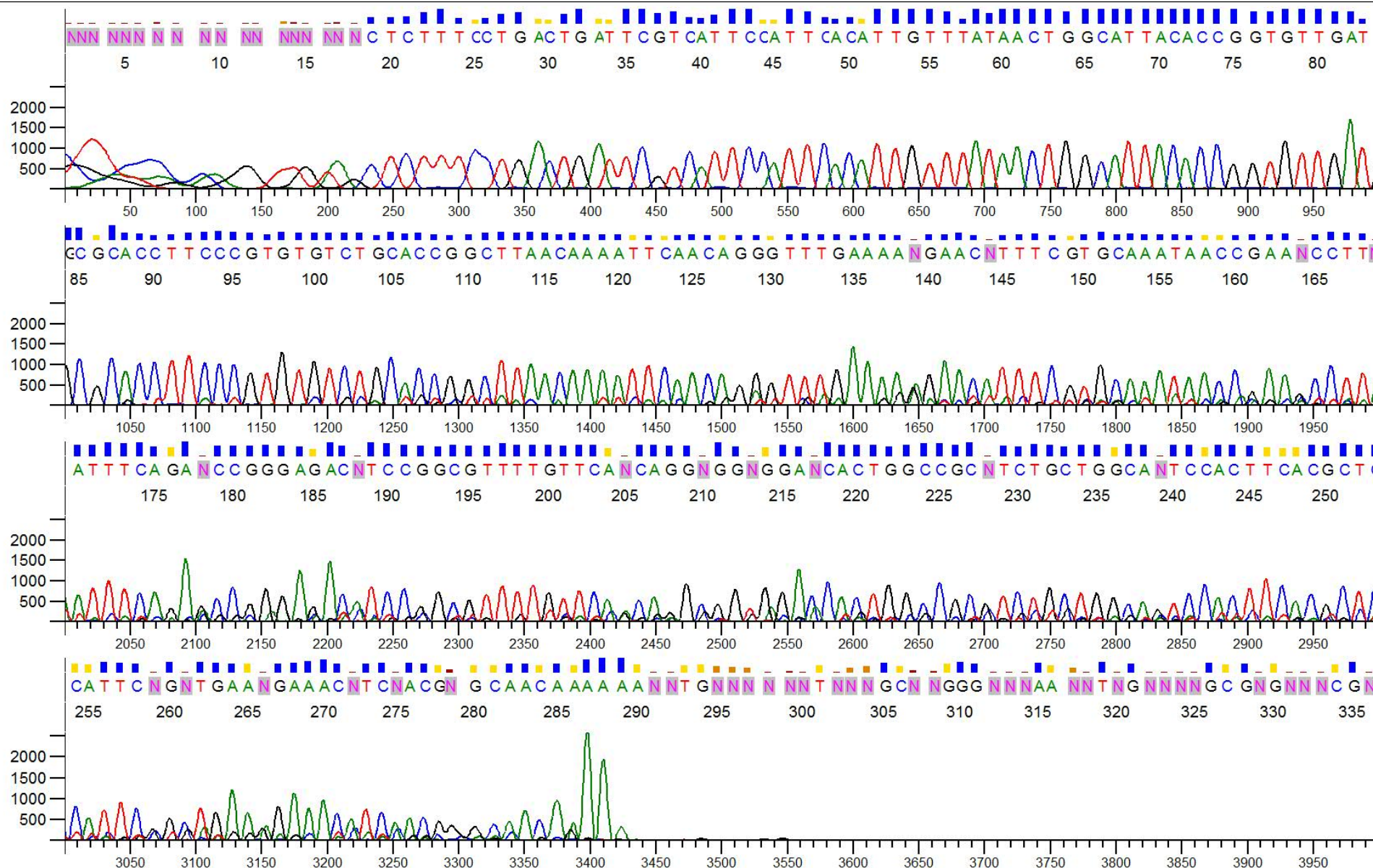

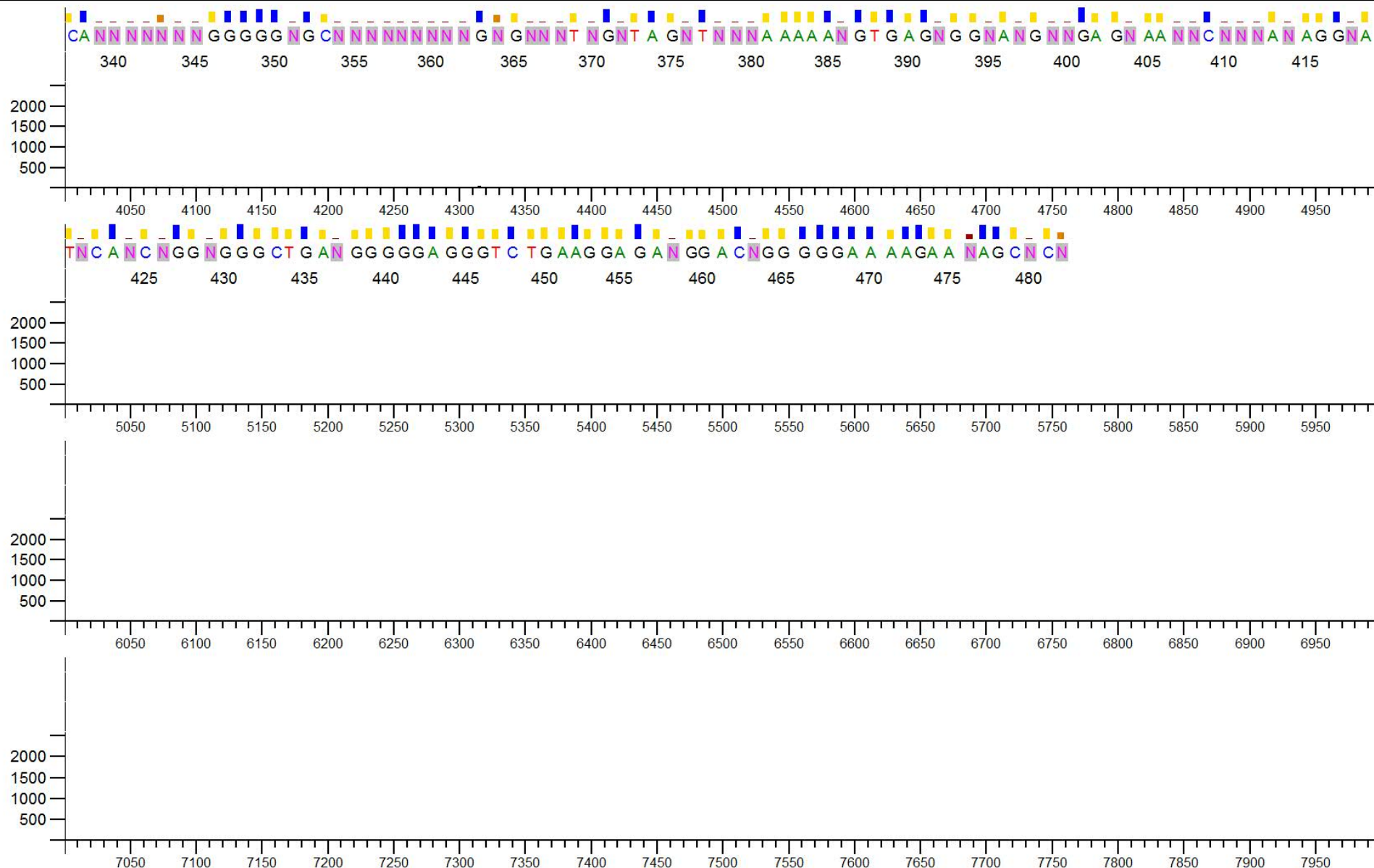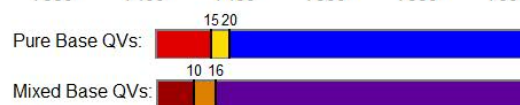

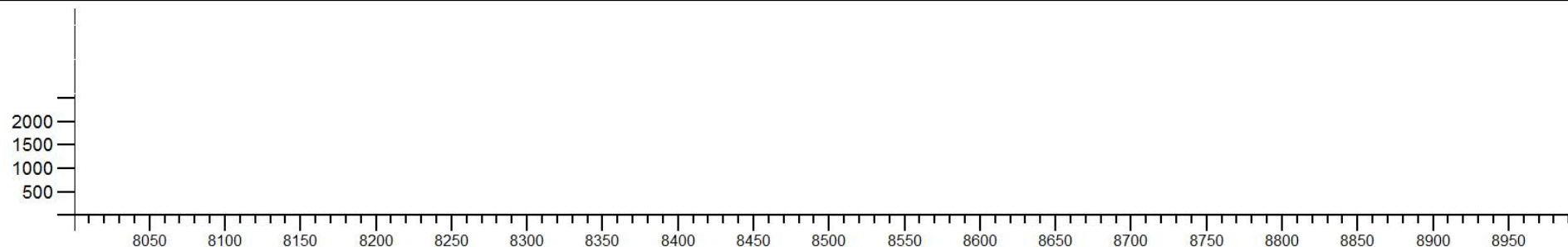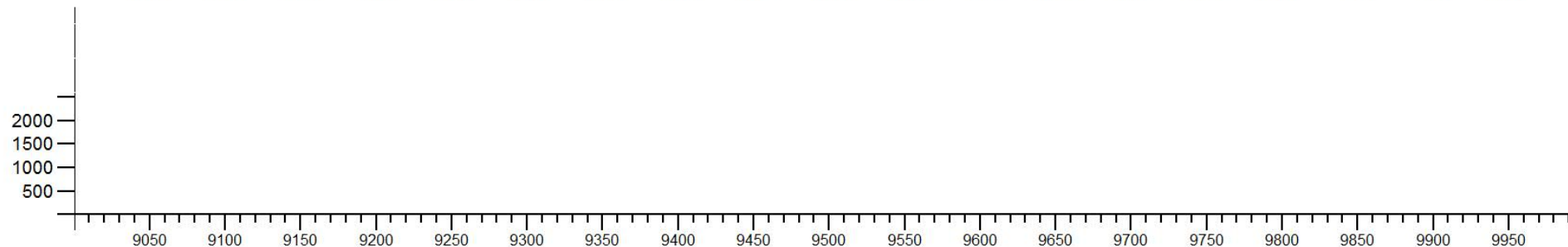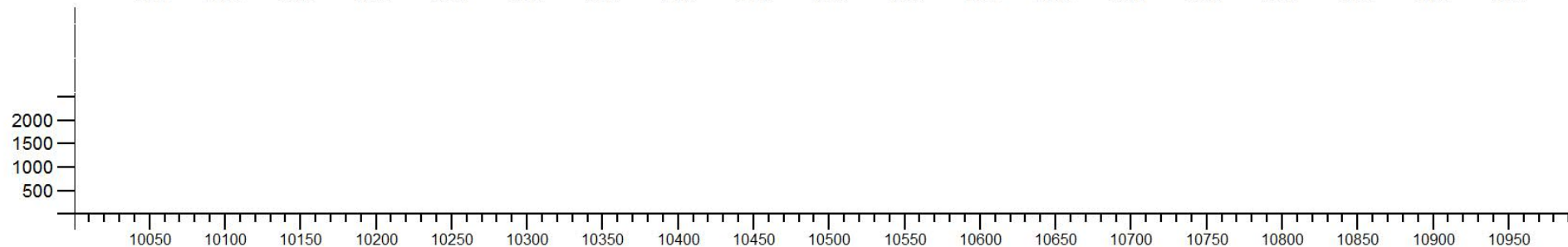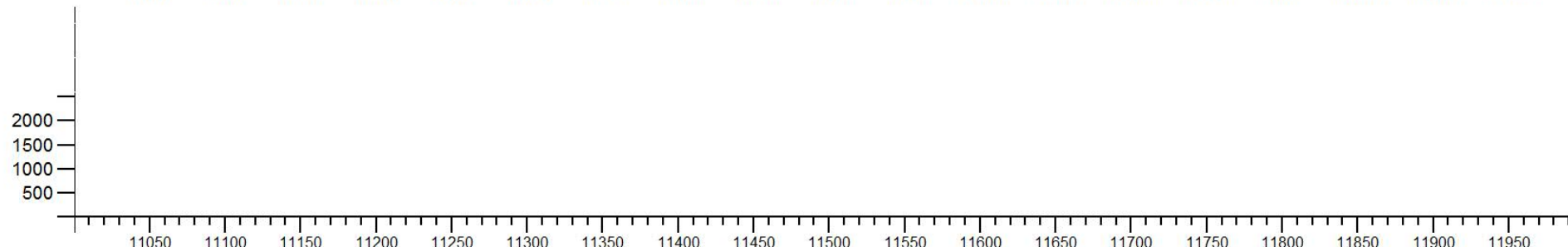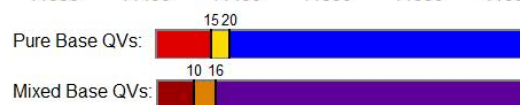

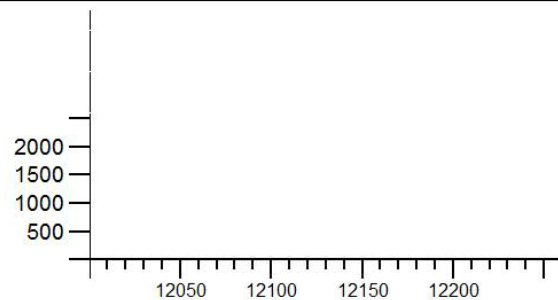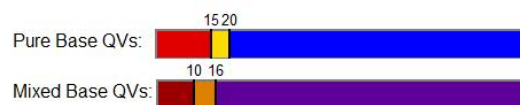

Supplement: Supplemental Information 1 — Chromatograms of: (1) recombined sequences of the H47 GI model from a number of mutants affected in recombination functions, and (2) recombined sequences of the pUYFRT model. [file peerj-05-3293-s001.zip › raw material/30-intRQ_out1_FA.pdf]

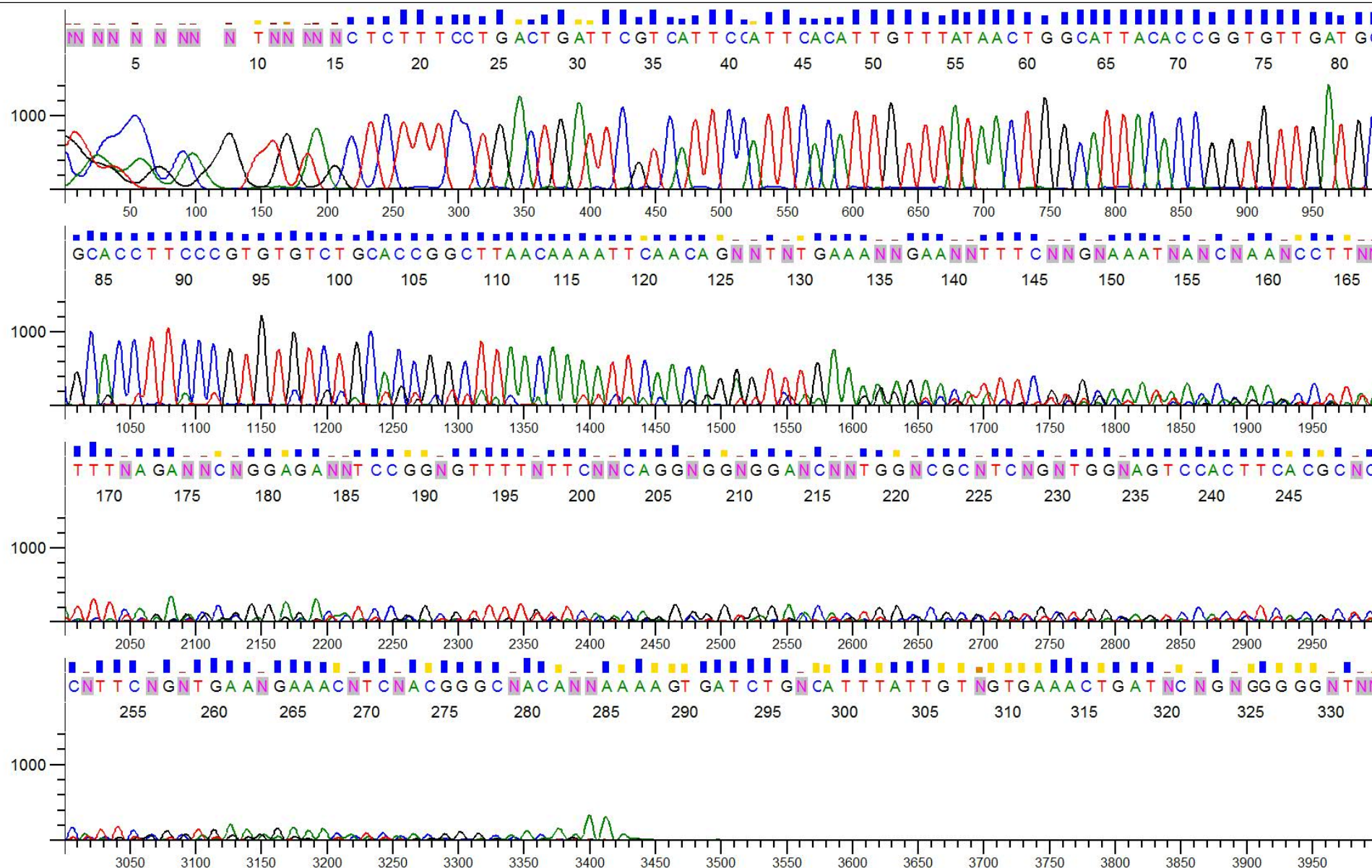

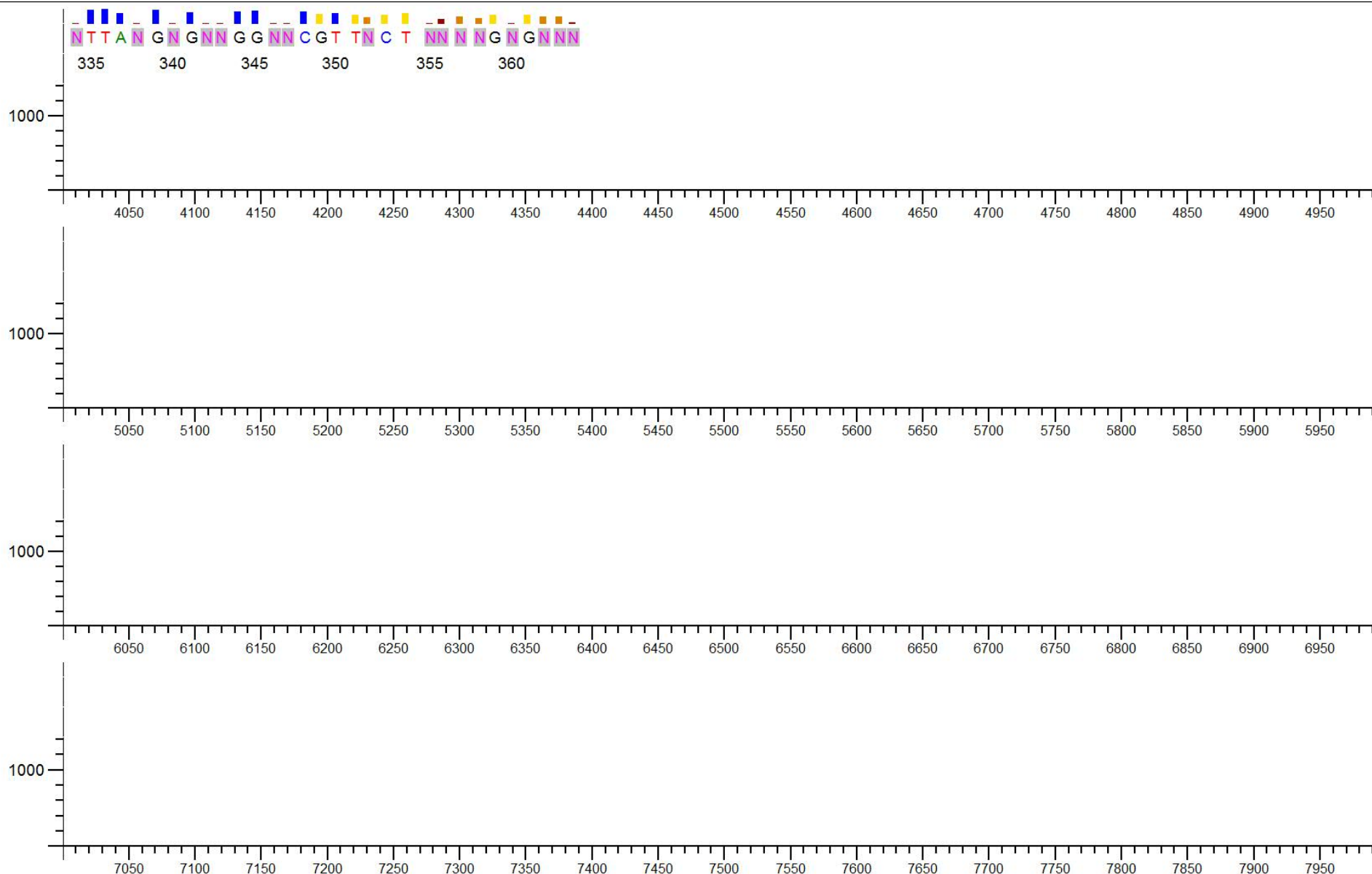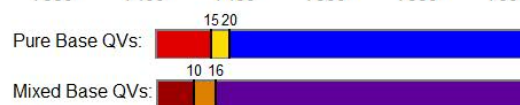

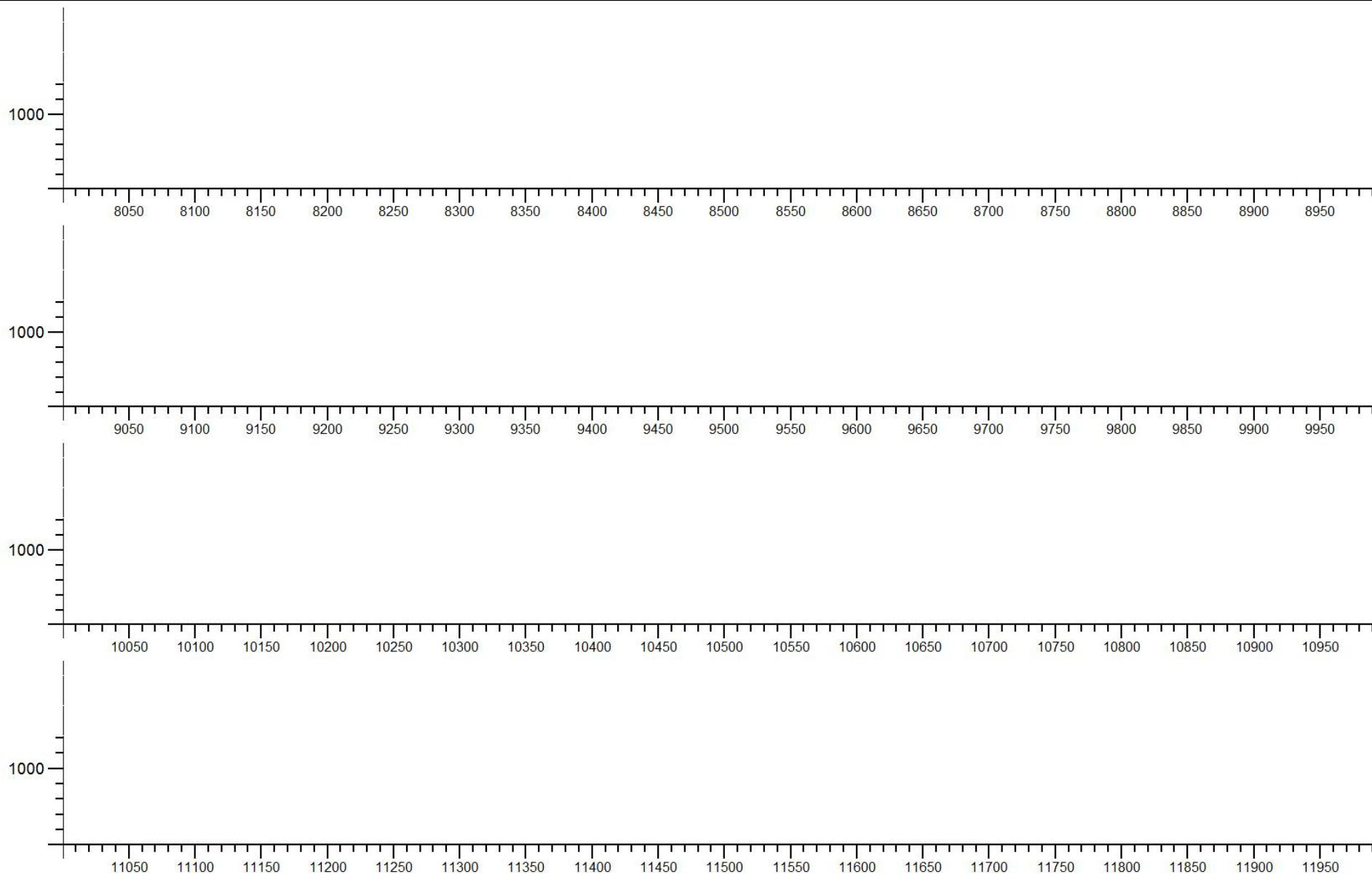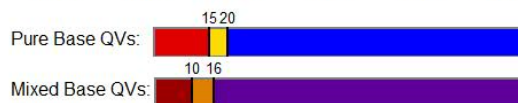

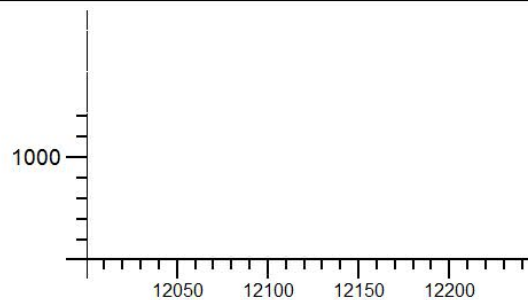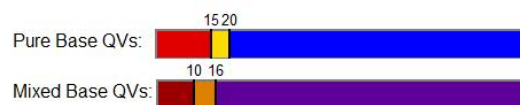

Supplement: Supplemental Information 1 — Chromatograms of: (1) recombined sequences of the H47 GI model from a number of mutants affected in recombination functions, and (2) recombined sequences of the pUYFRT model. [file peerj-05-3293-s001.zip › raw material/30-ruvB_out1_FA.pdf]

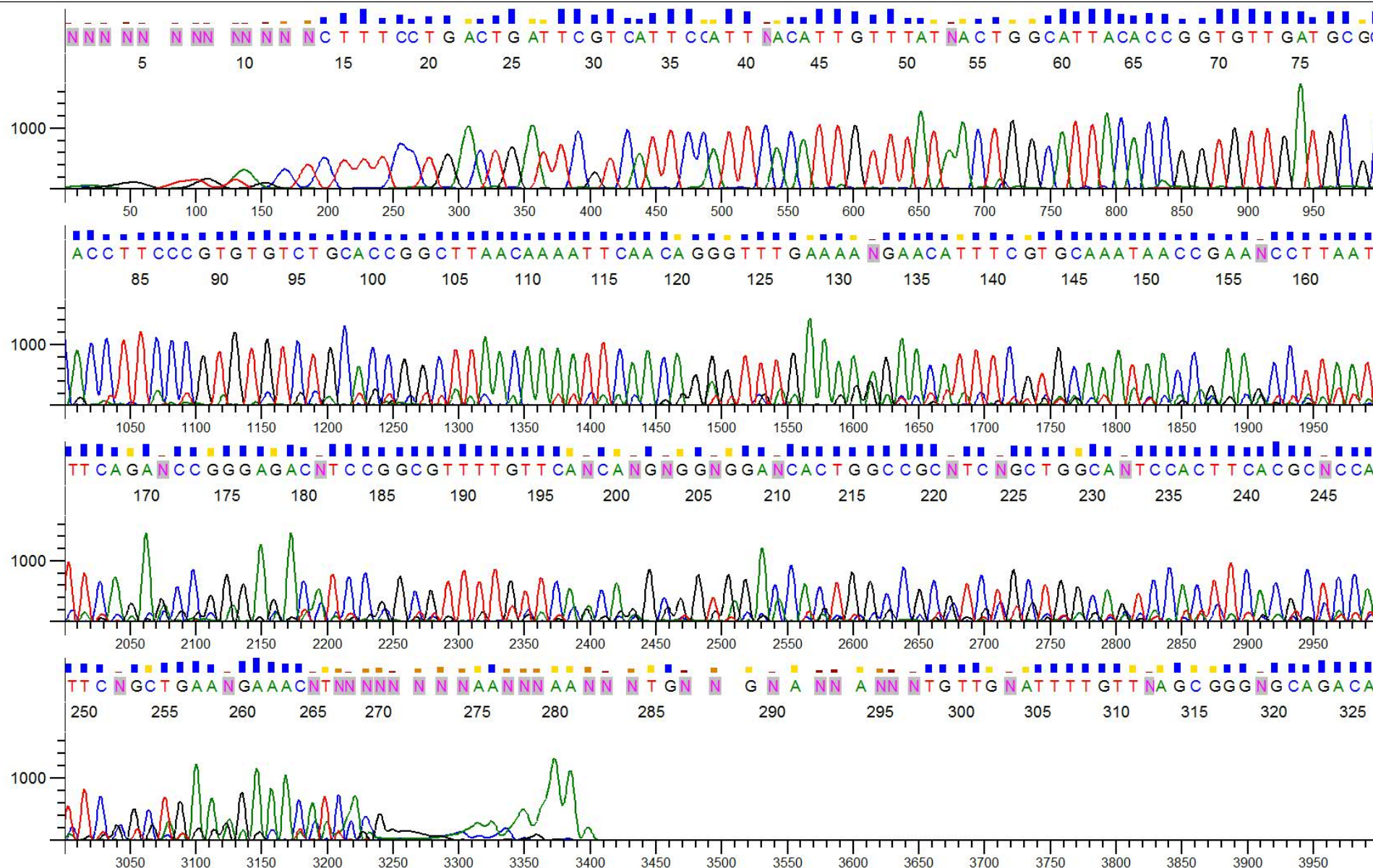

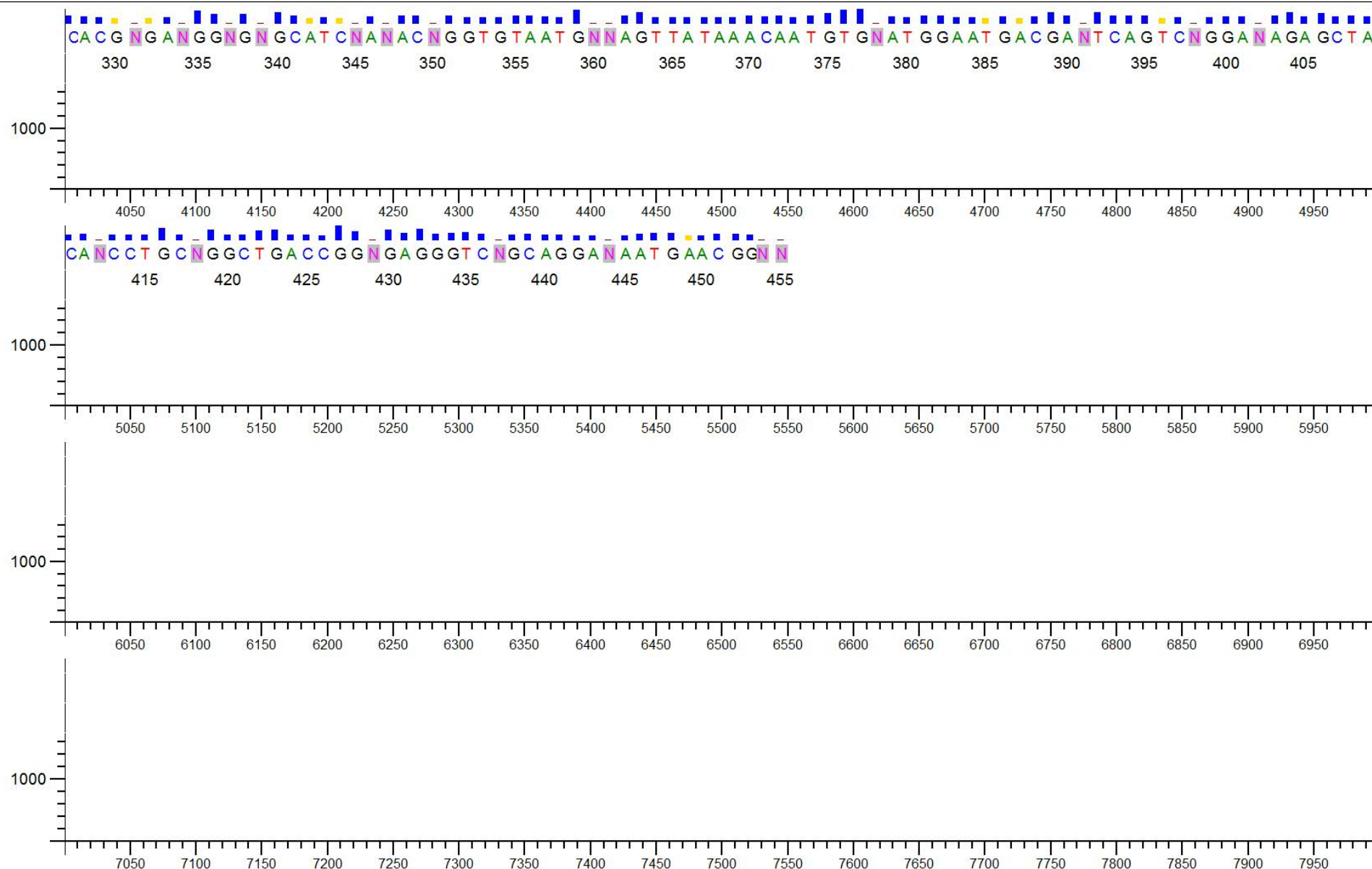

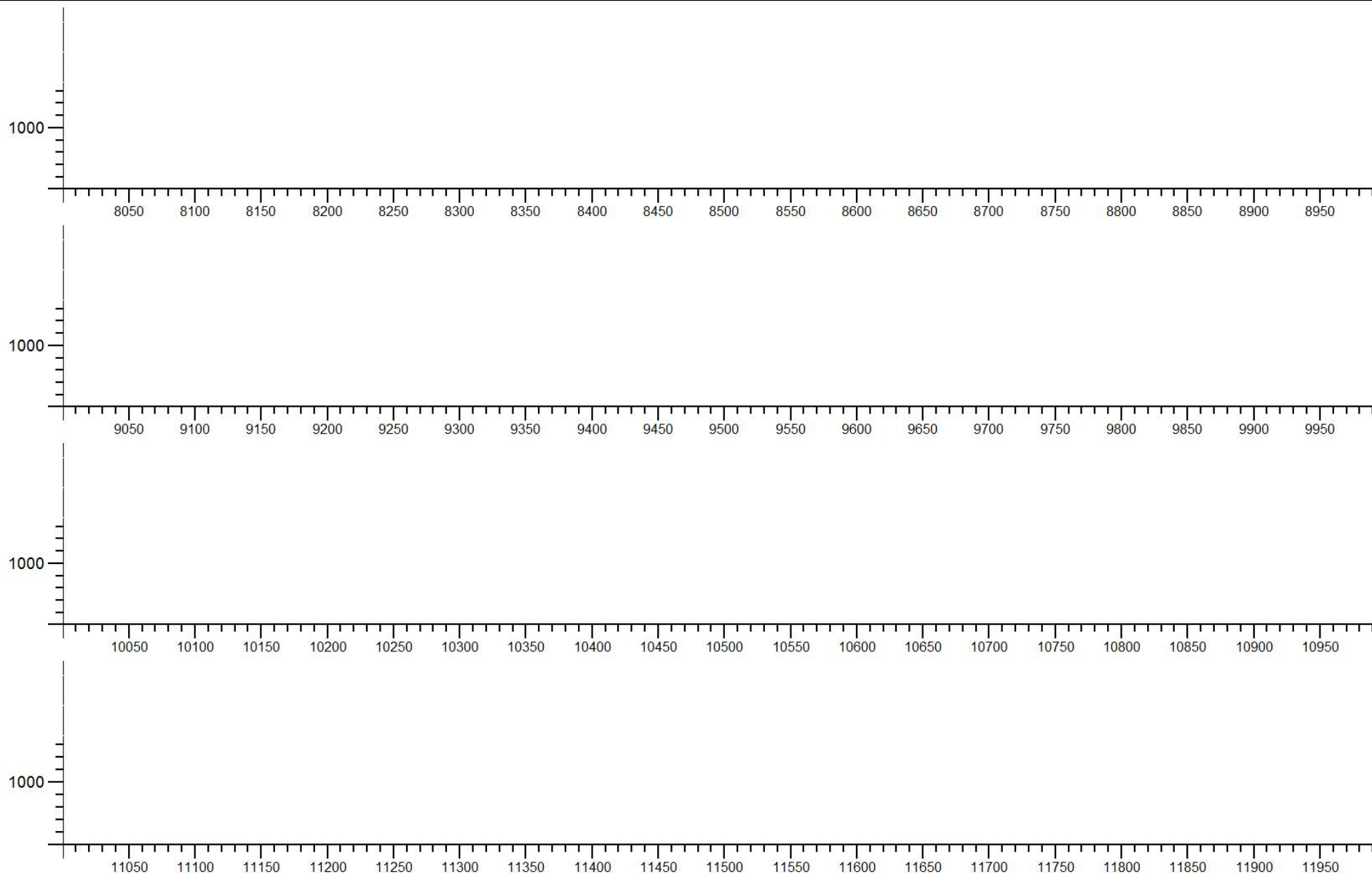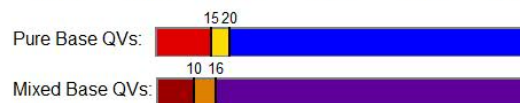

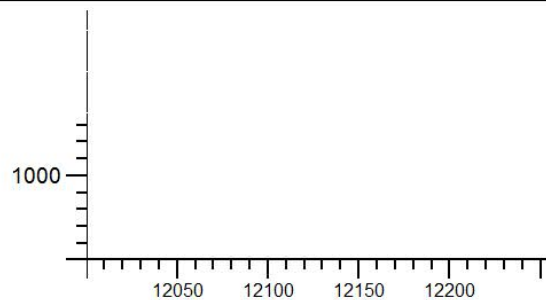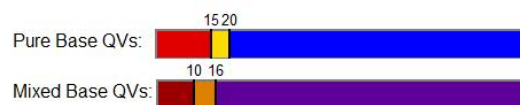

Supplement: Supplemental Information 1 — Chromatograms of: (1) recombined sequences of the H47 GI model from a number of mutants affected in recombination functions, and (2) recombined sequences of the pUYFRT model. [file peerj-05-3293-s001.zip › raw material/31-intRA_out1_FA.pdf]

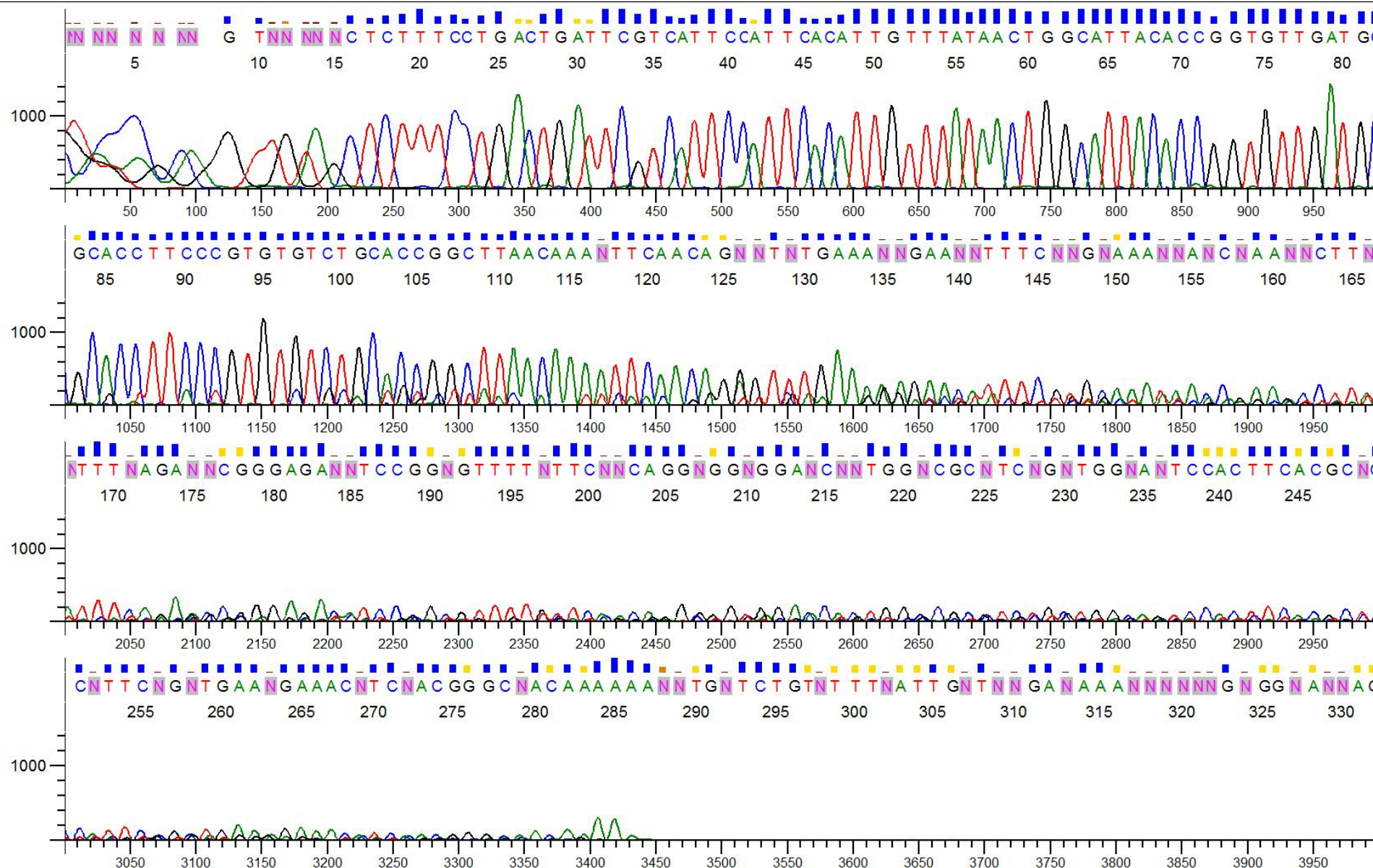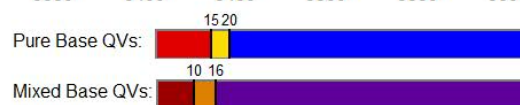

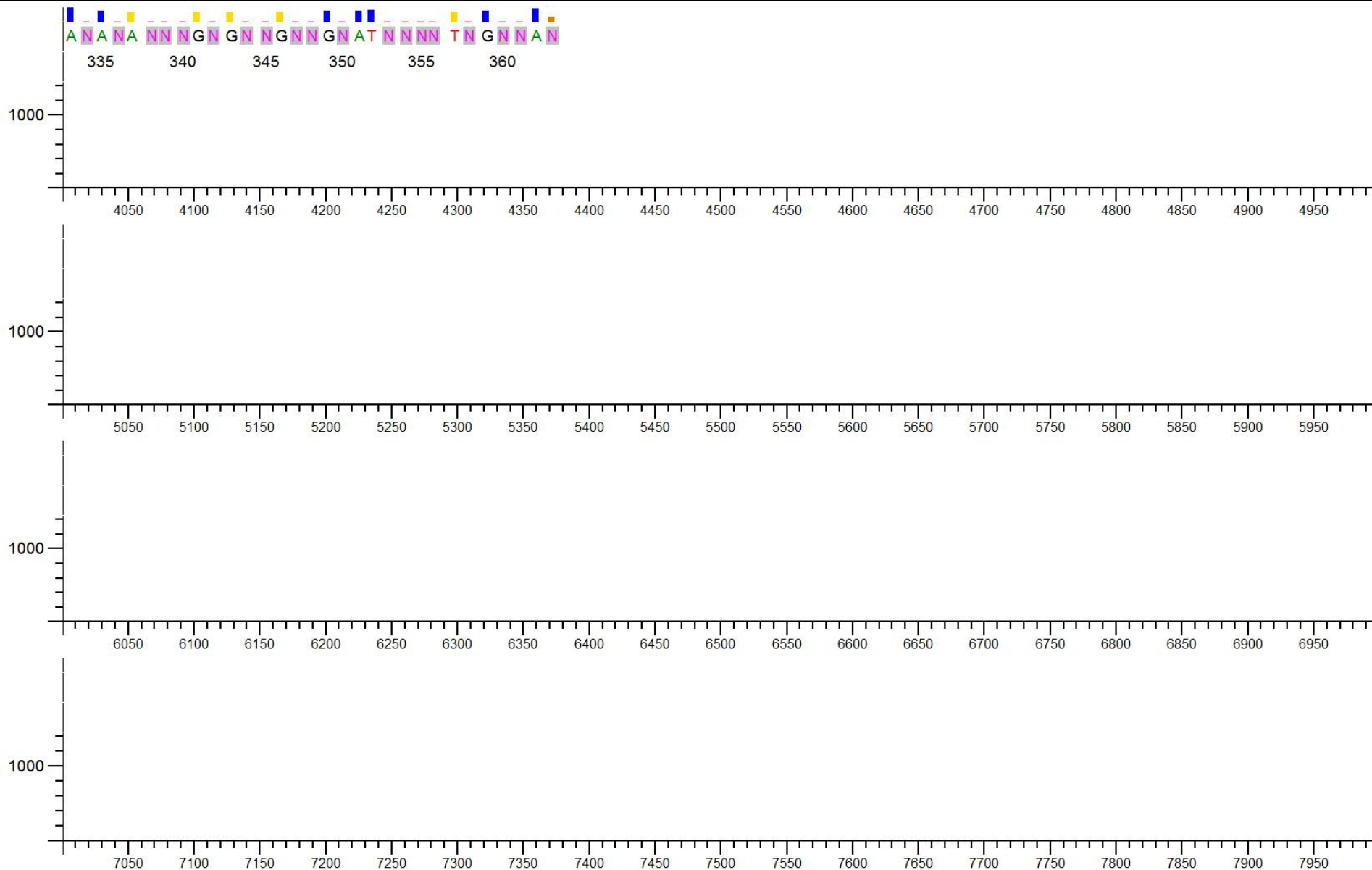

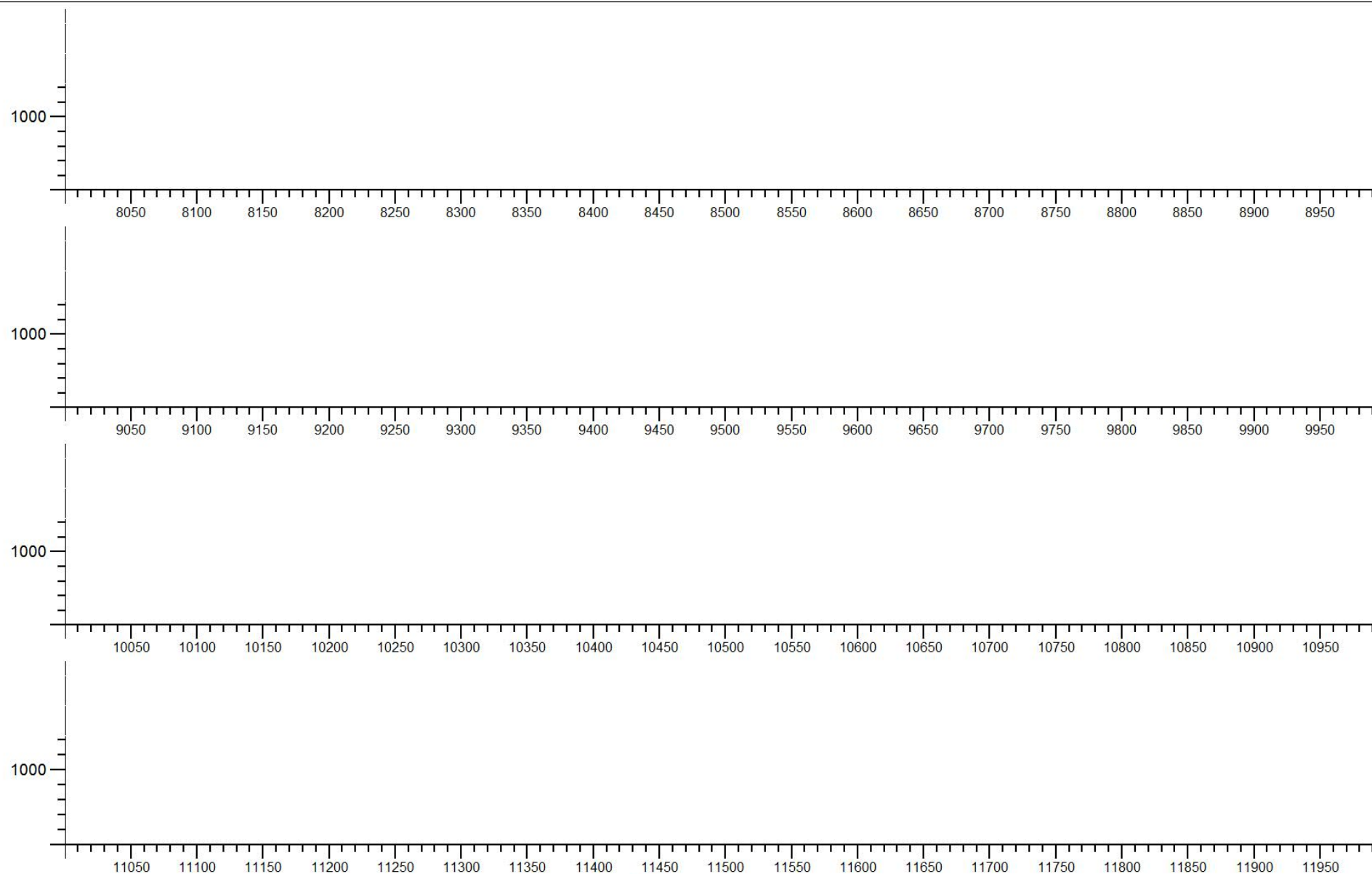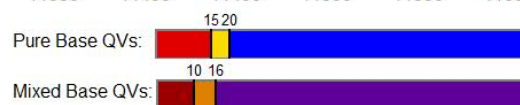

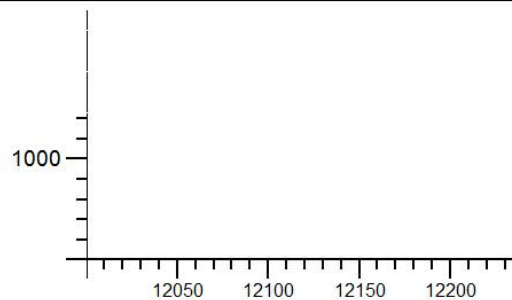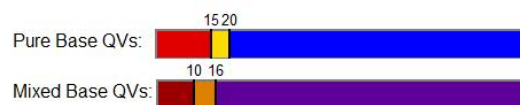

Supplement: Supplemental Information 1 — Chromatograms of: (1) recombined sequences of the H47 GI model from a number of mutants affected in recombination functions, and (2) recombined sequences of the pUYFRT model. [file peerj-05-3293-s001.zip › raw material/31-ruvC_out1_FA.pdf]

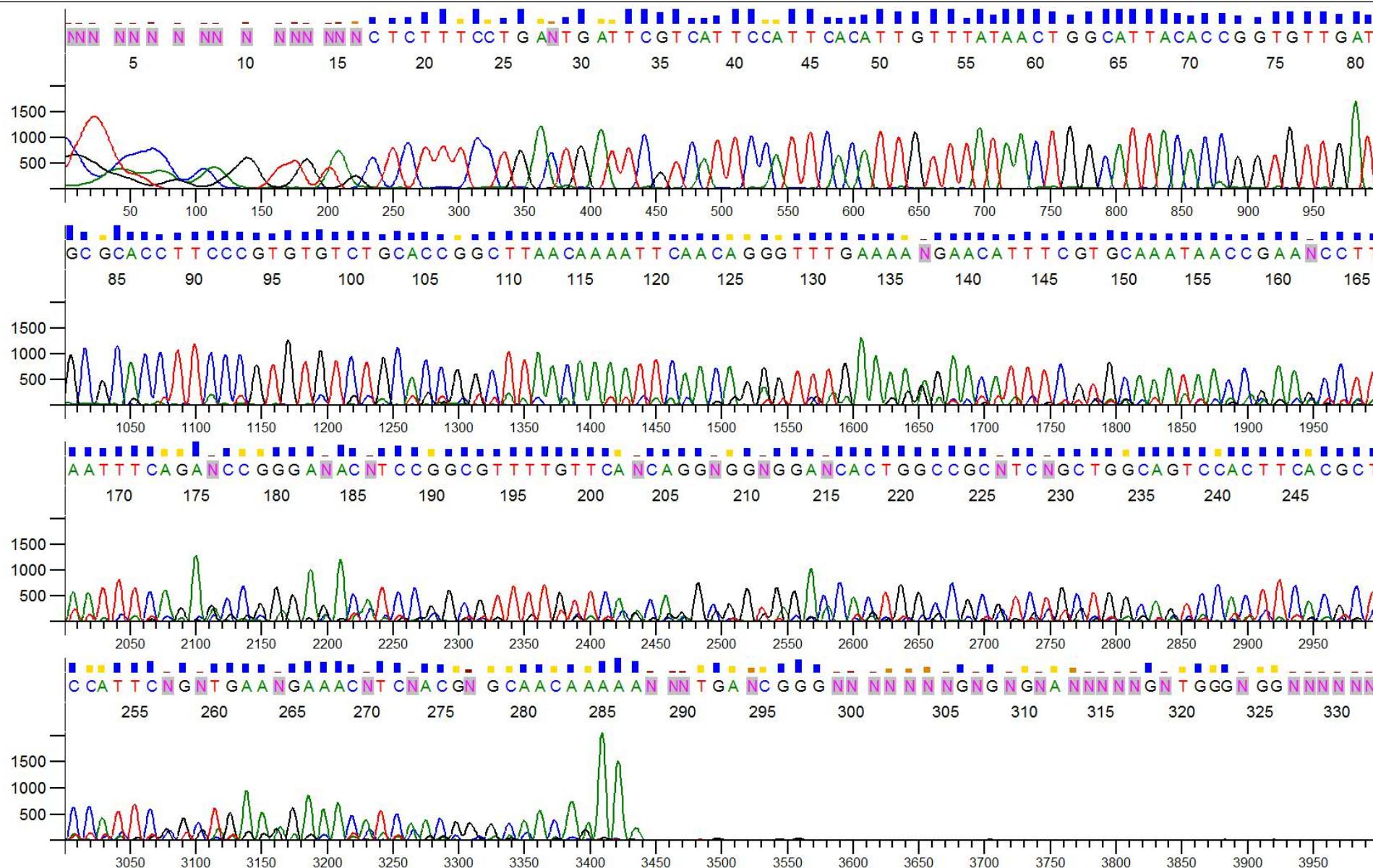

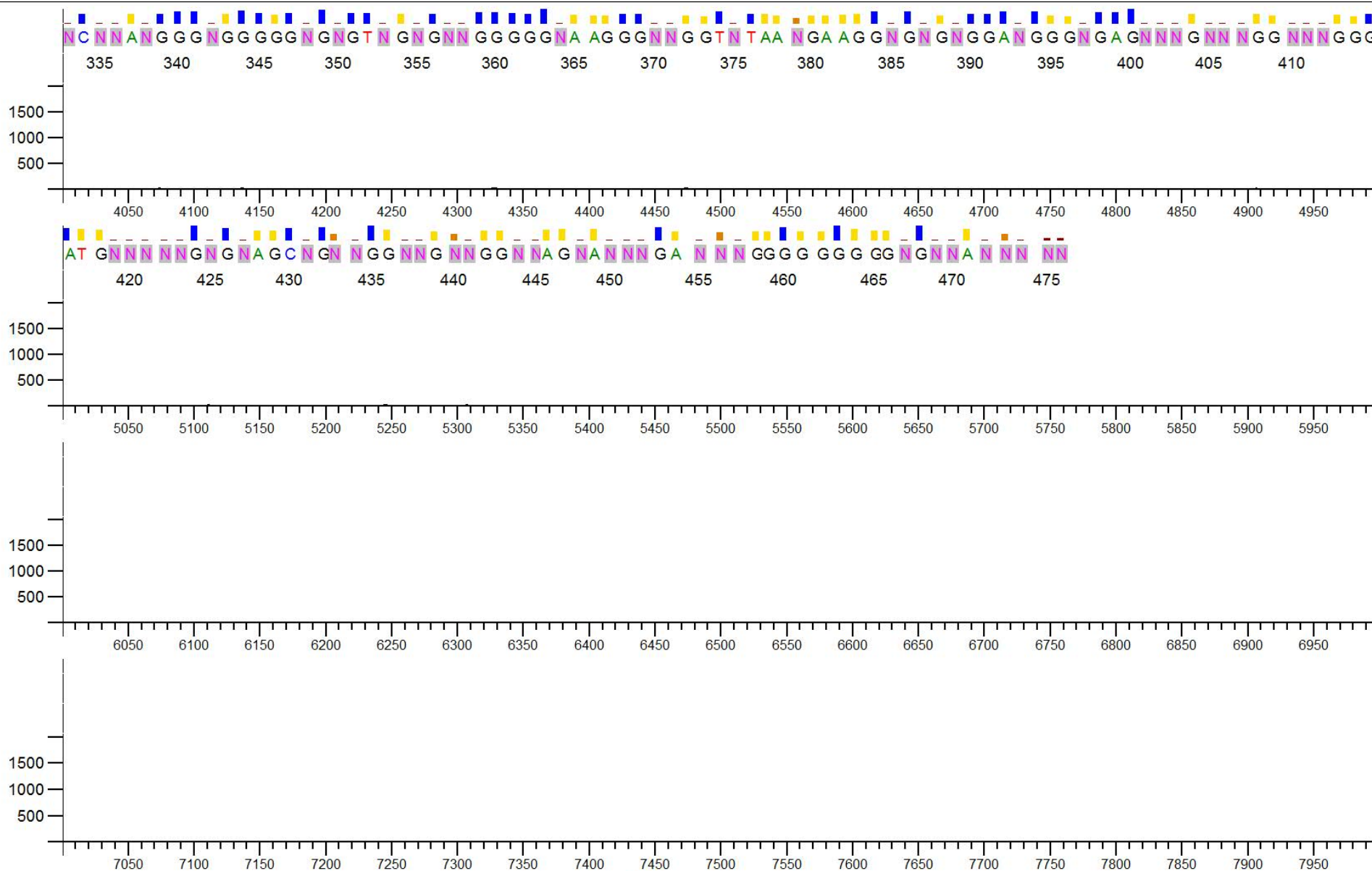

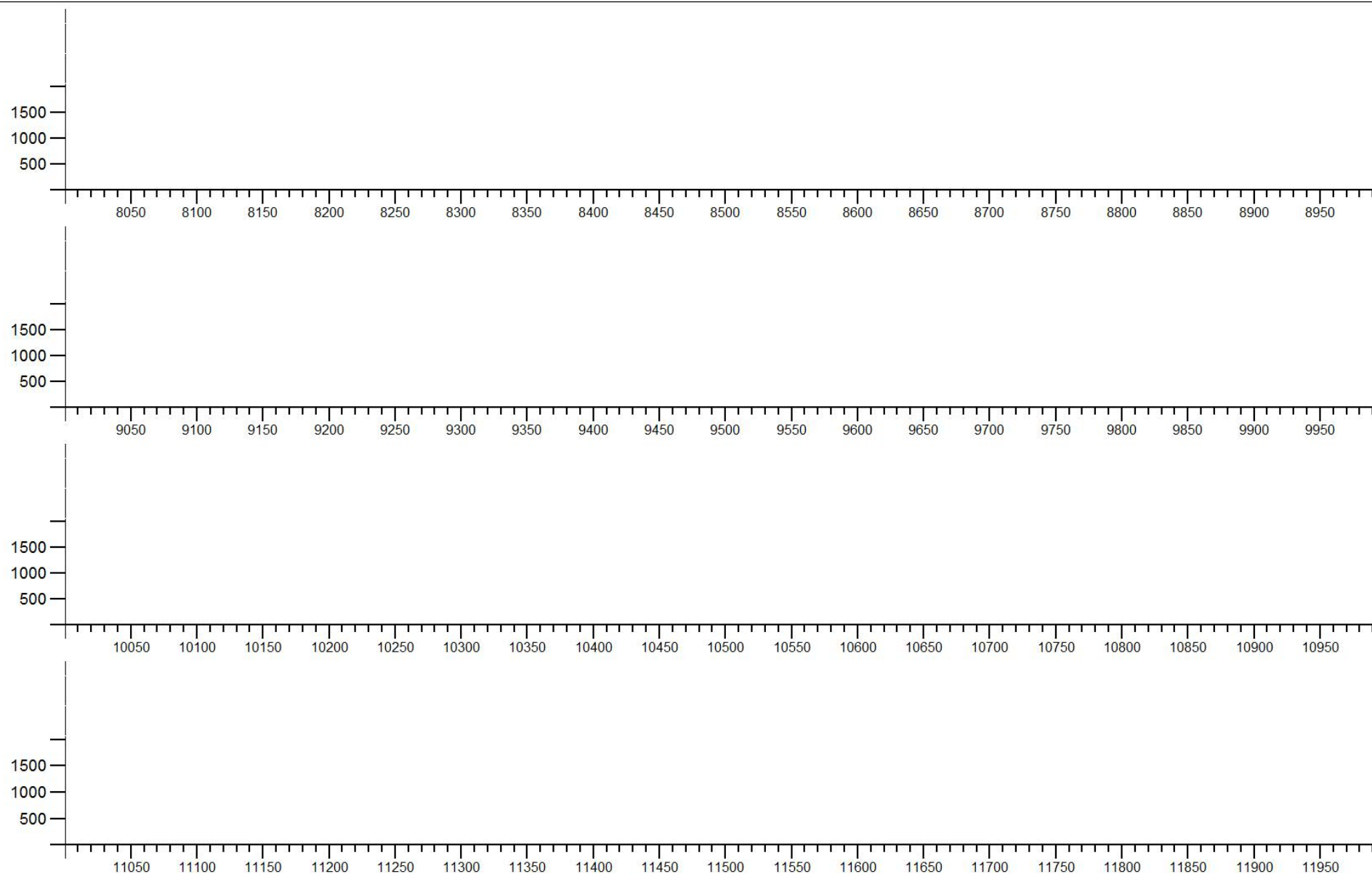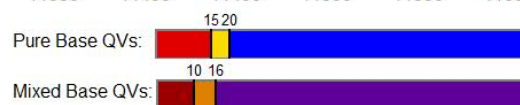

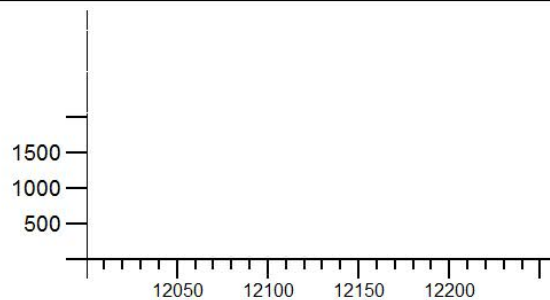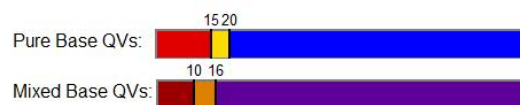

Supplement: Supplemental Information 1 — Chromatograms of: (1) recombined sequences of the H47 GI model from a number of mutants affected in recombination functions, and (2) recombined sequences of the pUYFRT model. [file peerj-05-3293-s001.zip › raw material/32-pinQpinRybcK_out1_FA.pdf]

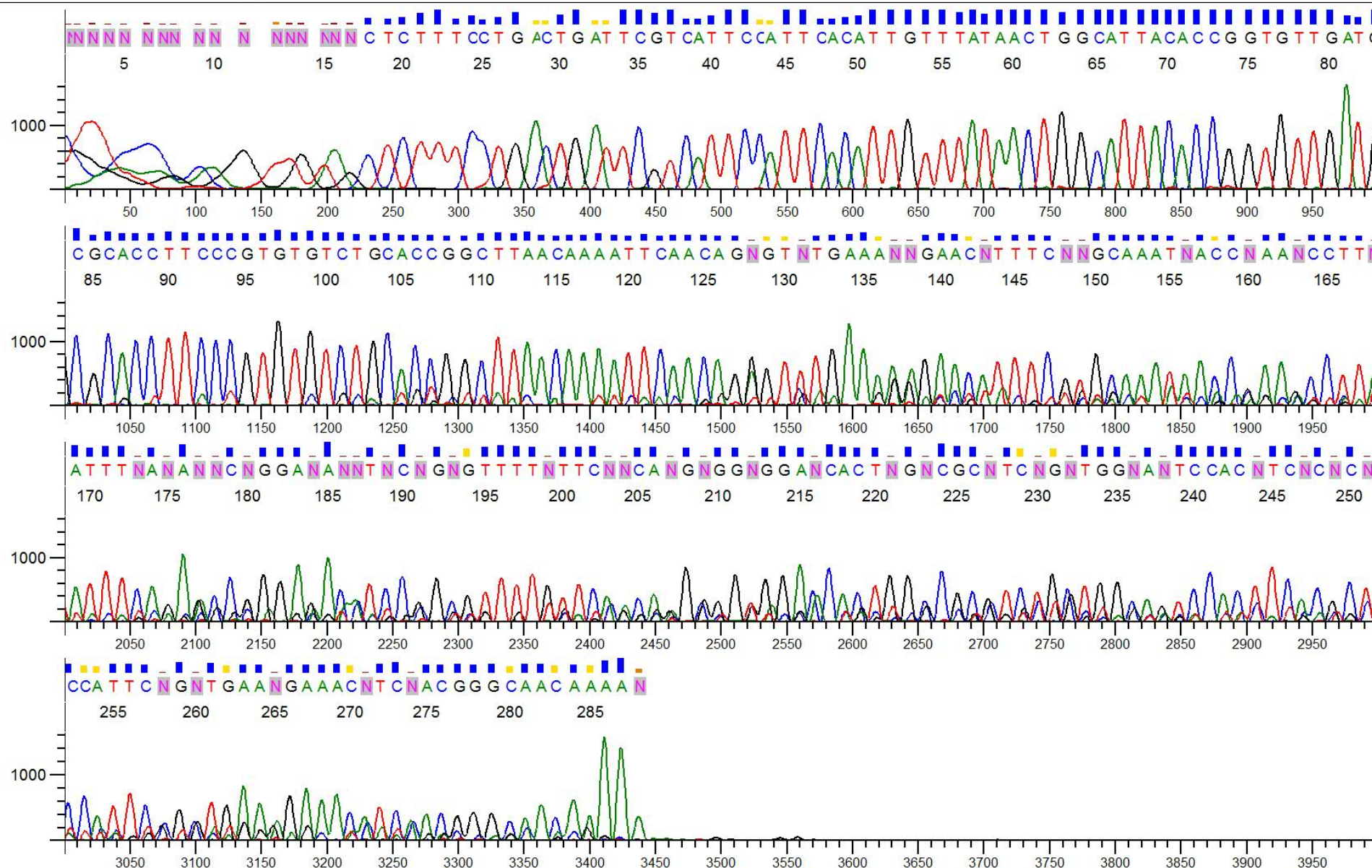

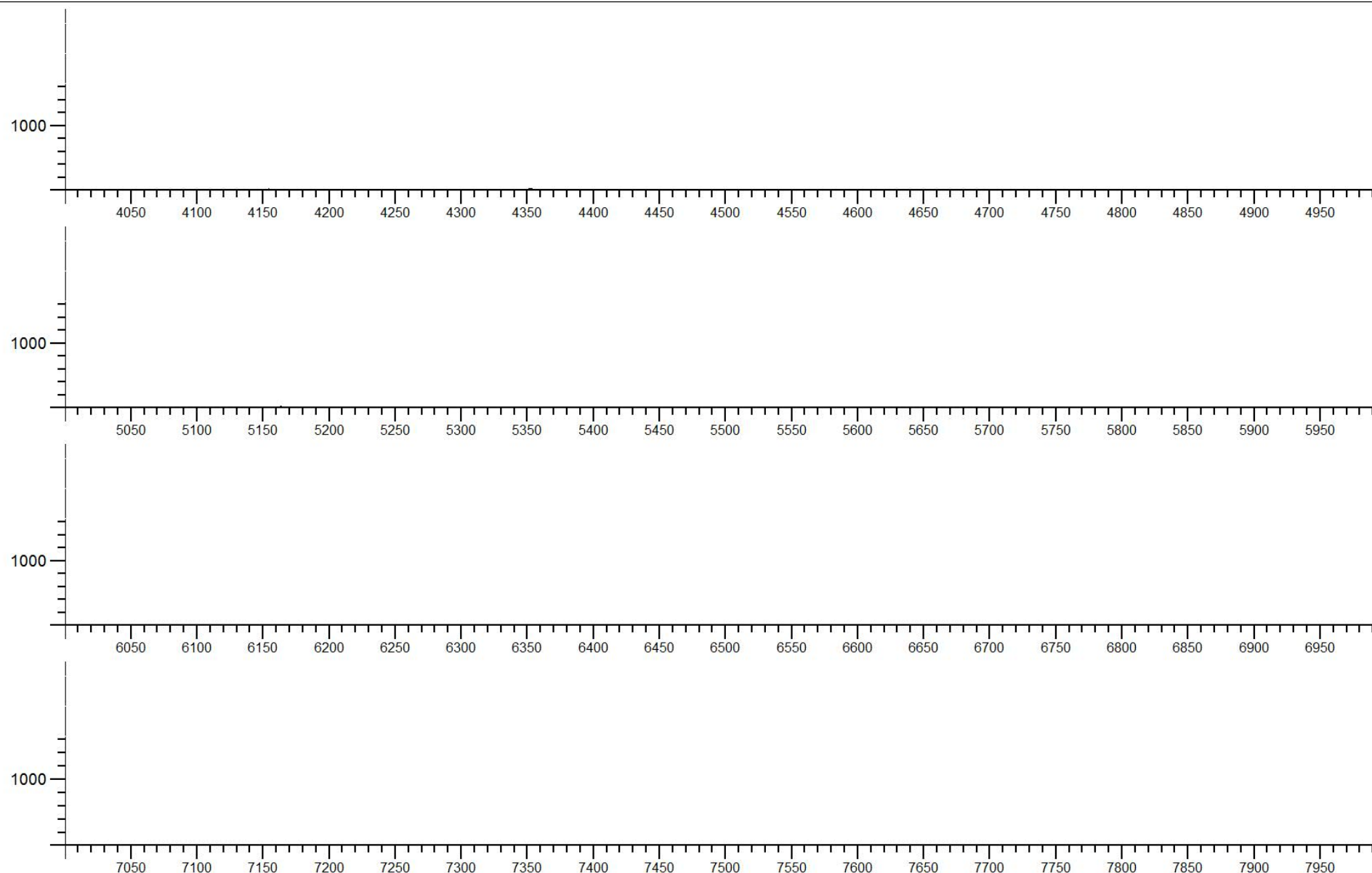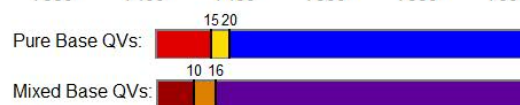

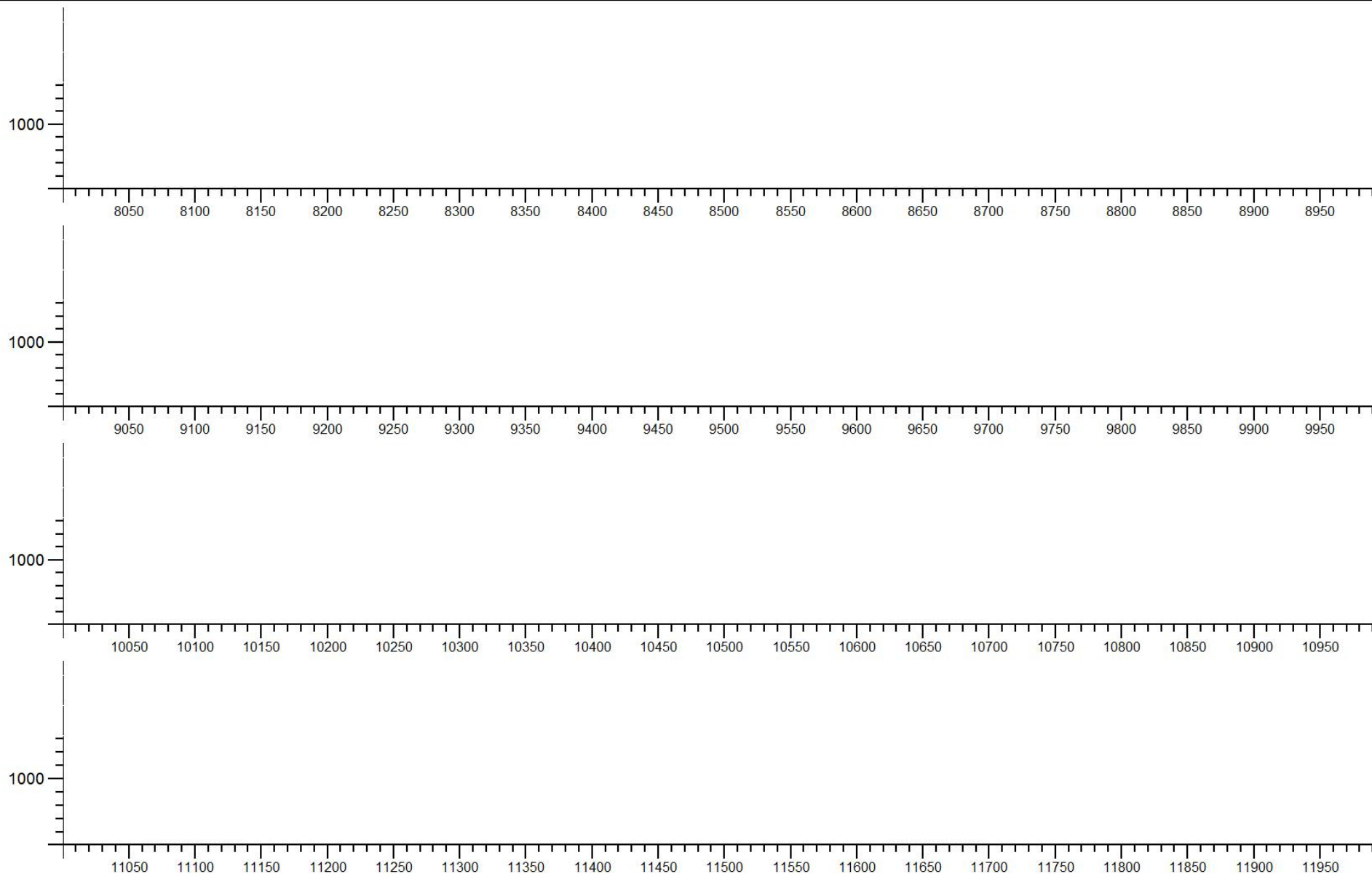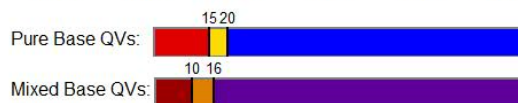

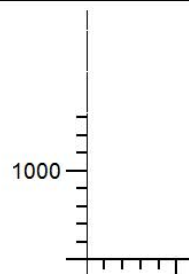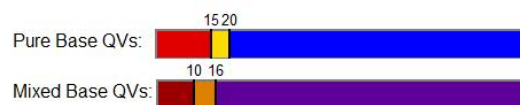

Supplement: Supplemental Information 1 — Chromatograms of: (1) recombined sequences of the H47 GI model from a number of mutants affected in recombination functions, and (2) recombined sequences of the pUYFRT model. [file peerj-05-3293-s001.zip › raw material/38-RecT_out1_FA.pdf]

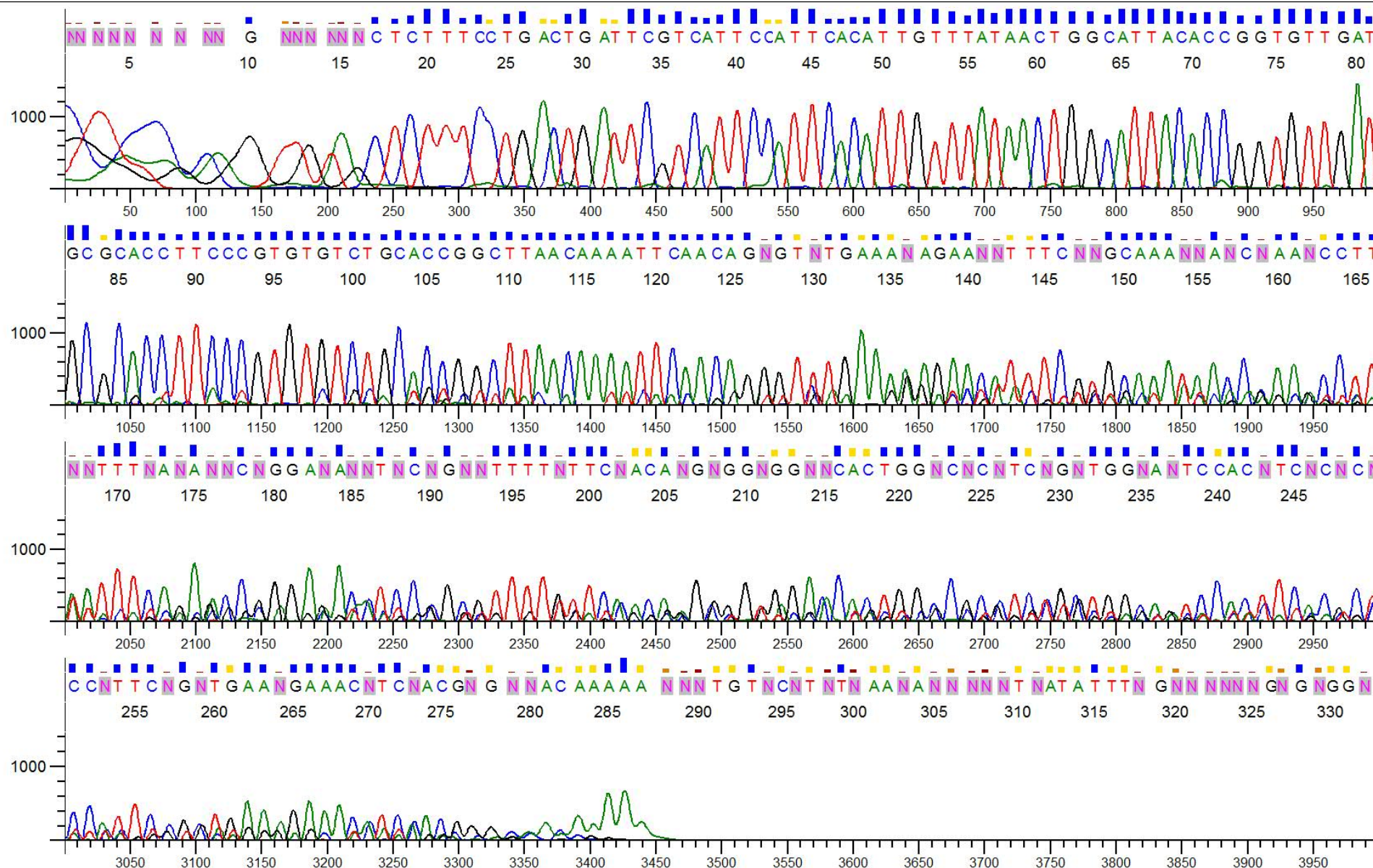

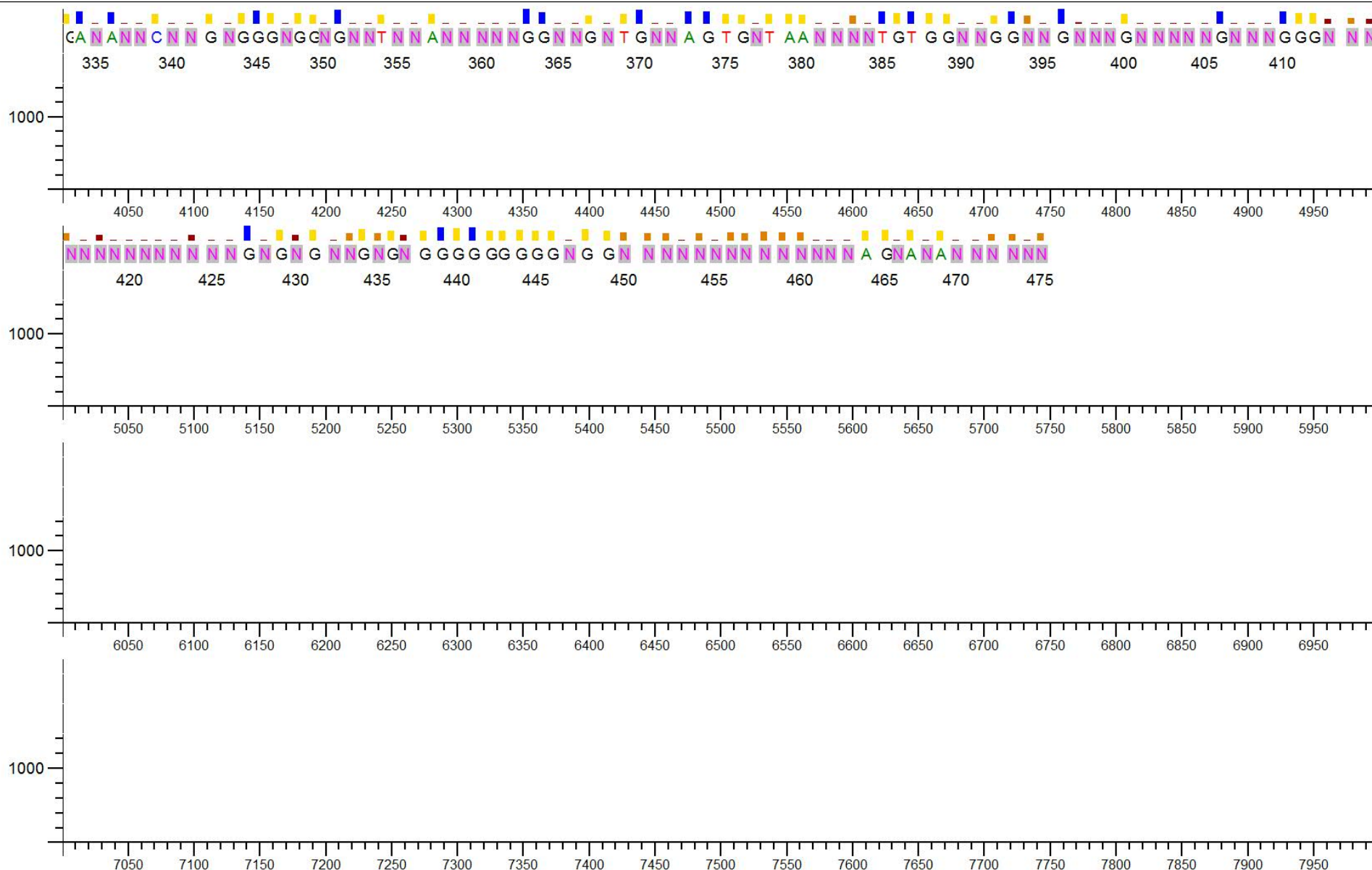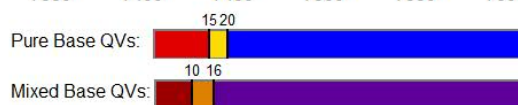

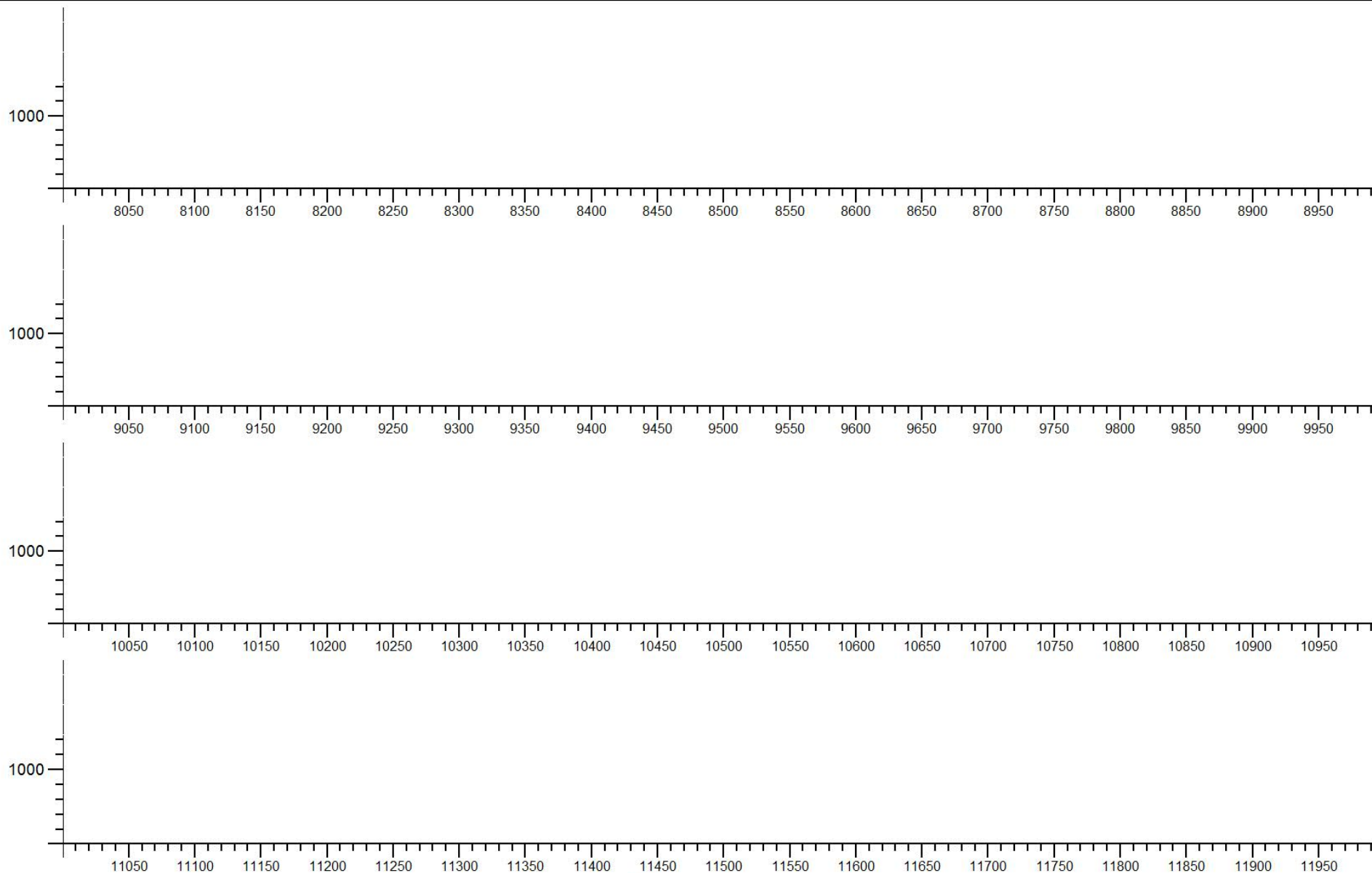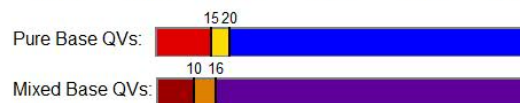

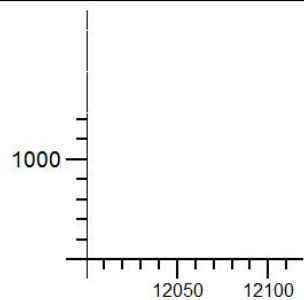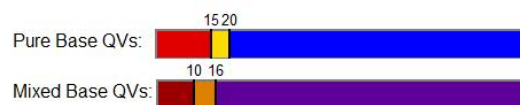

Supplement: Supplemental Information 1 — Chromatograms of: (1) recombined sequences of the H47 GI model from a number of mutants affected in recombination functions, and (2) recombined sequences of the pUYFRT model. [file peerj-05-3293-s001.zip › raw material/40-RecN_out1_FA.pdf]

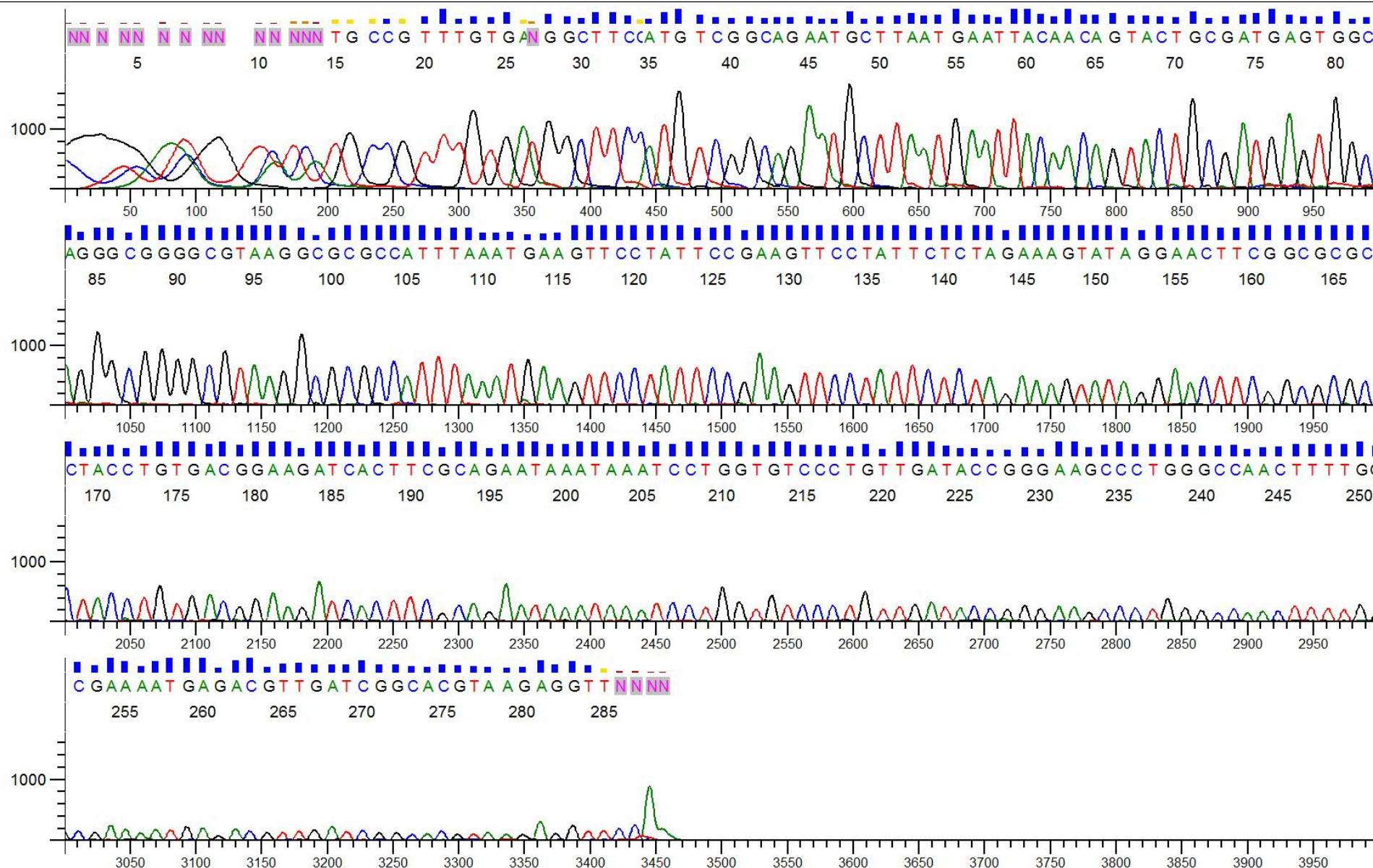

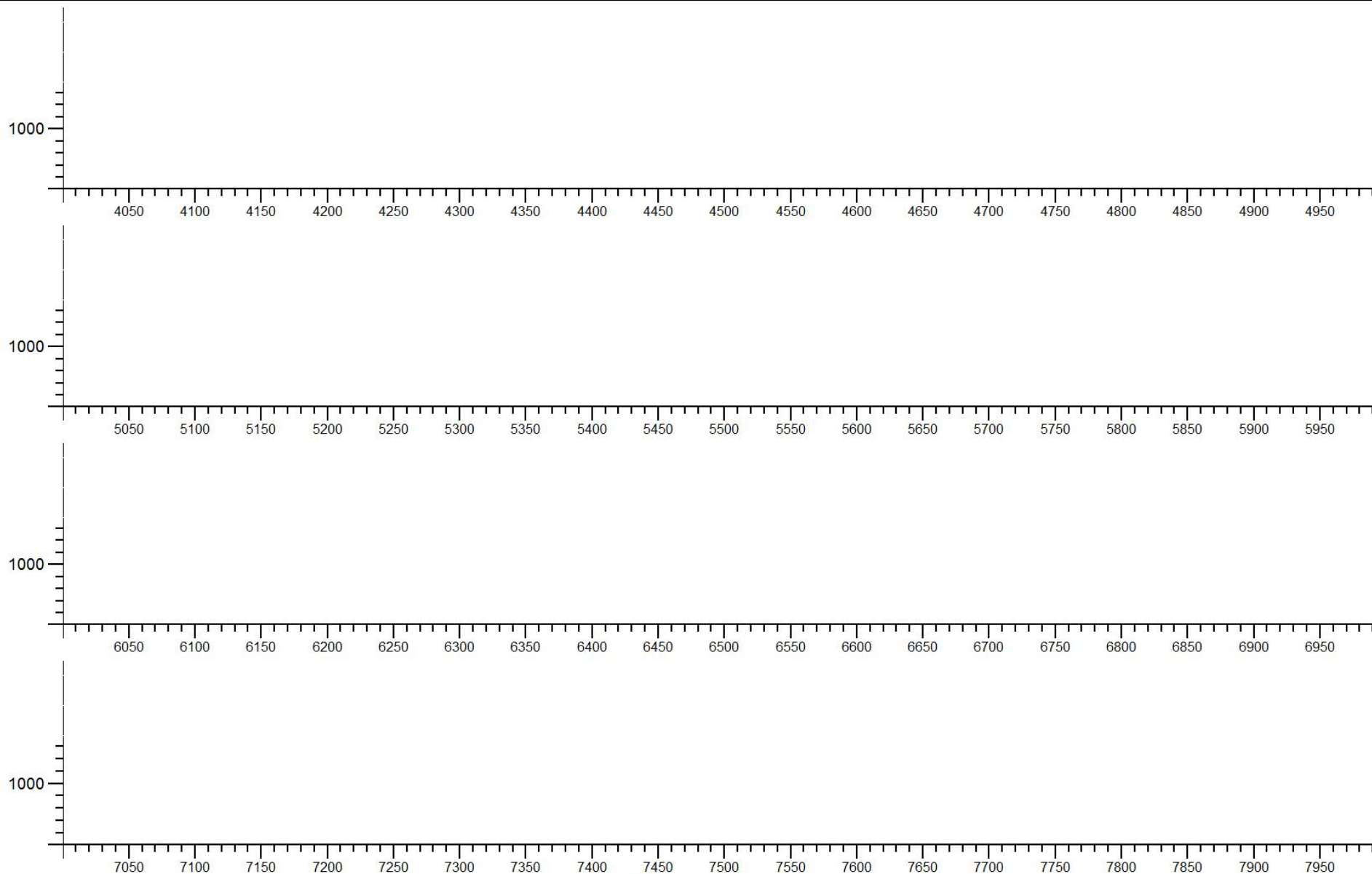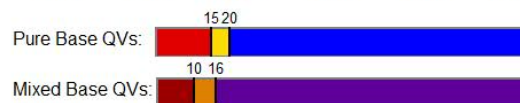

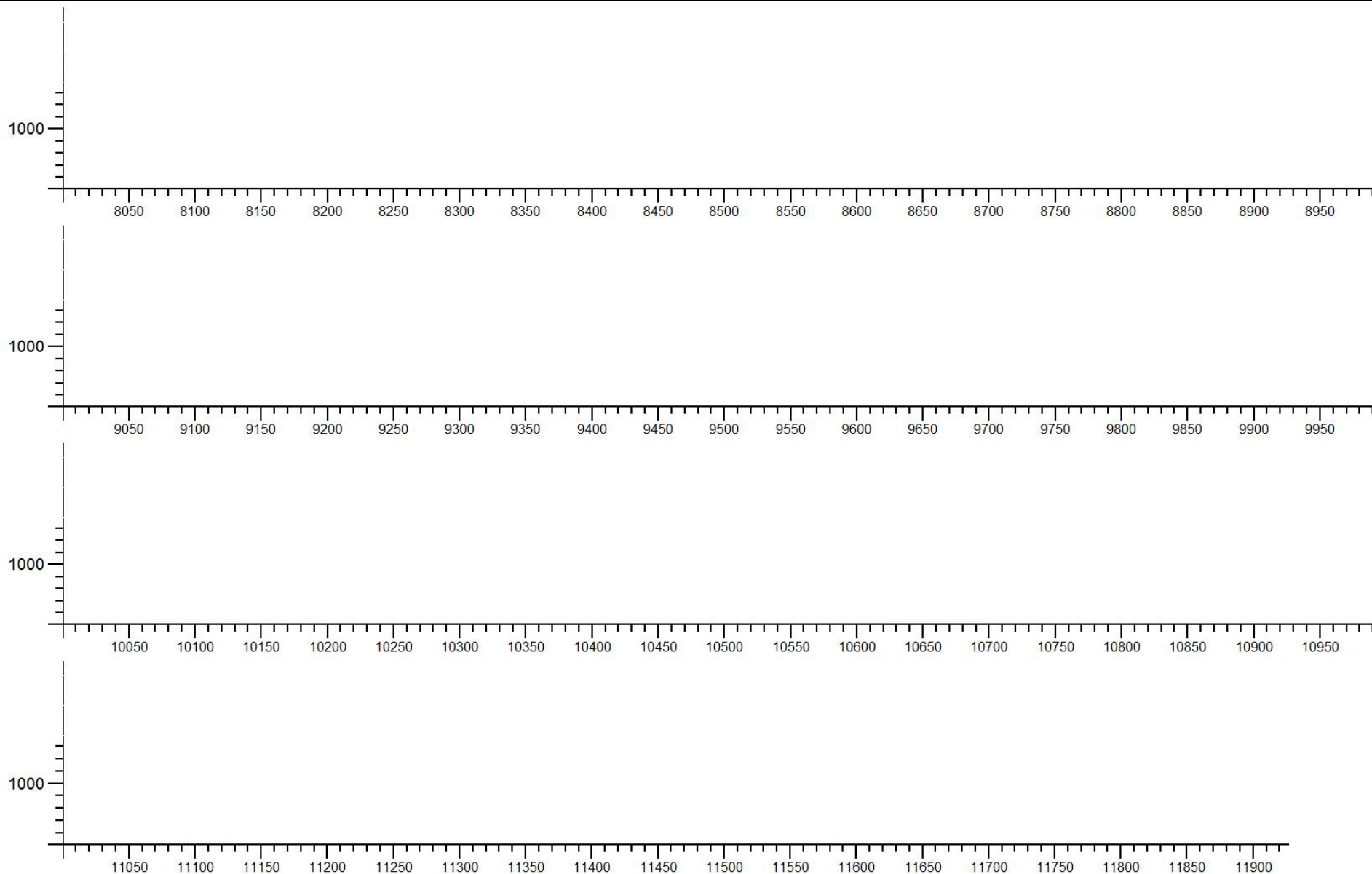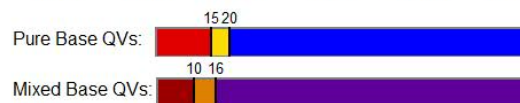

Supplement: Supplemental Information 1 — Chromatograms of: (1) recombined sequences of the H47 GI model from a number of mutants affected in recombination functions, and (2) recombined sequences of the pUYFRT model. [file peerj-05-3293-s001.zip › raw material/46-pUYFRT_recTA_in1_FA.pdf]

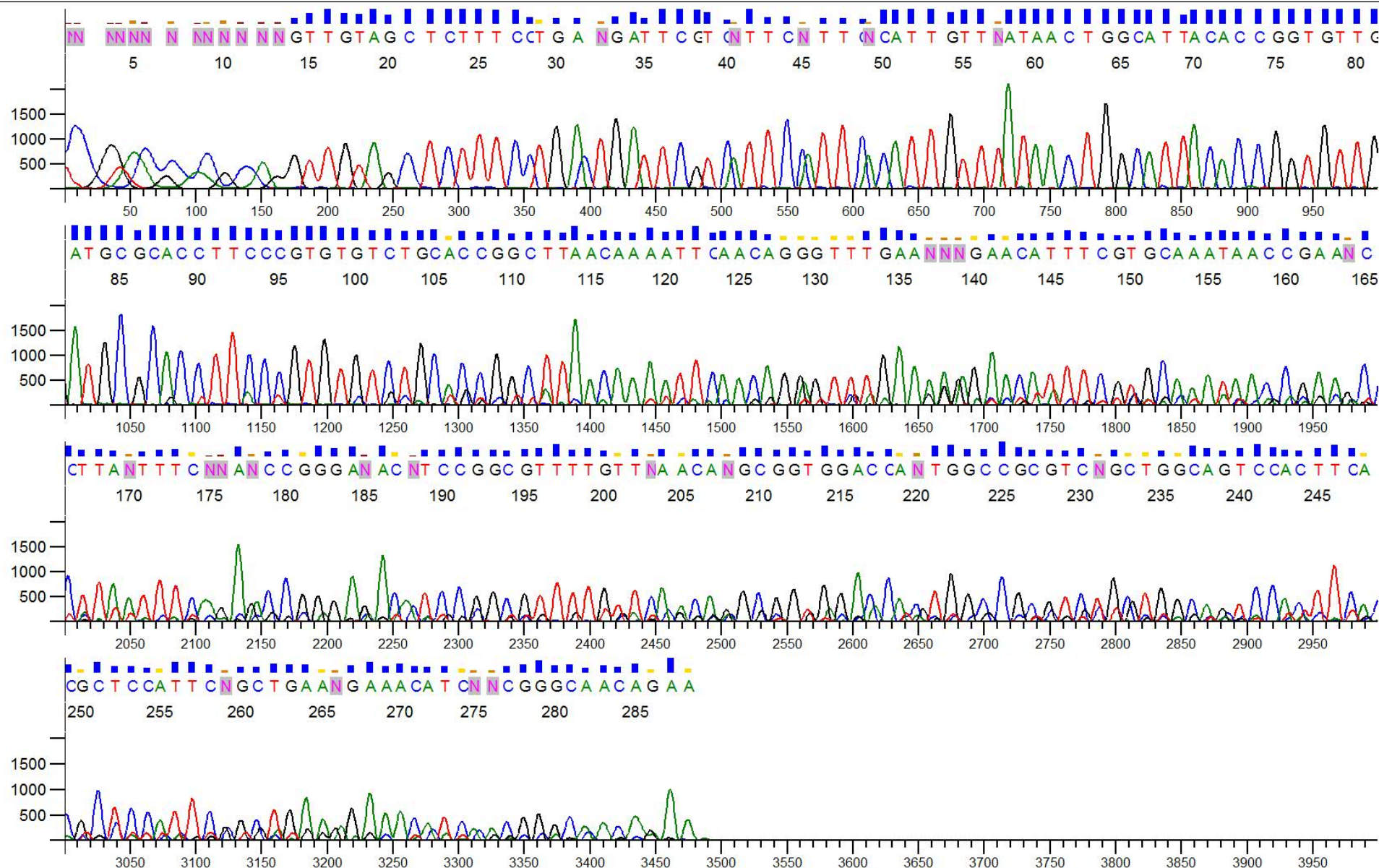

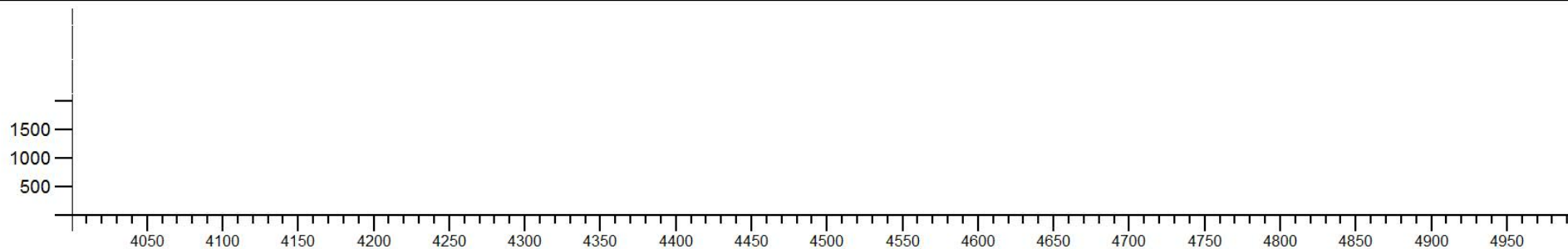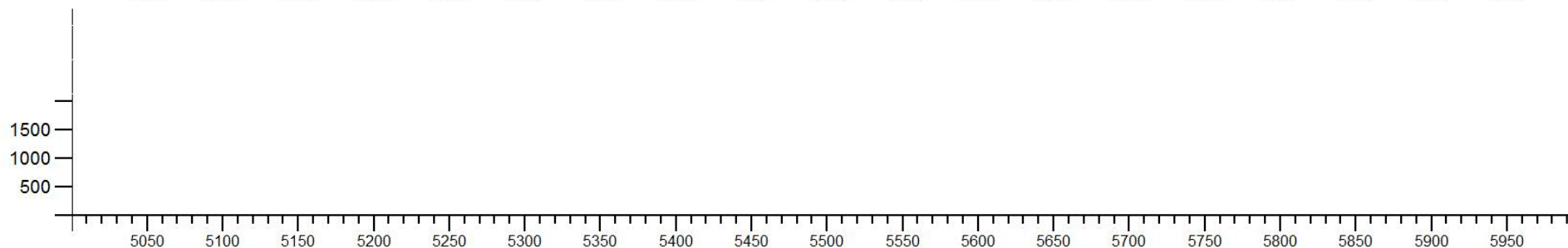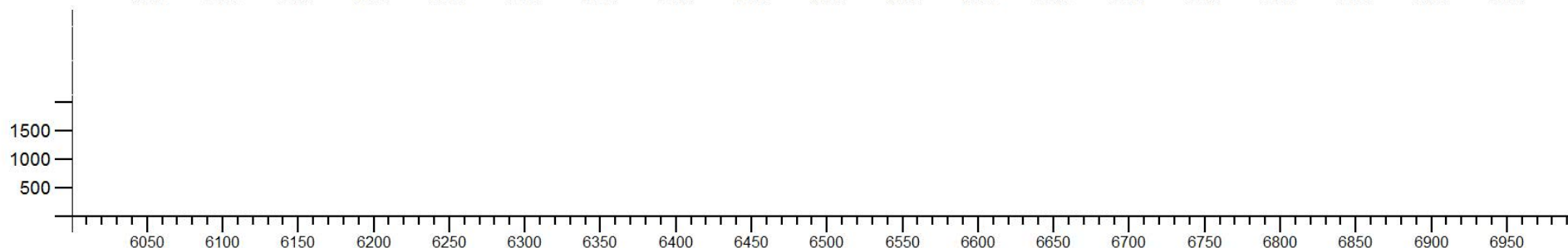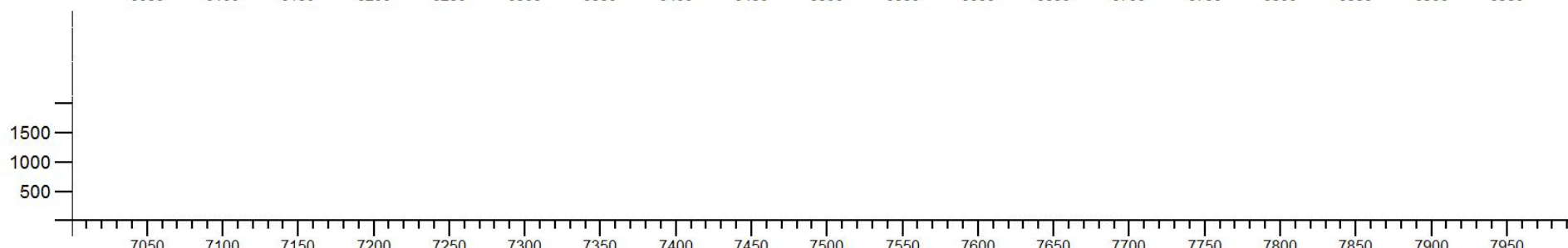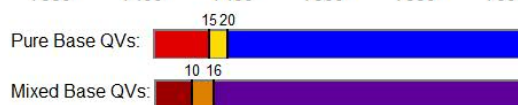

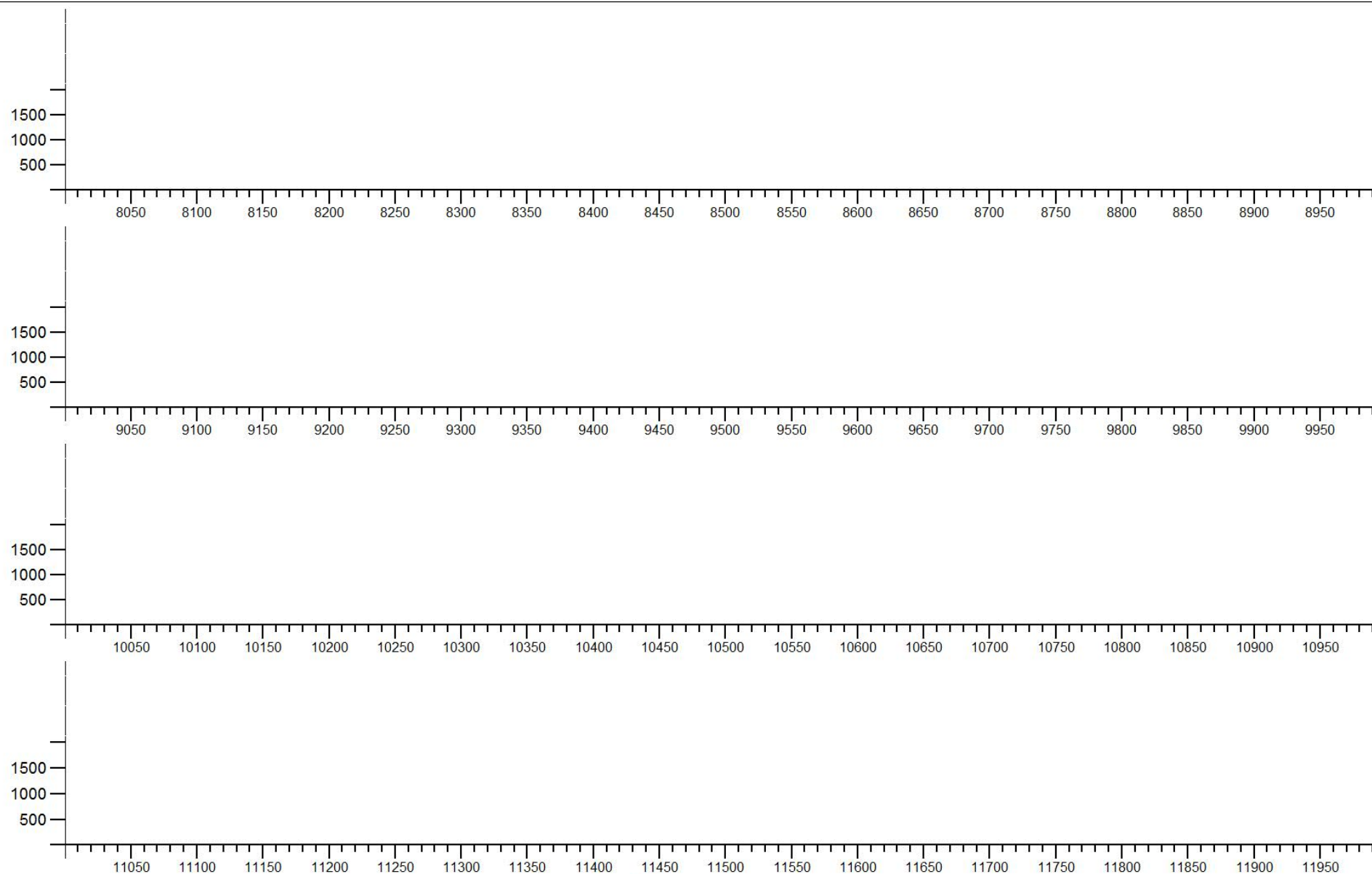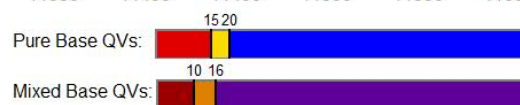

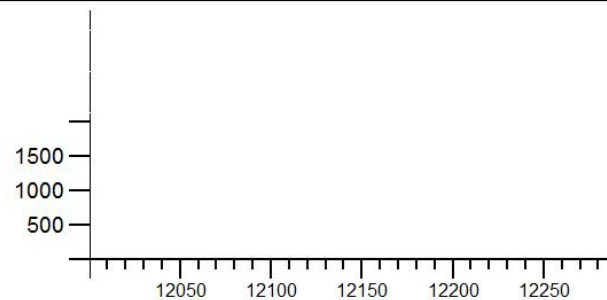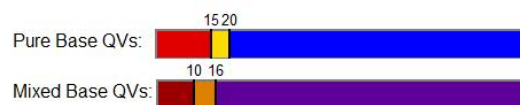

Supplement: Supplemental Information 1 — Chromatograms of: (1) recombined sequences of the H47 GI model from a number of mutants affected in recombination functions, and (2) recombined sequences of the pUYFRT model. [file peerj-05-3293-s001.zip › raw material/47-intD_out1_FA.pdf]

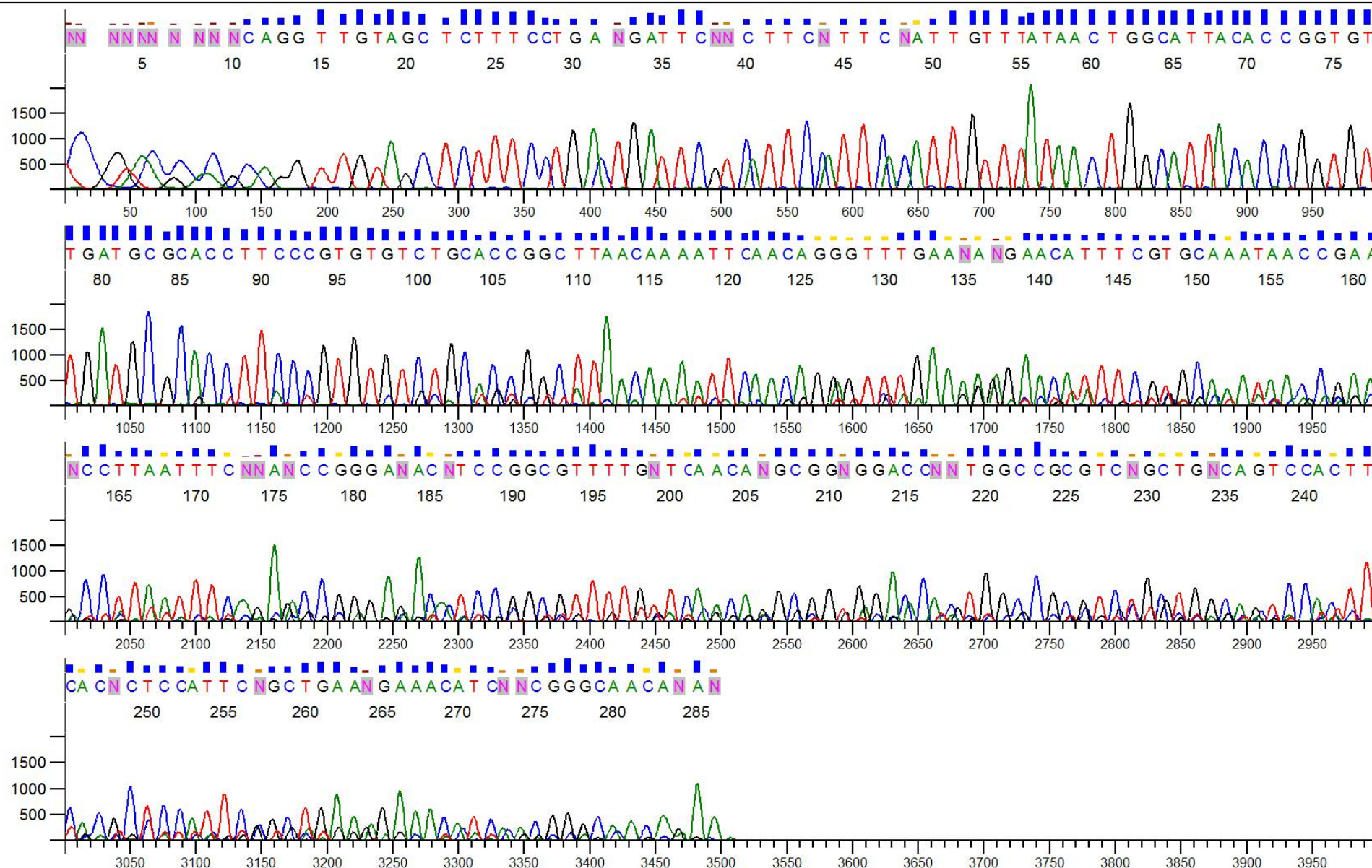

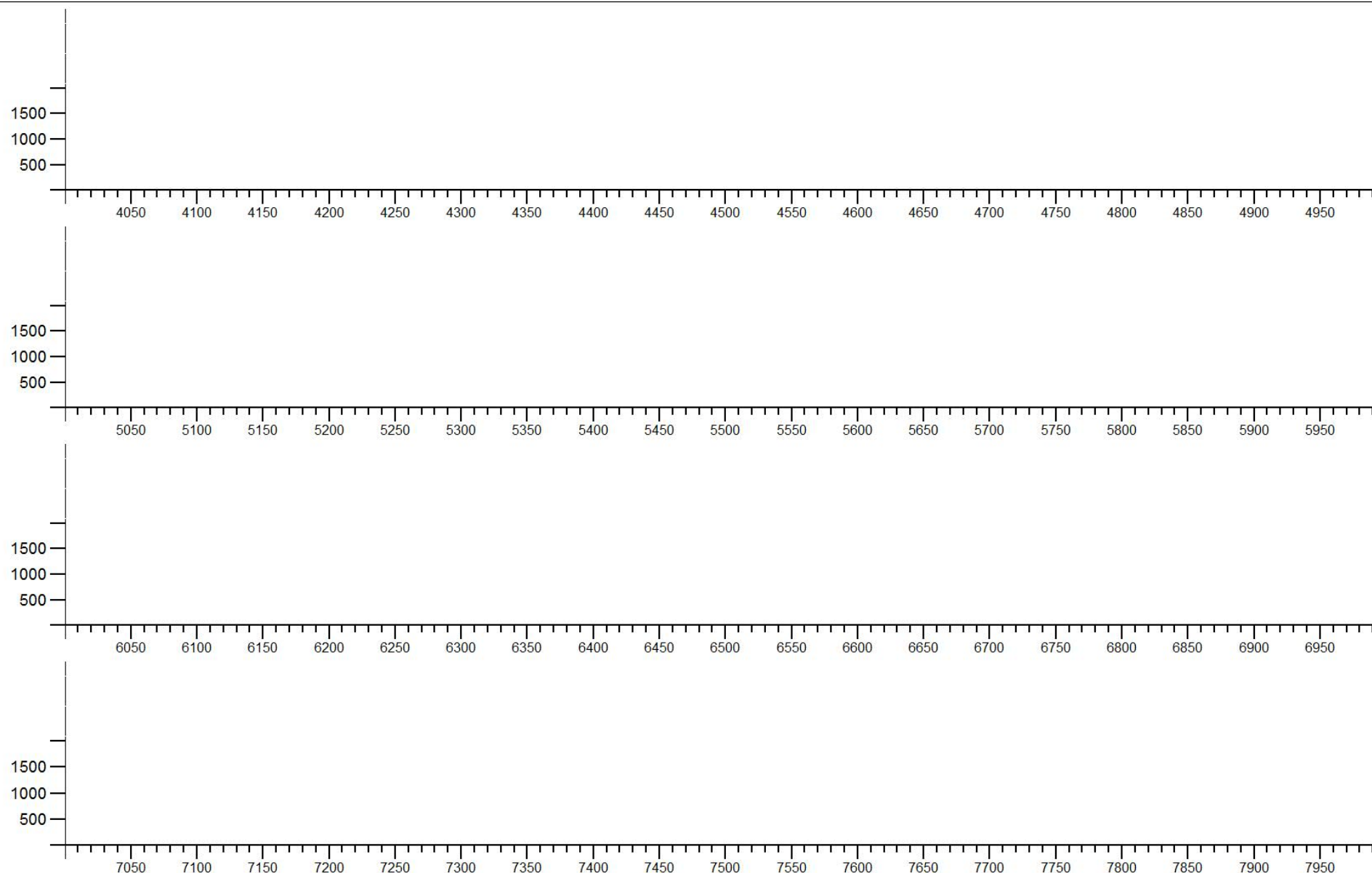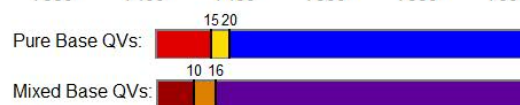

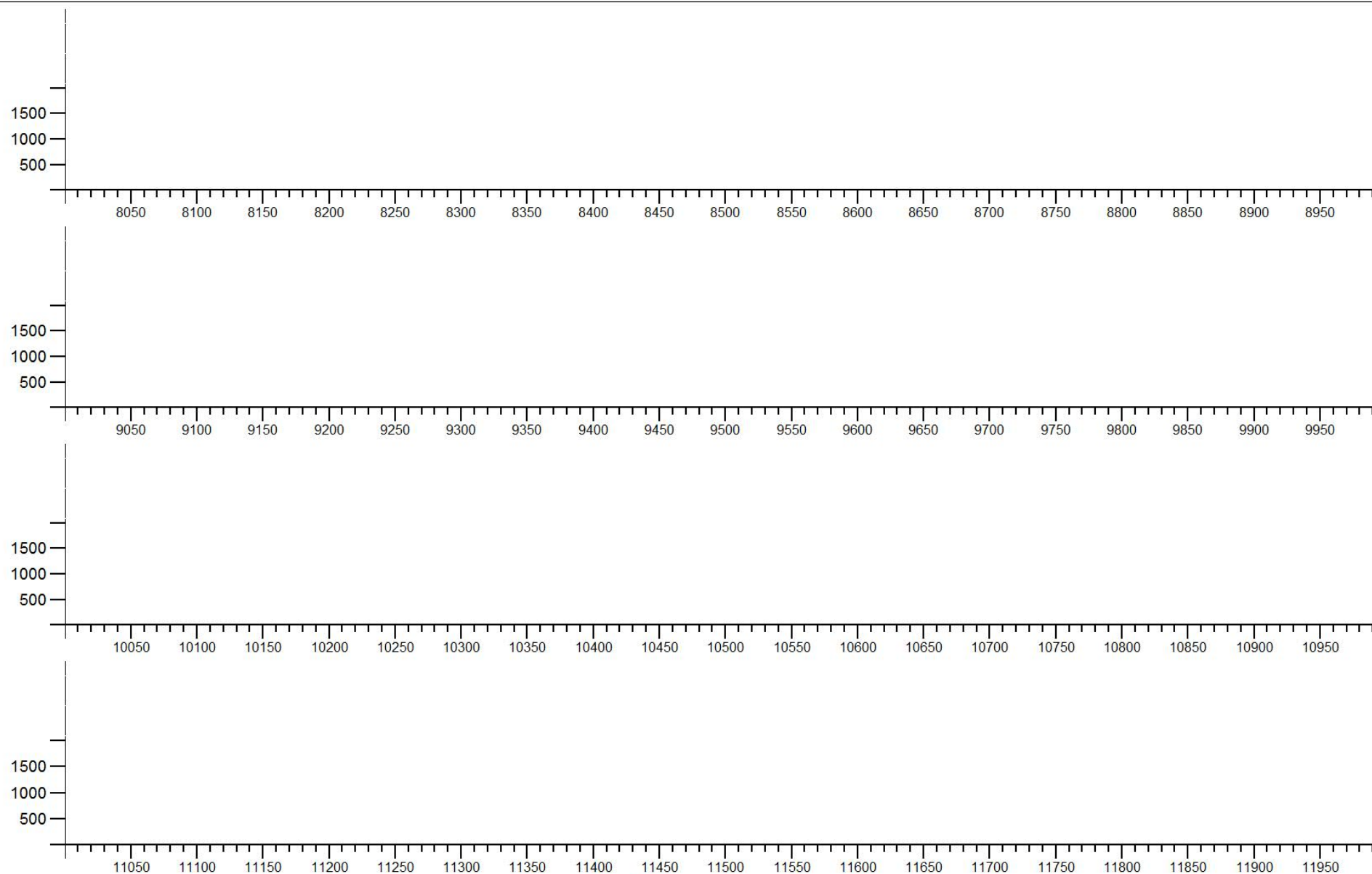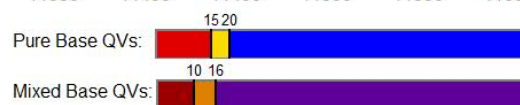

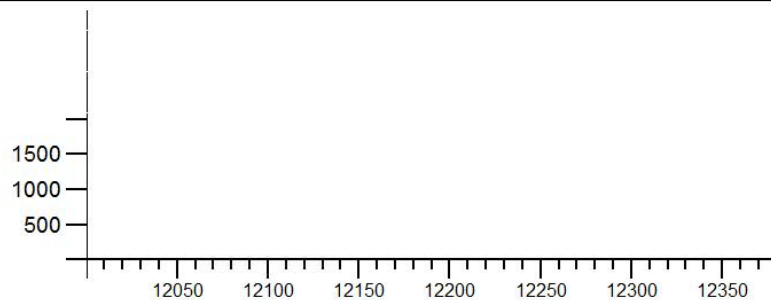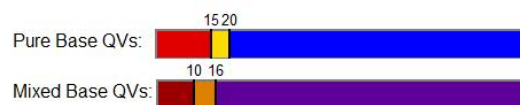

Supplement: Supplemental Information 1 — Chromatograms of: (1) recombined sequences of the H47 GI model from a number of mutants affected in recombination functions, and (2) recombined sequences of the pUYFRT model. [file peerj-05-3293-s001.zip › raw material/47-intF_out1_FA.pdf]

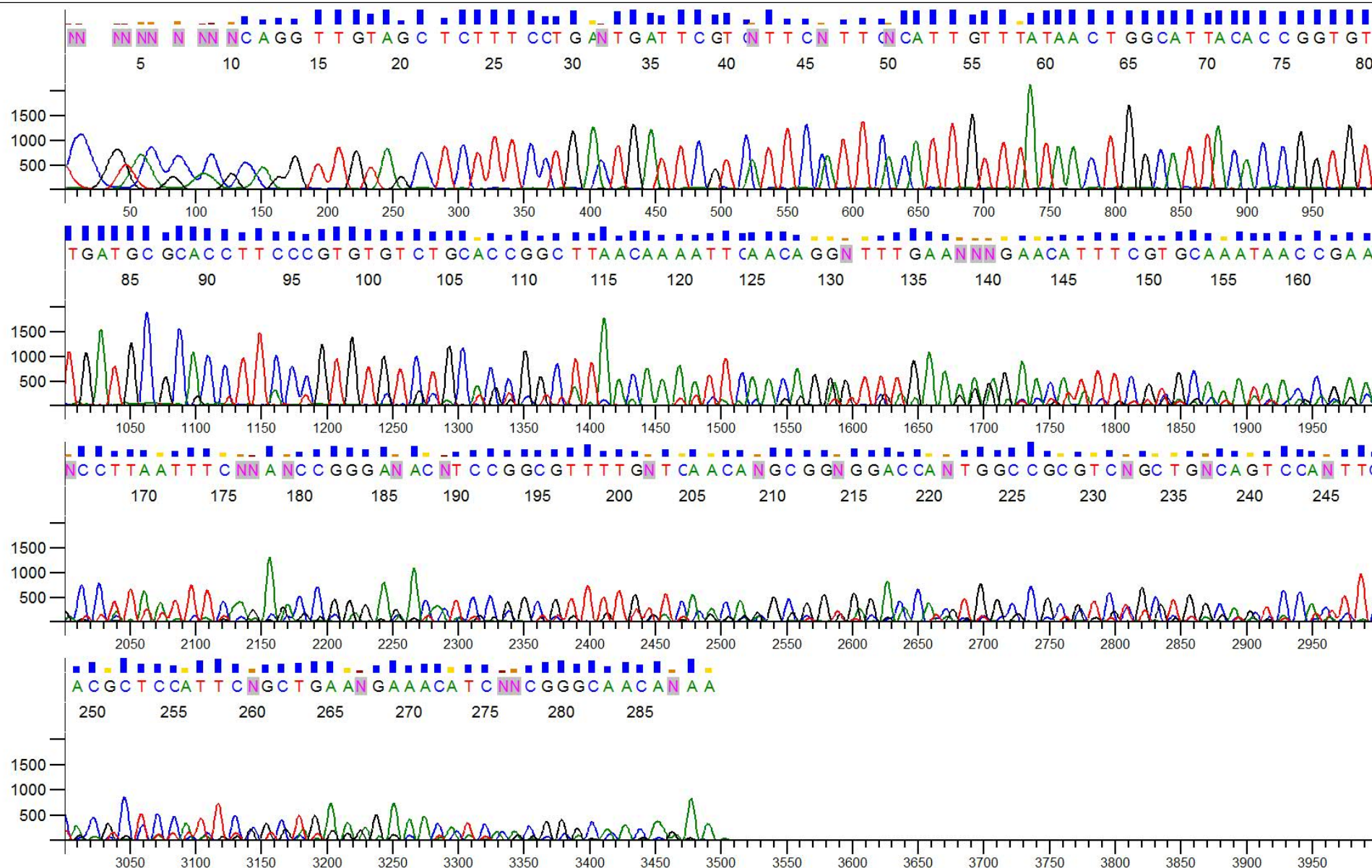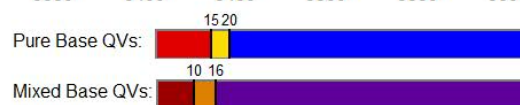

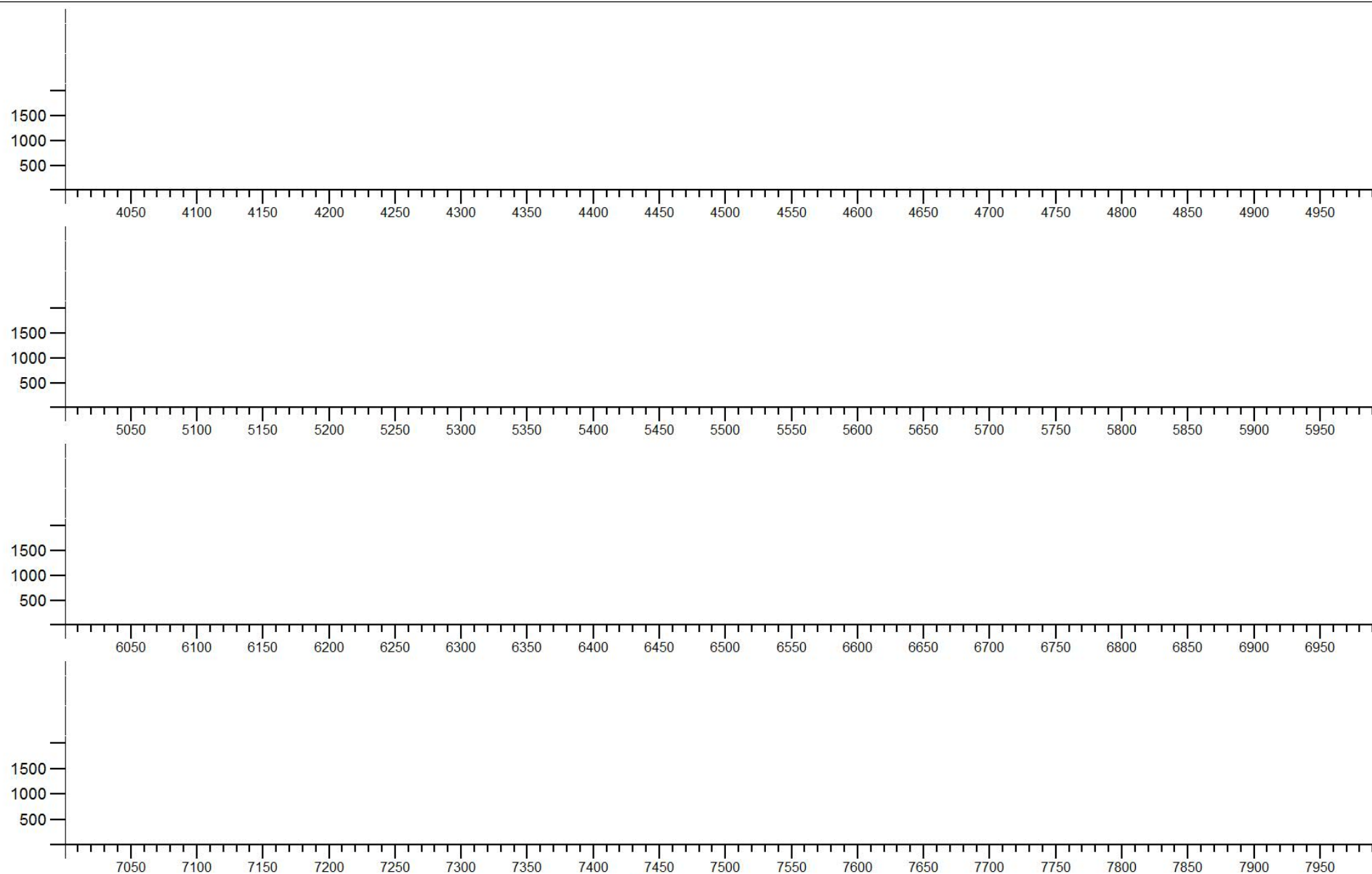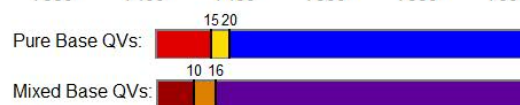

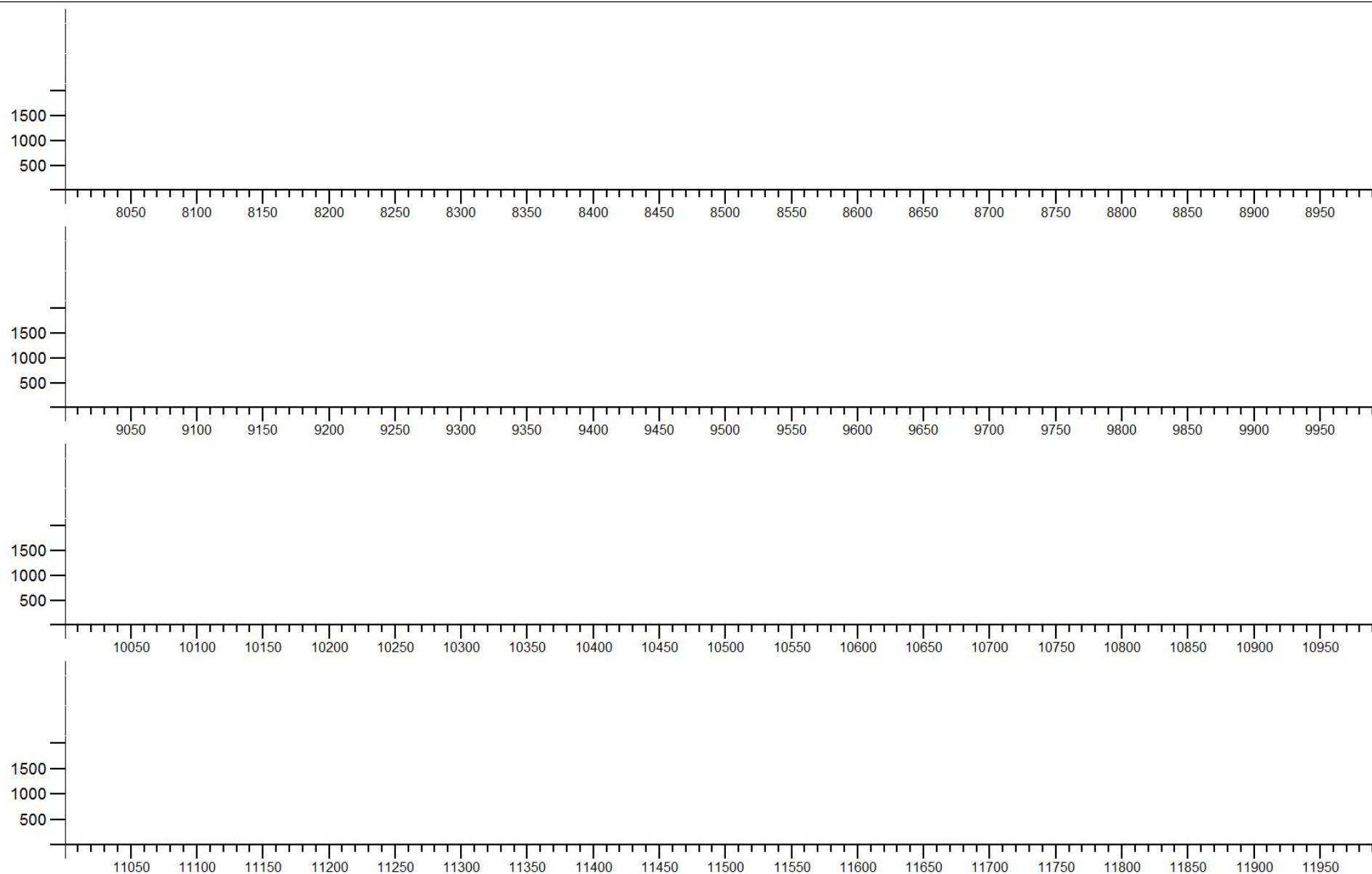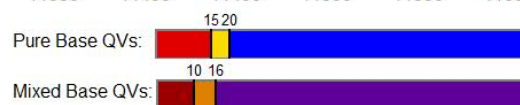

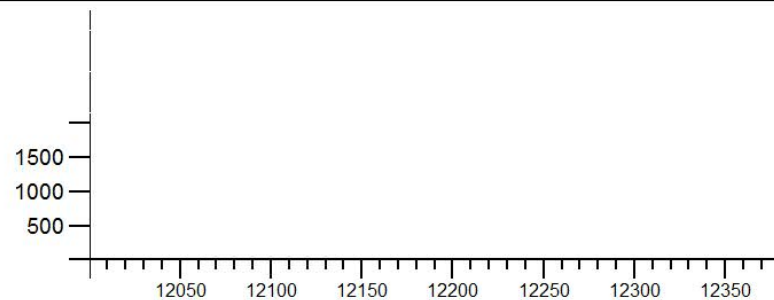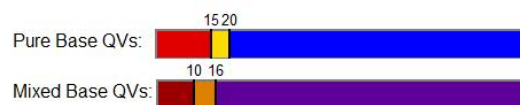

Supplement: Supplemental Information 1 — Chromatograms of: (1) recombined sequences of the H47 GI model from a number of mutants affected in recombination functions, and (2) recombined sequences of the pUYFRT model. [file peerj-05-3293-s001.zip › raw material/48-intS_out1_FA.pdf]

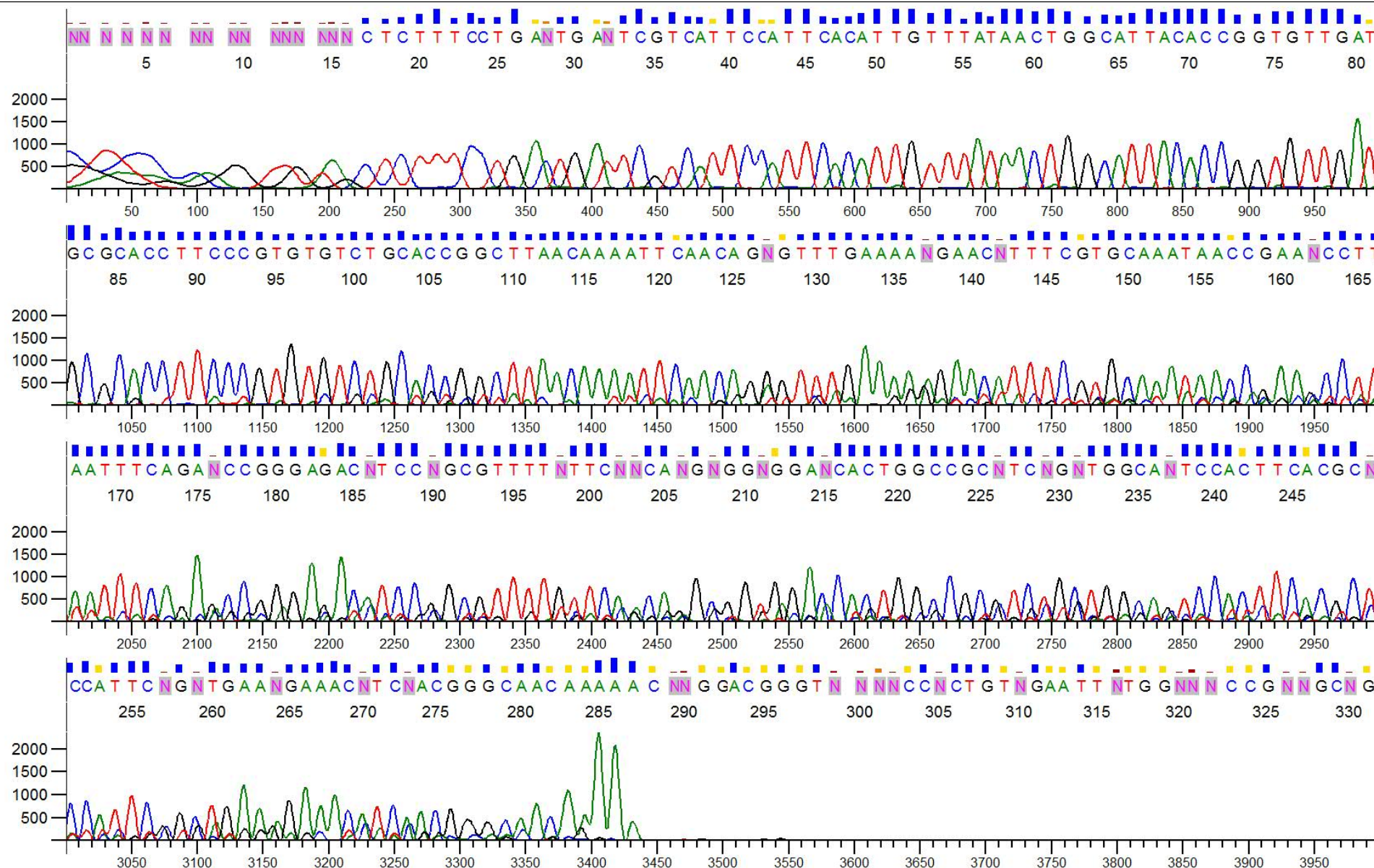

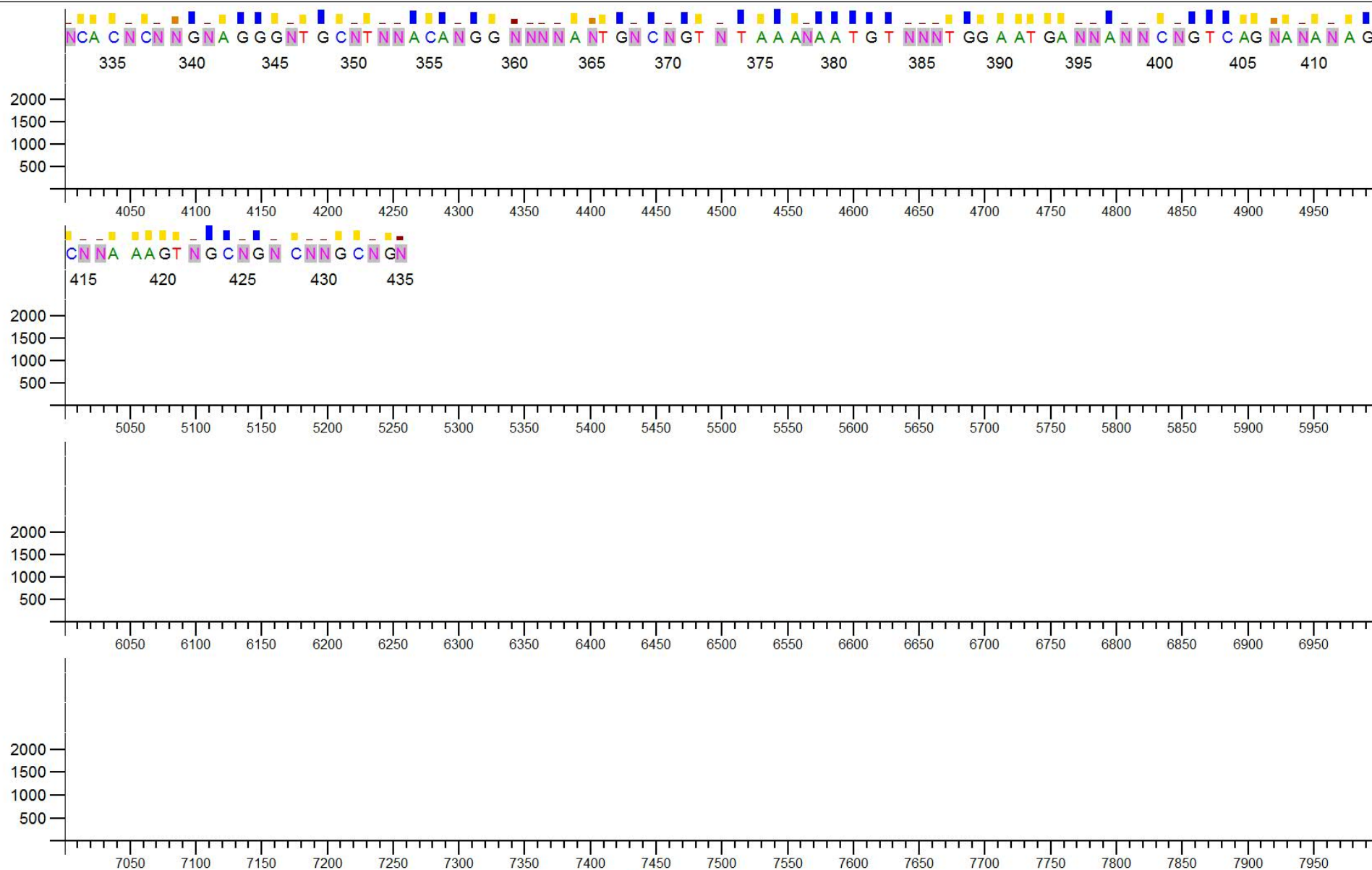

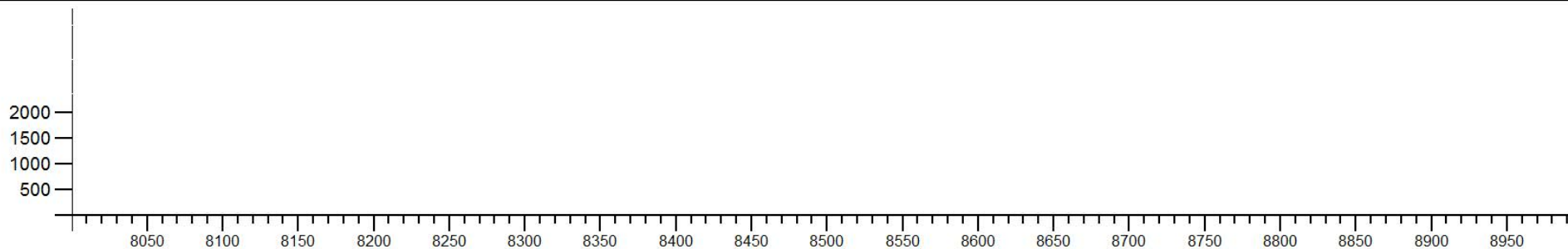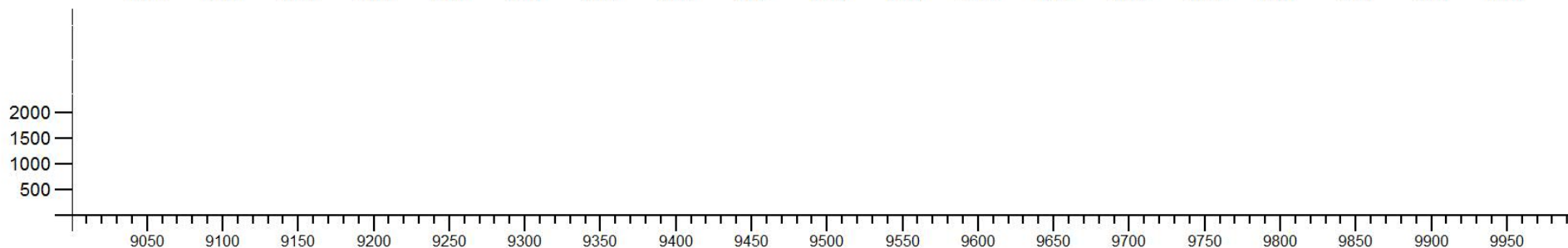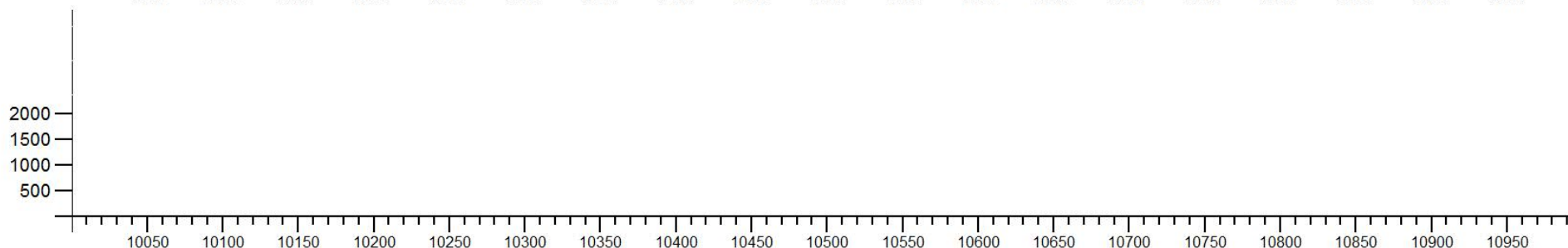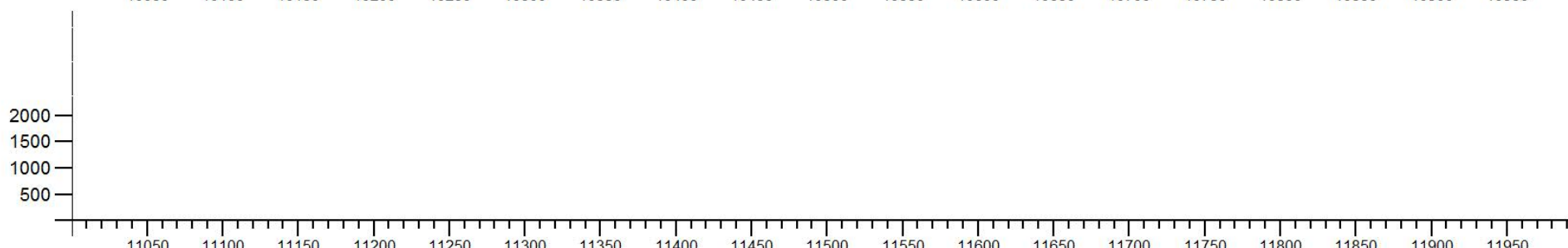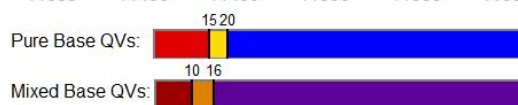

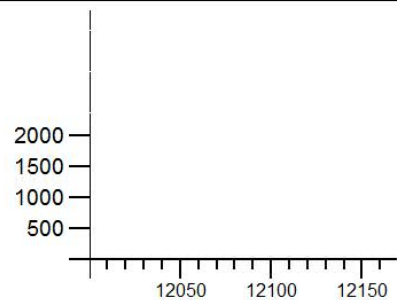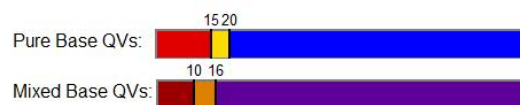

Supplement: Supplemental Information 1 — Chromatograms of: (1) recombined sequences of the H47 GI model from a number of mutants affected in recombination functions, and (2) recombined sequences of the pUYFRT model. [file peerj-05-3293-s001.zip › raw material/49-XerC_out1_FA.pdf]

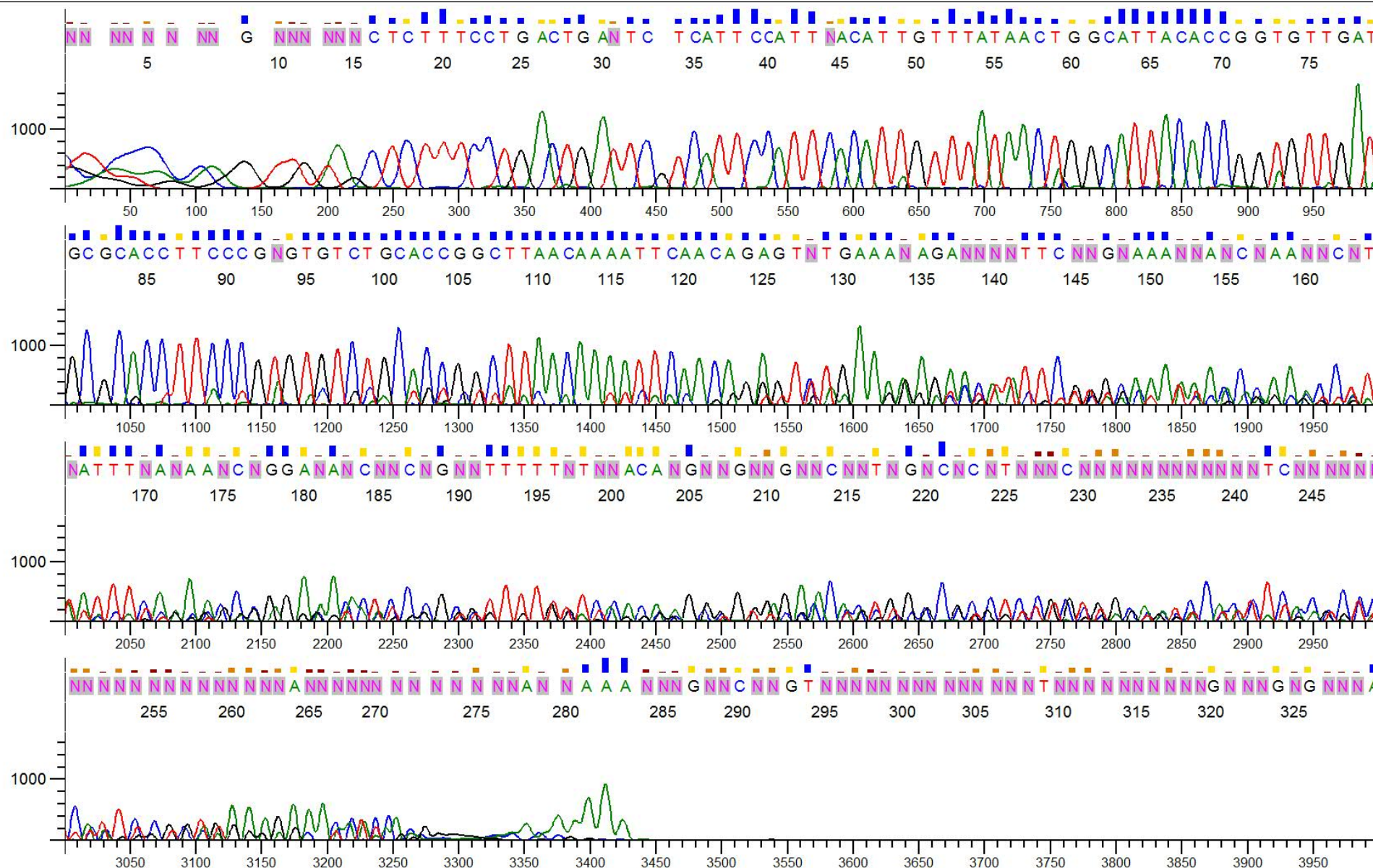

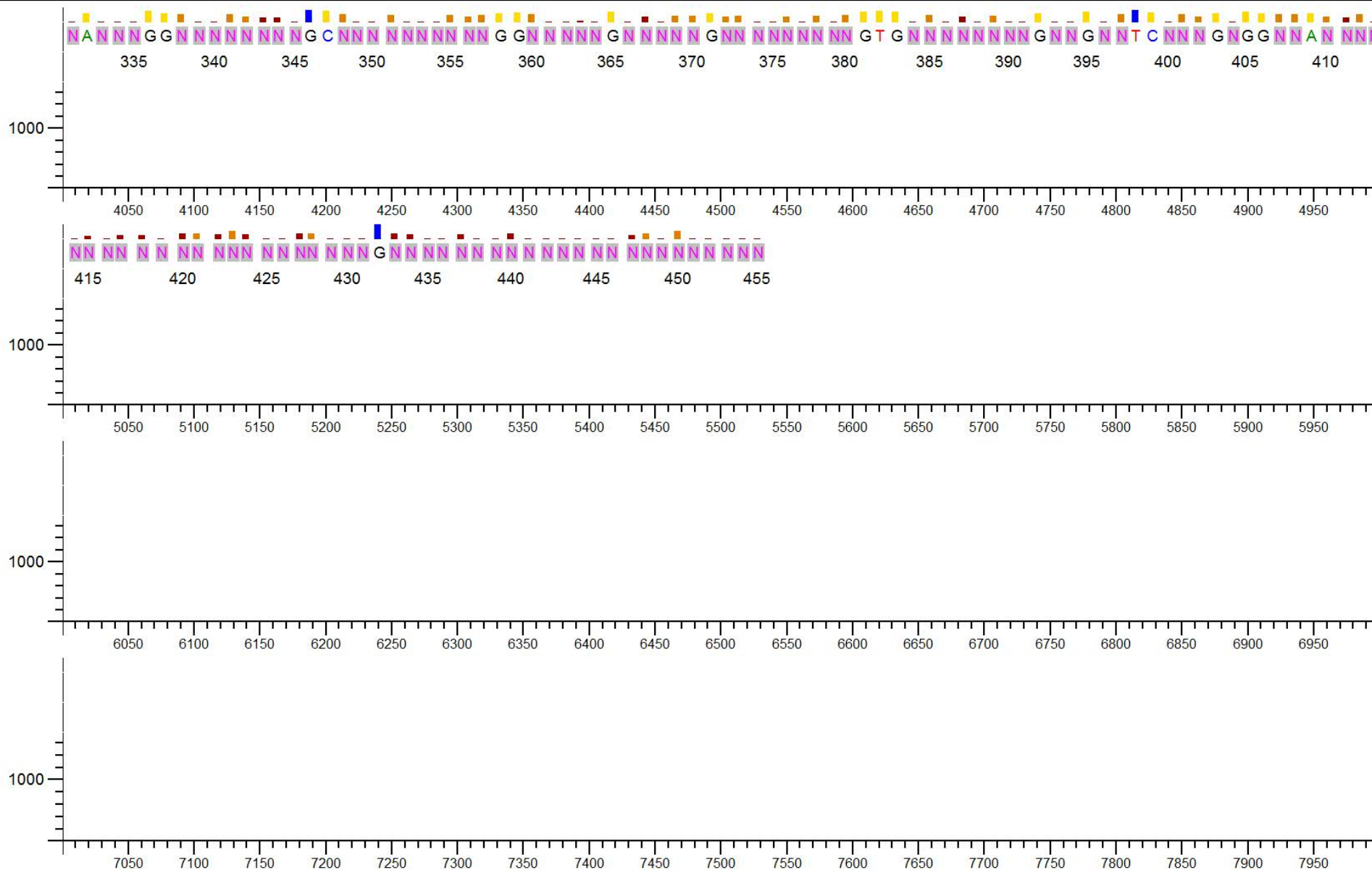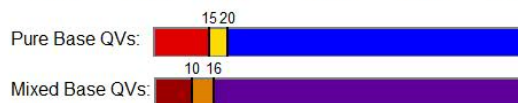

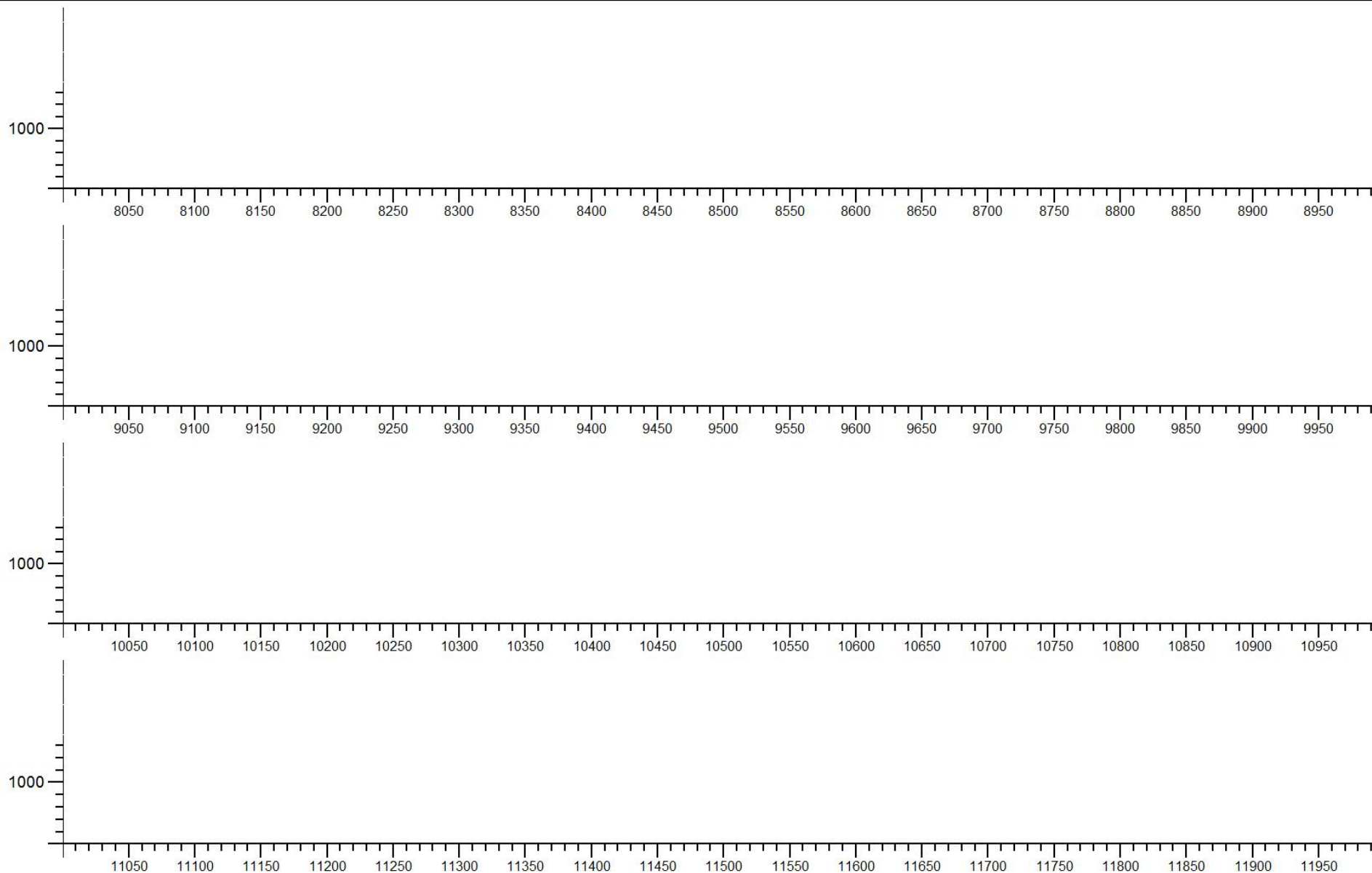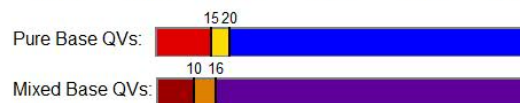

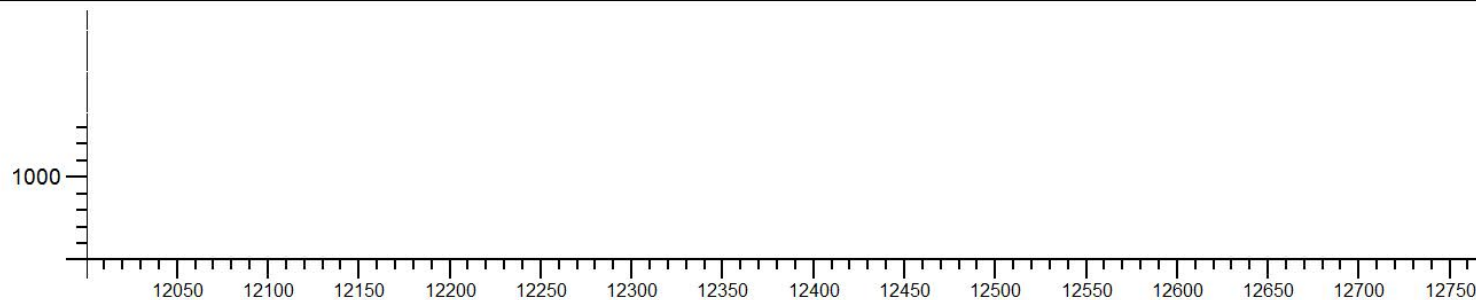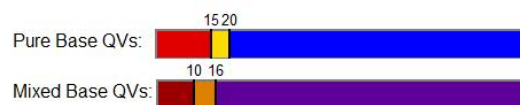

Supplement: Supplemental Information 1 — Chromatograms of: (1) recombined sequences of the H47 GI model from a number of mutants affected in recombination functions, and (2) recombined sequences of the pUYFRT model. [file peerj-05-3293-s001.zip › raw material/4-SbcC_out1_FA.pdf]

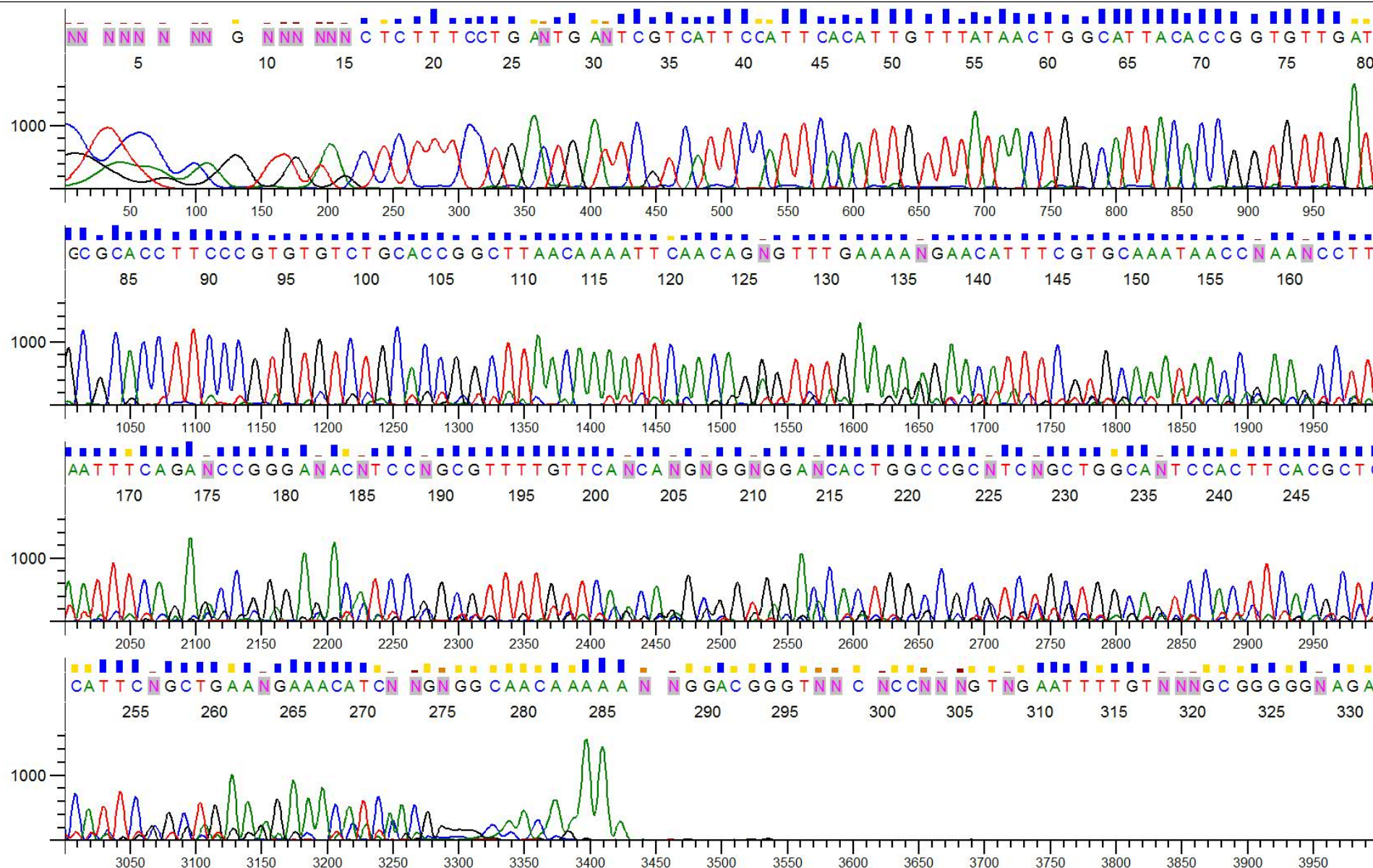

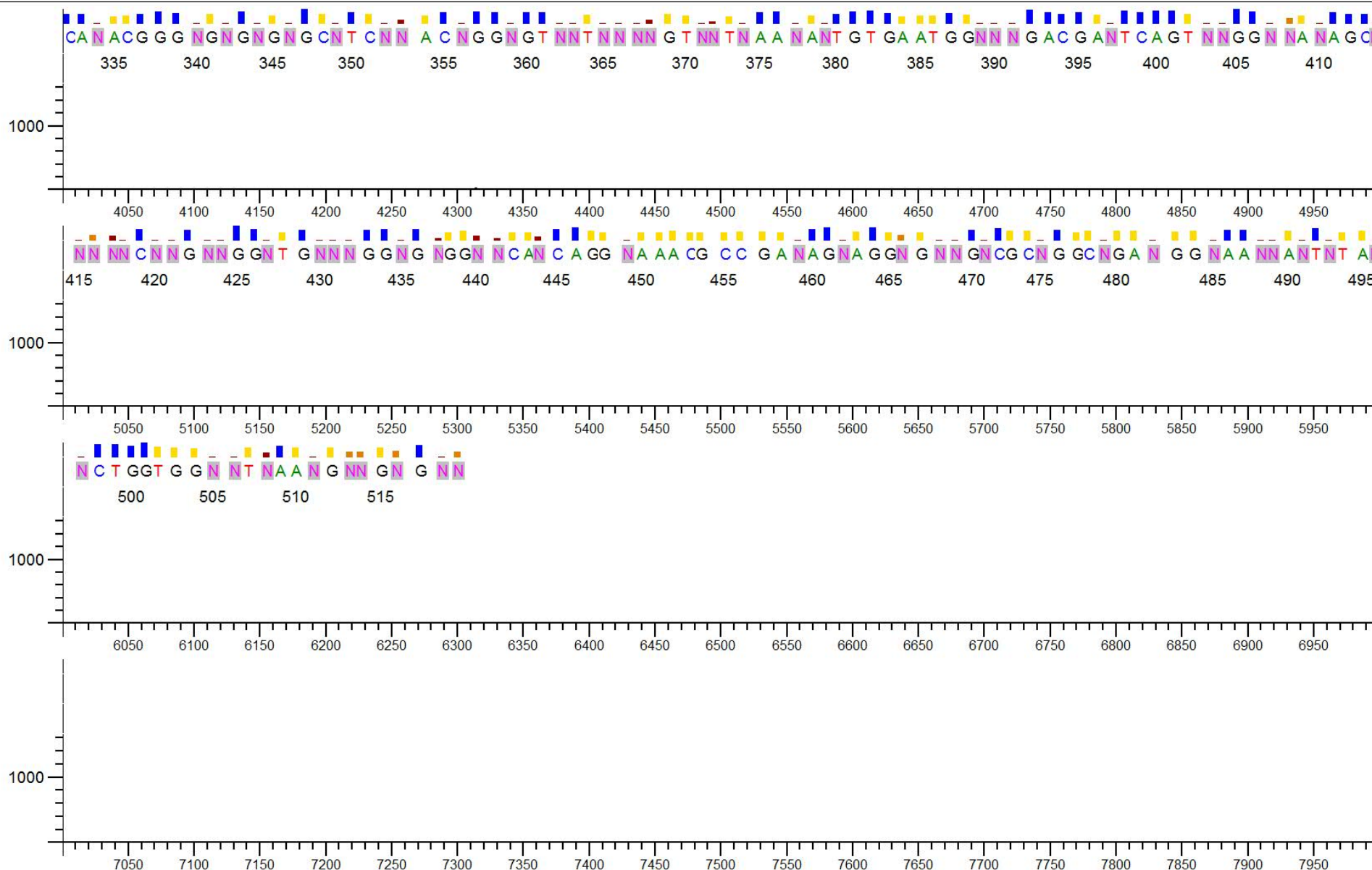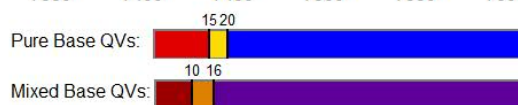

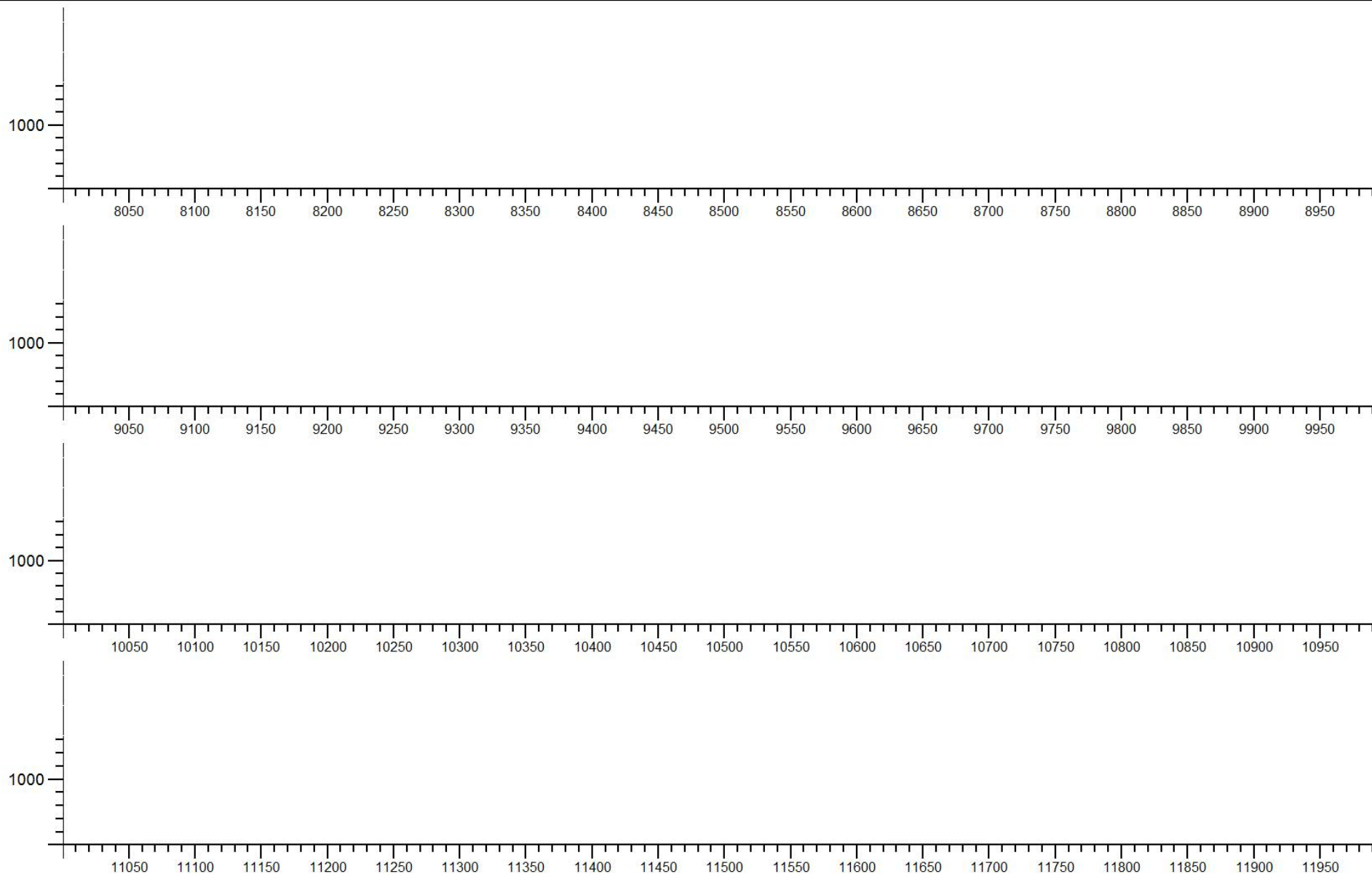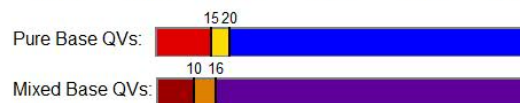

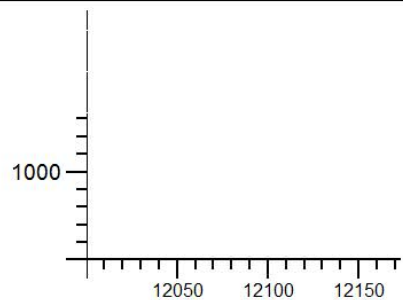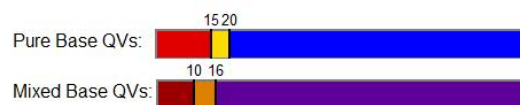

Supplement: Supplemental Information 1 — Chromatograms of: (1) recombined sequences of the H47 GI model from a number of mutants affected in recombination functions, and (2) recombined sequences of the pUYFRT model. [file peerj-05-3293-s001.zip › raw material/50-XerD_out1_FA.pdf]

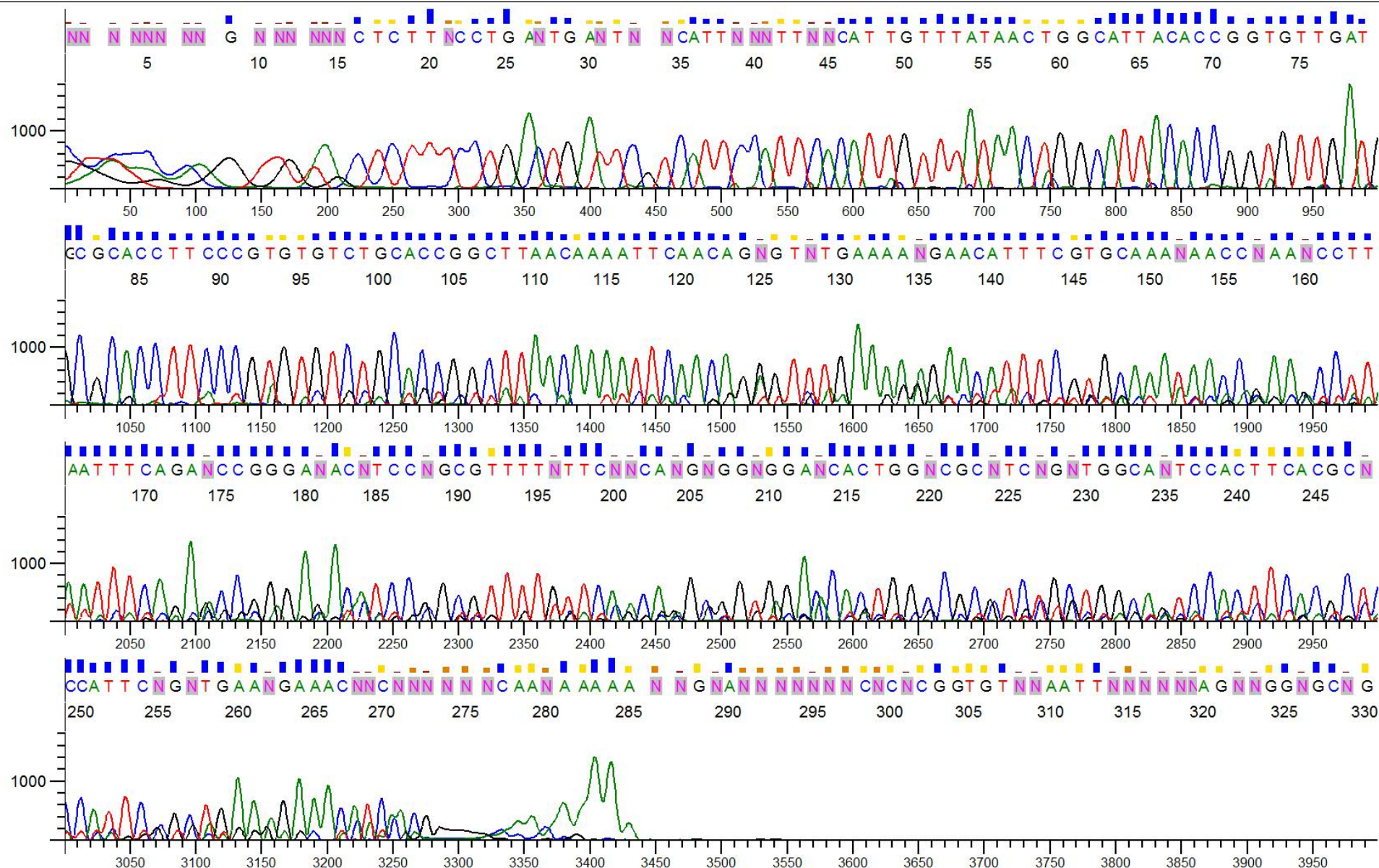

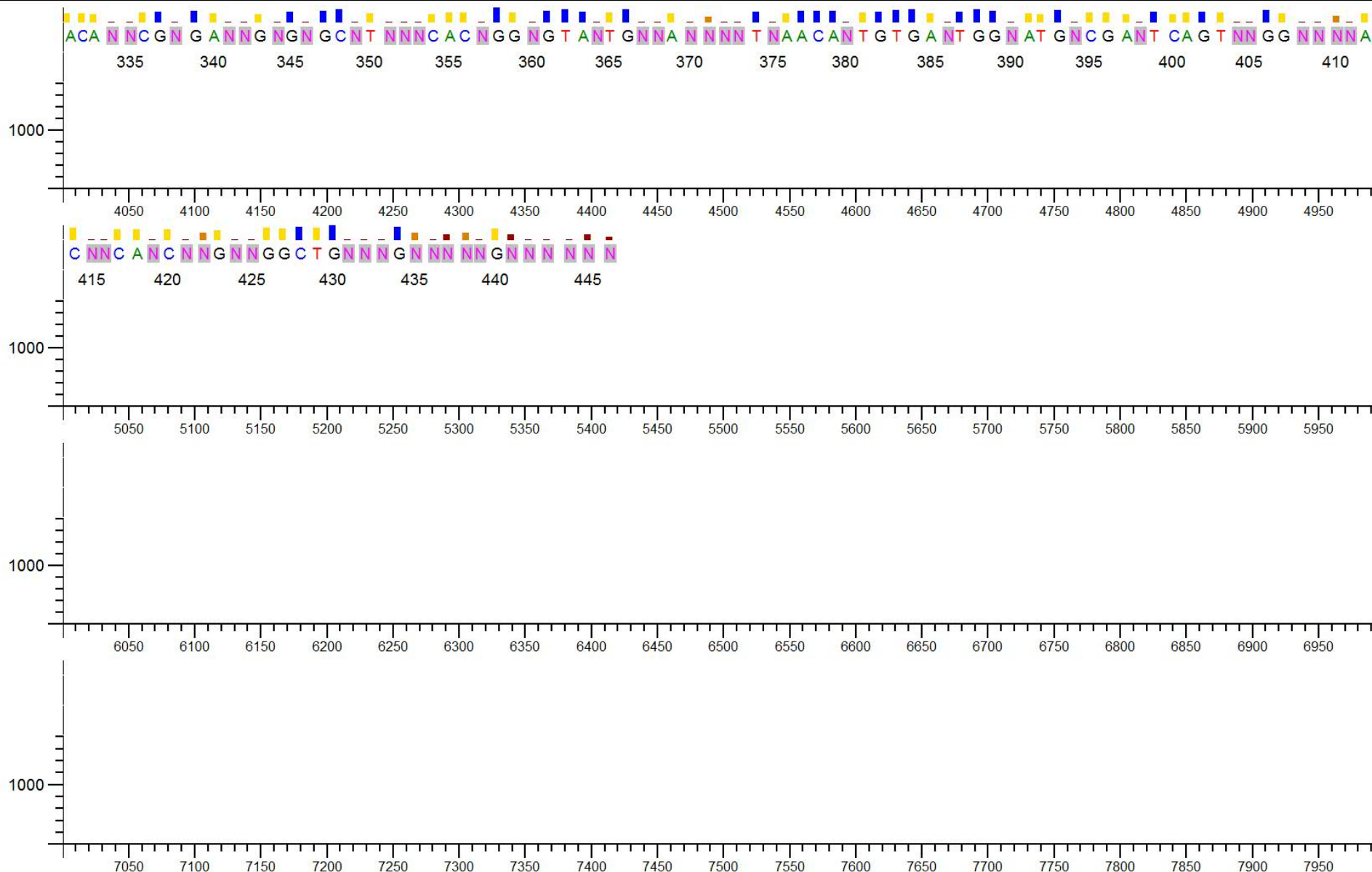

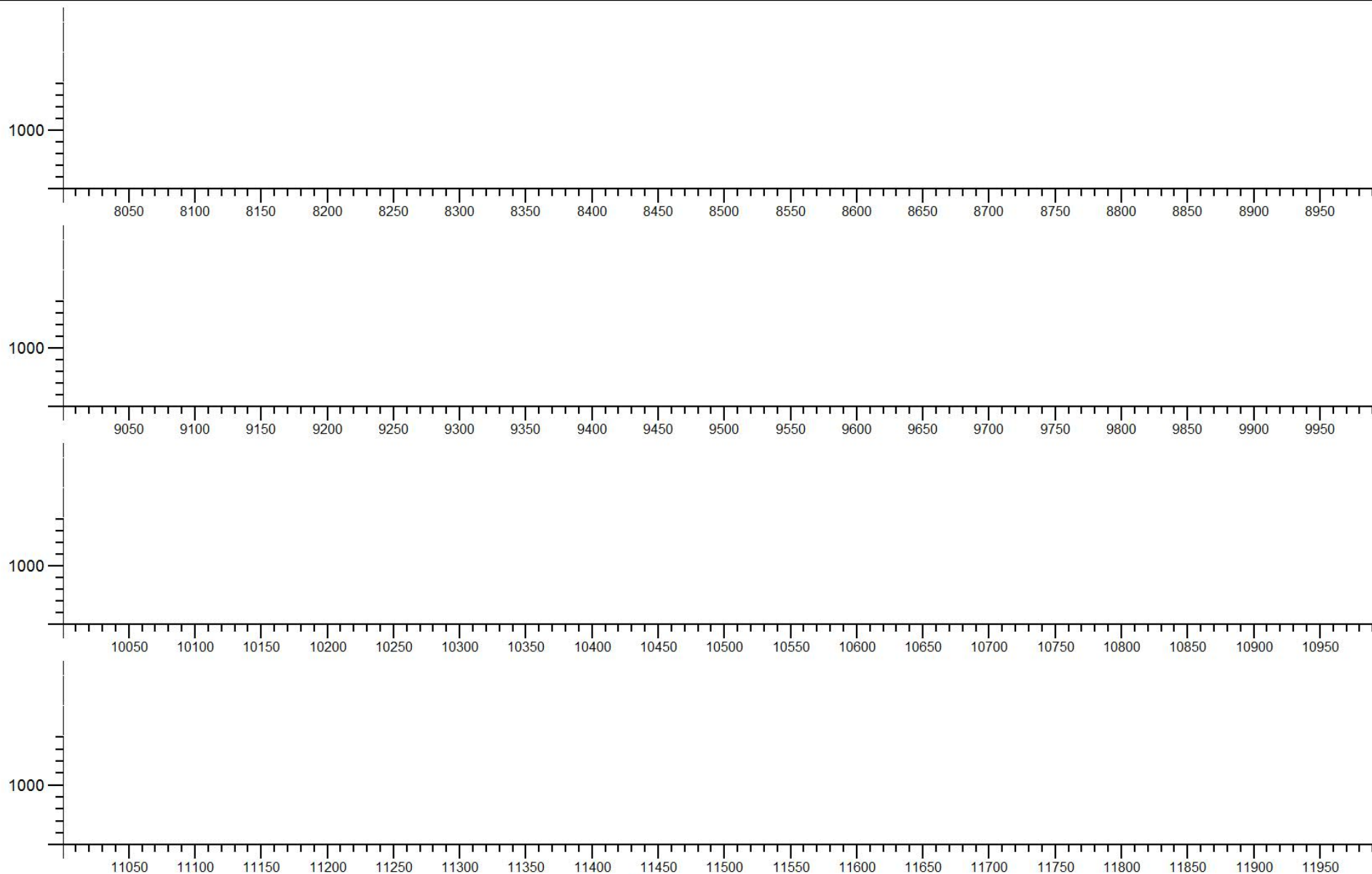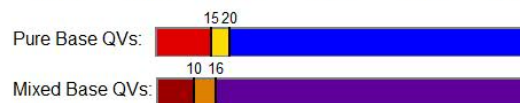

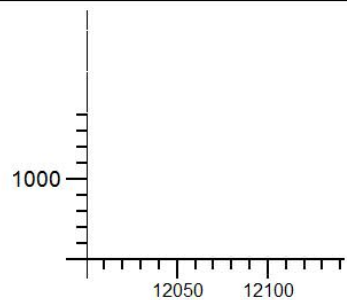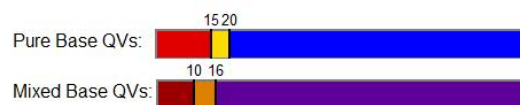

Supplement: Supplemental Information 1 — Chromatograms of: (1) recombined sequences of the H47 GI model from a number of mutants affected in recombination functions, and (2) recombined sequences of the pUYFRT model. [file peerj-05-3293-s001.zip › raw material/51-FimB_out1_FA.pdf]

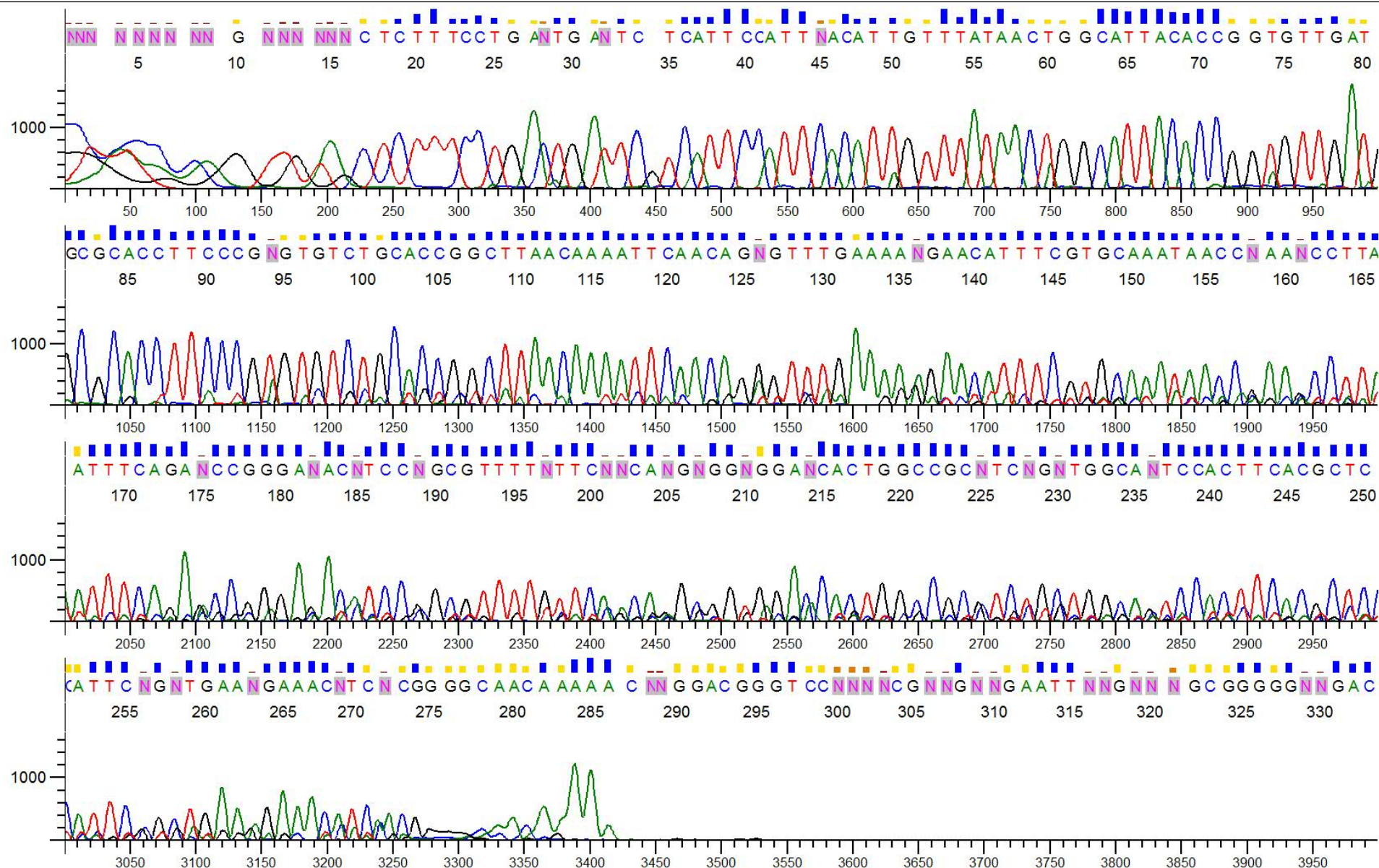

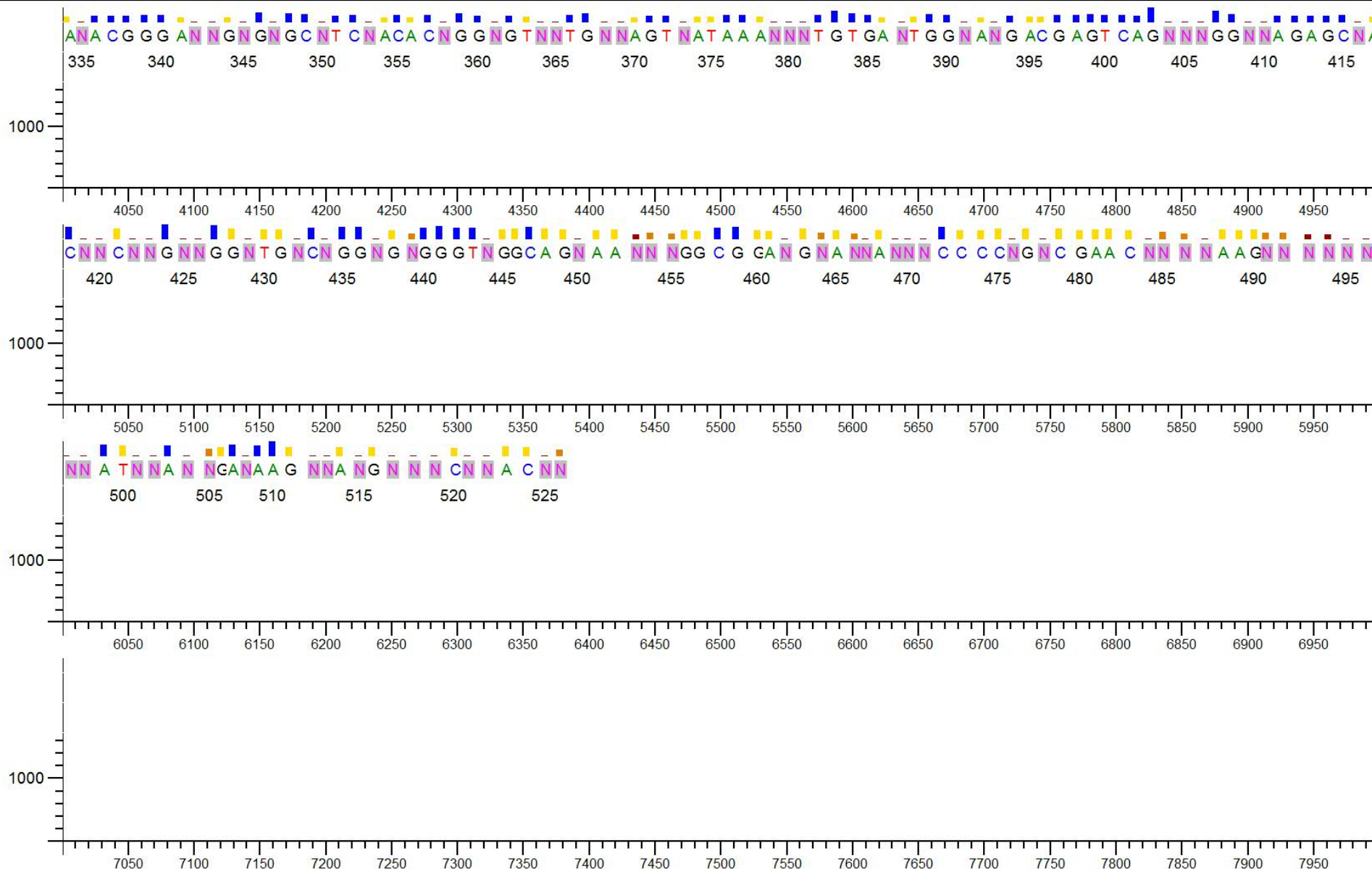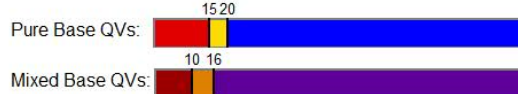

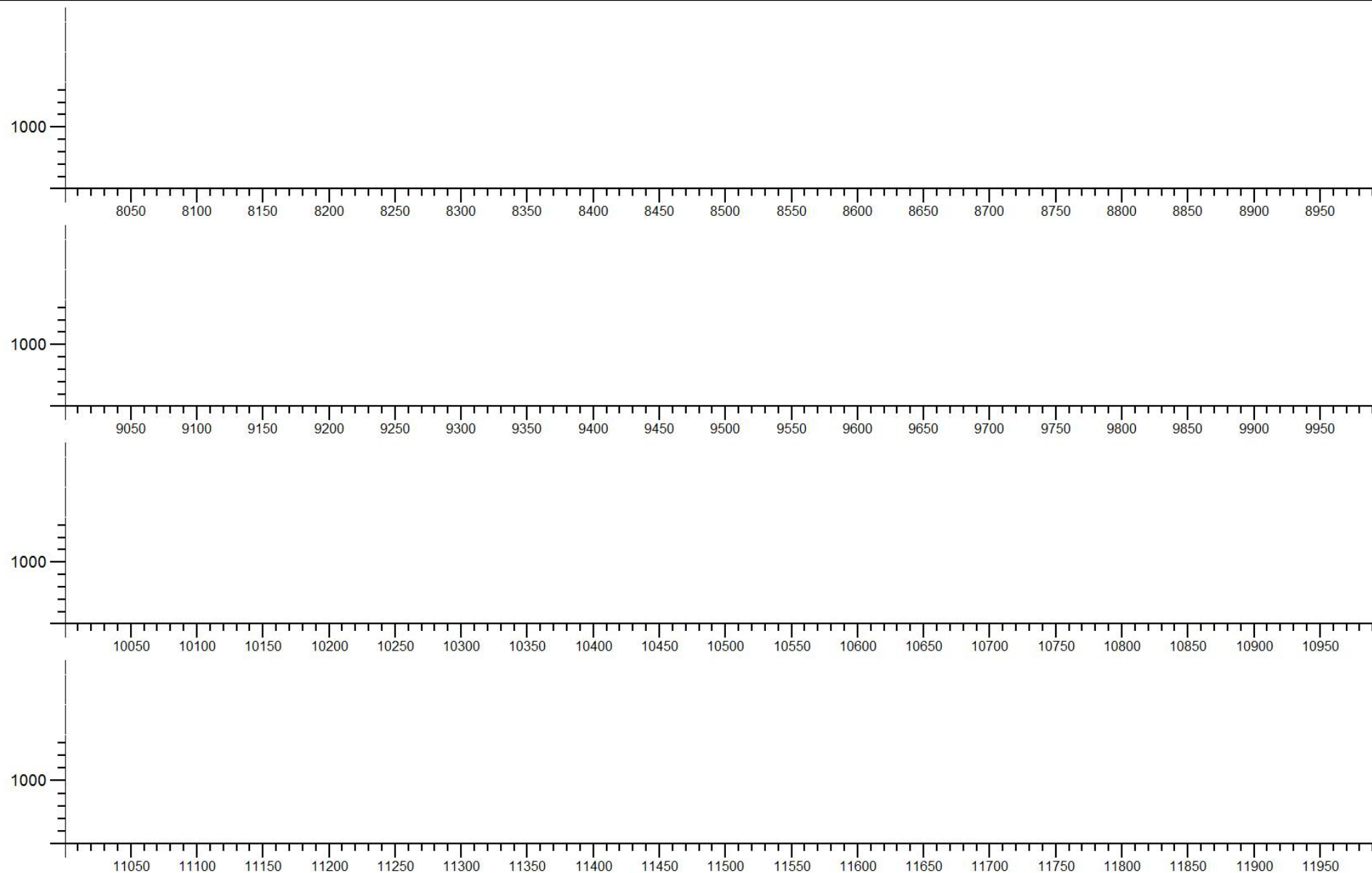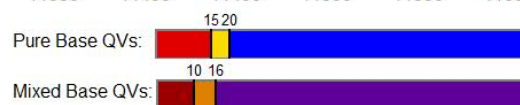

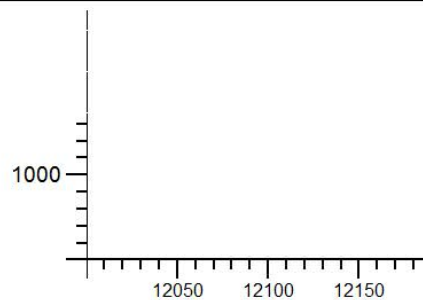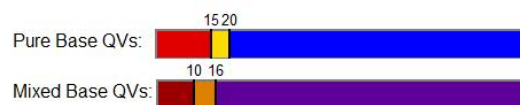

Supplement: Supplemental Information 1 — Chromatograms of: (1) recombined sequences of the H47 GI model from a number of mutants affected in recombination functions, and (2) recombined sequences of the pUYFRT model. [file peerj-05-3293-s001.zip › raw material/52-FimE_out1_FA.pdf]

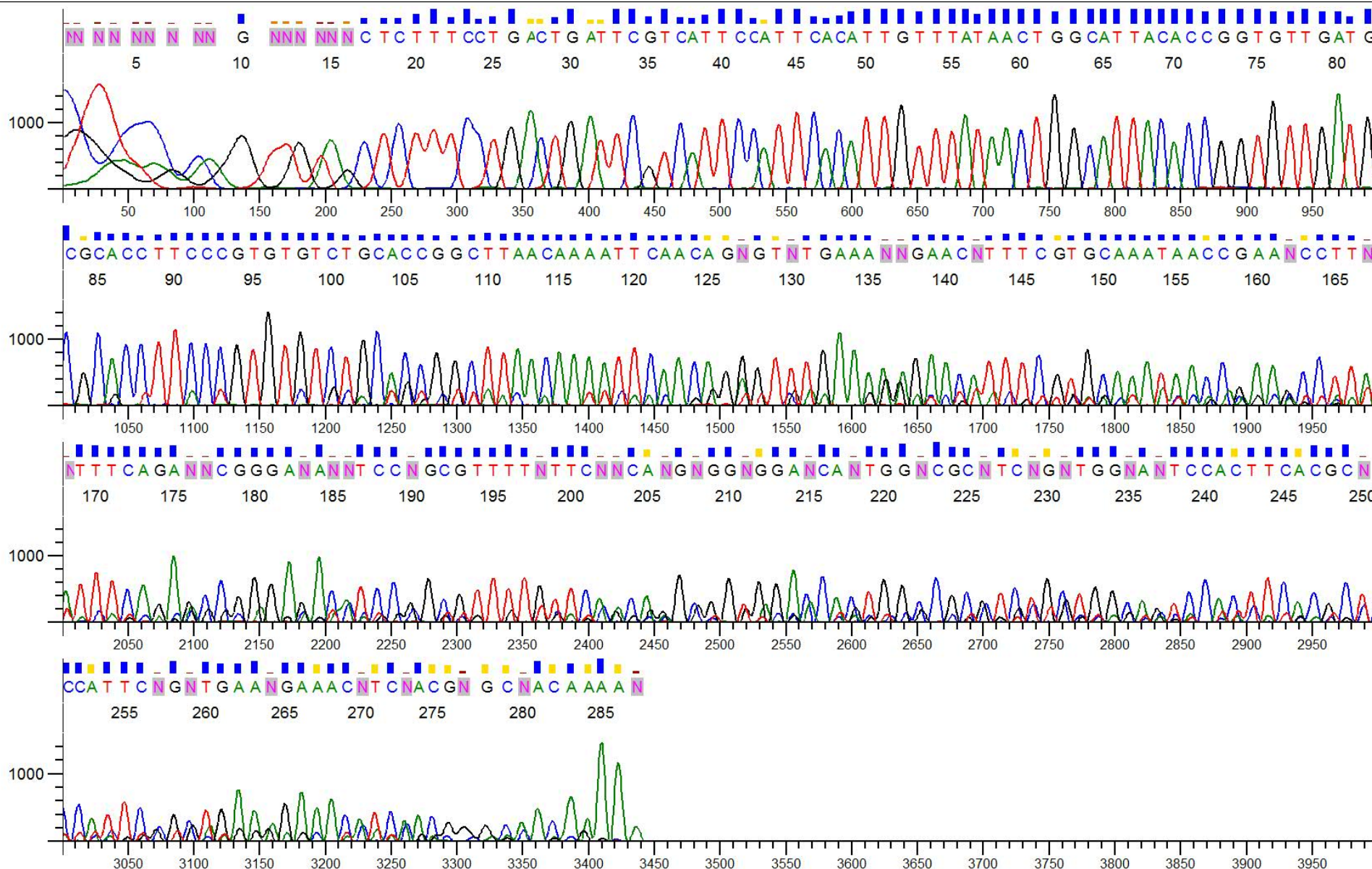

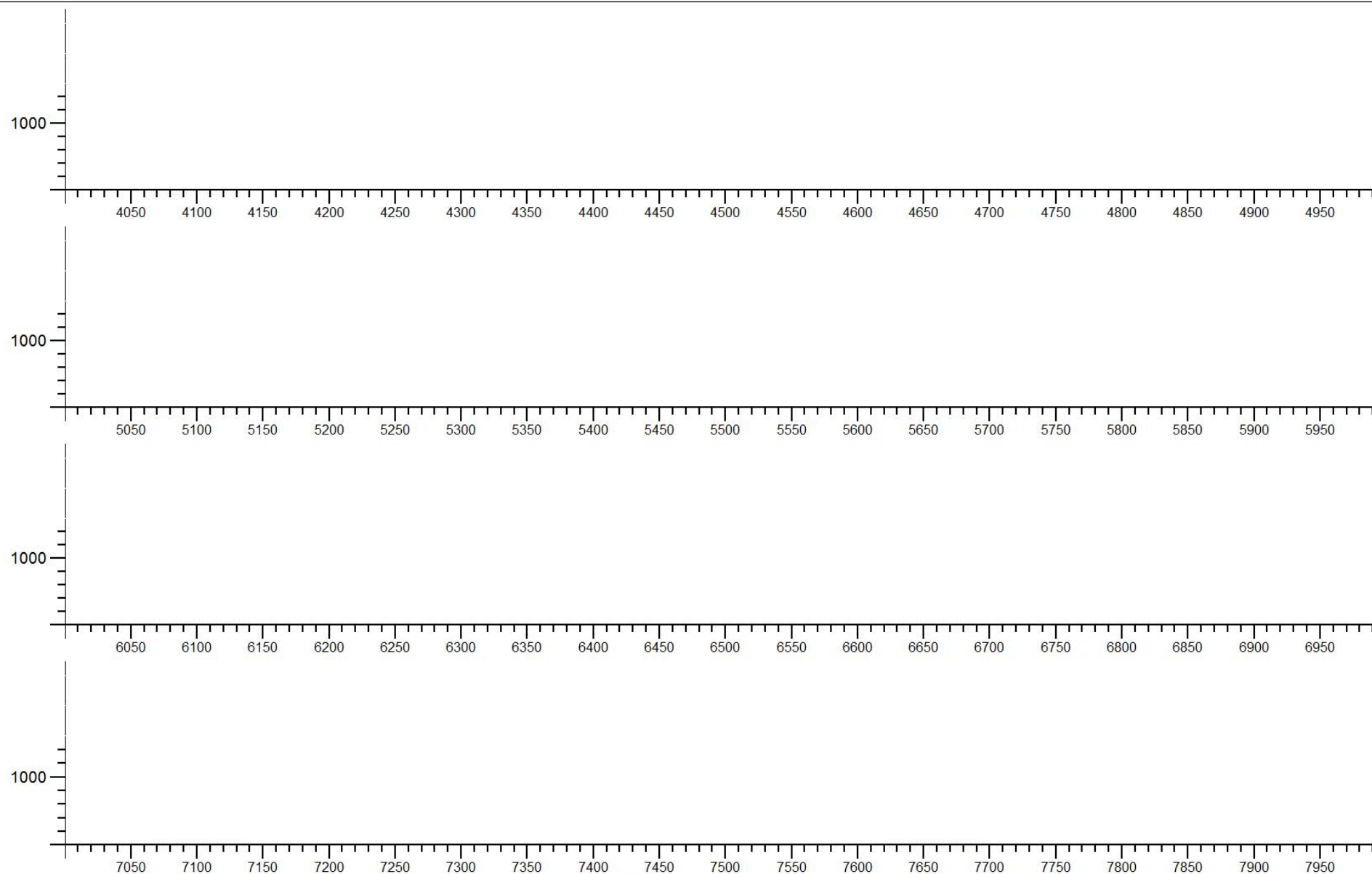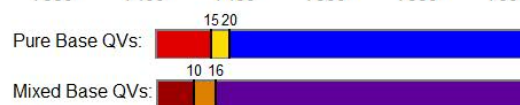

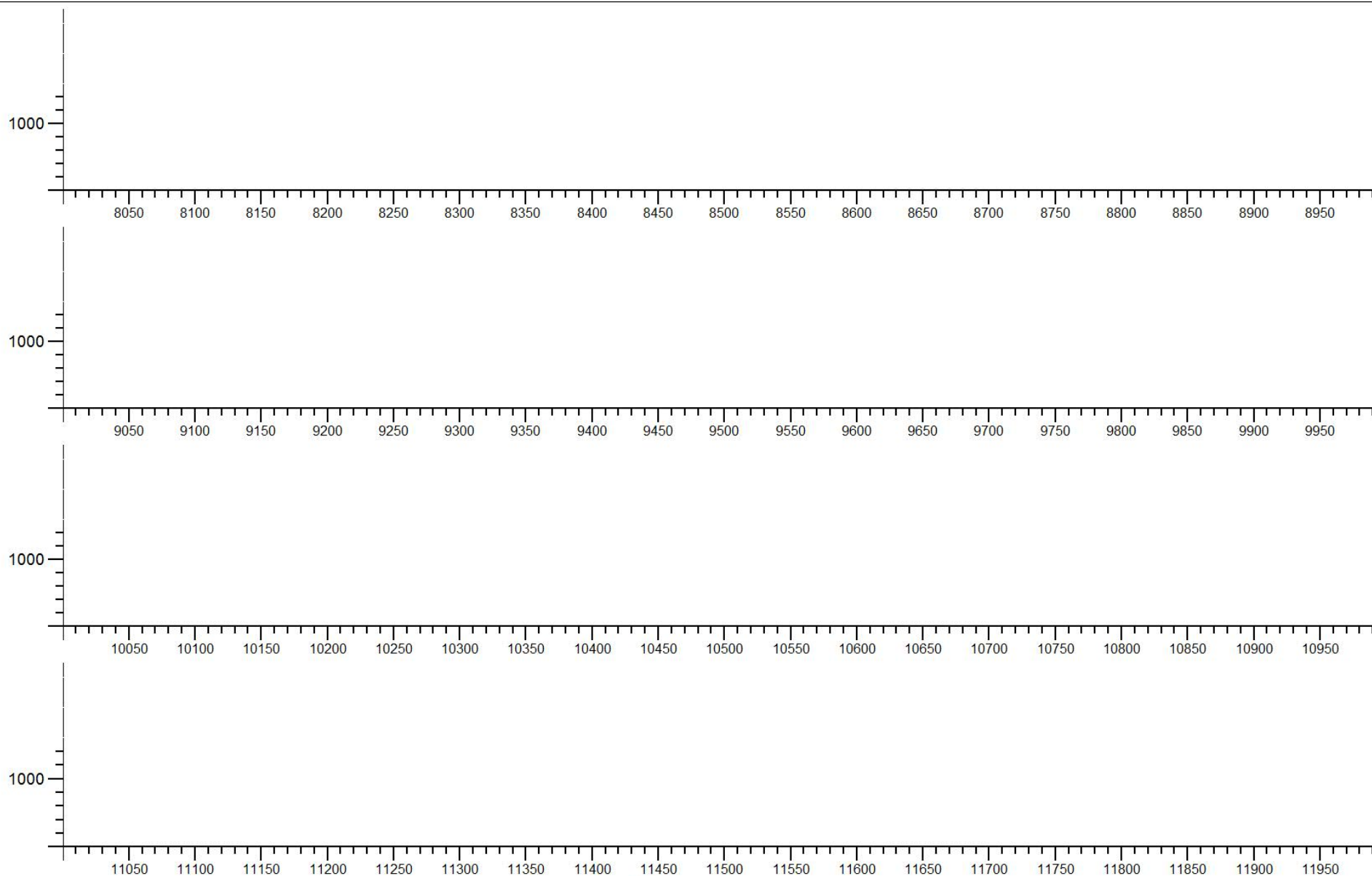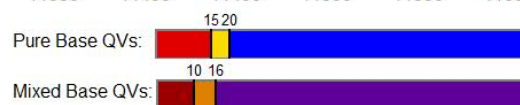

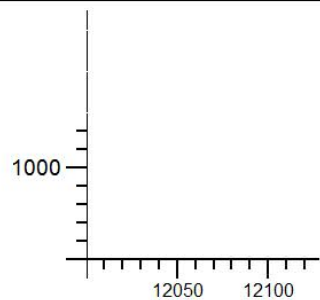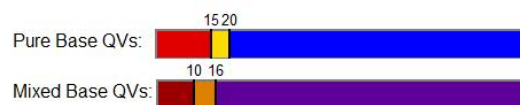

Supplement: Supplemental Information 1 — Chromatograms of: (1) recombined sequences of the H47 GI model from a number of mutants affected in recombination functions, and (2) recombined sequences of the pUYFRT model. [file peerj-05-3293-s001.zip › raw material/54-fimBE_out1_FA.pdf]

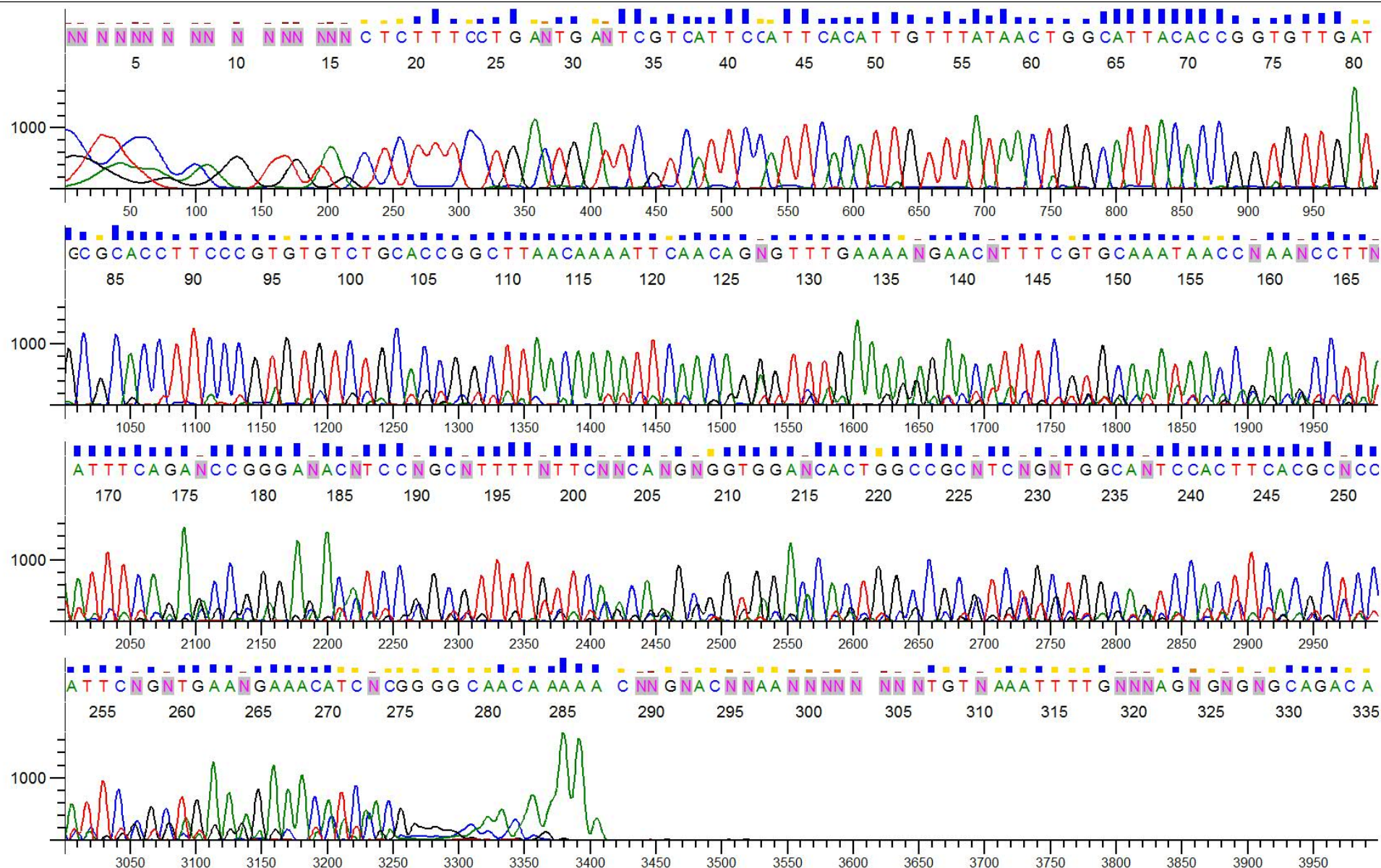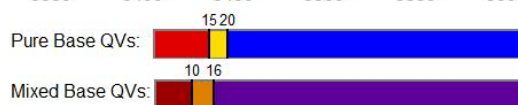

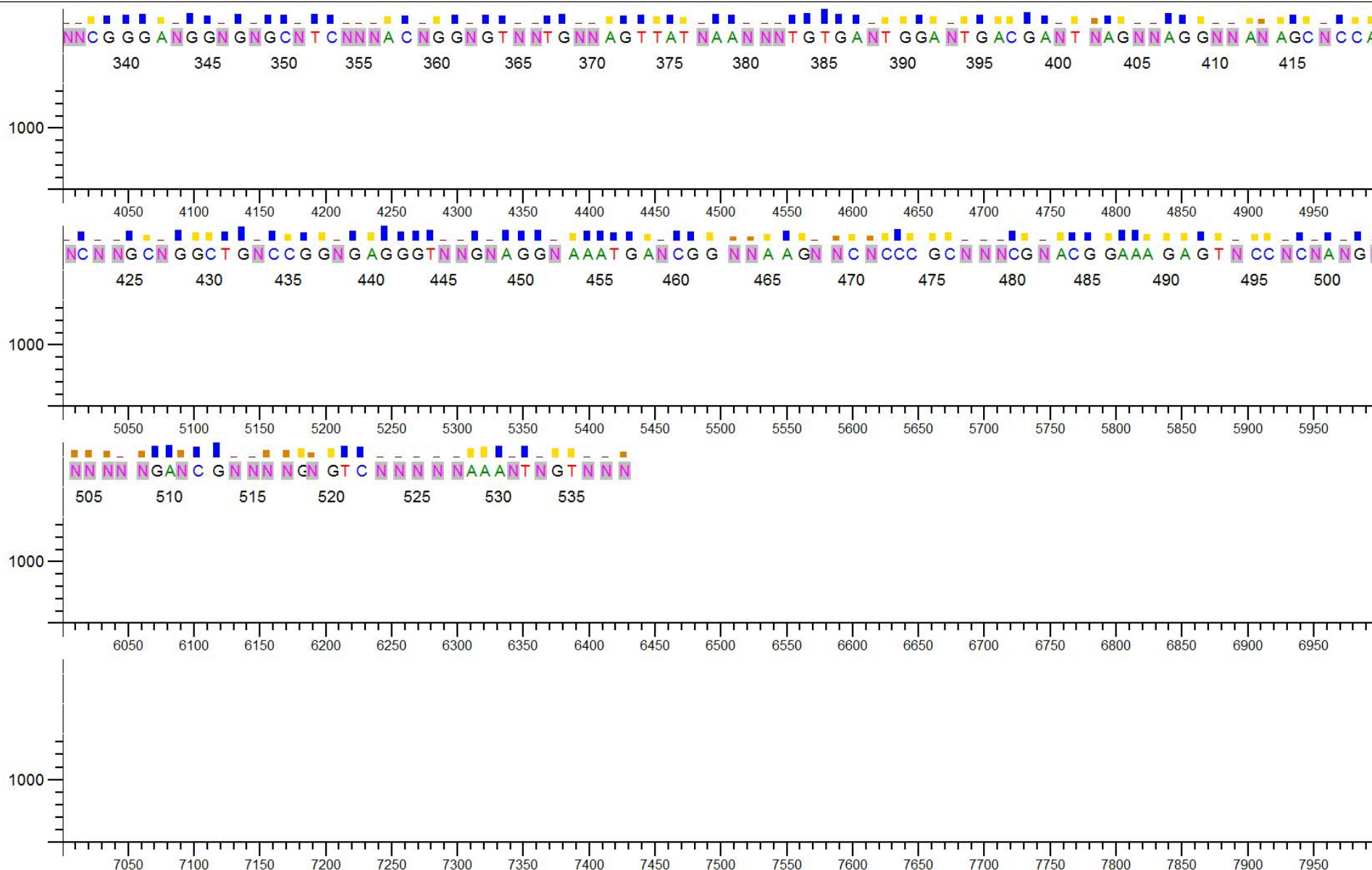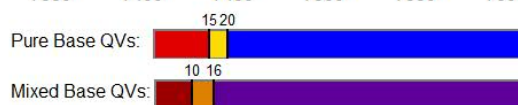

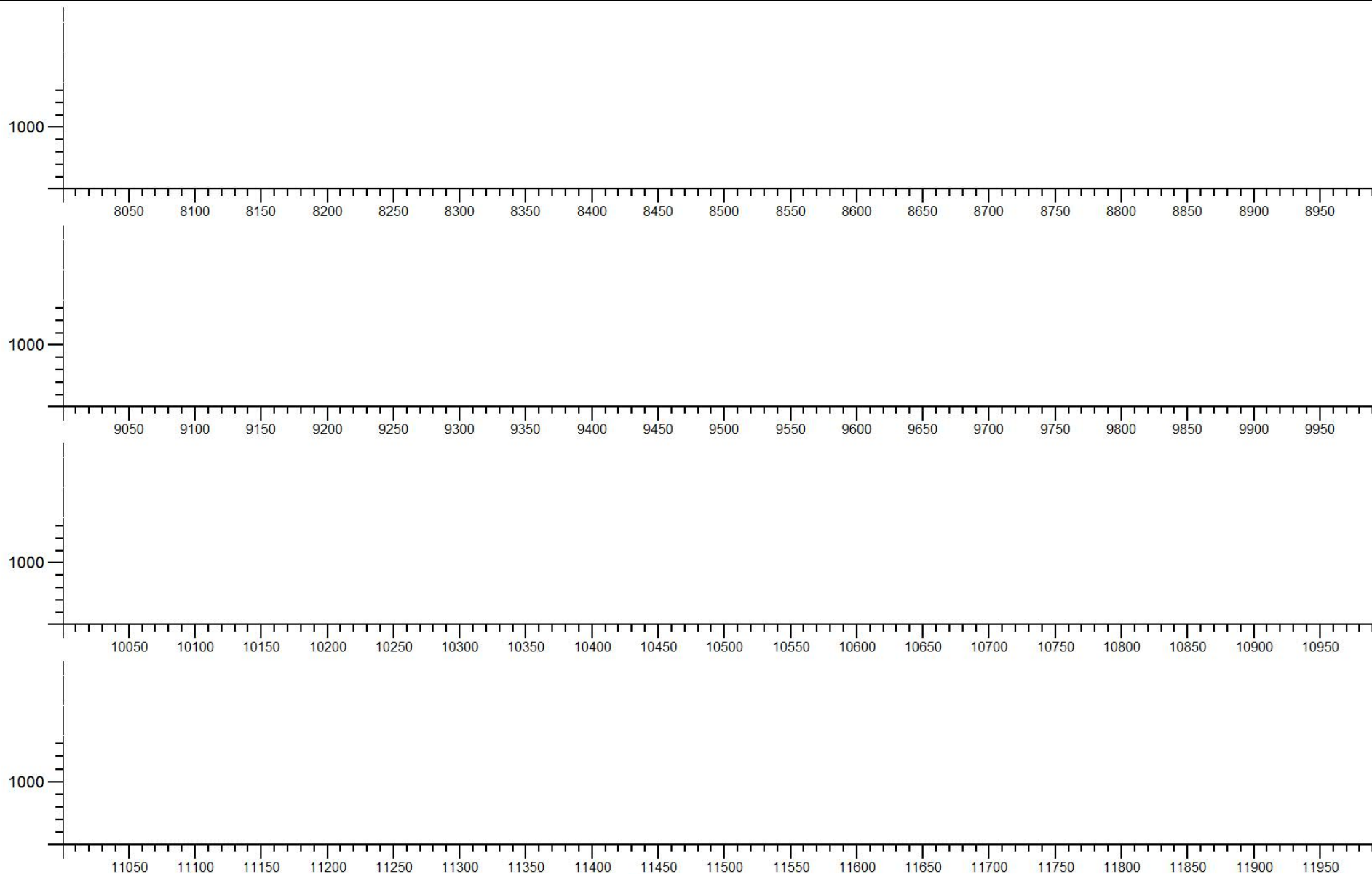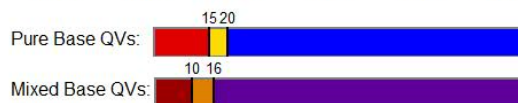

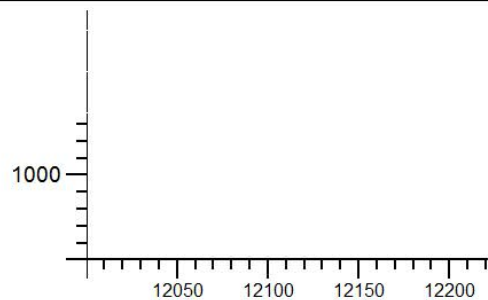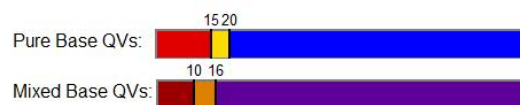

Supplement: Supplemental Information 1 — Chromatograms of: (1) recombined sequences of the H47 GI model from a number of mutants affected in recombination functions, and (2) recombined sequences of the pUYFRT model. [file peerj-05-3293-s001.zip › raw material/54-Pin_out1_FA.pdf]

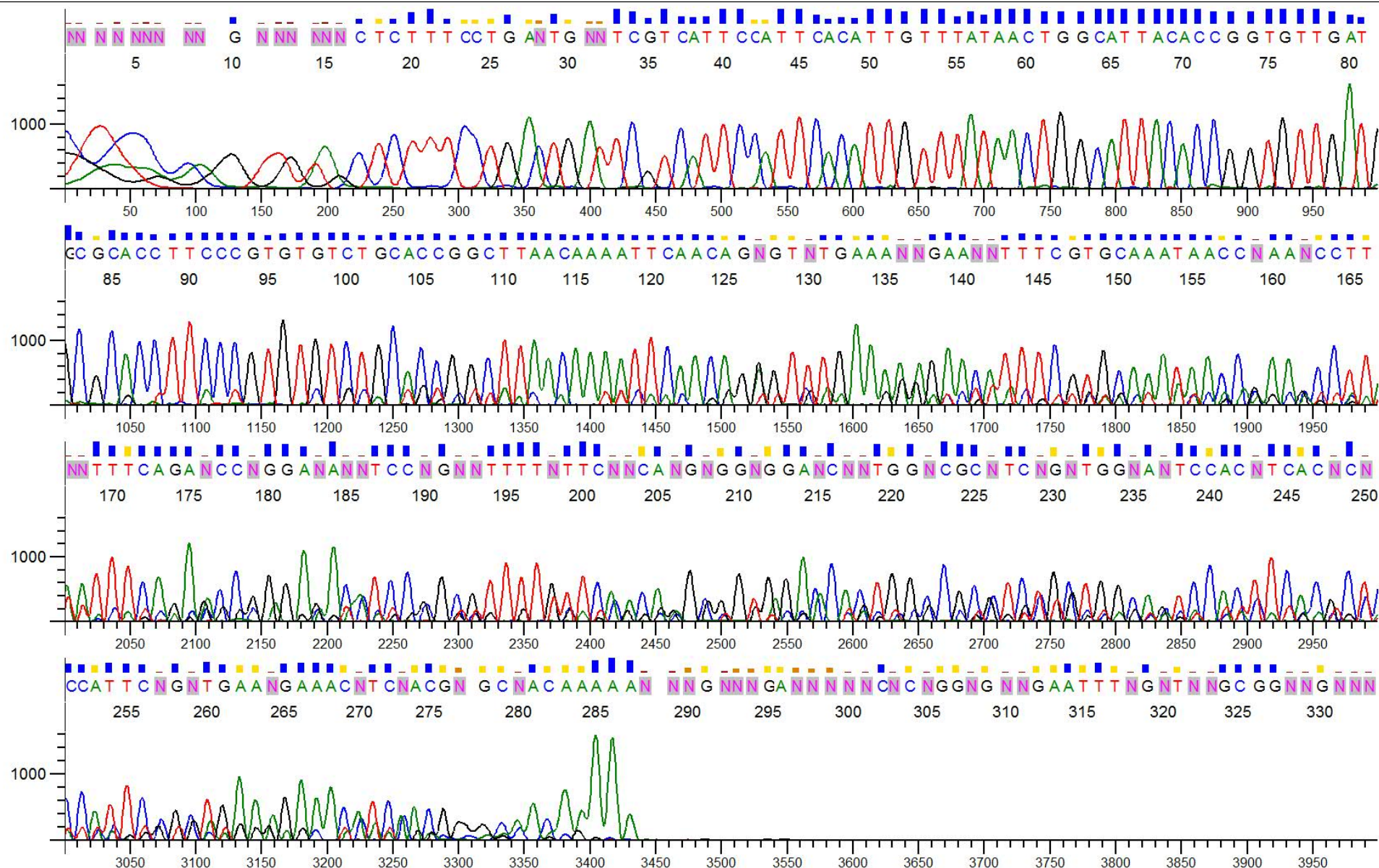

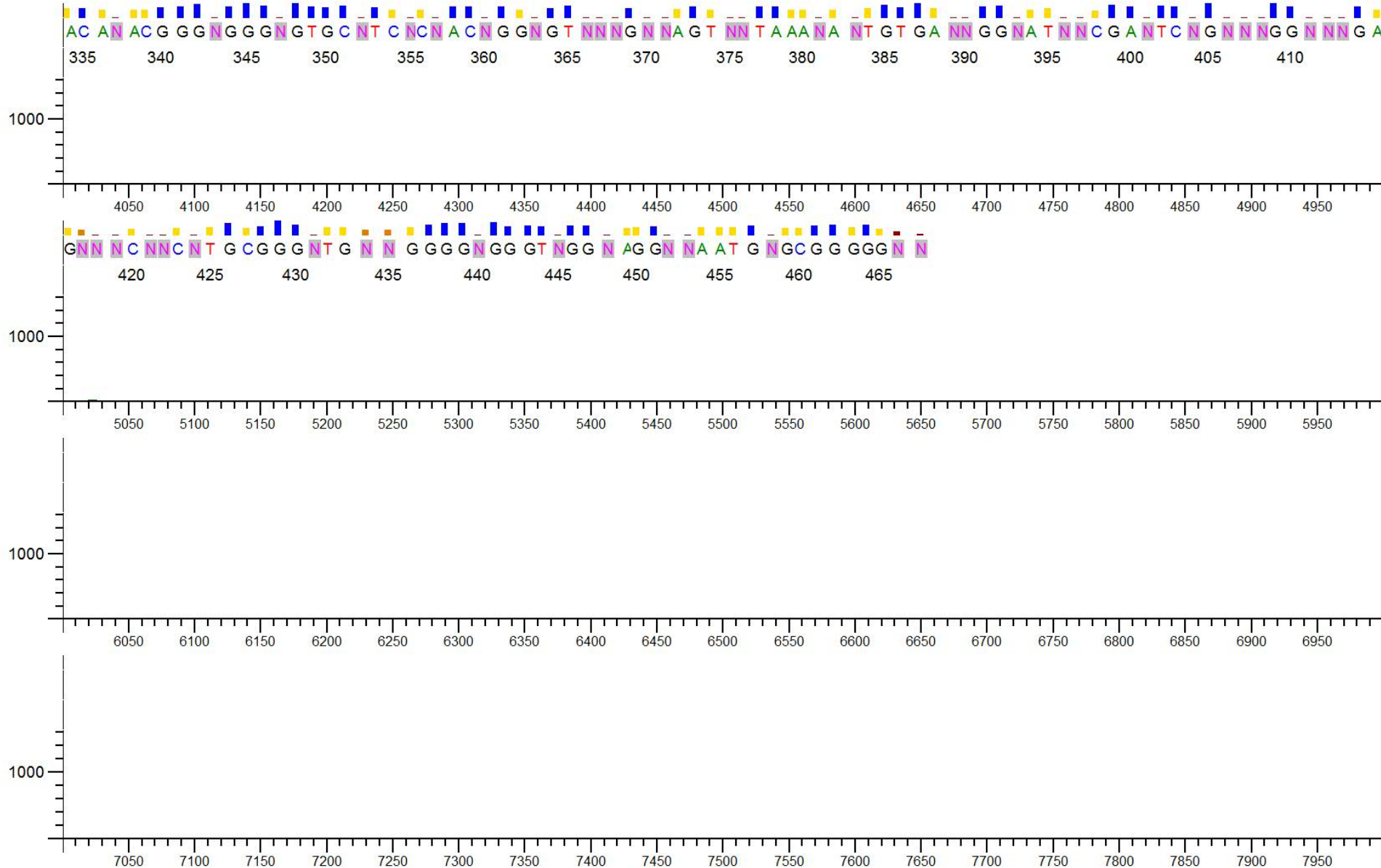

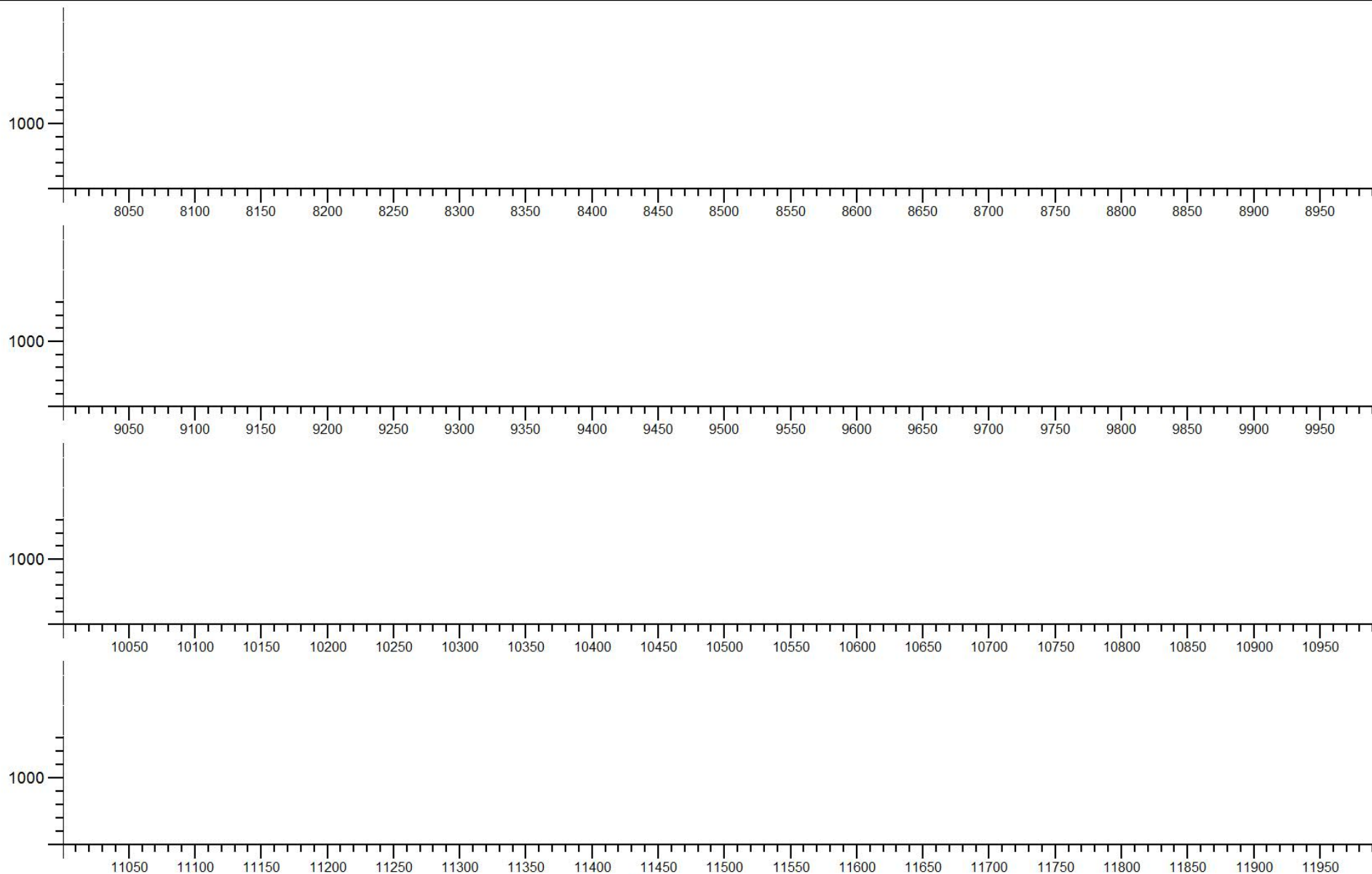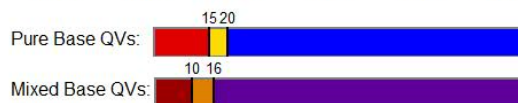

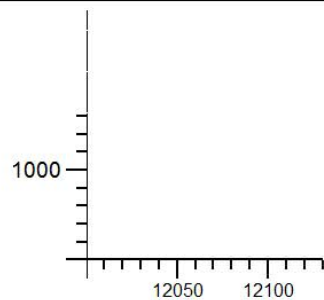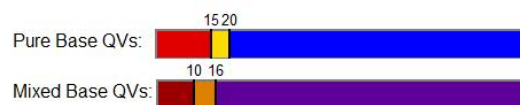

Supplement: Supplemental Information 1 — Chromatograms of: (1) recombined sequences of the H47 GI model from a number of mutants affected in recombination functions, and (2) recombined sequences of the pUYFRT model. [file peerj-05-3293-s001.zip › raw material/55-PinR_out1_FA.pdf]

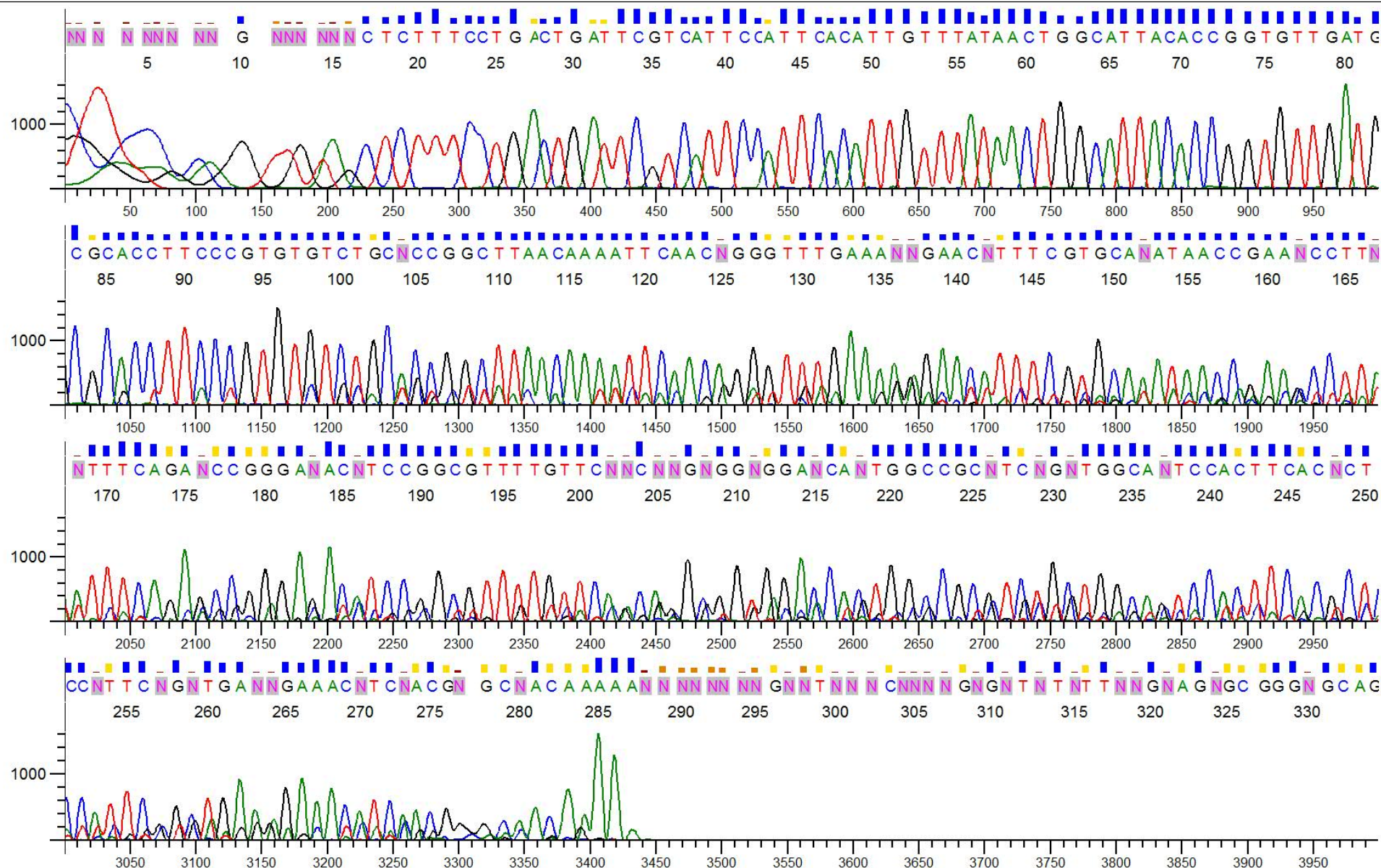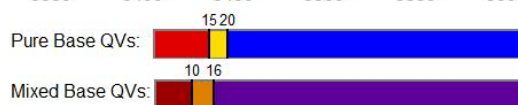

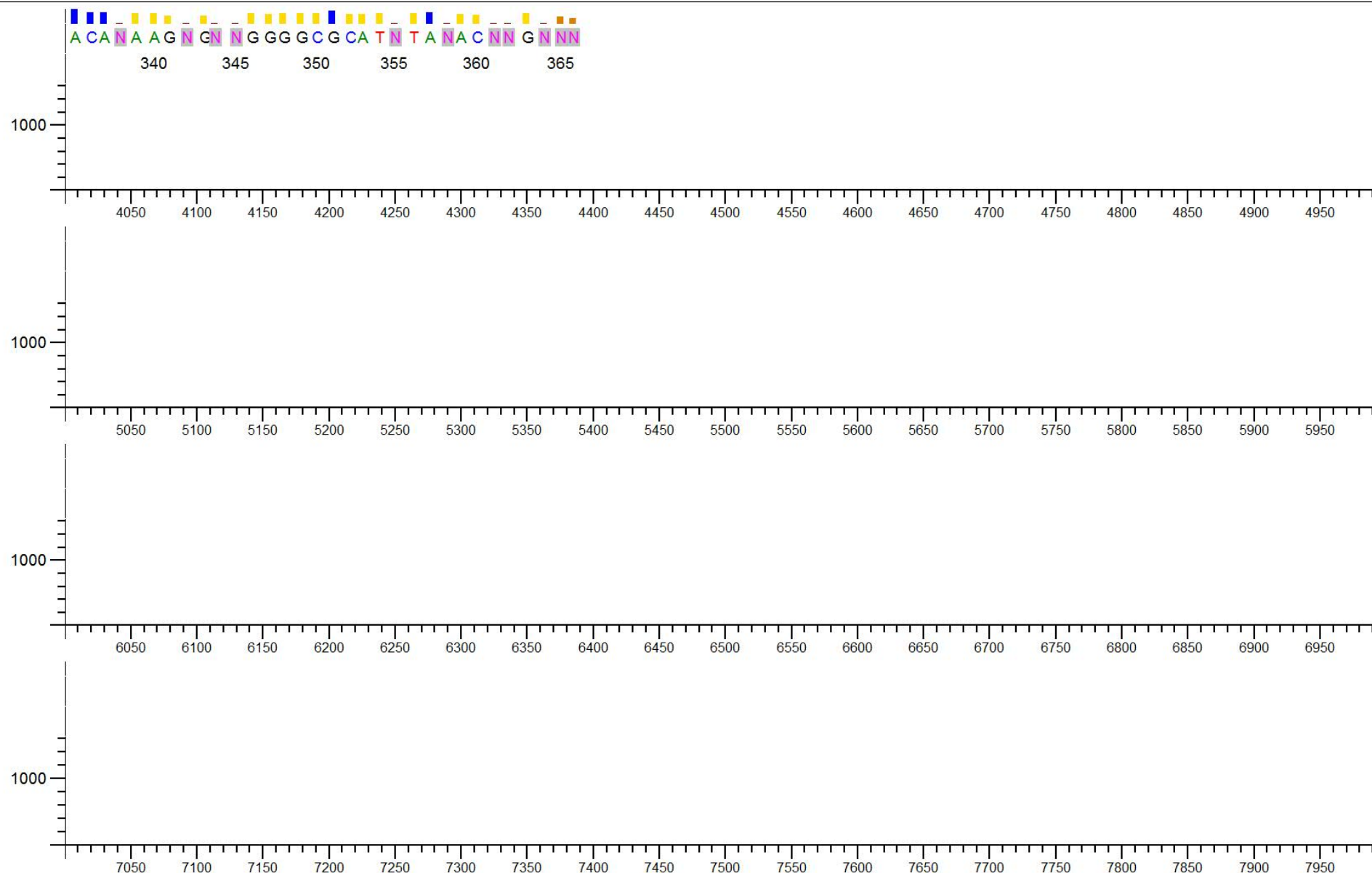

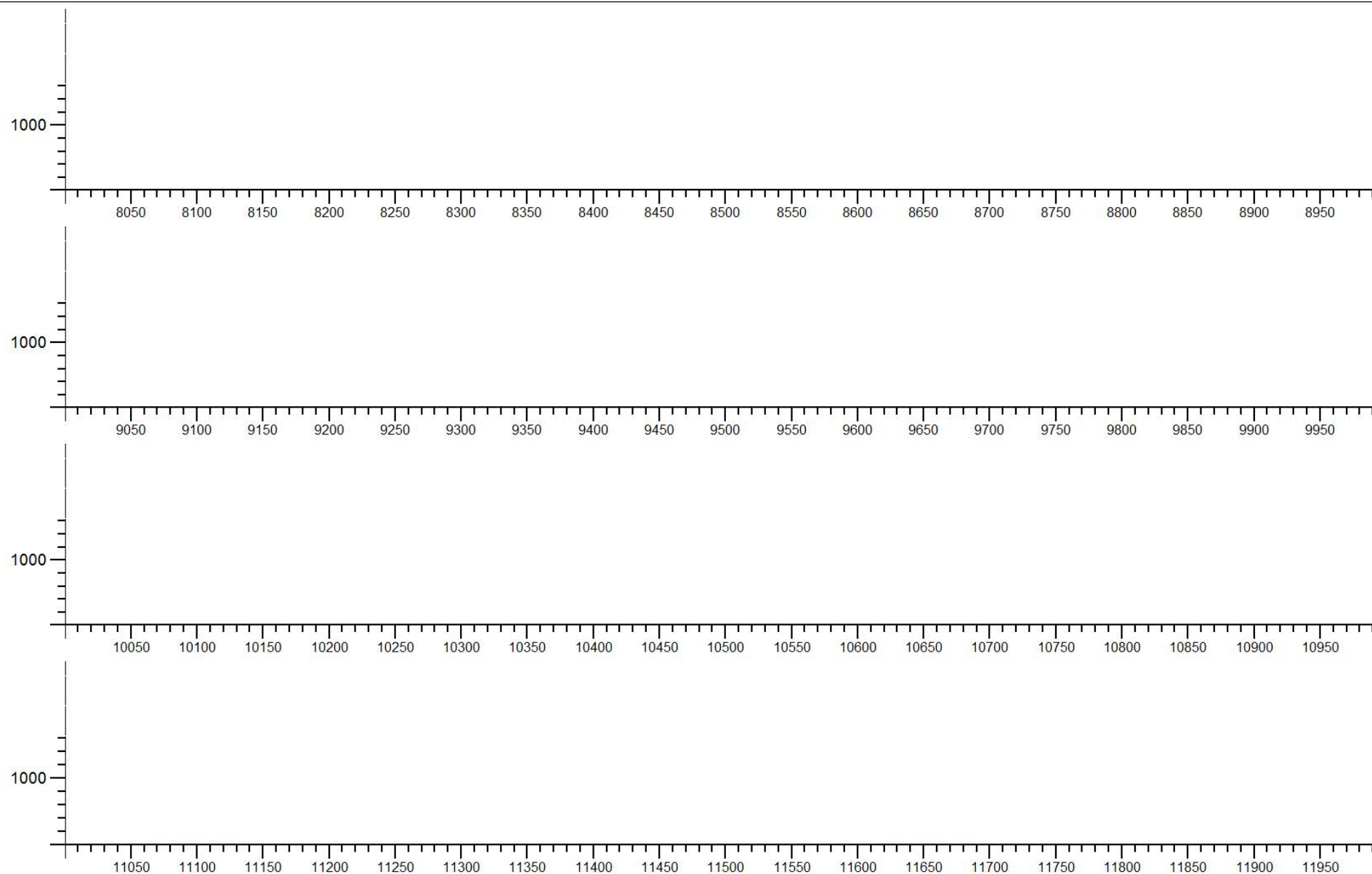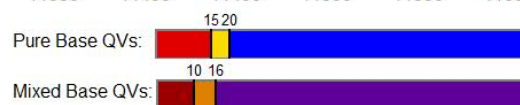

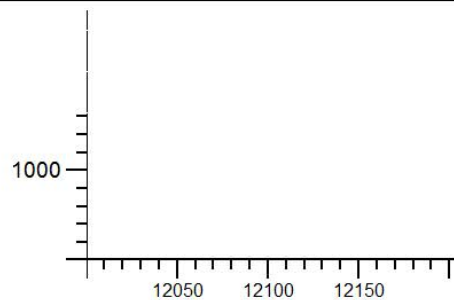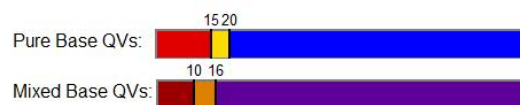

Supplement: Supplemental Information 1 — Chromatograms of: (1) recombined sequences of the H47 GI model from a number of mutants affected in recombination functions, and (2) recombined sequences of the pUYFRT model. [file peerj-05-3293-s001.zip › raw material/55-xerCxerD_out1_FA.pdf]

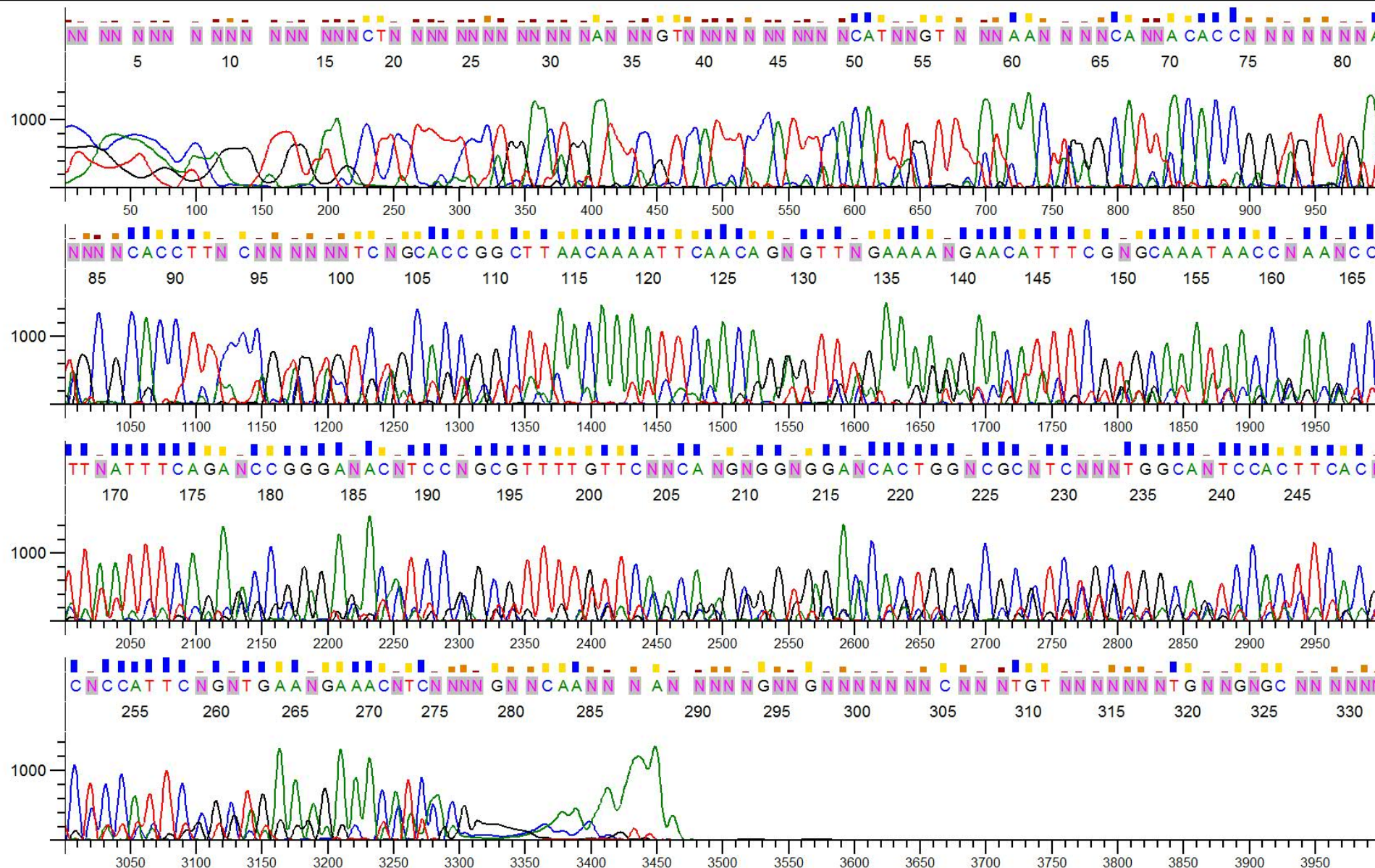

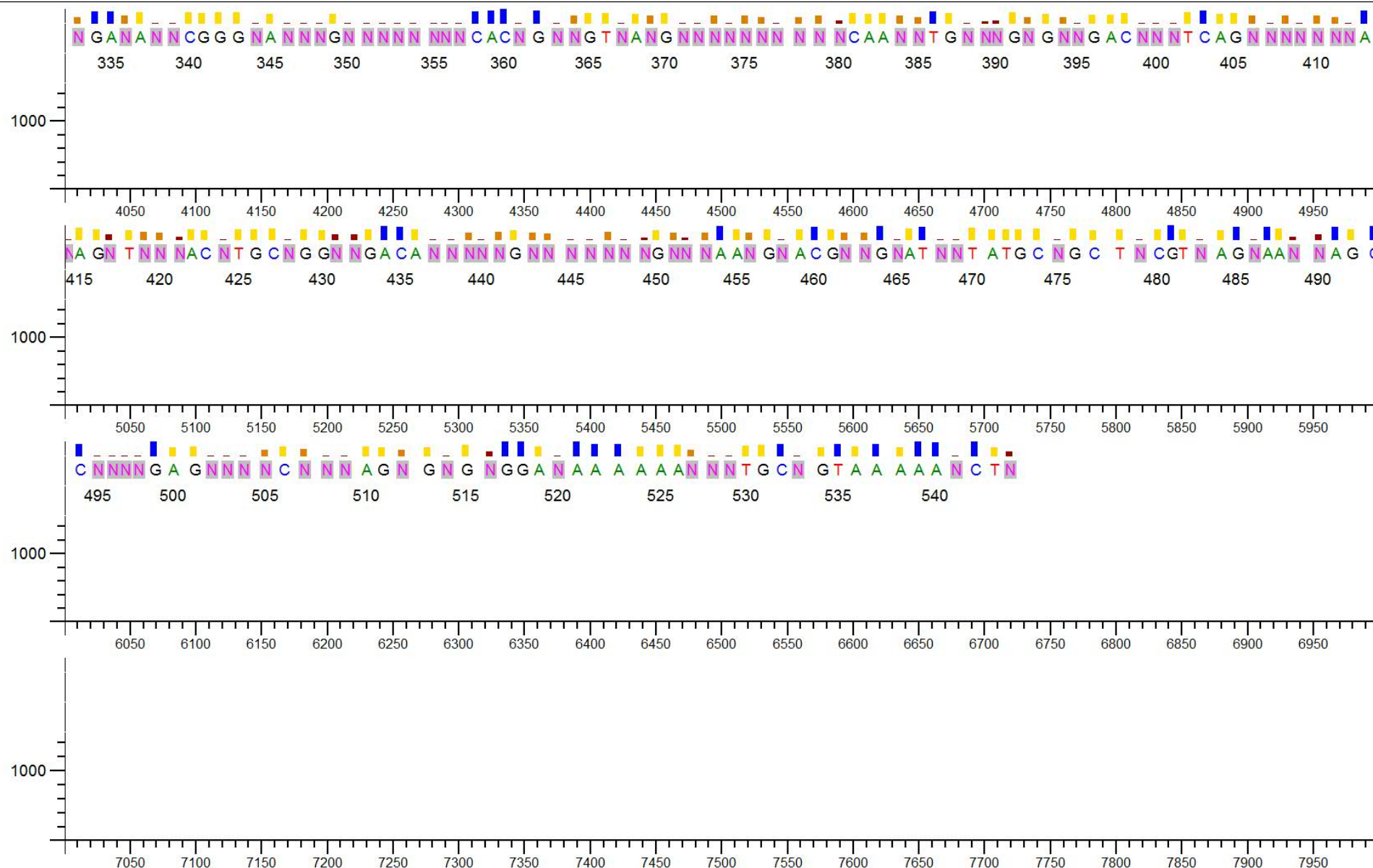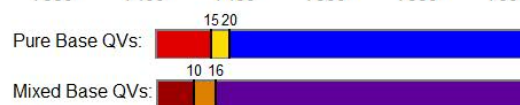

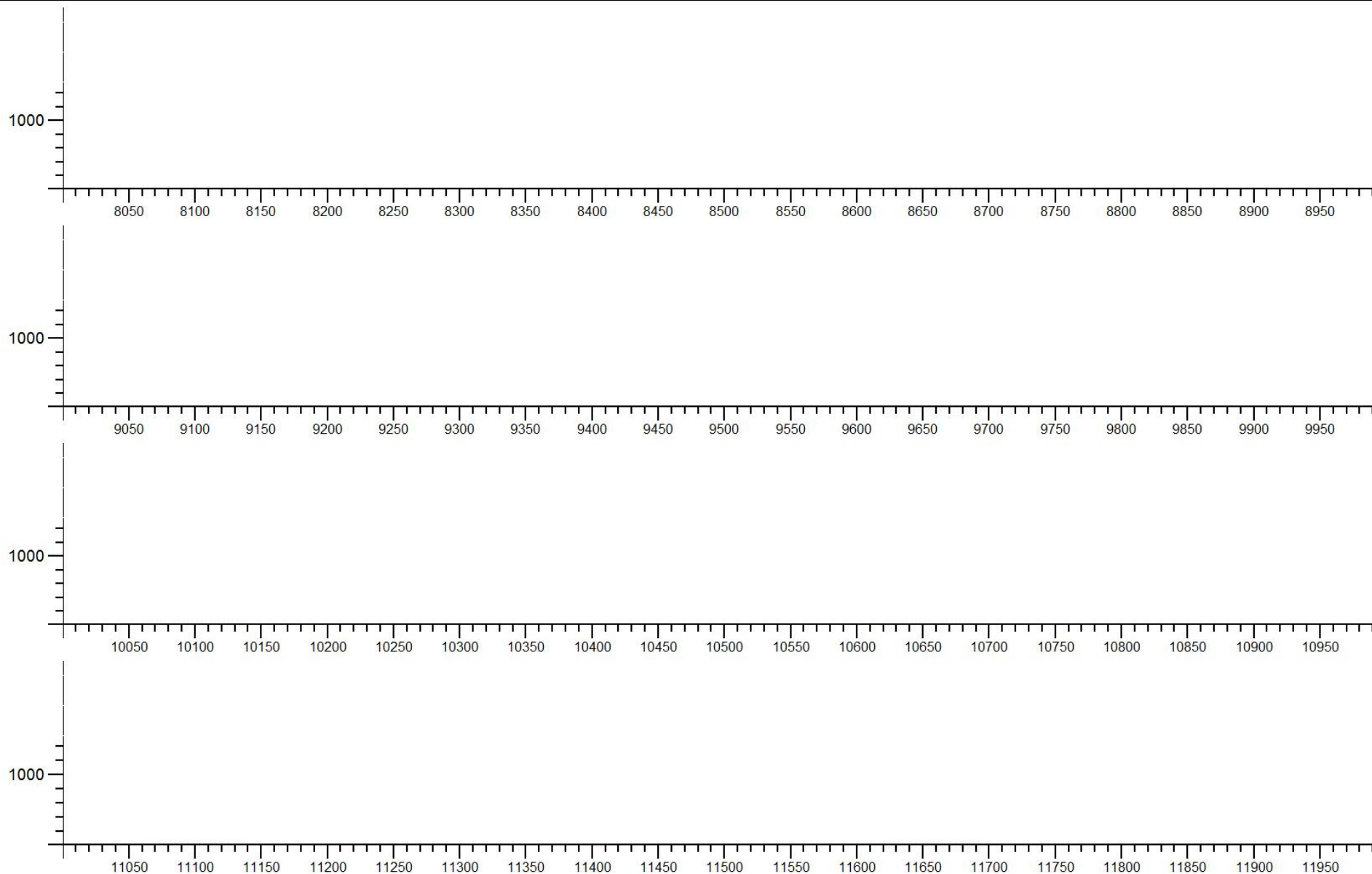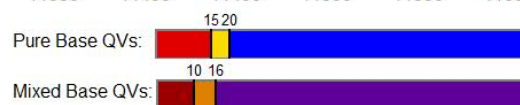

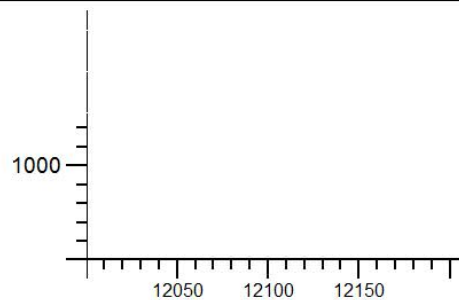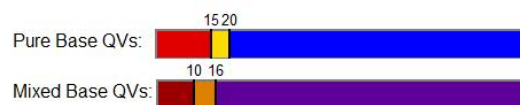

Supplement: Supplemental Information 1 — Chromatograms of: (1) recombined sequences of the H47 GI model from a number of mutants affected in recombination functions, and (2) recombined sequences of the pUYFRT model. [file peerj-05-3293-s001.zip › raw material/56-IntE_out1_FA.pdf]

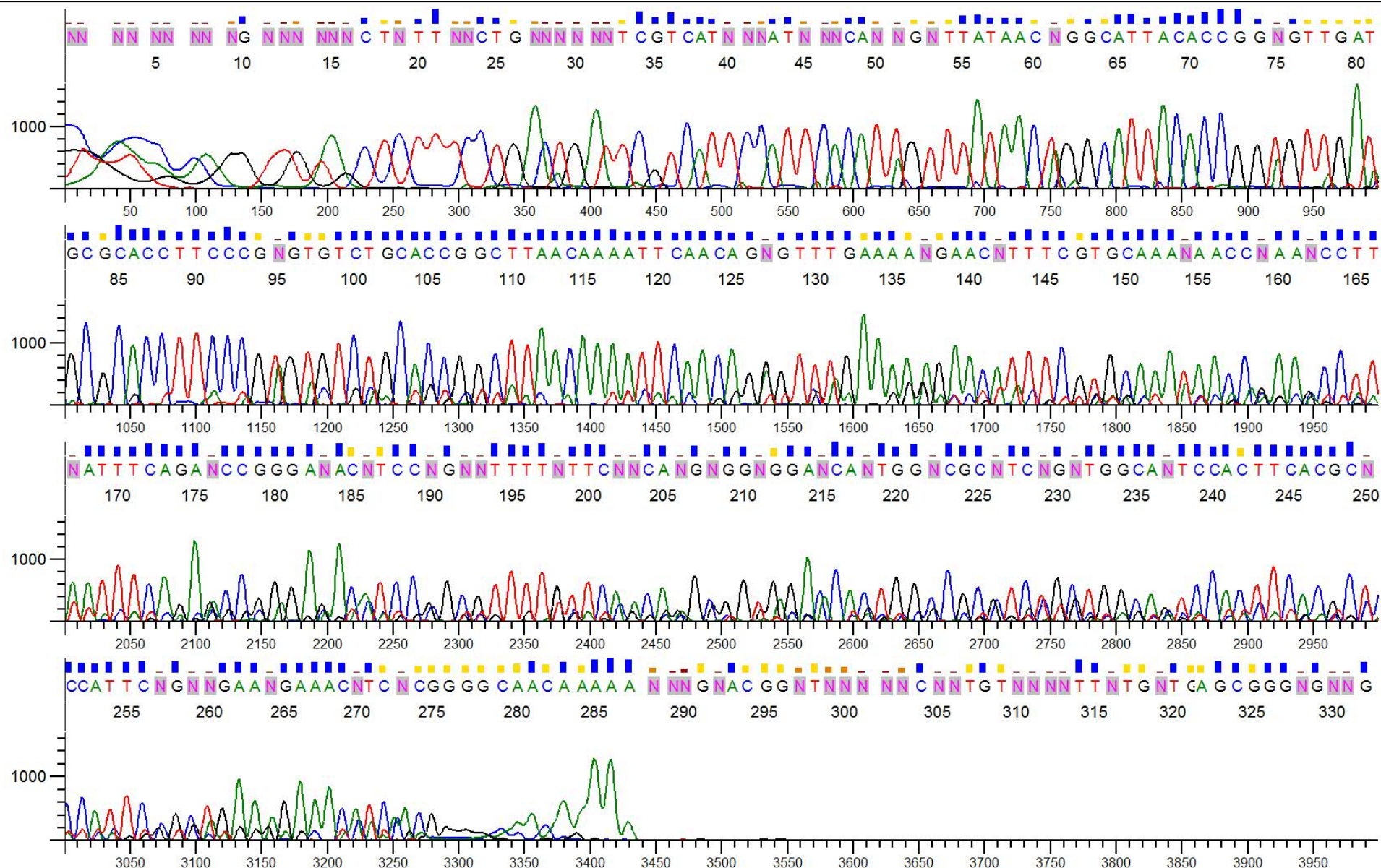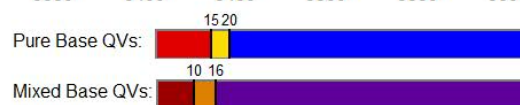

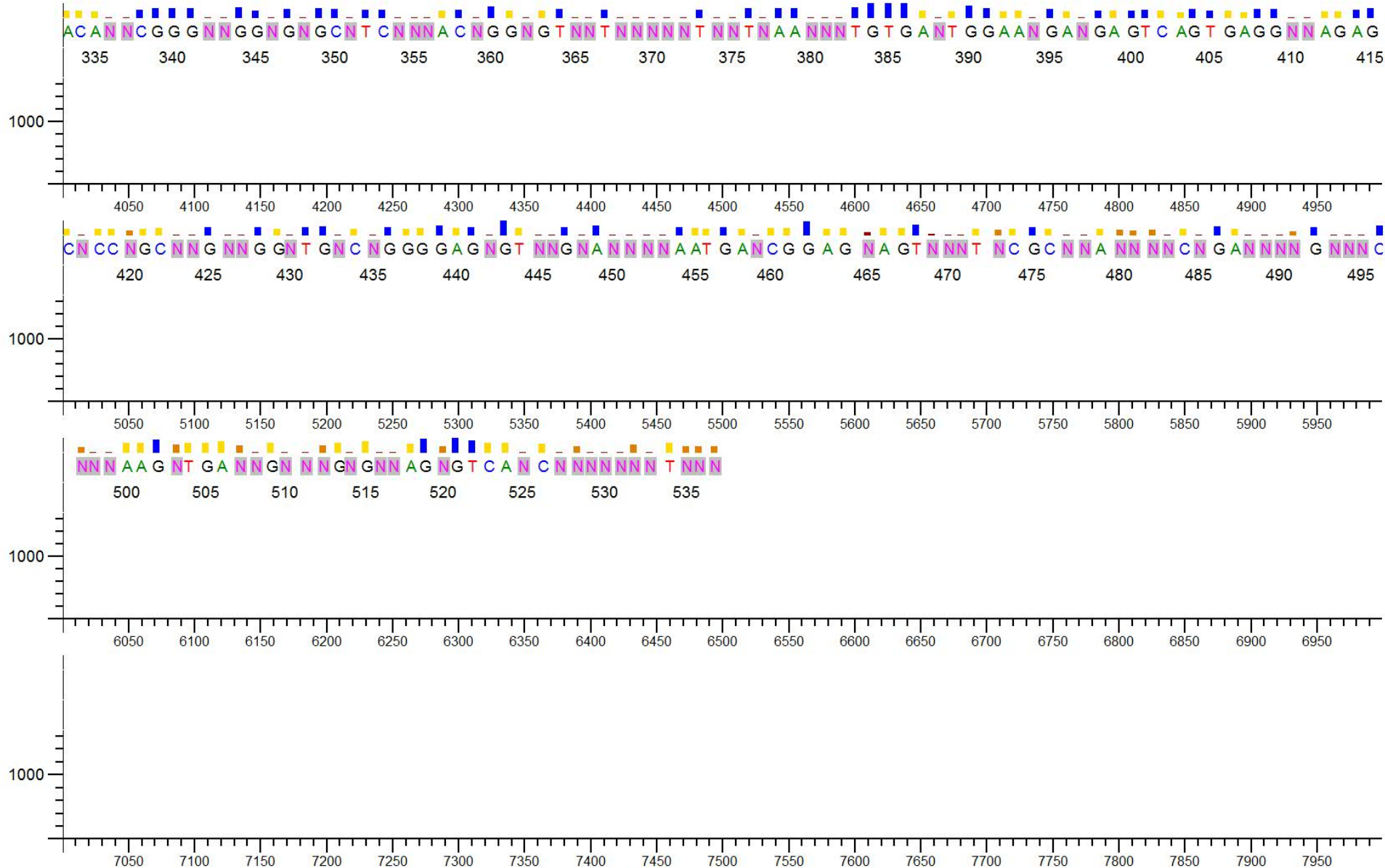

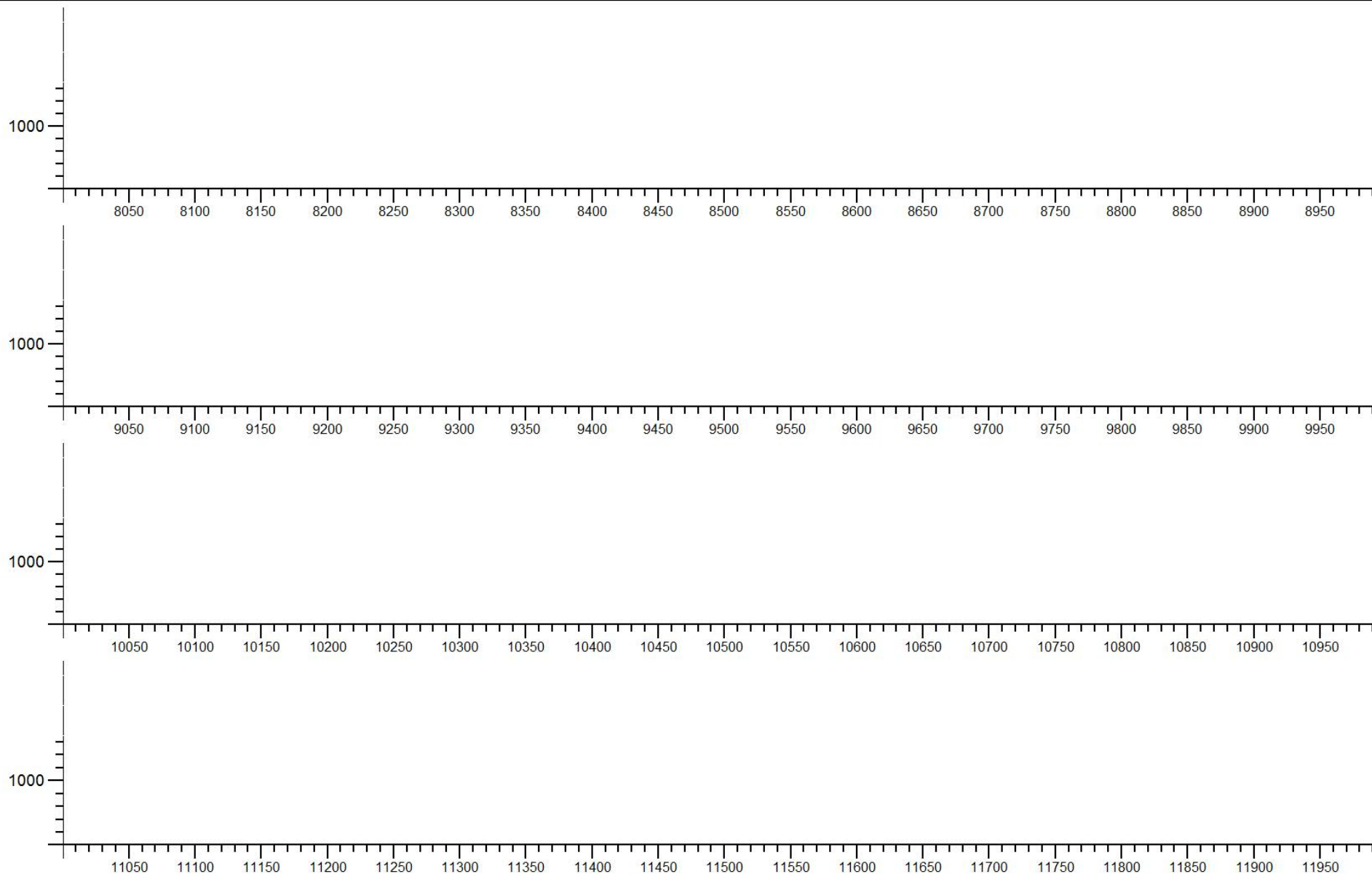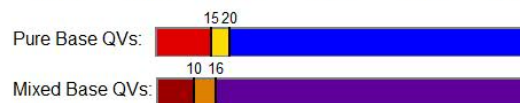

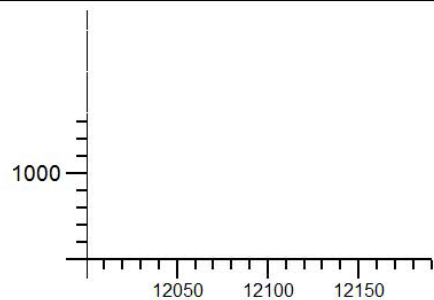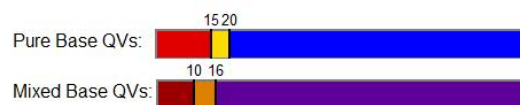

Supplement: Supplemental Information 1 — Chromatograms of: (1) recombined sequences of the H47 GI model from a number of mutants affected in recombination functions, and (2) recombined sequences of the pUYFRT model. [file peerj-05-3293-s001.zip › raw material/57-IntR_out1_FA.pdf]

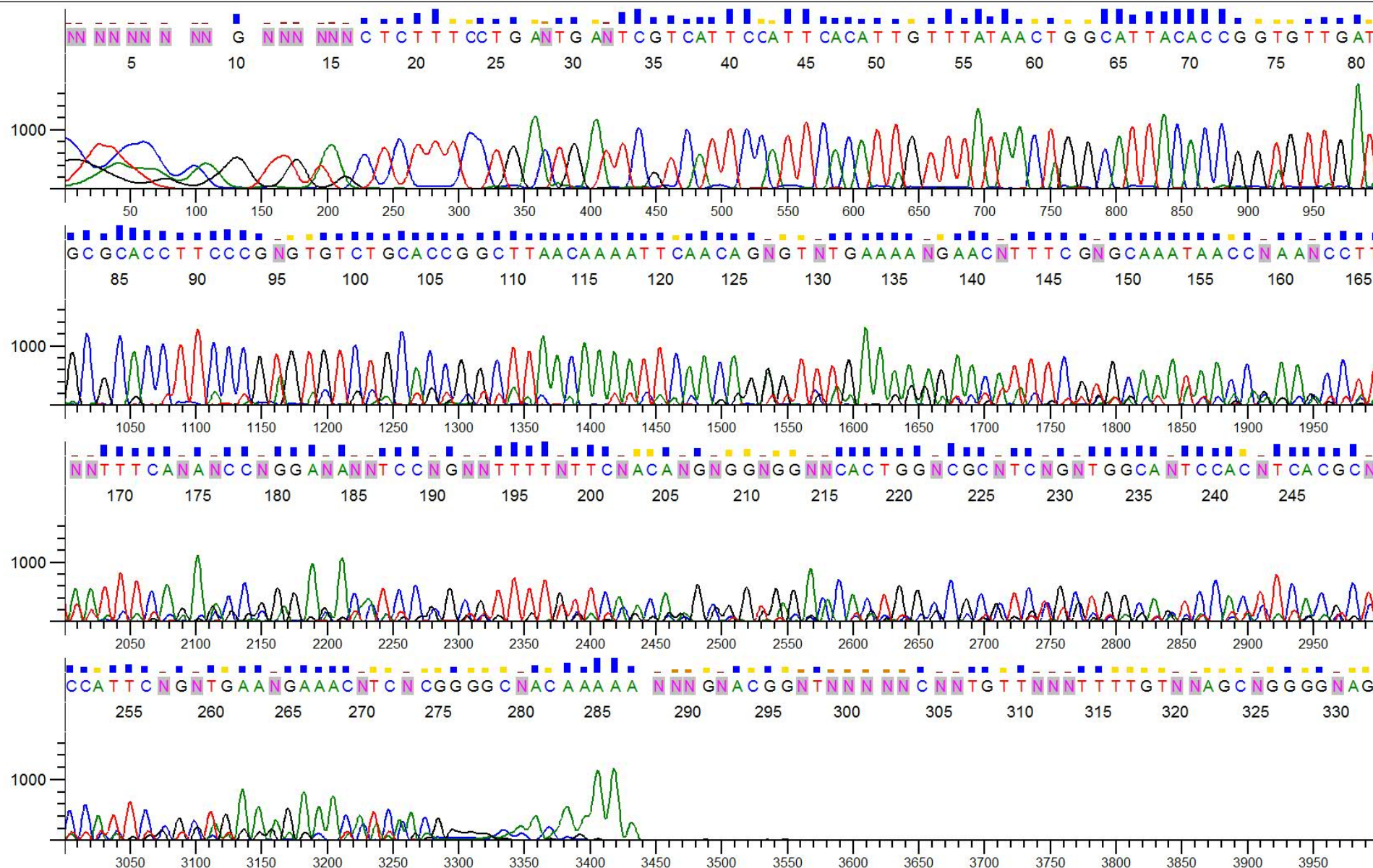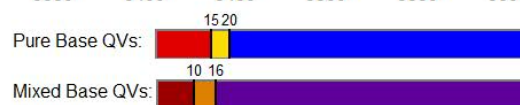

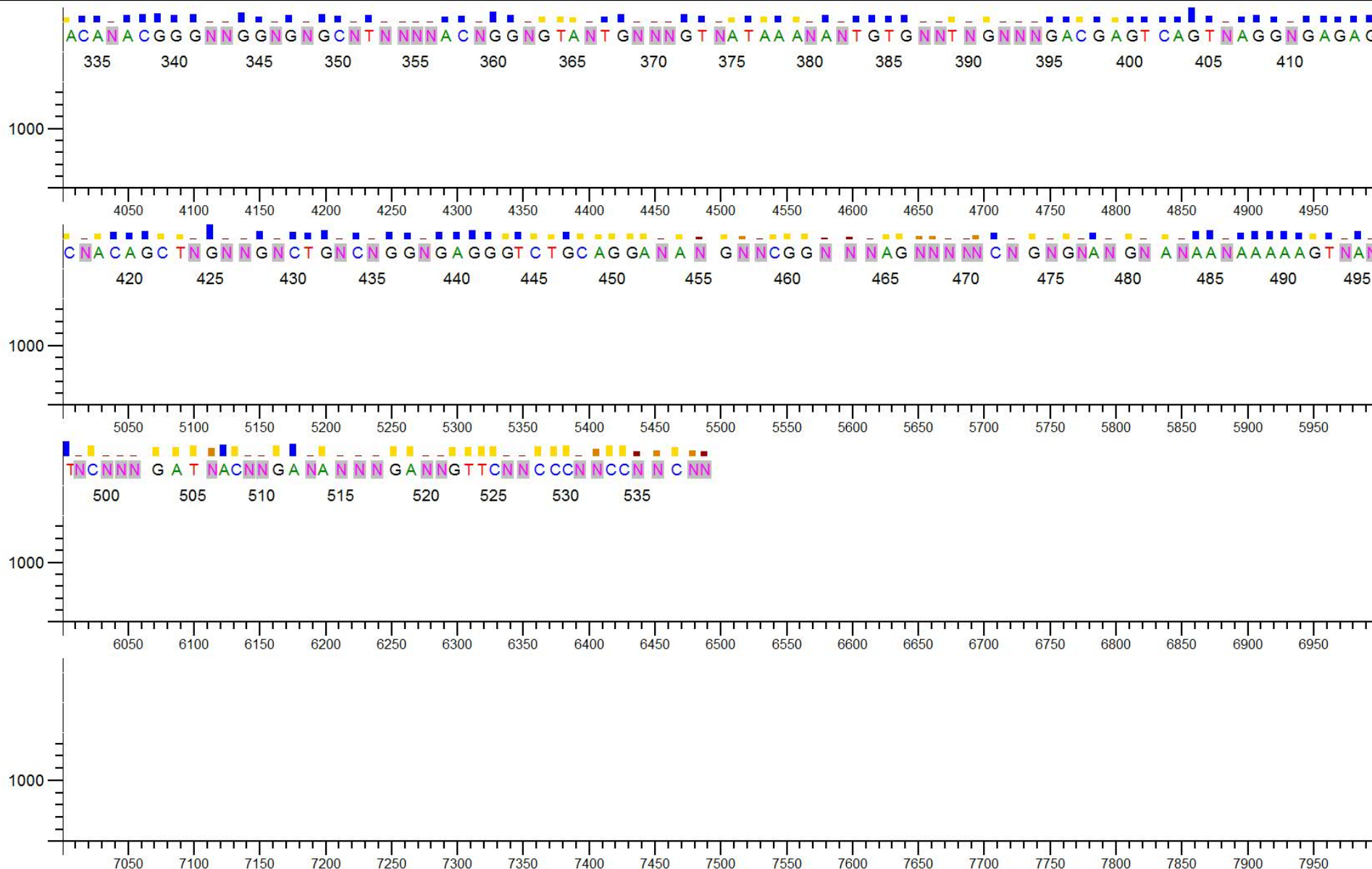

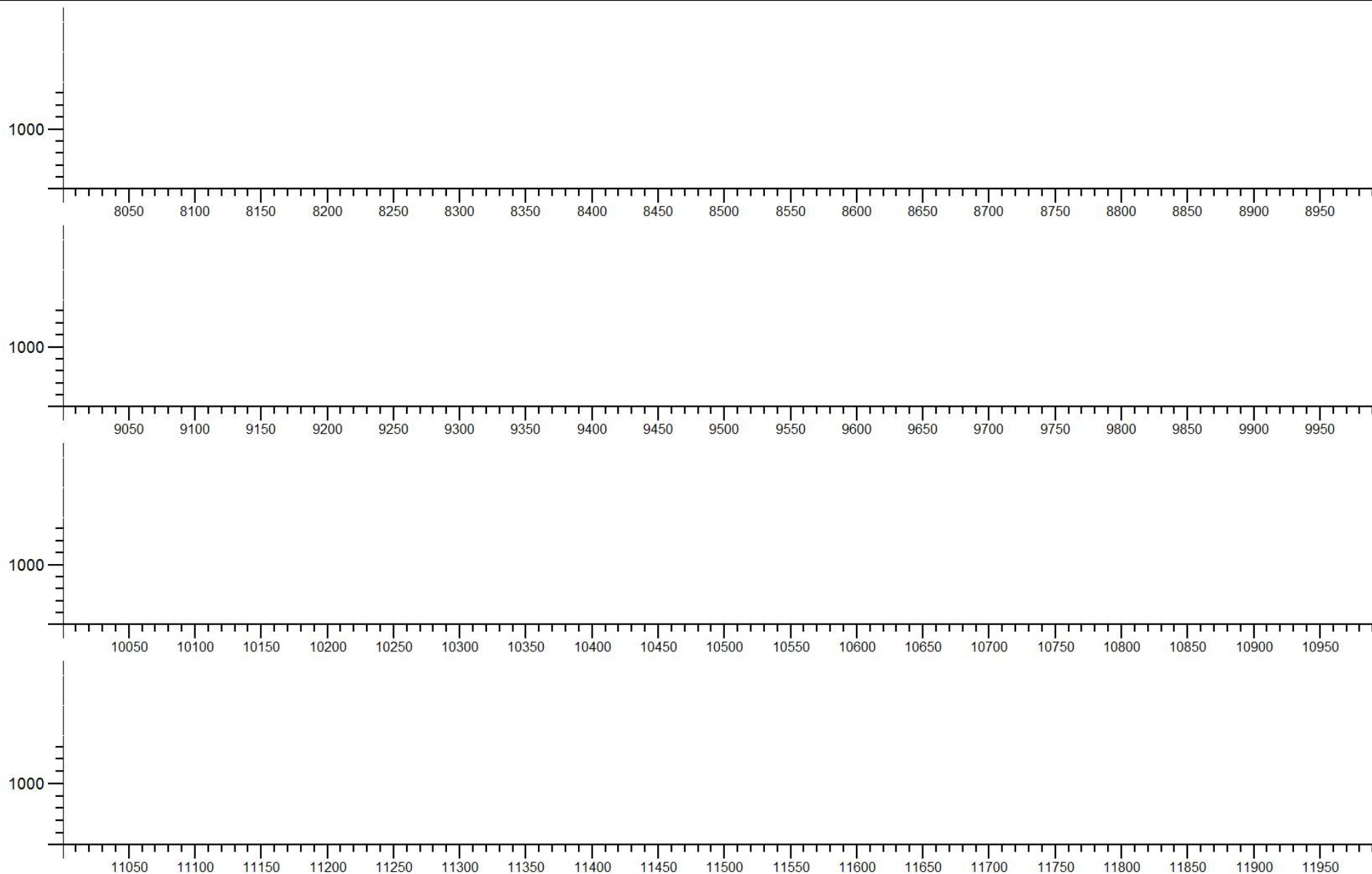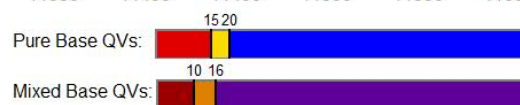

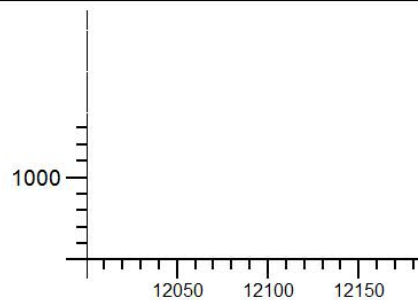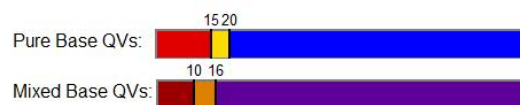

Supplement: Supplemental Information 1 — Chromatograms of: (1) recombined sequences of the H47 GI model from a number of mutants affected in recombination functions, and (2) recombined sequences of the pUYFRT model. [file peerj-05-3293-s001.zip › raw material/58-IntQ_out1_FA.pdf]

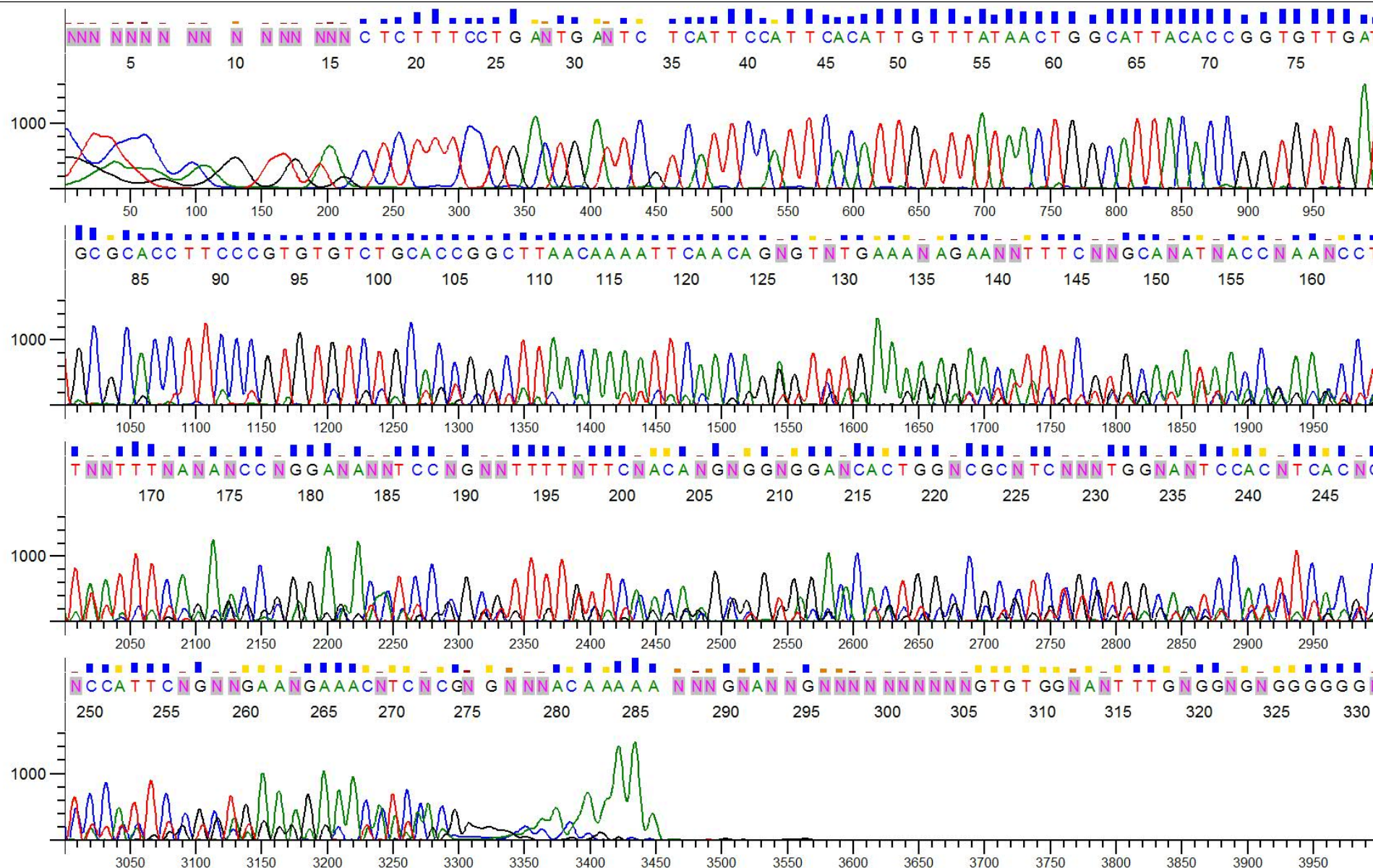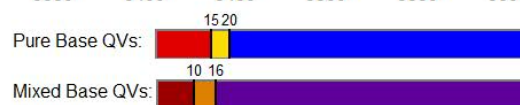

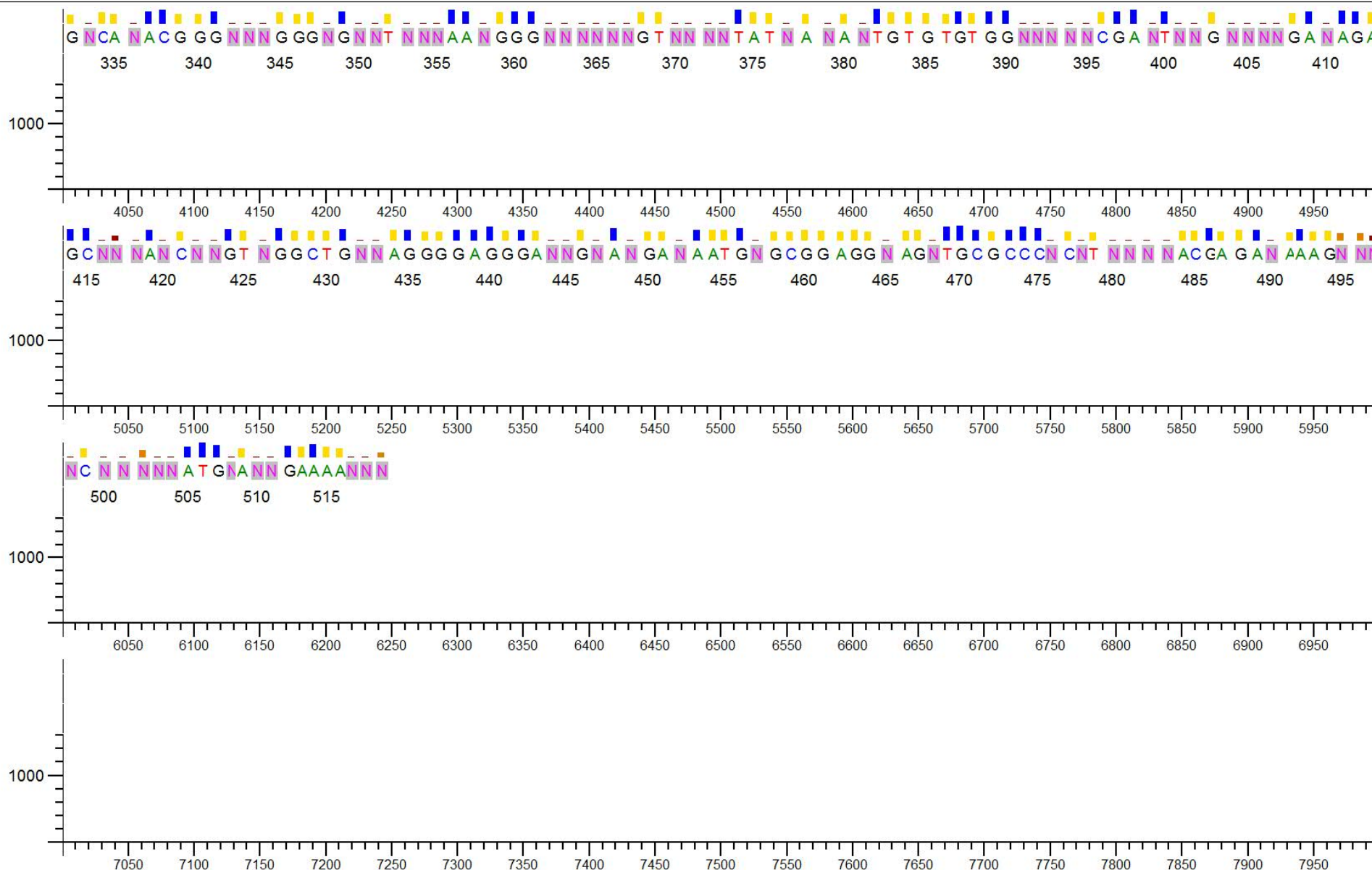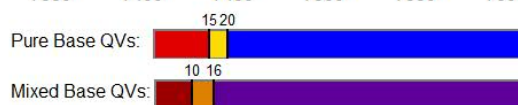

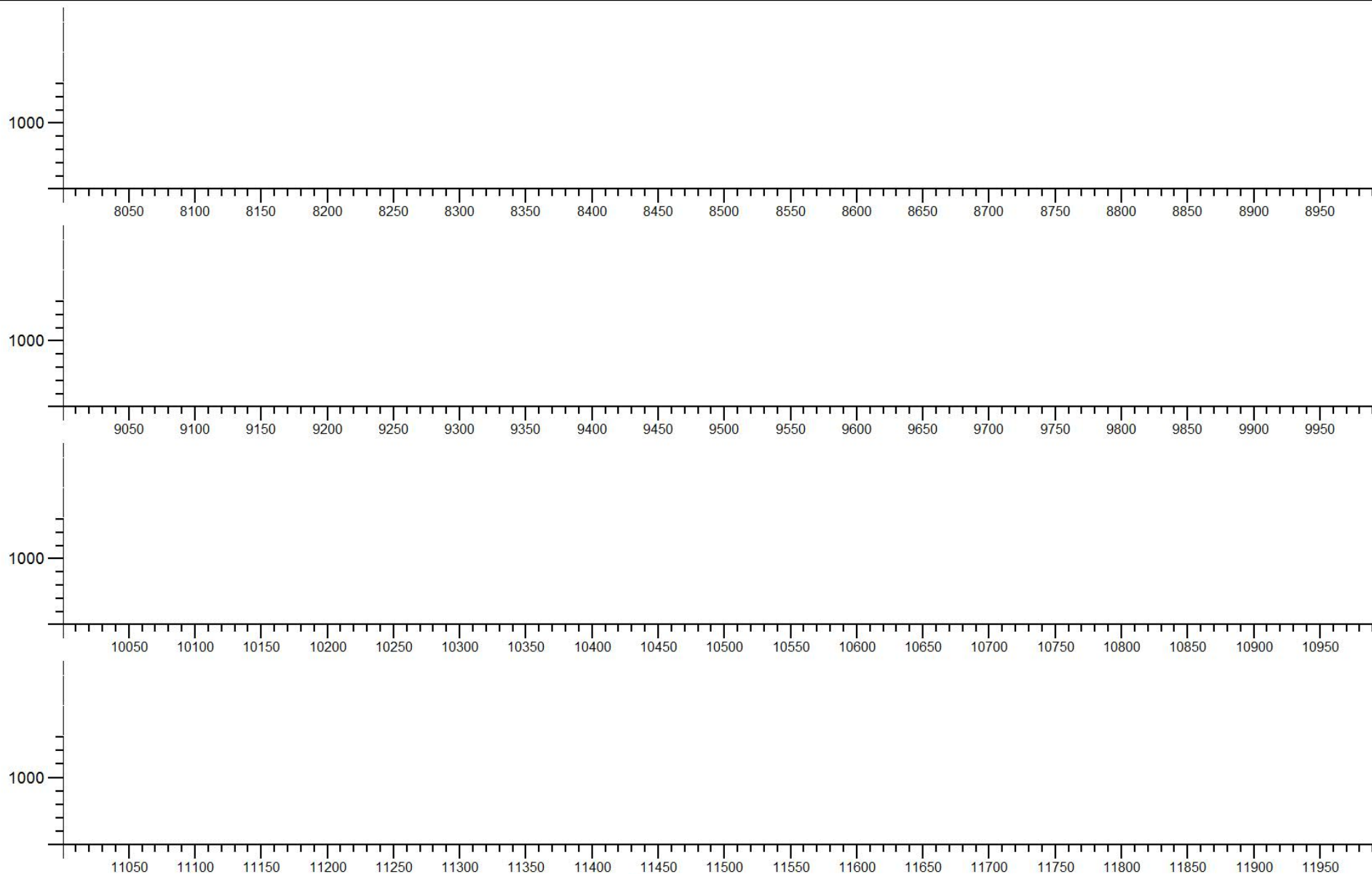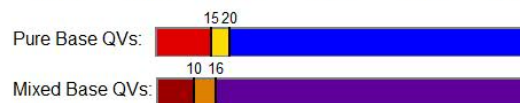

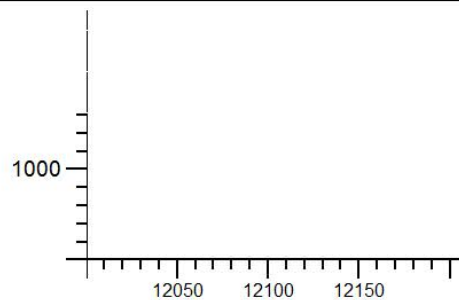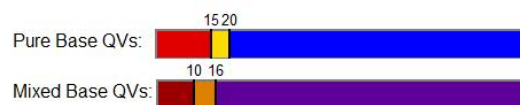

Supplement: Supplemental Information 1 — Chromatograms of: (1) recombined sequences of the H47 GI model from a number of mutants affected in recombination functions, and (2) recombined sequences of the pUYFRT model. [file peerj-05-3293-s001.zip › raw material/59-intA_out1_FA.pdf]

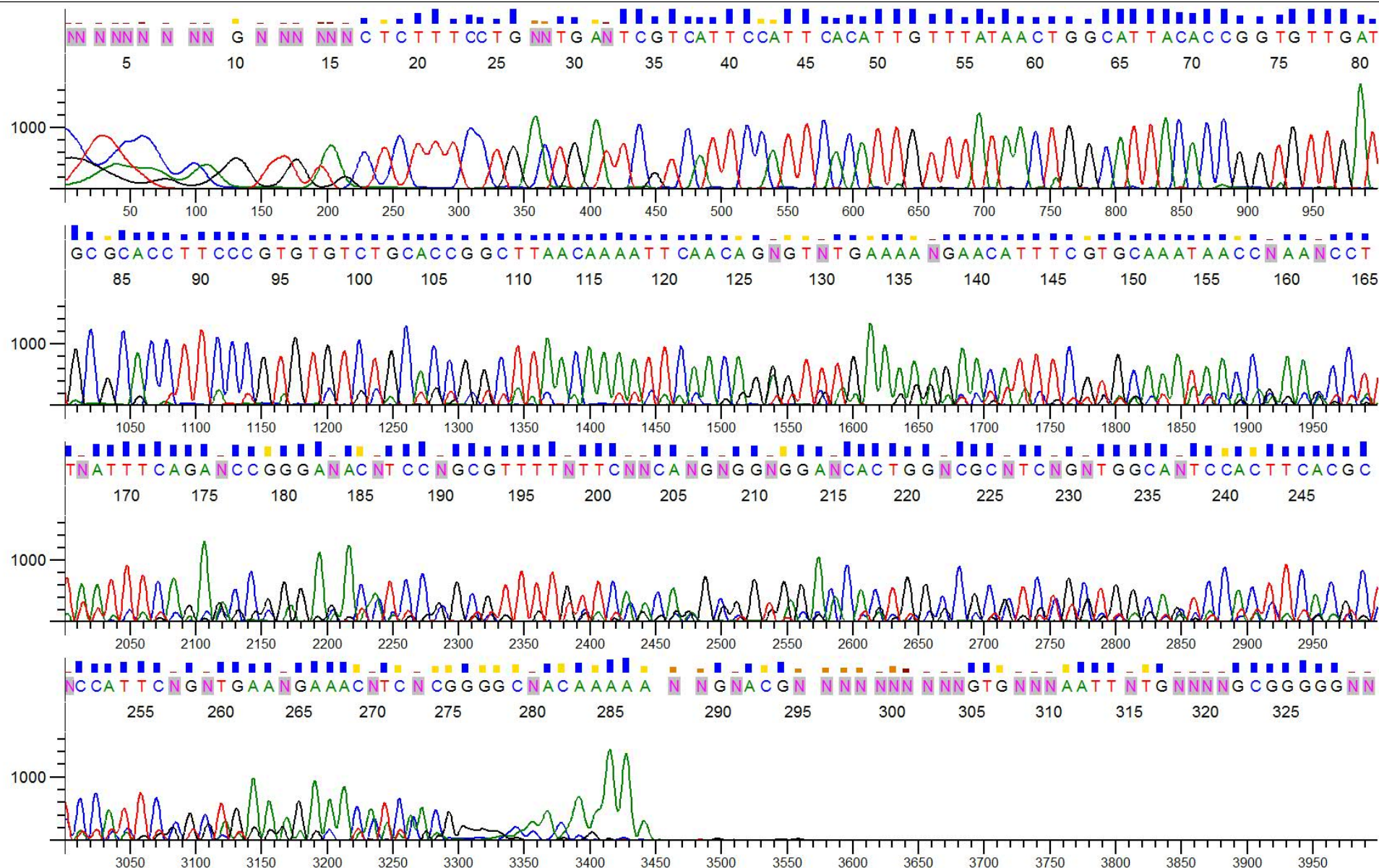

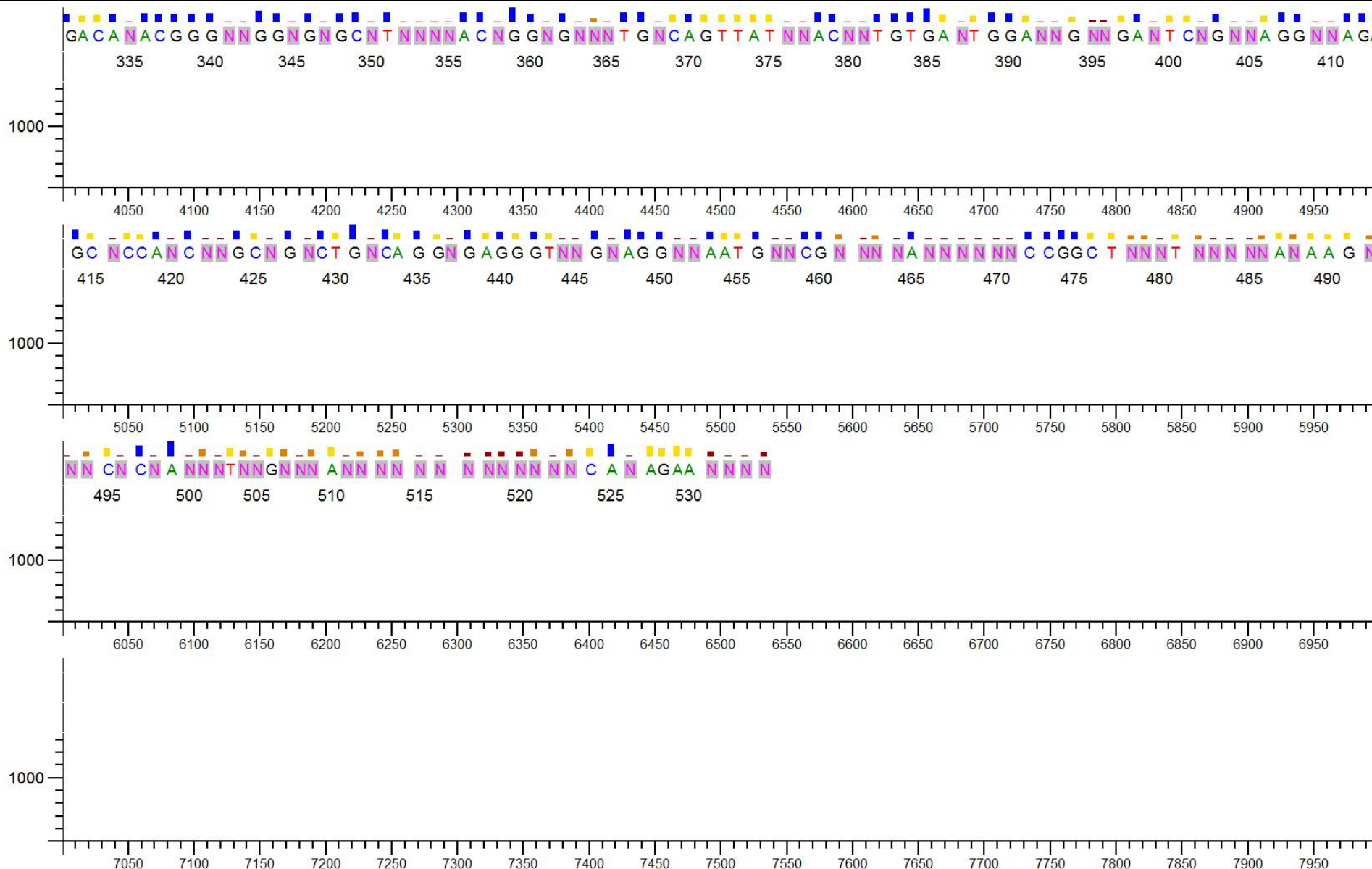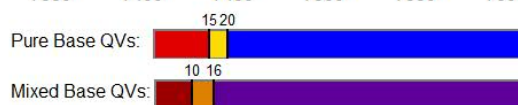

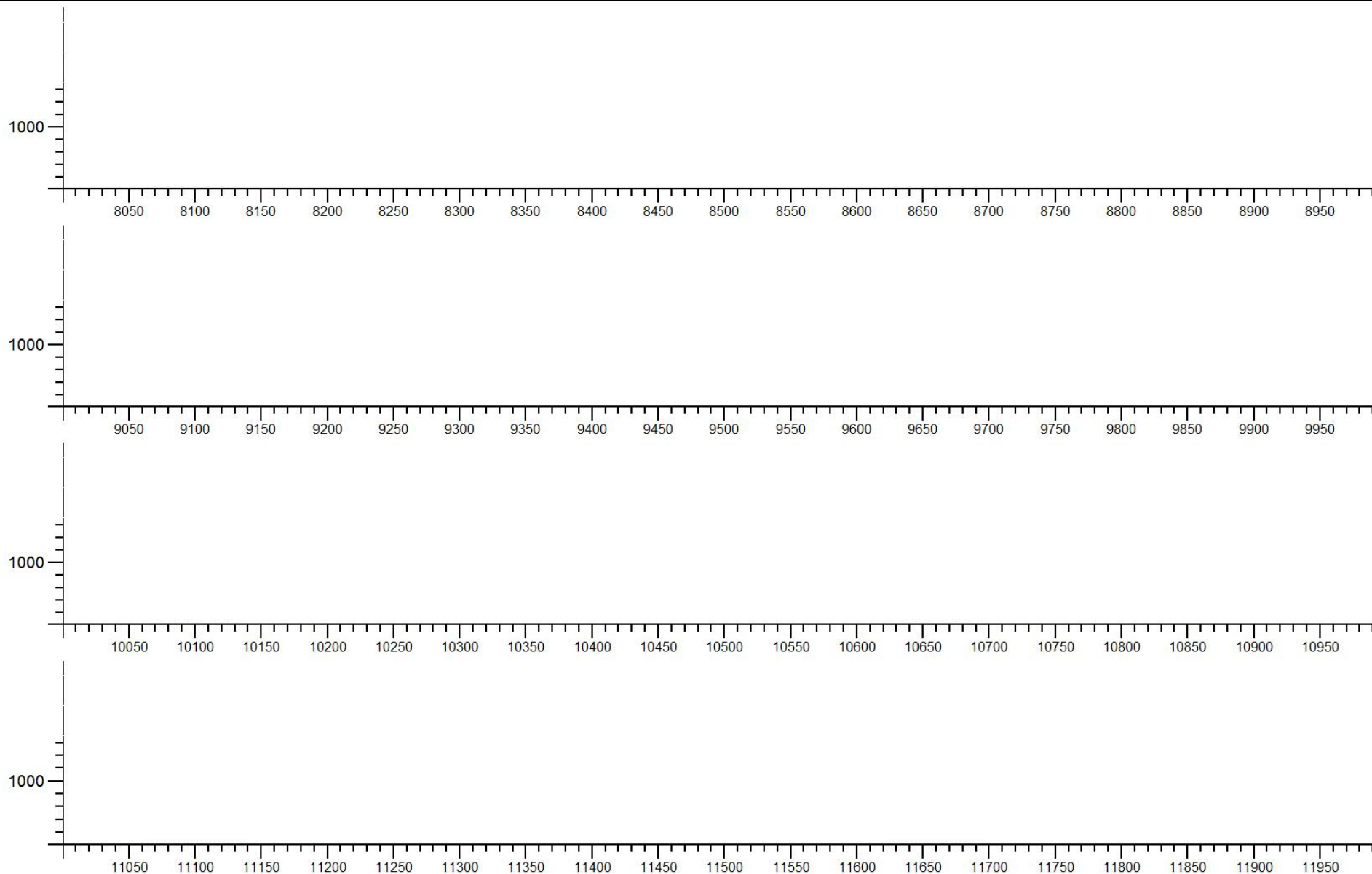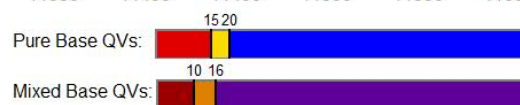

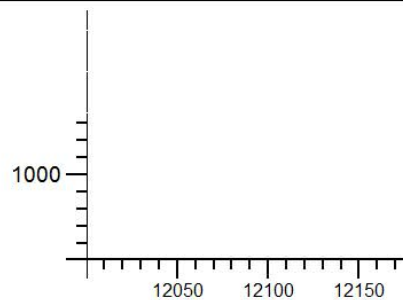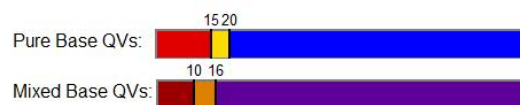

Supplement: Supplemental Information 1 — Chromatograms of: (1) recombined sequences of the H47 GI model from a number of mutants affected in recombination functions, and (2) recombined sequences of the pUYFRT model. [file peerj-05-3293-s001.zip › raw material/60-IntB_out1_FA.pdf]

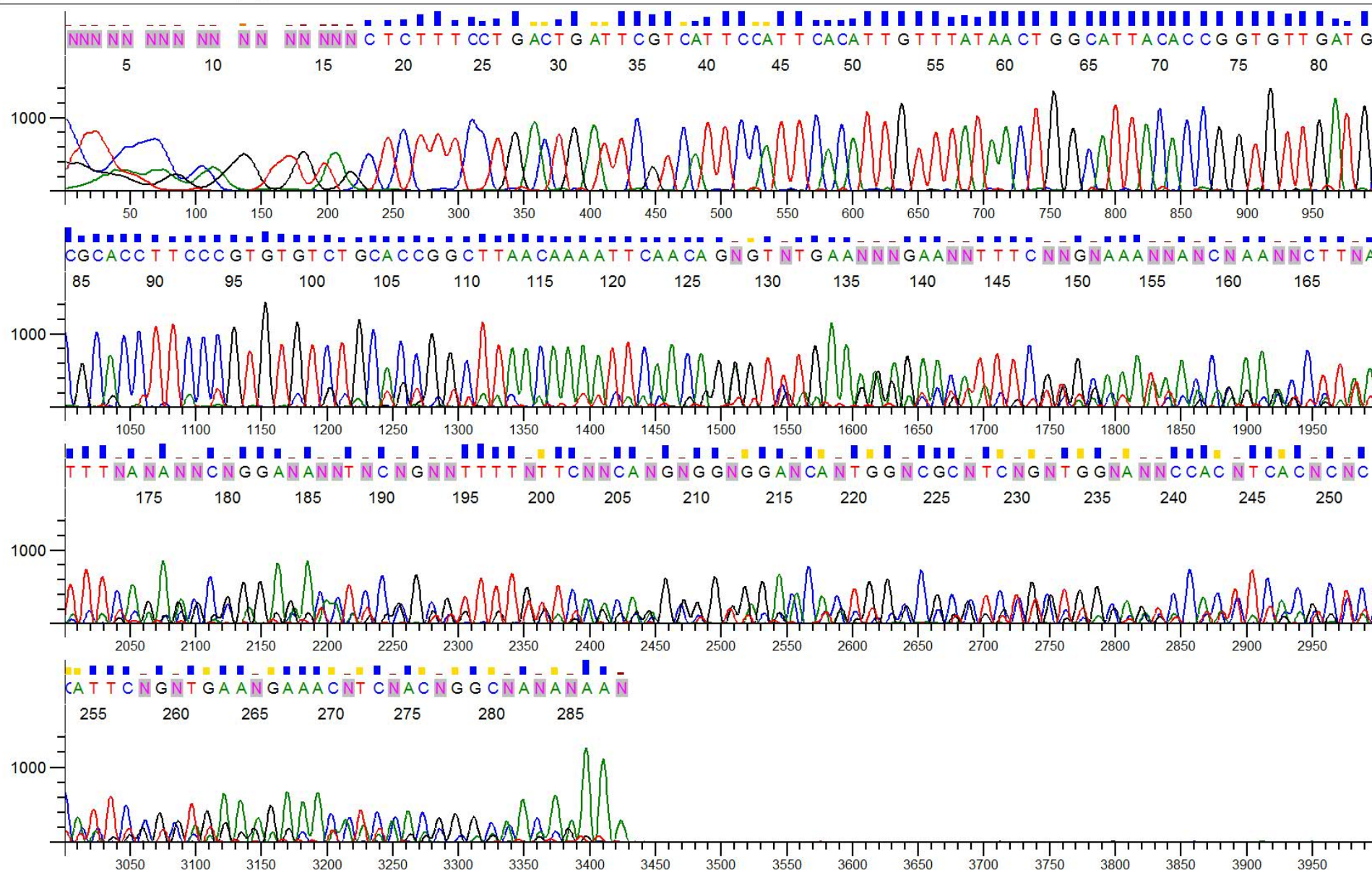

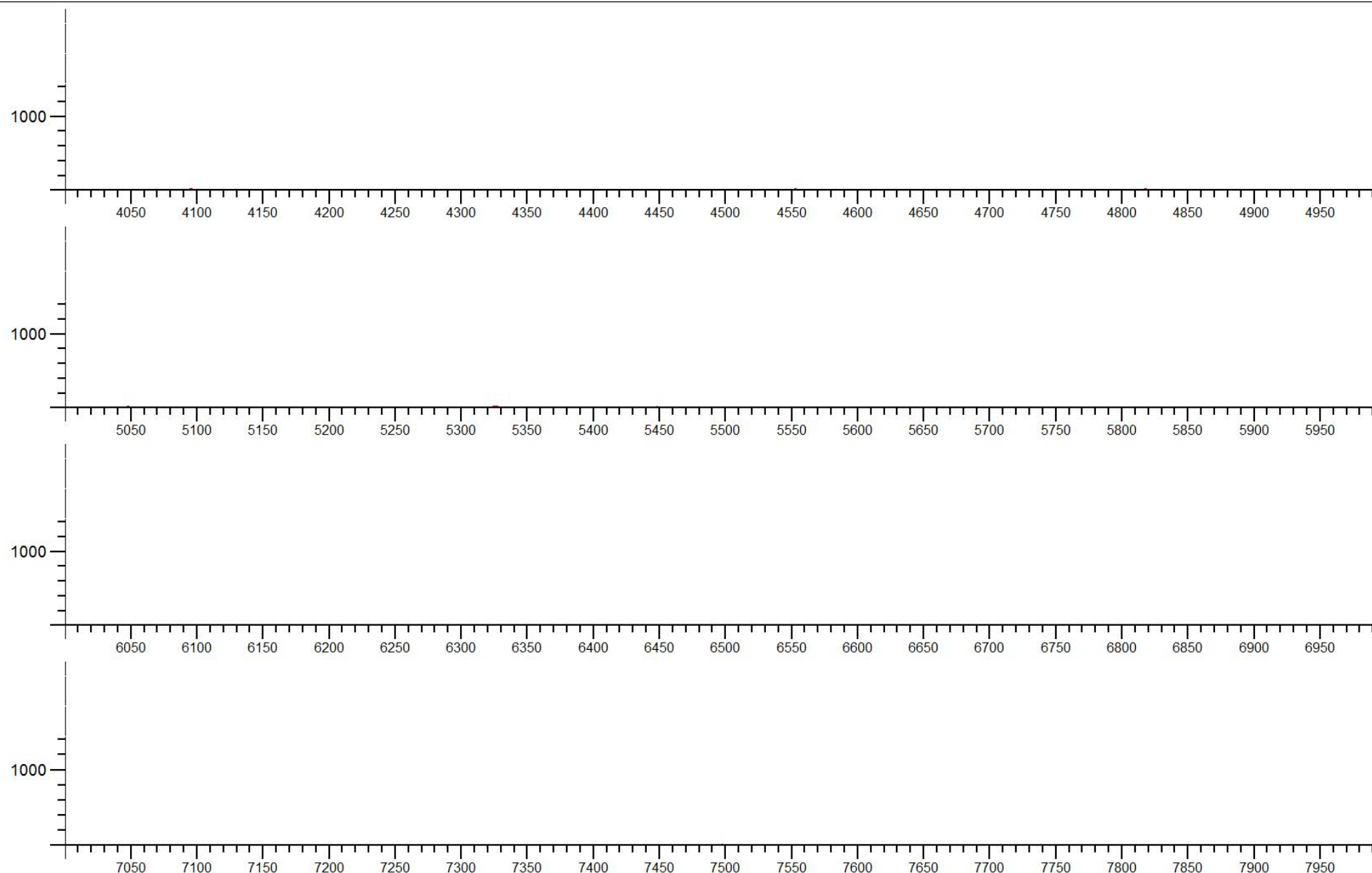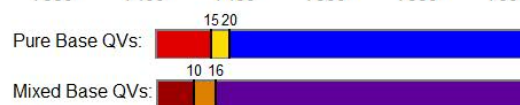

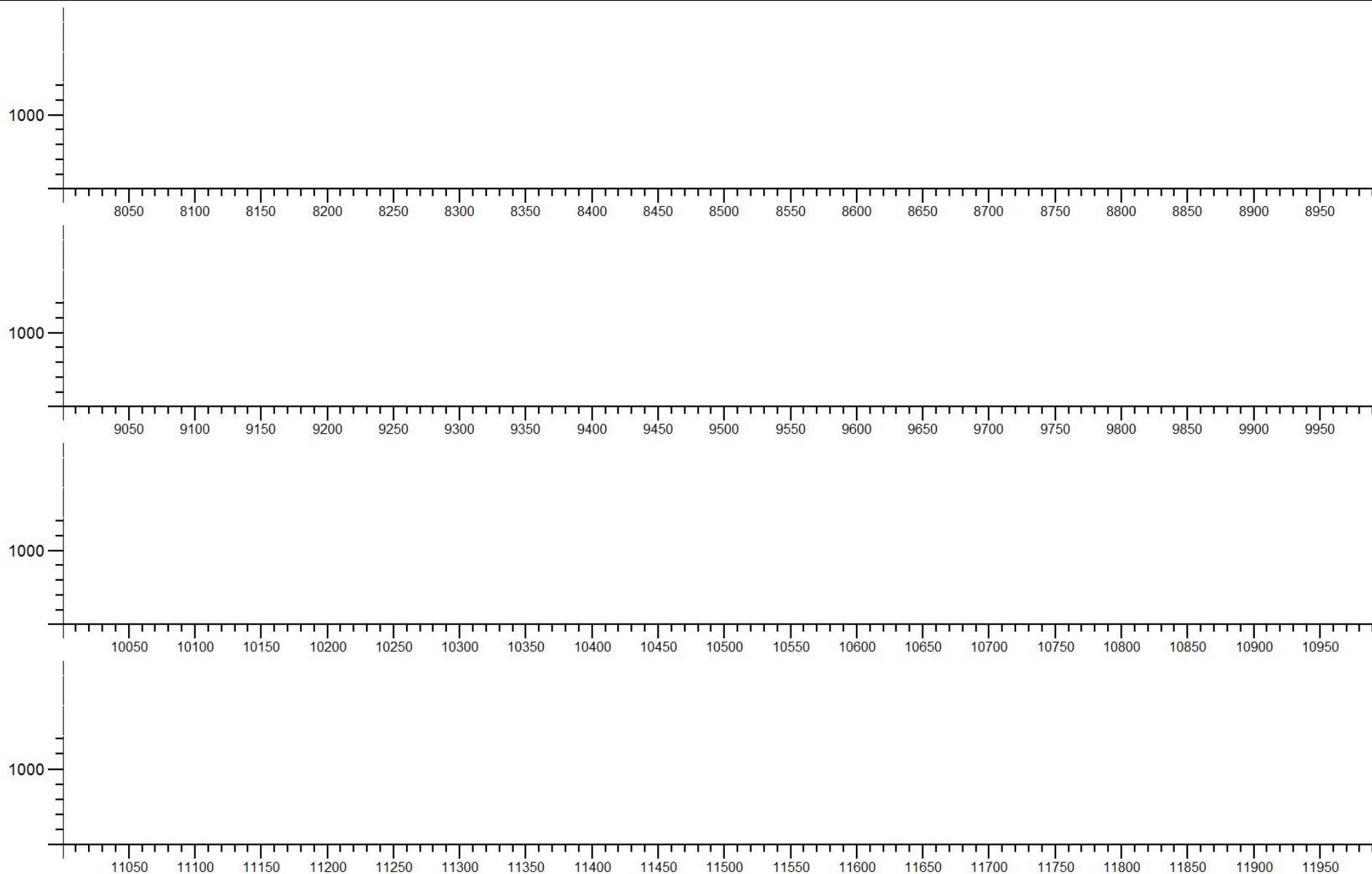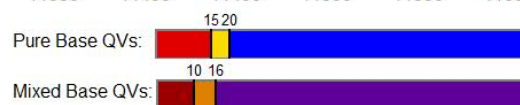

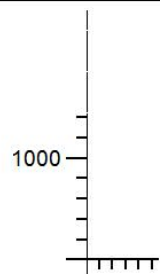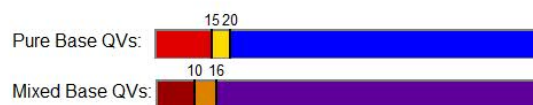

Supplement: Supplemental Information 1 — Chromatograms of: (1) recombined sequences of the H47 GI model from a number of mutants affected in recombination functions, and (2) recombined sequences of the pUYFRT model. [file peerj-05-3293-s001.zip › raw material/63-RecQ_out1_FA.pdf]

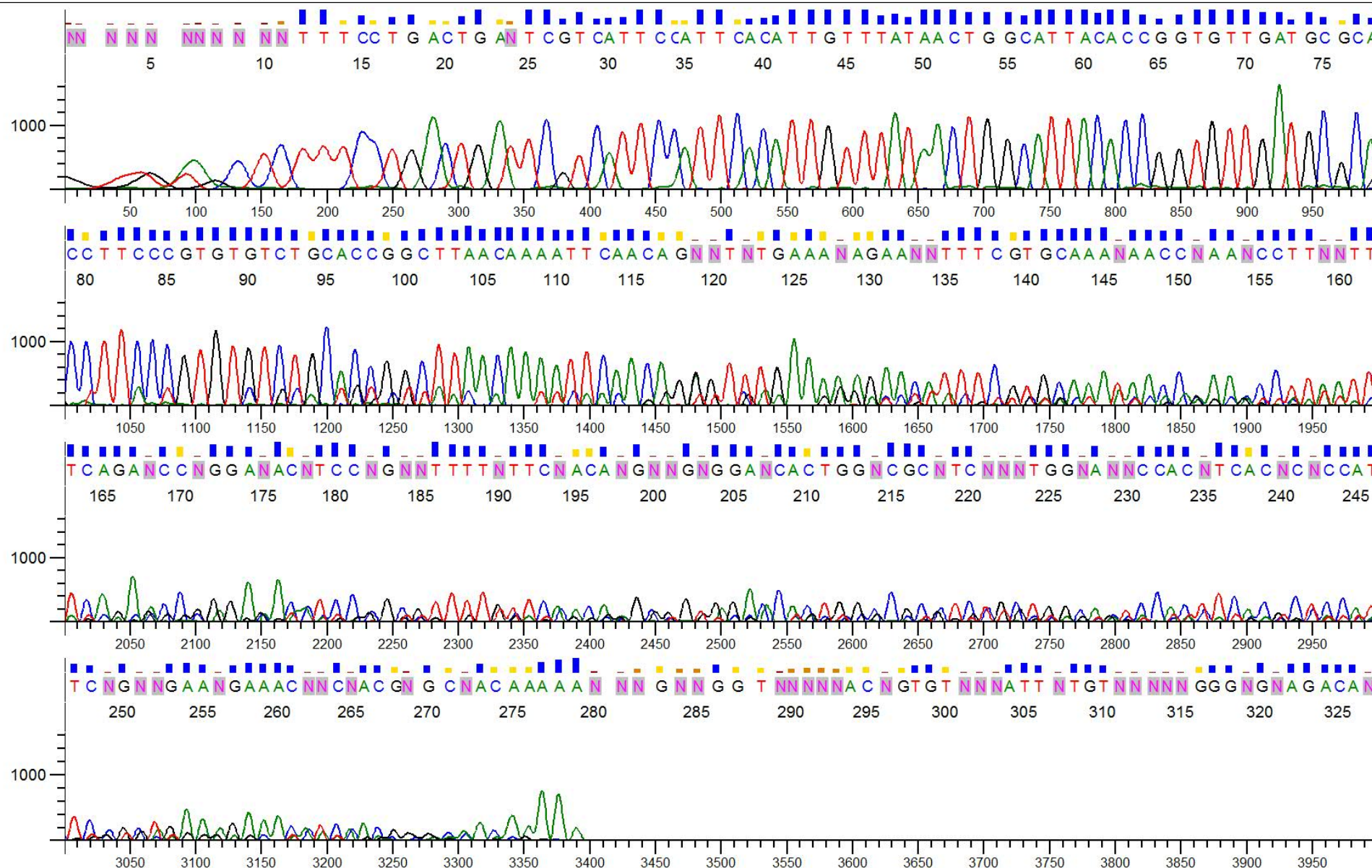

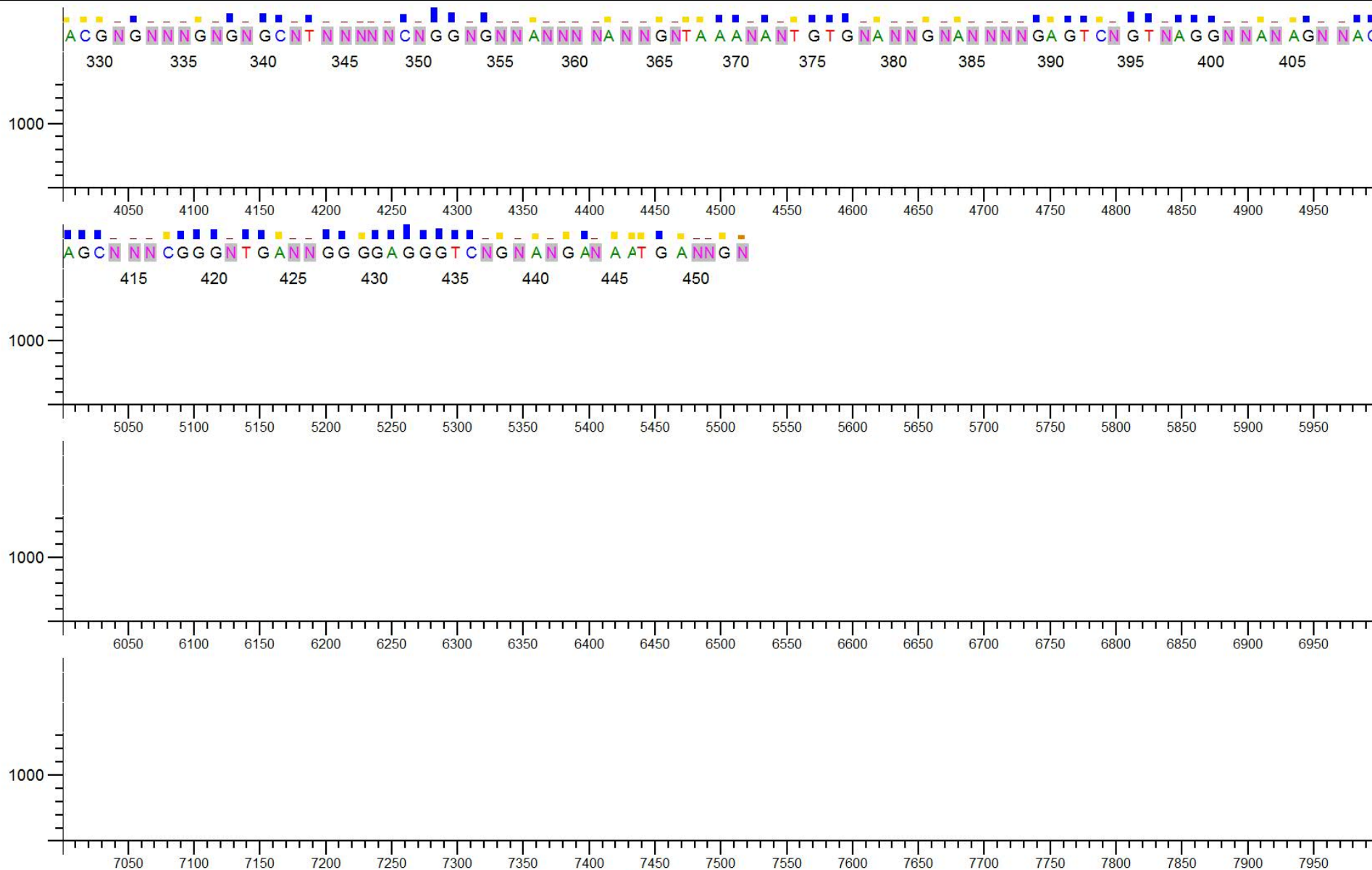

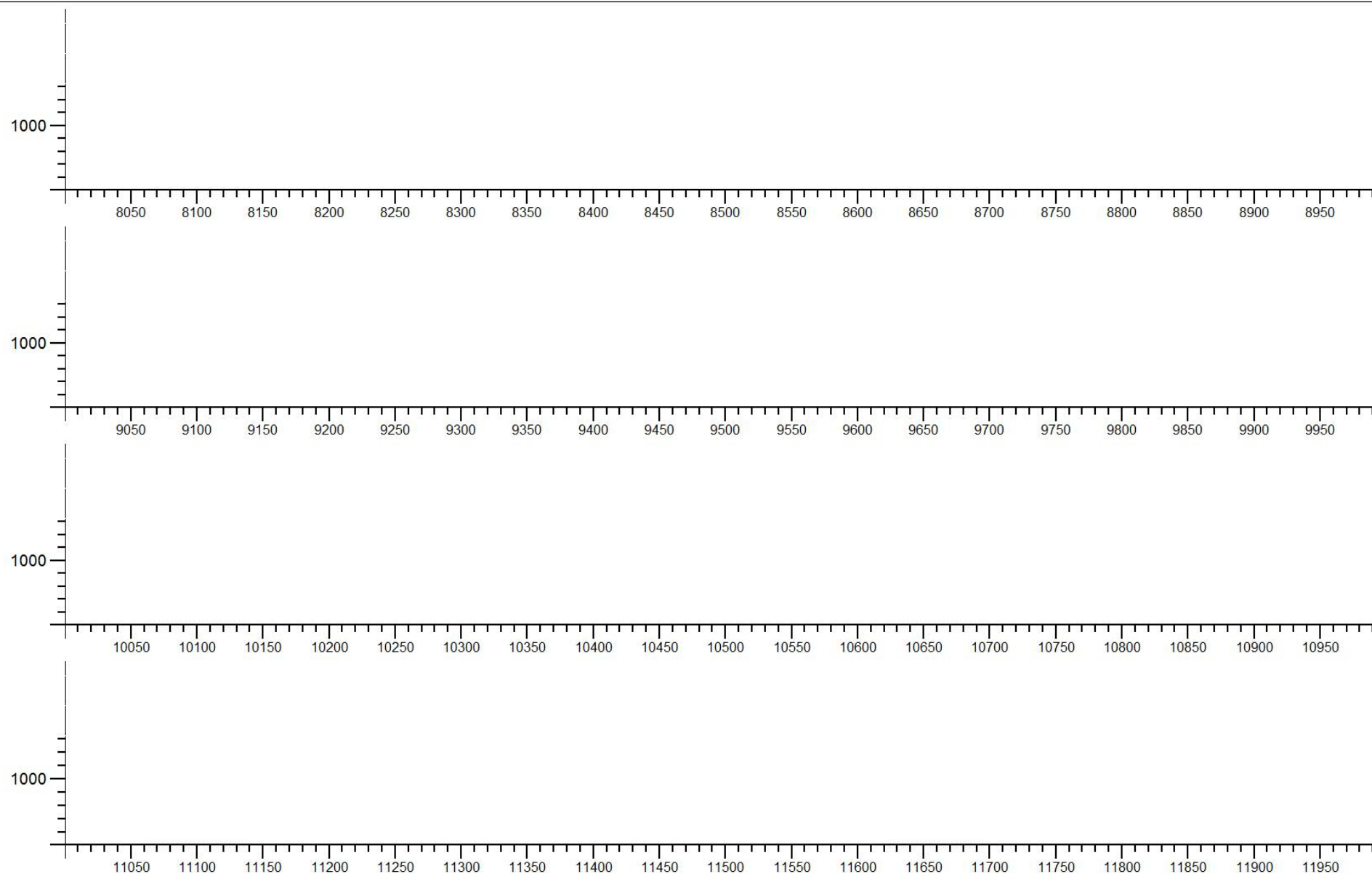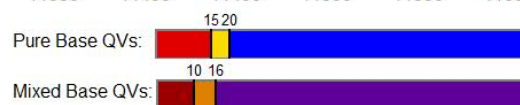

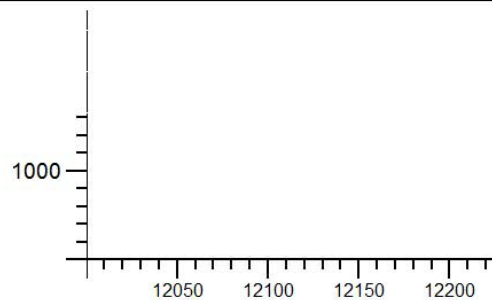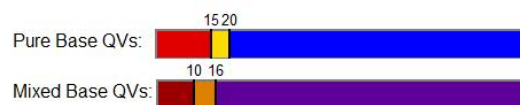

Supplement: Supplemental Information 1 — Chromatograms of: (1) recombined sequences of the H47 GI model from a number of mutants affected in recombination functions, and (2) recombined sequences of the pUYFRT model. [file peerj-05-3293-s001.zip › raw material/72_recTrecA_out1_FA.pdf]

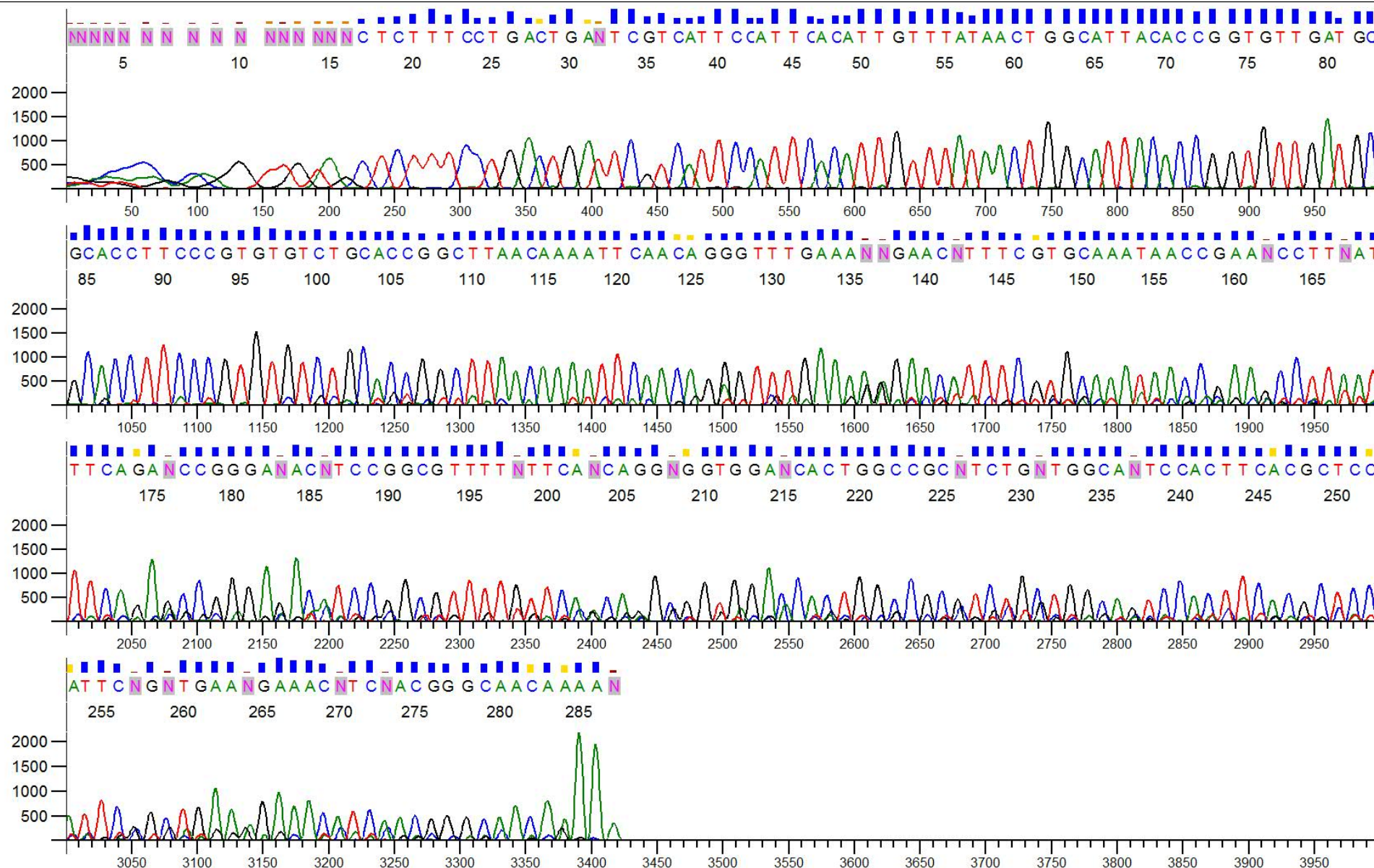

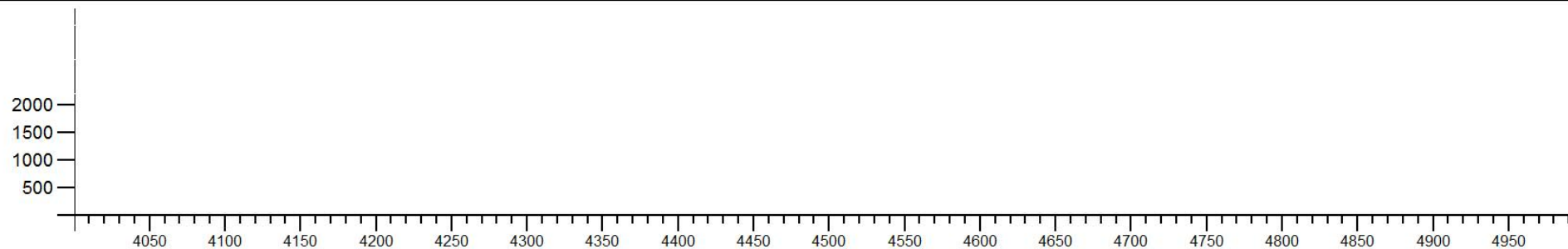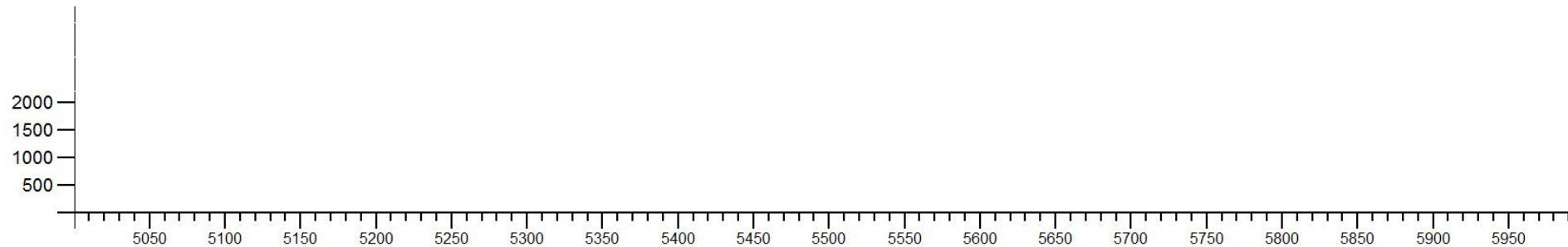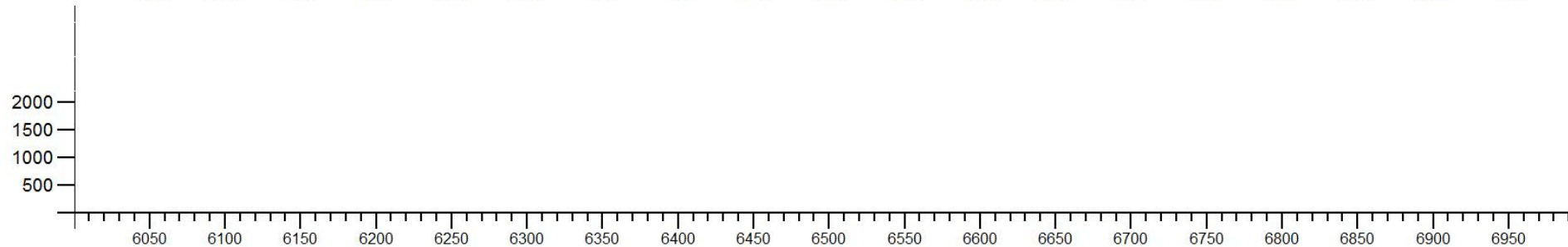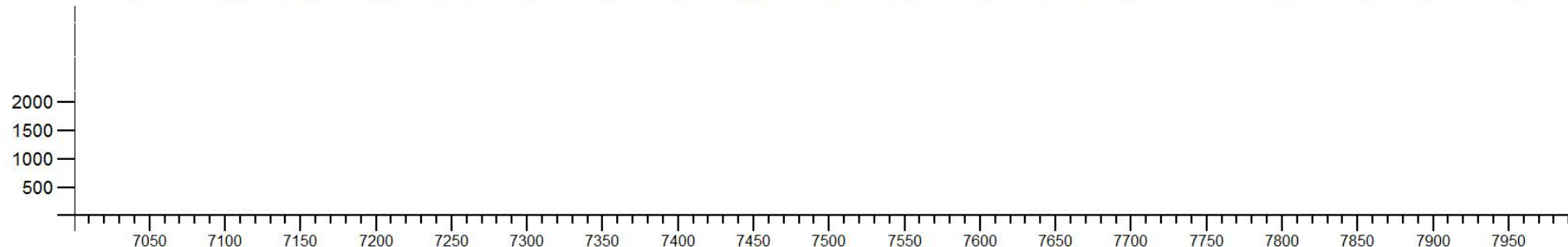

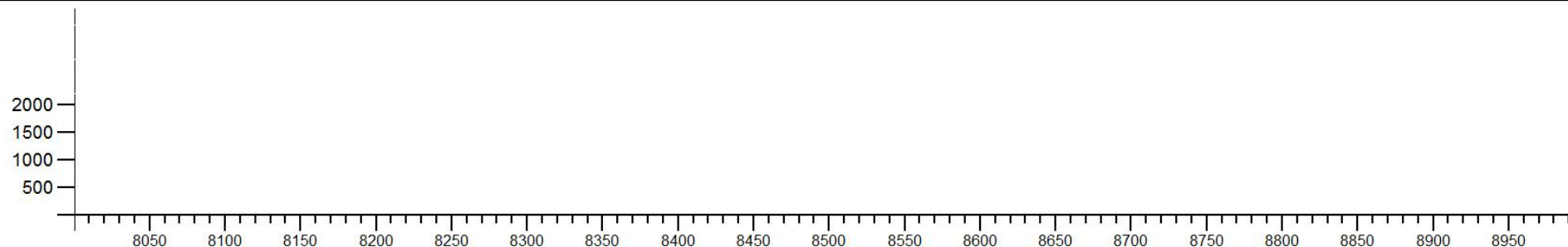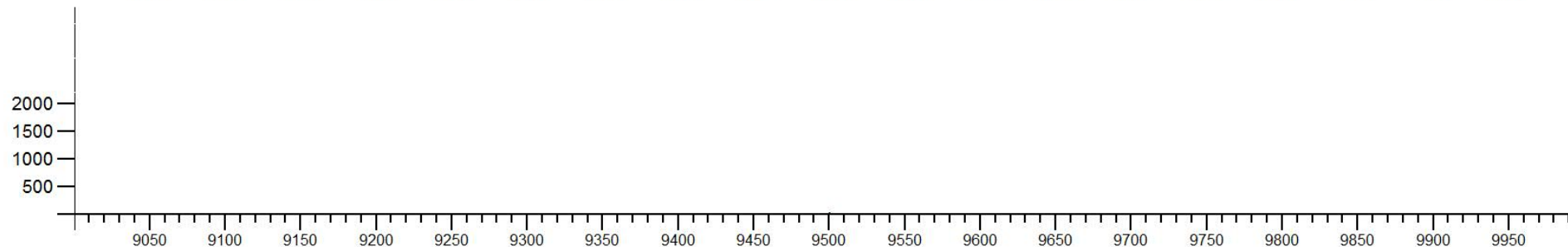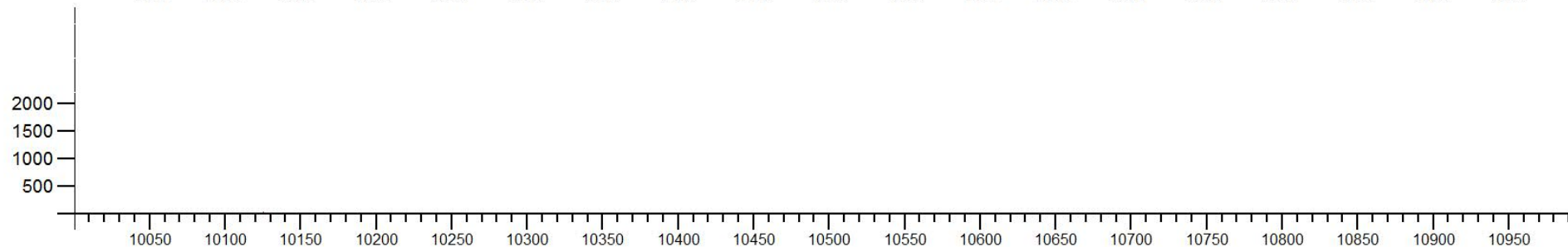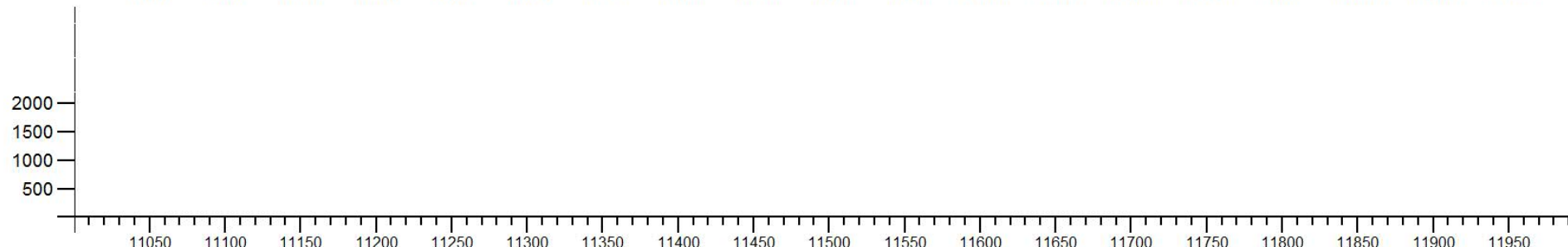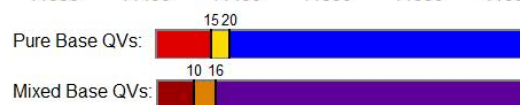

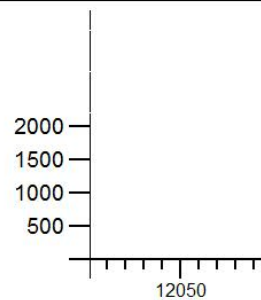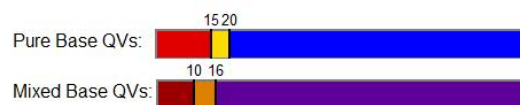

Supplement: Supplemental Information 1 — Chromatograms of: (1) recombined sequences of the H47 GI model from a number of mutants affected in recombination functions, and (2) recombined sequences of the pUYFRT model. [file peerj-05-3293-s001.zip › raw material/88-intSintBintFintA_out1_FA.pdf]

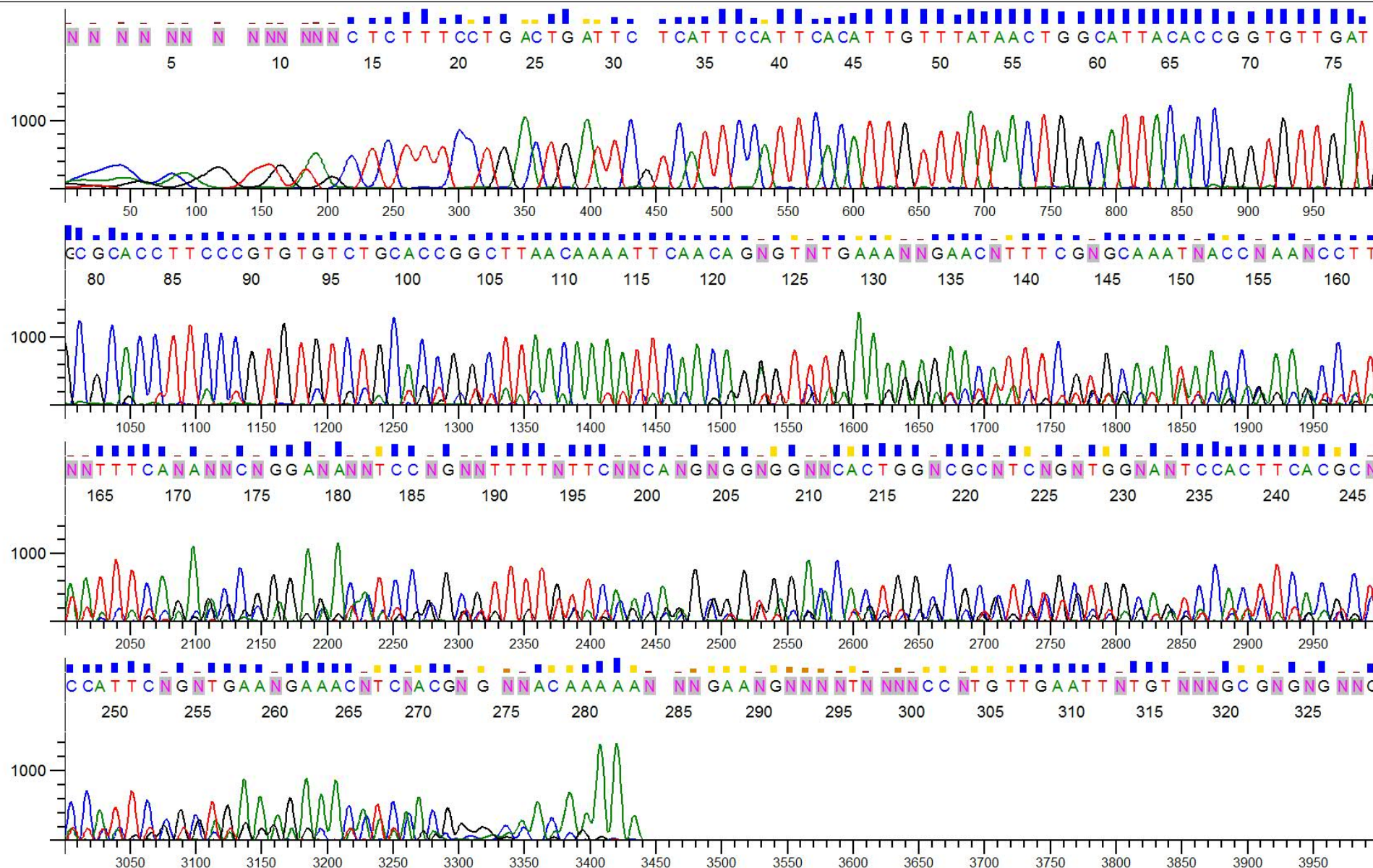

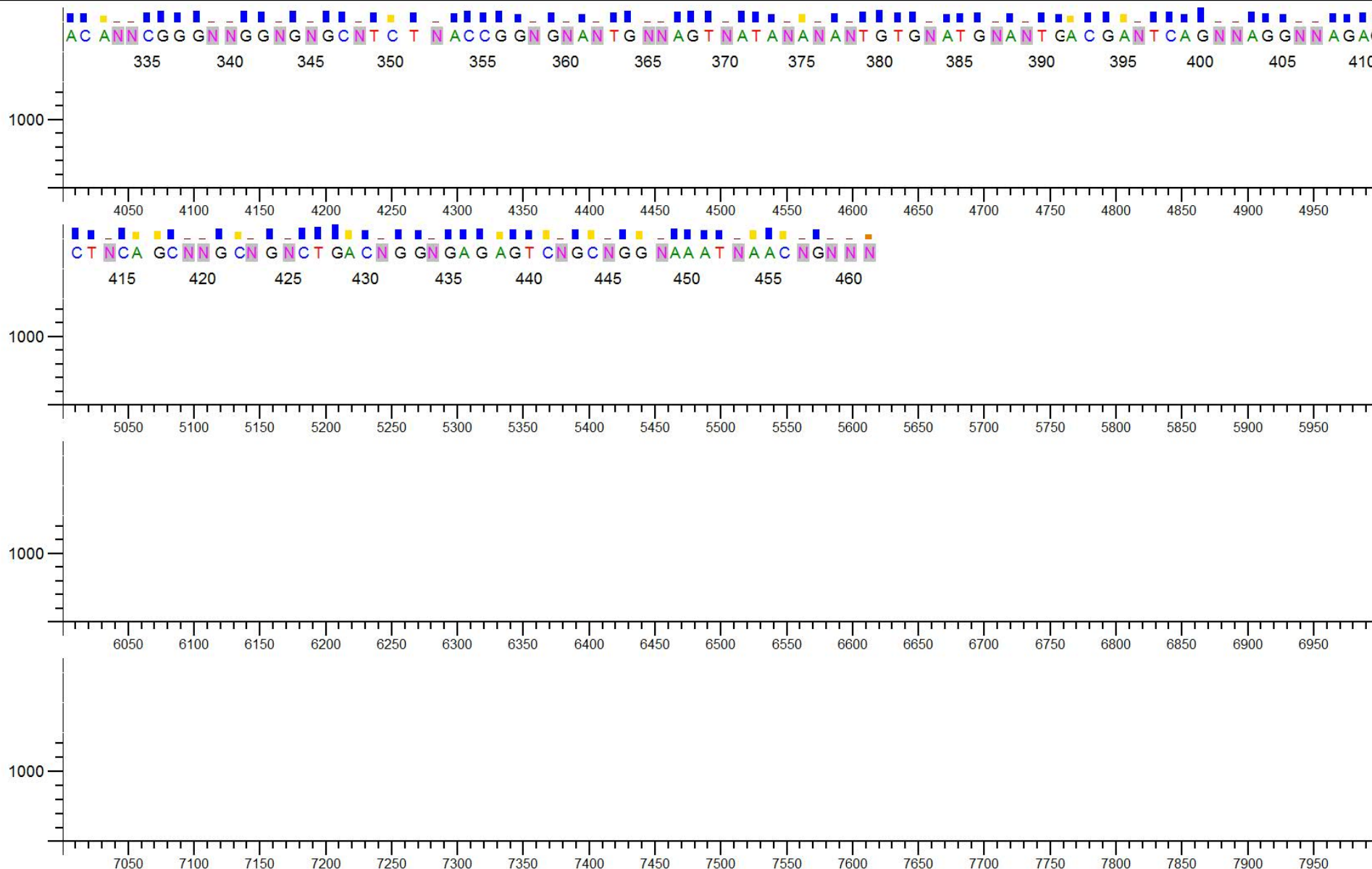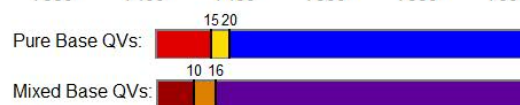

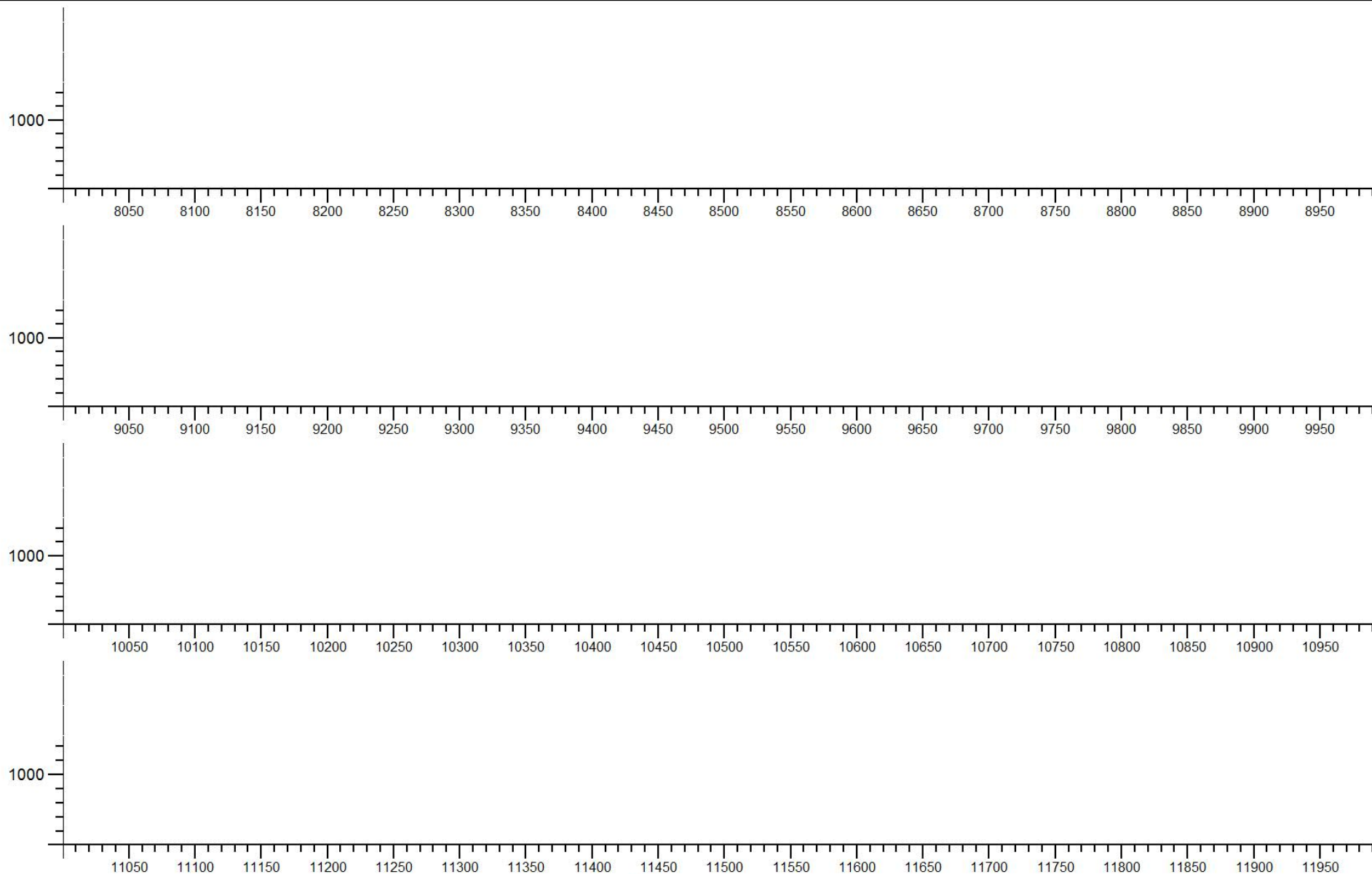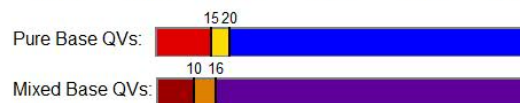

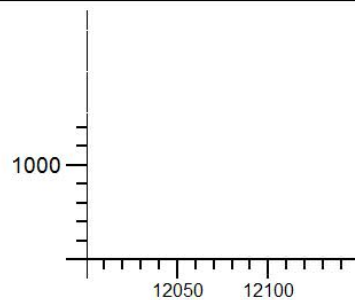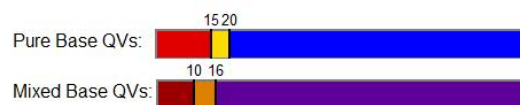

Supplement: Supplemental Information 1 — Chromatograms of: (1) recombined sequences of the H47 GI model from a number of mutants affected in recombination functions, and (2) recombined sequences of the pUYFRT model. [file peerj-05-3293-s001.zip › raw material/96-RecA_out1_FA.pdf]

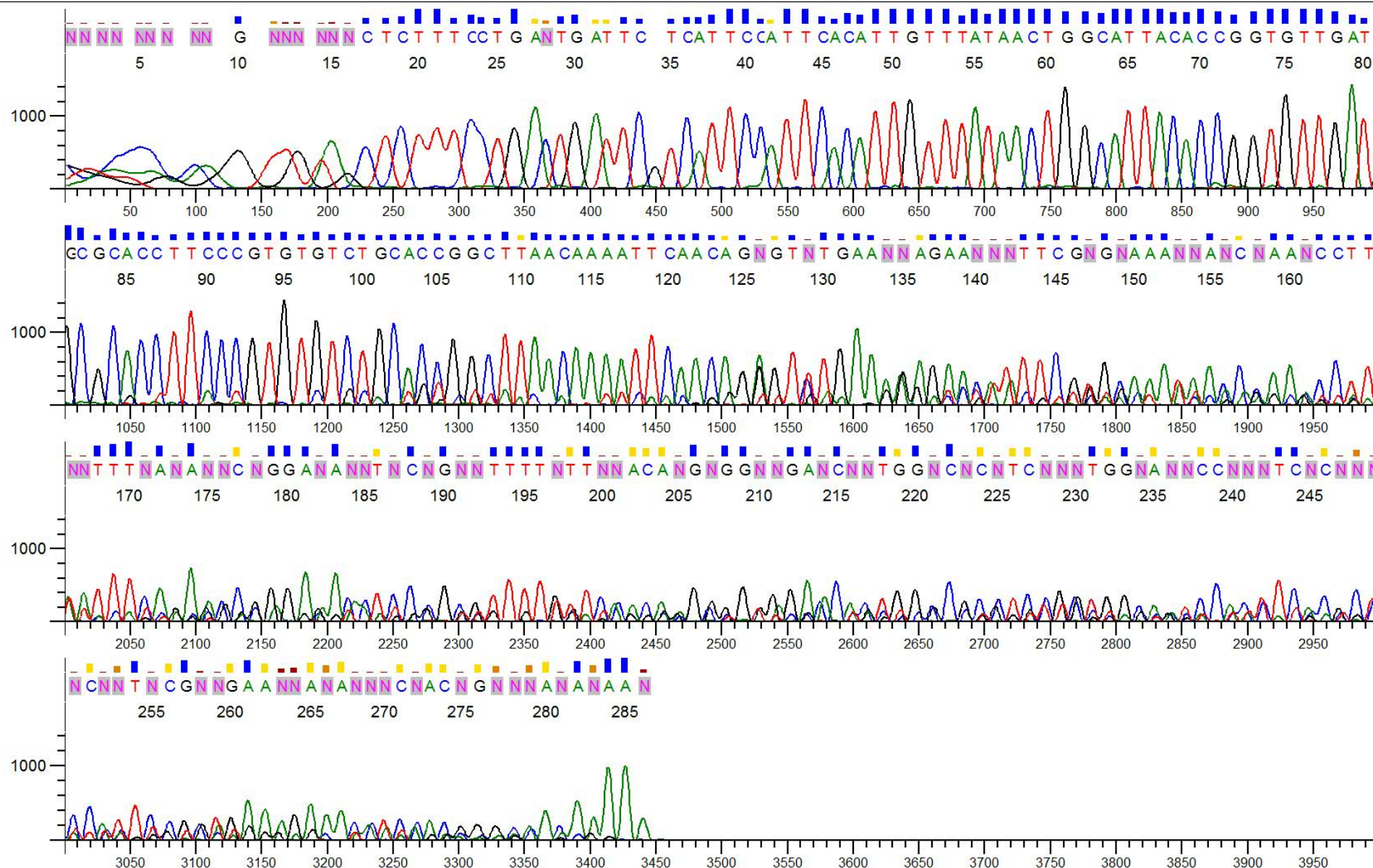

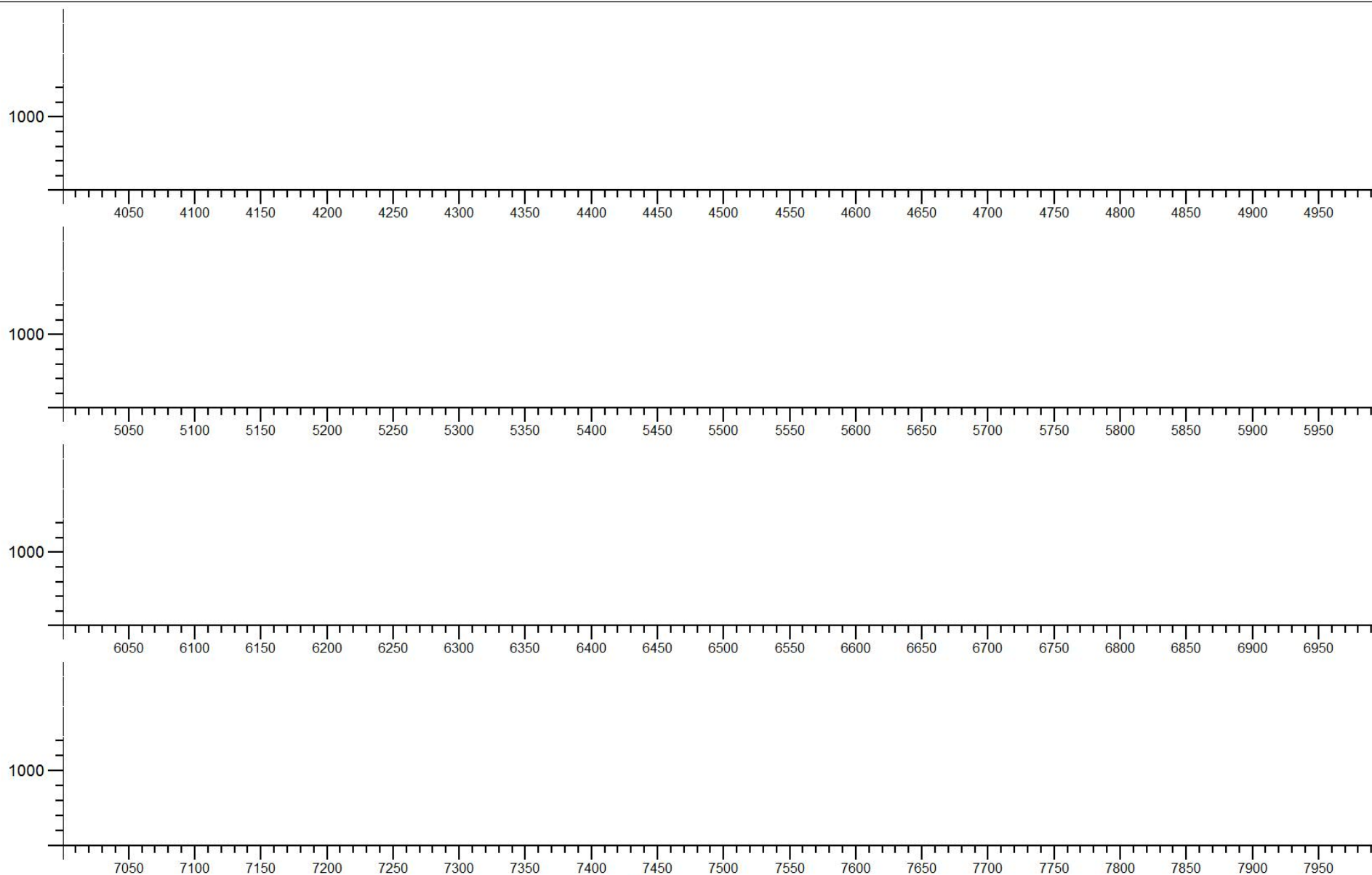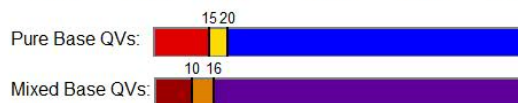

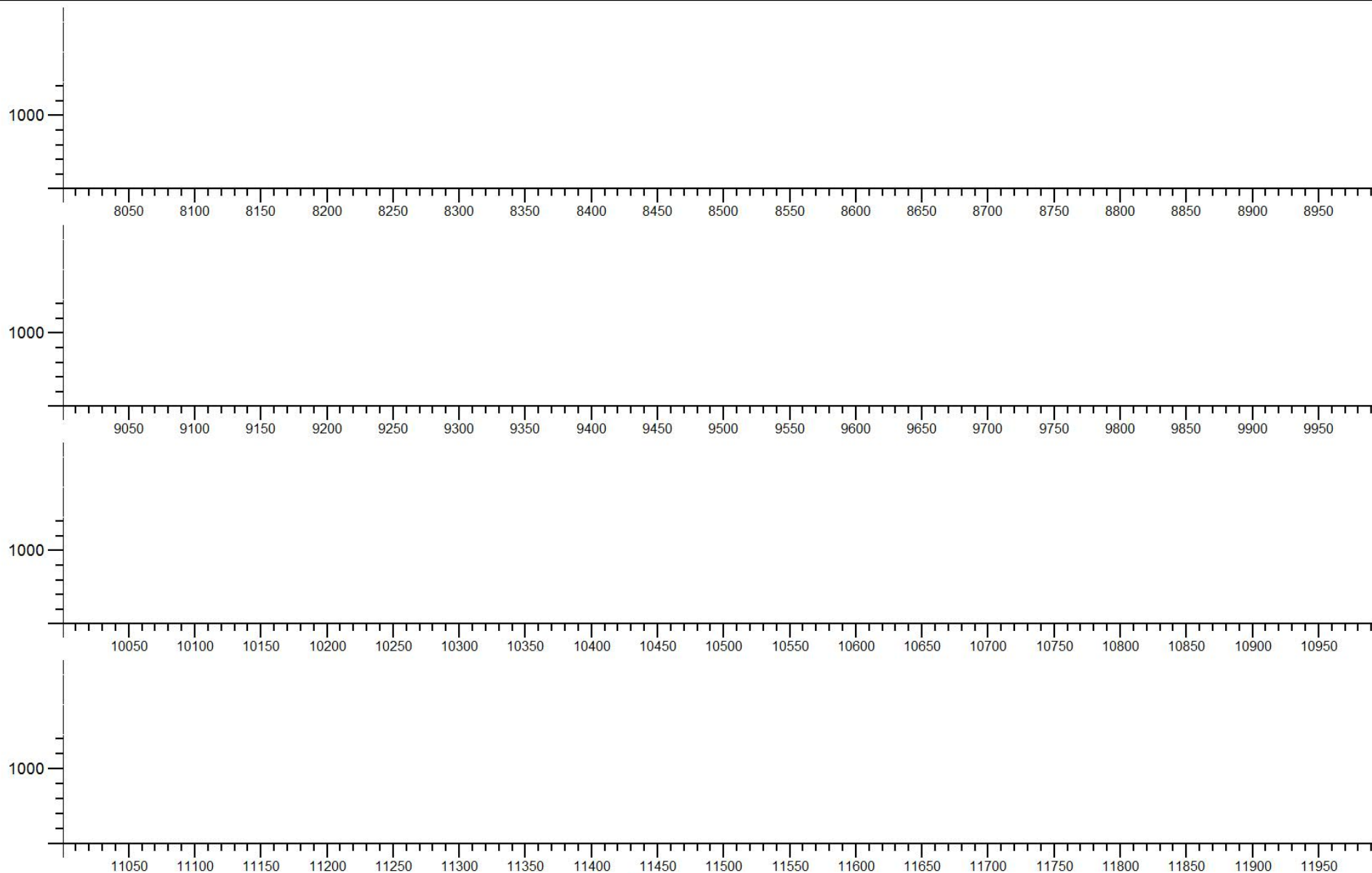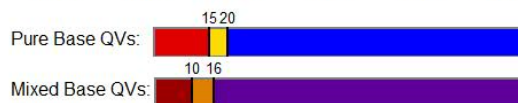

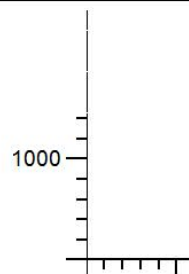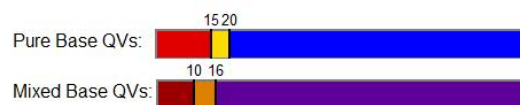

Supplement: Supplemental Information 1 — Chromatograms of: (1) recombined sequences of the H47 GI model from a number of mutants affected in recombination functions, and (2) recombined sequences of the pUYFRT model. [file peerj-05-3293-s001.zip › raw material/96-RecF_out1_FA.pdf]

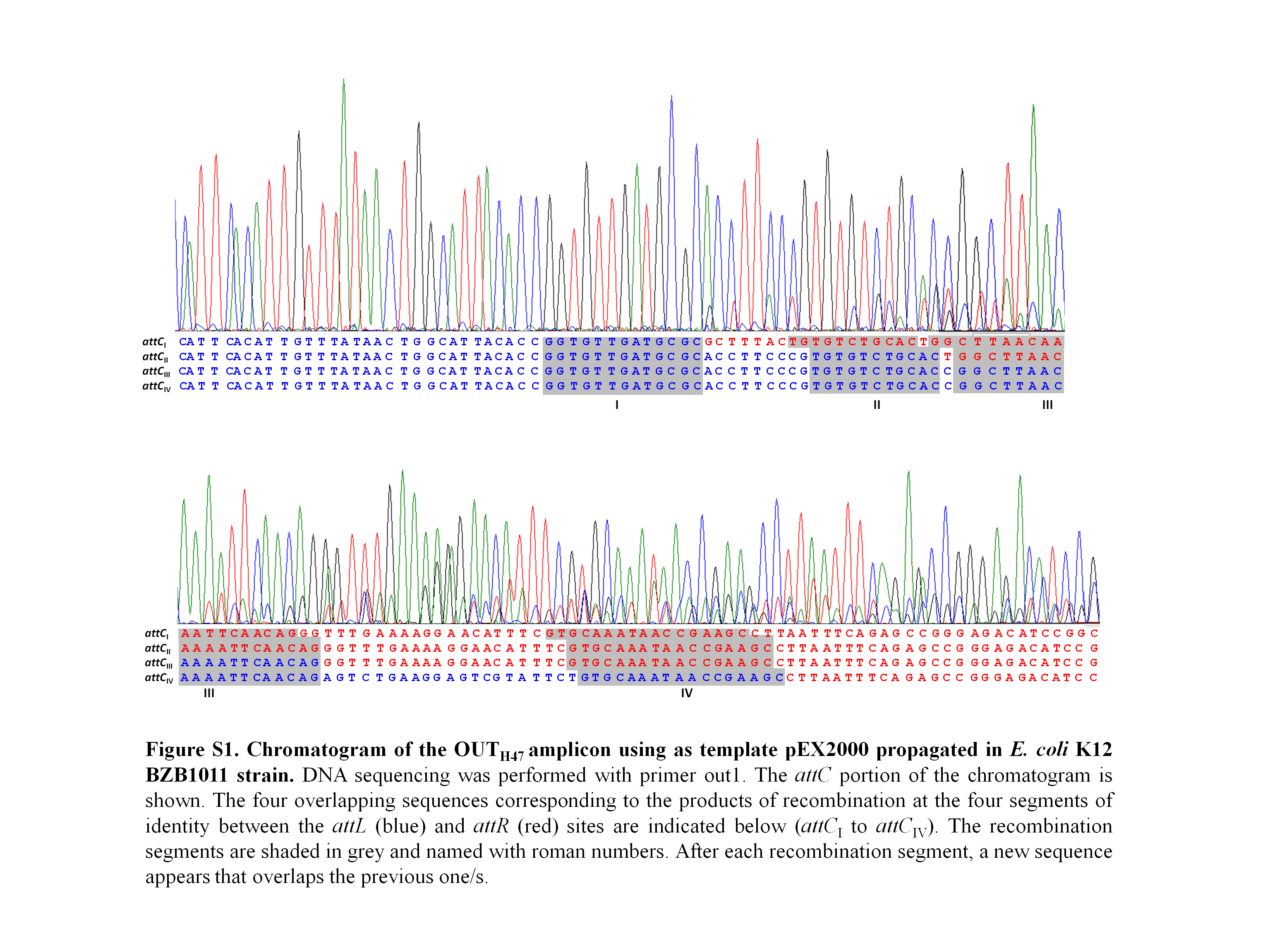

Supplement: Figure S1 — DNA sequencing was performed with primer out1. The attC portion of the chromatogram is shown. The four overlapping sequences corresponding to the products of recombination at the four segments of identity between the attL (blue) and attR (red) sites are indicated below (attC_I to attC_IV). The recombination segments are shaded in grey and named with roman numbers. After each recombination segment, a new sequence appears that overlaps the previous one/s. [file peerj-05-3293-s002.png]
