# Supplementary material for: Analysis of RecA-independent recombination events between short direct repeats related to a genomic island and to a plasmid in Escherichia coli K12
Source: PeerJ. 2017 May 9;5:e3293. doi: 10.7717/peerj.3293 (PMC5426353; doi:10.7717/peerj.3293)
Supplement: Table S1 [file peerj-05-3293-s003.docx]

**Table S1.** **Deletion mutations carried by *E. coli* K12 BZB1011 derivative strains**

| **Mutant alleles** ^(a)^ | **Keio knockout strains**  **carrying the allele** |
| --- | --- |
| *ΔpinQ766::kan* | JW1538 ^(b)^ |
| *ΔpinR737::kan* | JW1368 ^(b)^ |
| *ΔybcK752::kan* | JW0532 ^(b)^ |
| *ΔpinQ766 ΔpinR737 ΔybcK752::kan* | JW1538, JW1368 and JW0532 |
| *Δpin-746::kan* | JW1144 ^(b)^ |
| *ΔintS756::kan* | JW2345 ^(b)^ |
| *ΔintB741::kan* | JW4227 ^(b)^ |
| *ΔintF725::kan* | JW0275 ^(b)^ |
| *ΔintA777::kan* | JW2602 ^(b)^ |
| *ΔintS756 ΔintB741 ΔintF725 ΔintA777::kan* | JW2345, JW4227, JW0275 and JW2602 |
| *ΔintD746::kan* | JW0525 ^(b)^ |
| *ΔintR772::kan* | JW1339 ^(b)^ |
| *ΔintD746 ΔintR772::kan* | JW0525 and JW1339 |
| *ΔintQ726::kan* | JW1571 ^(b)^ |
| *ΔintD746 ΔintQ726::kan* | JW0525 and JW1571 |
| *ΔintR772 ΔintQ726::kan* | JW1339 and JW1571 |
| *ΔintE729::kan* | JW1126 ^(b)^ |
| *ΔintD746 ΔintA777::kan* | JW0525 and JW2602 |
| *ΔintA777 ΔintQ726::kan* | JW2602 and JW1571 |
| *ΔintR772 ΔintA777::kan* | JW1339 and JW2602 |
| *ΔxerC757::kan* | JW3784 ^(b)^ |
| *ΔxerD745::kan* | JW2862 ^(b)^ |
| *ΔxerC757 ΔxerD745::kan* | JW3784 and JW2862 |
| *ΔfimB780::kan* | JW4275 ^(b)^ |
| *ΔfimE781::kan* | JW4276 ^(b)^ |
| *ΔfimB780 ΔfimE781::kan* | JW4275 and JW4276 |
| *ΔrecE787::kan* | JW1344 ^(b)^ |
| *ΔrecT786::kan* ^(d)^ | JW1343 ^(b)^ |
| *ΔrecA774::kan* ^(d)^ | JW2669 ^(c)^ |
| *ΔrecT786 ΔrecA774::kan* | JW1343 and JW2669 |
| *ΔtopB761::kan* | JW1752 ^(c)^ |
| *ΔrecB745::kan* | JW2788 ^(c)^ |
| *ΔrecC747::kan* | JW2790 ^(c)^ |
| *ΔrecD744::kan* | JW2787 ^(c)^ |
| *ΔrecF735::kan* | JW3677 ^(c)^ |
| *ΔrecR776::kan* | JW0461 ^(c)^ |
| *ΔrecO737::kan* | JW2549 ^(c)^ |
| *ΔrecJ743::kan* | JW2860 ^(c)^ |
| *ΔrecN772::kan* | JW5416 ^(c)^ |
| *ΔrecQ767::kan* | JW5855 ^(c)^ |
| *ΔrecG756::kan* | JW3627 ^(c)^ |
| *ΔsbcB780::kan* | JW1993 ^(c)^ |
| *ΔsbcC761::kan* | JW0387 ^(c)^ |
| *ΔsbcD762::kan* | JW0388 ^(c)^ |
| *ΔruvA786::kan* | JW1850 ^(c)^ |
| *ΔruvB785::kan* | JW1849 ^(c)^ |
| *ΔruvC789::kan* | JW1852 ^(c)^ |
| *ΔrecX773::kan* | JW2668 ^(c)^ |
| *ΔradA785::kan* | JW4352 ^(c)^ |
| *ΔexoX769::kan* | JW1833 ^(c)^ |
| *ΔrarA788::kan* | JW0875 ^(c)^ |
| *ΔseqA735::kan* | JW0674 ^(c)^ |
| *ΔhelD777::kan* | JW0945 ^(c)^ |
| *ΔuvrD769::kan* | JW3786 ^(c)^ |

^(a)^ Allele numbers following the Coli Genetic Stock Center

^(b)^ Provided by NBRP (NIG, Japan): E. coli

^(c)^ Provided by the Coli Genetic Stock Center

^(d)^ Deletion mutations confirmed by PCR using primers external to the genes
